# Supplementary figures and images for: Tracing the evolution of aneuploid cancers by multiregional sequencing with CRUST
Source: Brief Bioinform. 2021 Aug 3;22(6):bbab292. doi: 10.1093/bib/bbab292 (PMC8981300; doi:10.1093/bib/bbab292)

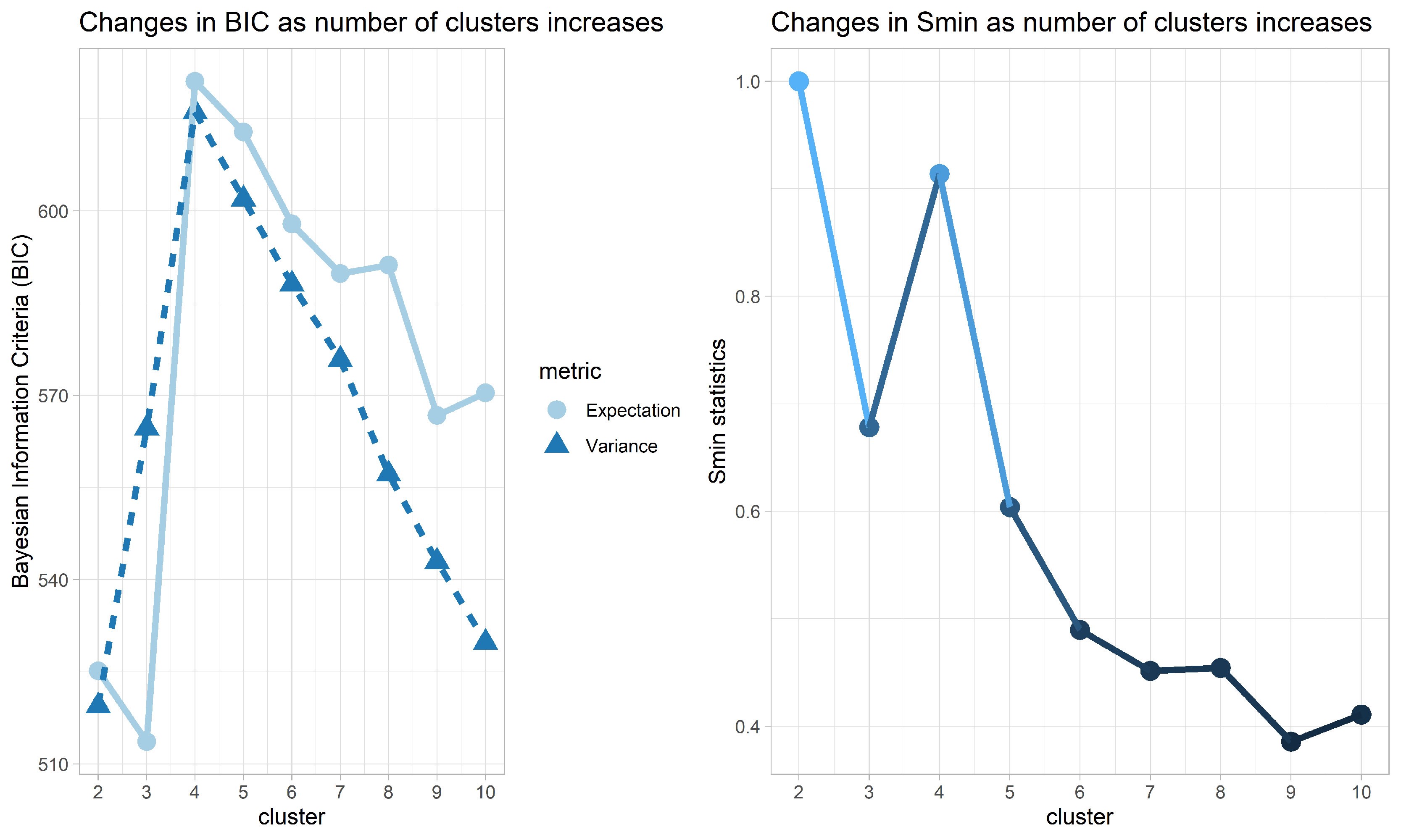

Supplement: Supplementary_Figure_1_bbab292 [file supplementary_figure_1_bbab292.jpeg]

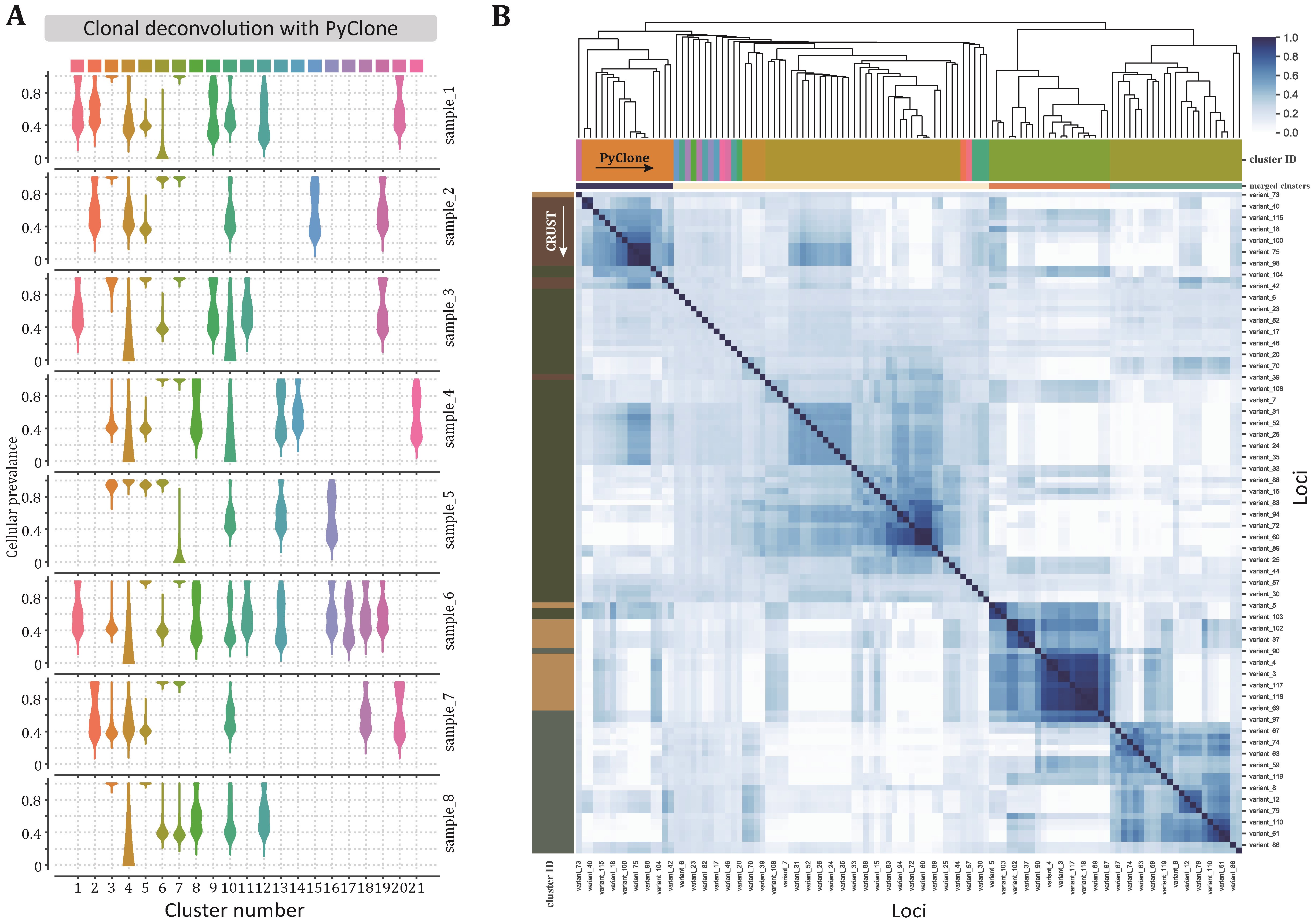

Supplement: Supplementary_Figure_3_bbab292 [file supplementary_figure_3_bbab292.jpeg]

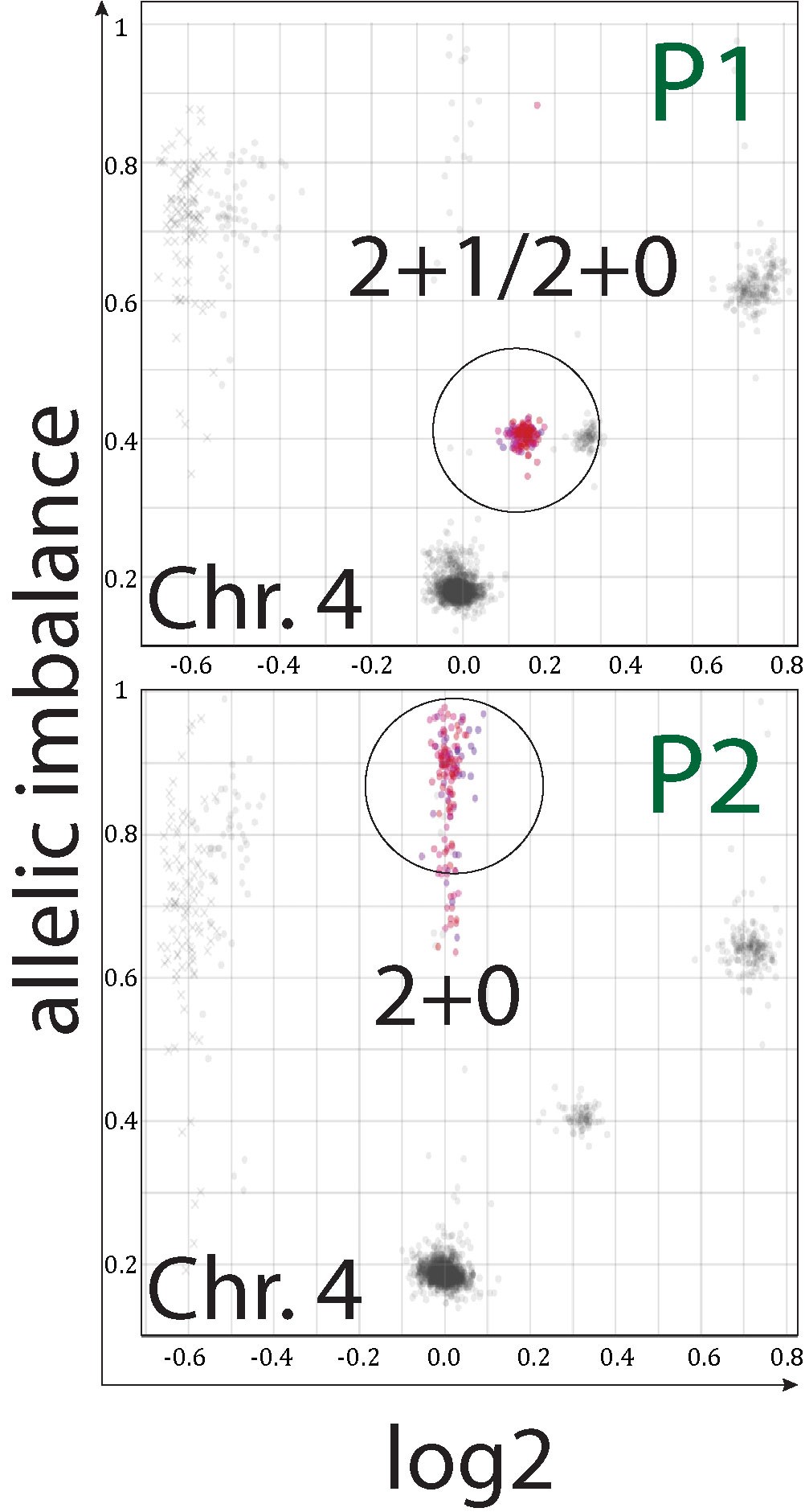

Supplement: Supplementary_Figure_4_bbab292 [file supplementary_figure_4_bbab292.jpeg]

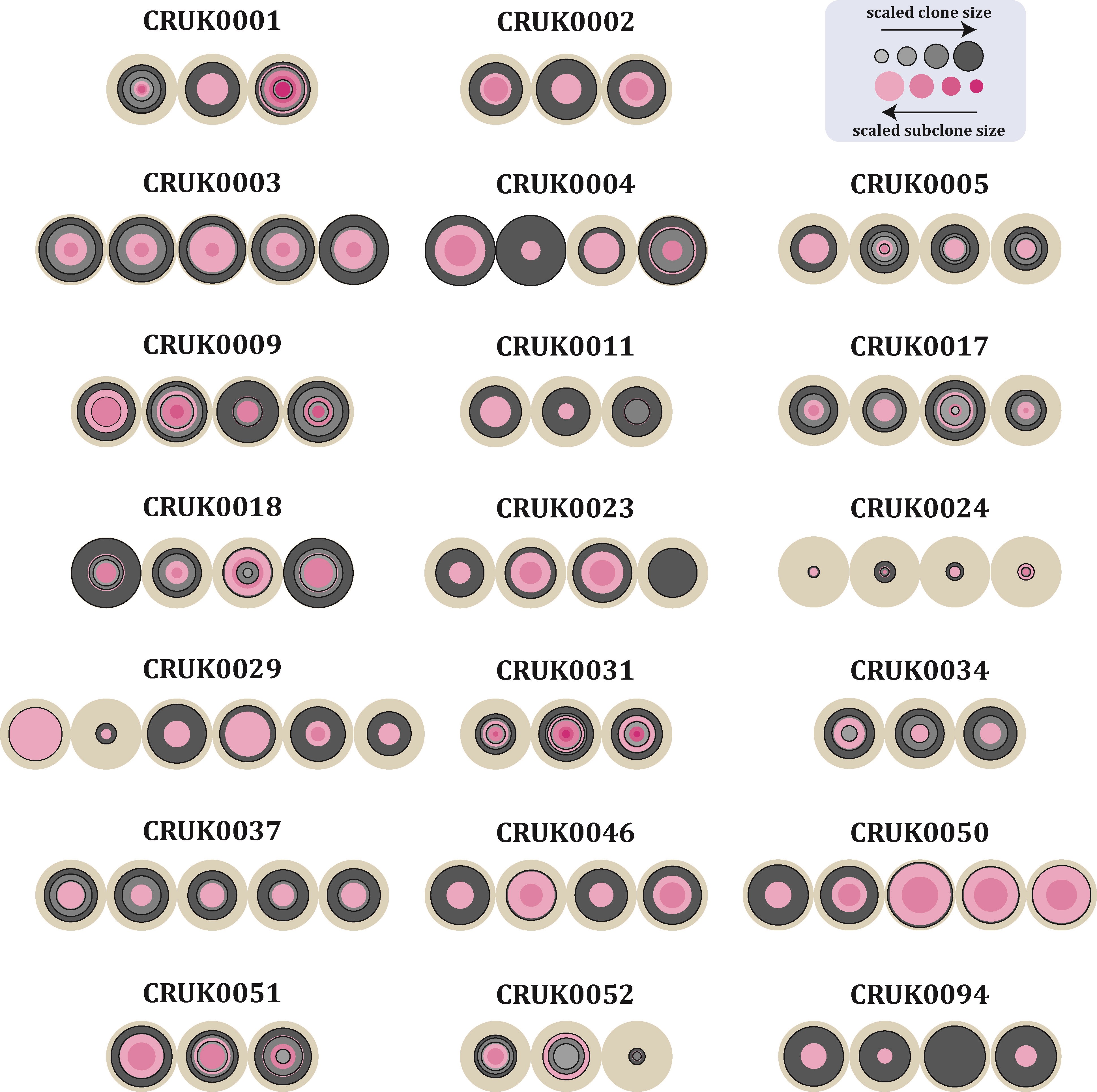

Supplement: Supplementary_Figure_6_bbab292 [file supplementary_figure_6_bbab292.jpeg]

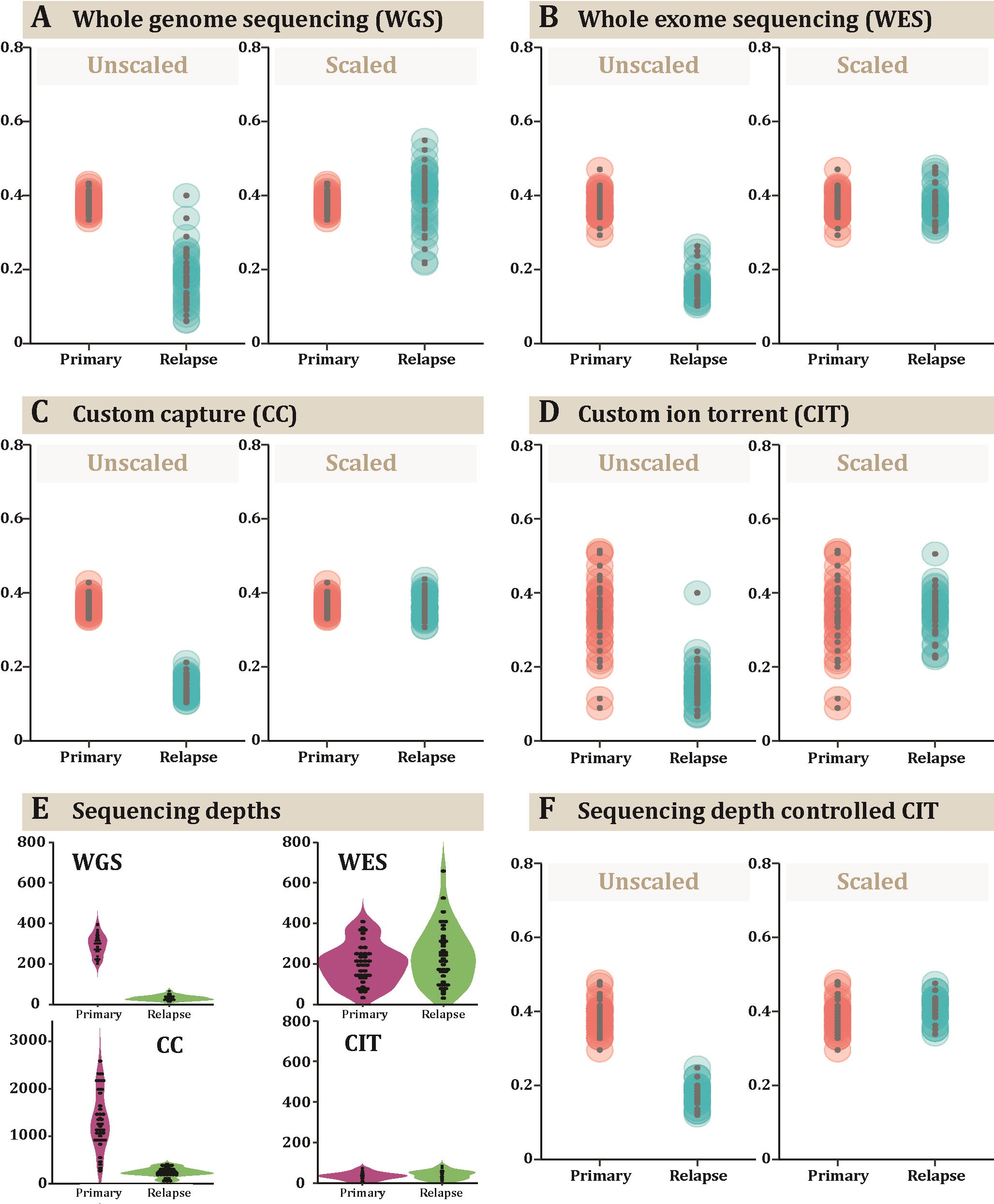

Supplement: Supplementary_Figure_7_bbab292 [file supplementary_figure_7_bbab292.jpeg]
