# Supplementary material for: Tracing the evolution of aneuploid cancers by multiregional sequencing with CRUST
Source: Brief Bioinform. 2021 Aug 3;22(6):bbab292. doi: 10.1093/bib/bbab292 (PMC8981300; doi:10.1093/bib/bbab292)

NB22_P1
Chromosome 1

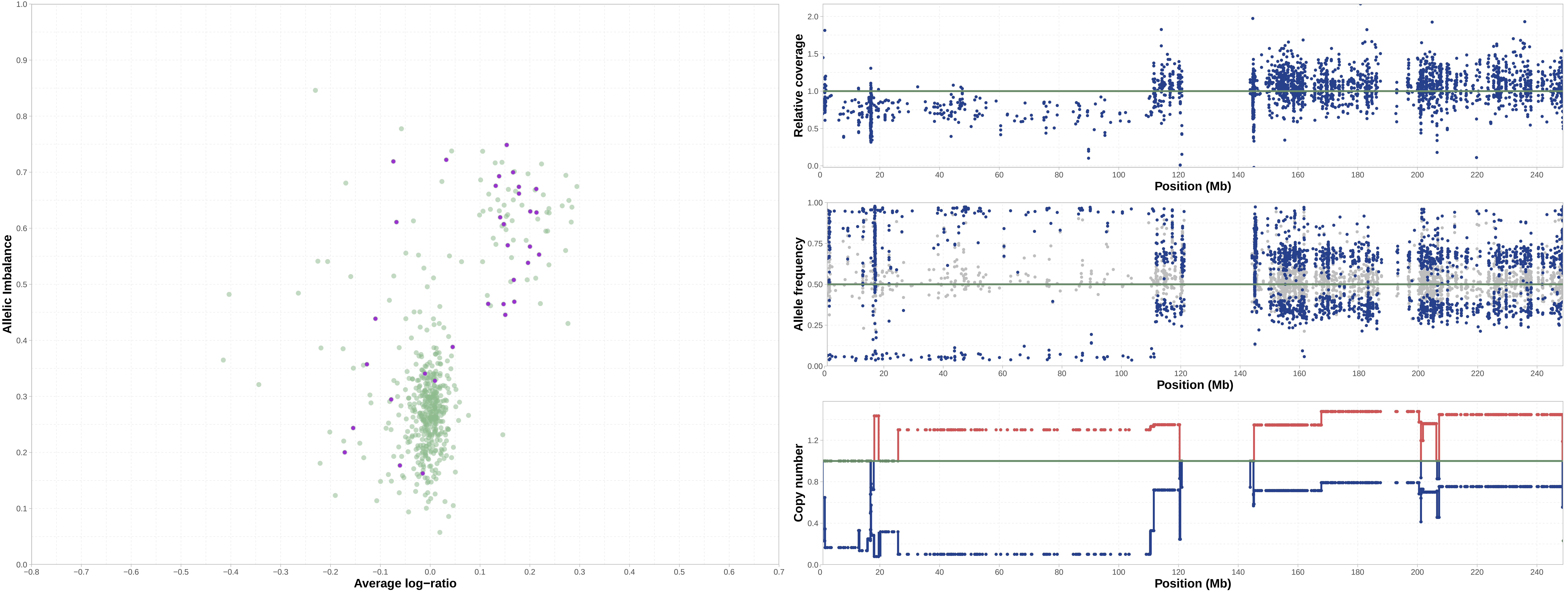

NB22_P1
Chromosome 2

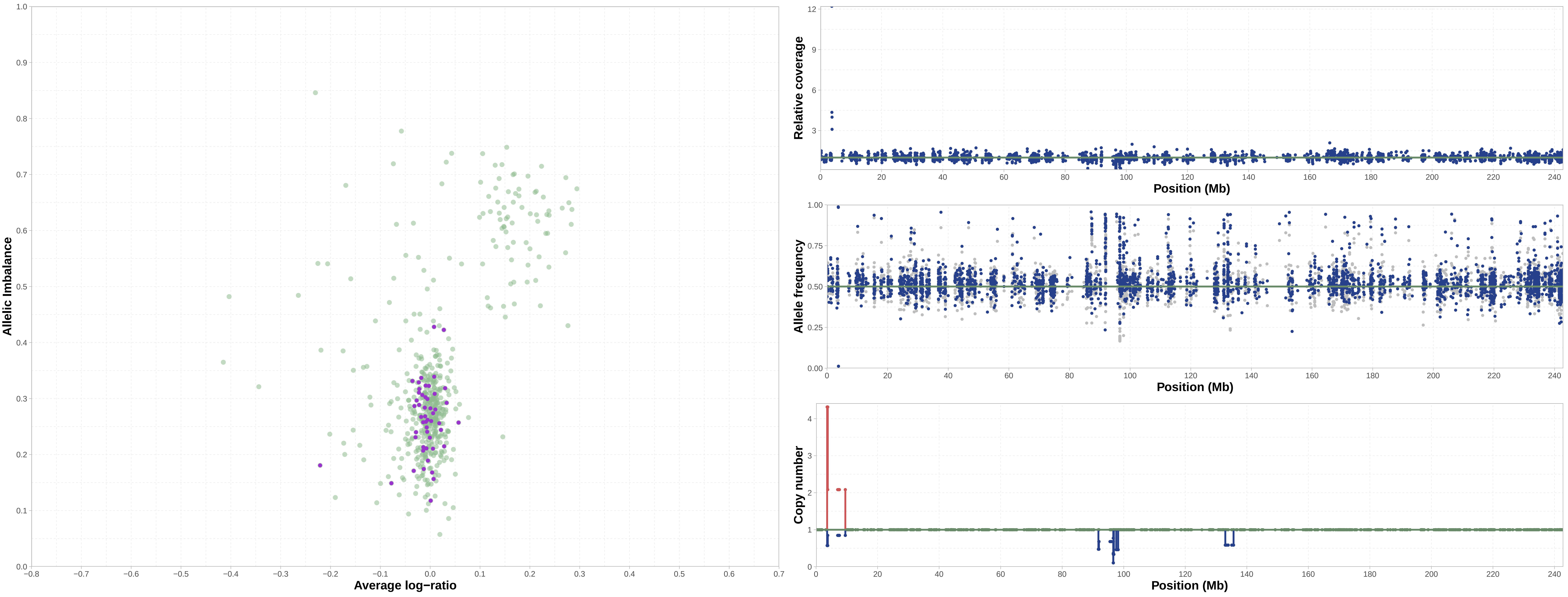

NB22_P1
Chromosome 3

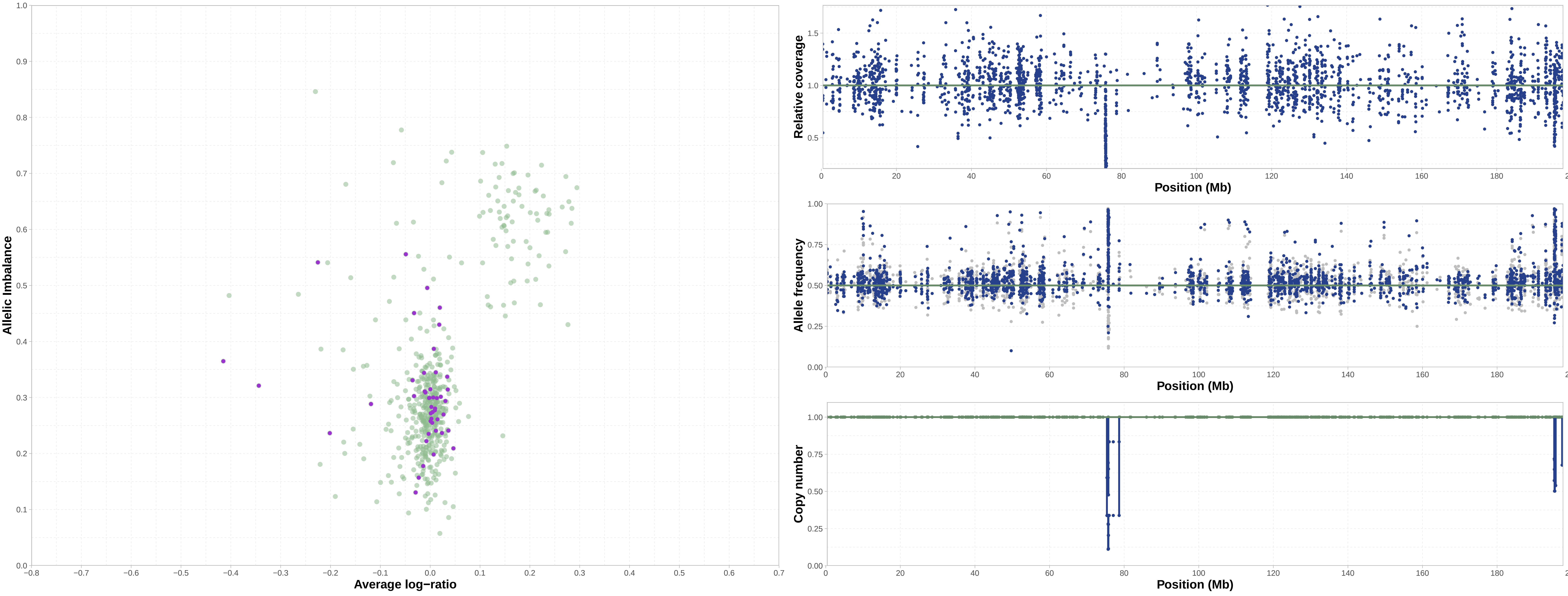

NB22_P1
Chromosome 4

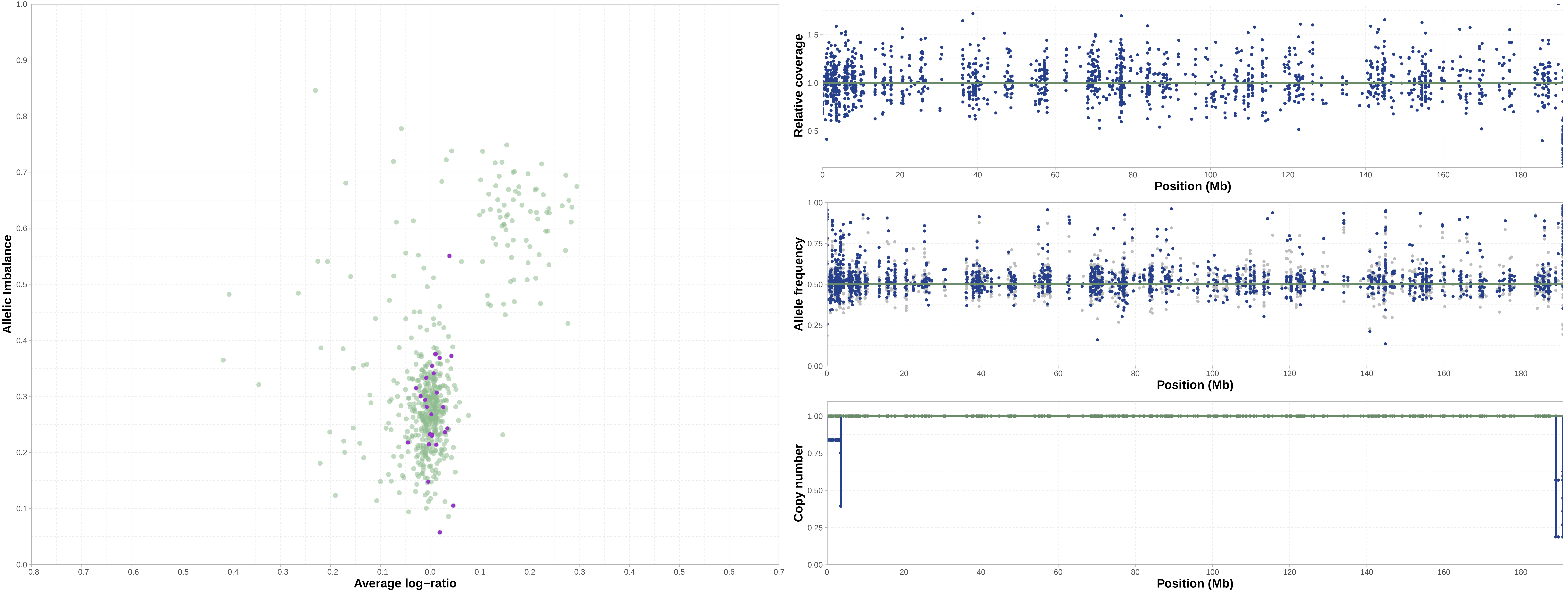

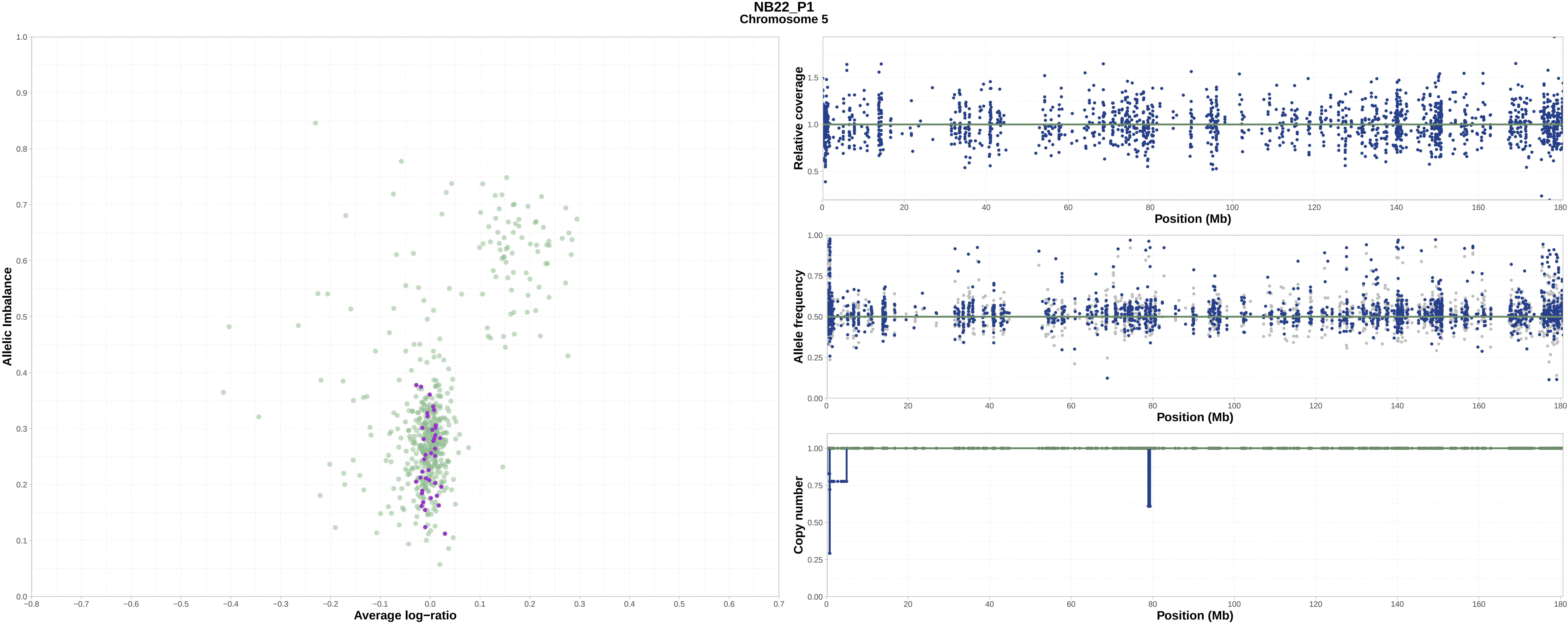

NB22_P1
Chromosome 5
Allelic Imbalance
Average log−ratio
Relative coverage
Allele frequency
Copy number
Position (Mb)

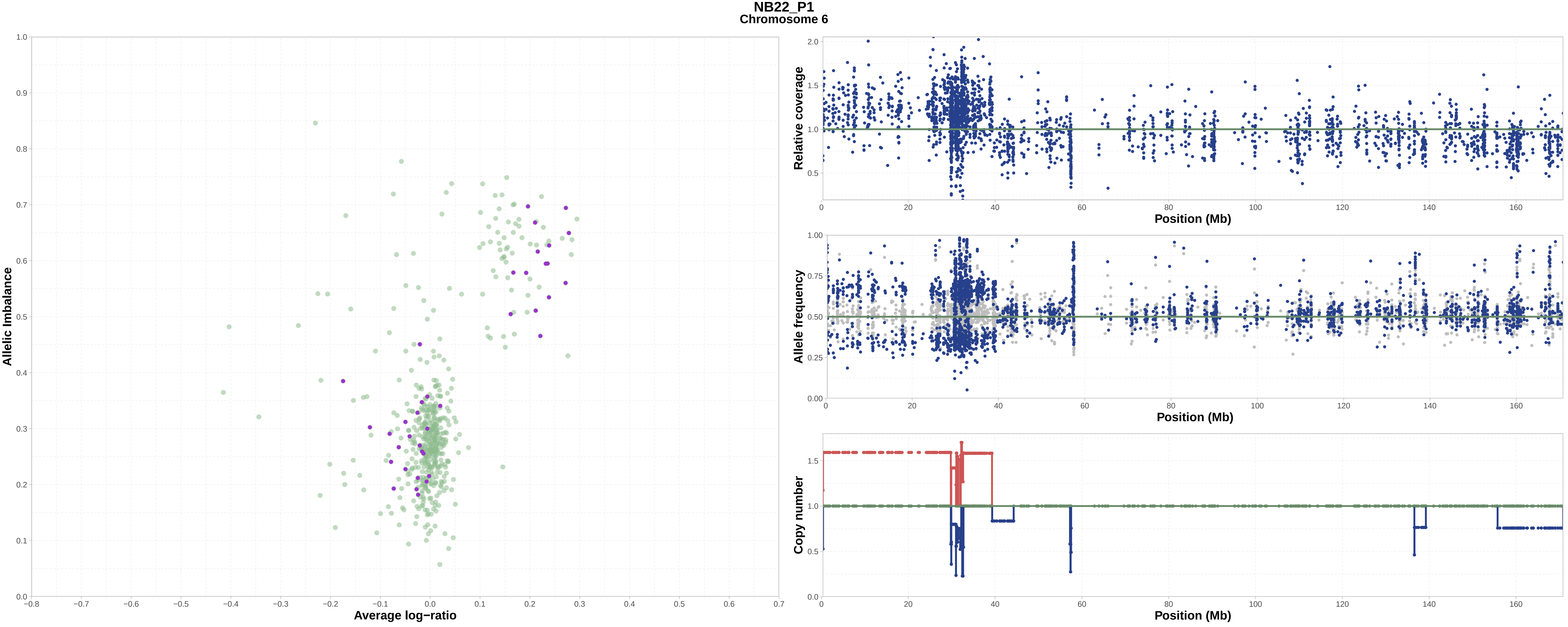
NB22_P1
Chromosome 6
Allelic Imbalance
Average log−ratio
Relative coverage
Position (Mb)
Allele frequency
Position (Mb)
Copy number
Position (Mb)

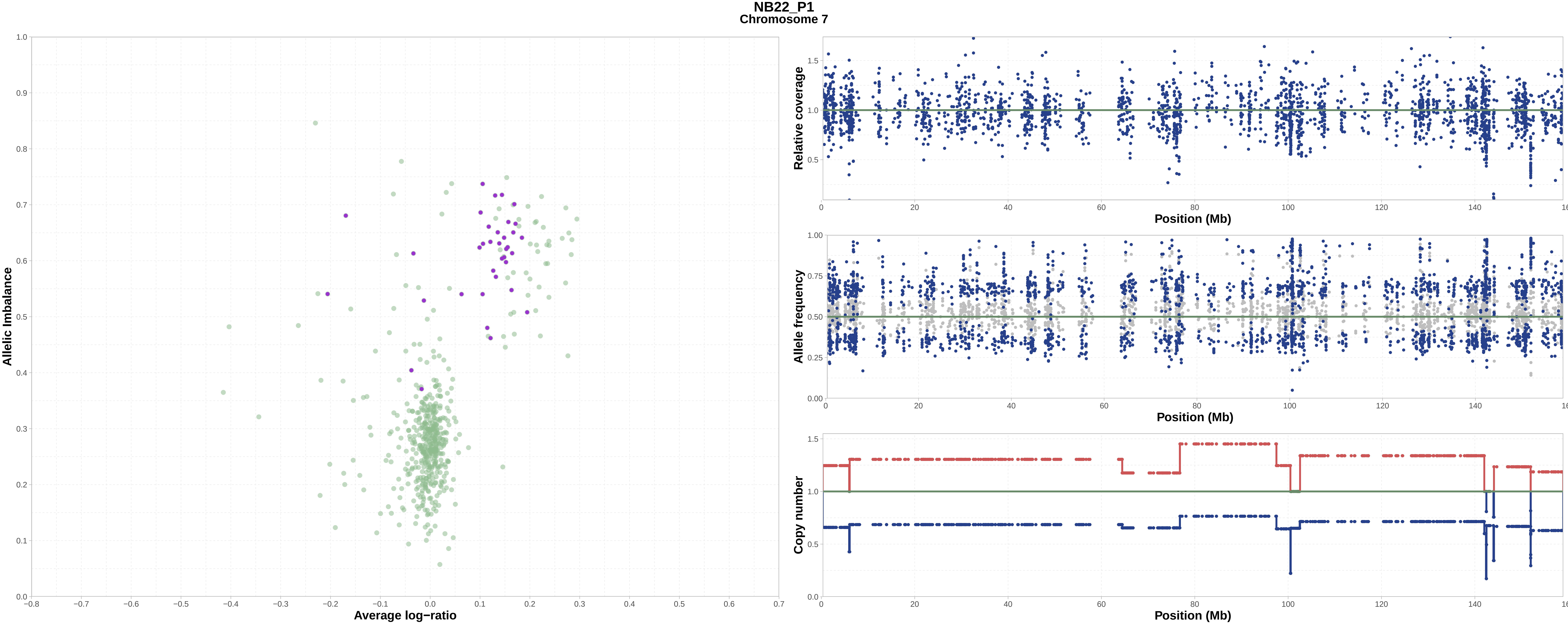
NB22_P1
Chromosome 7
Allelic Imbalance
Average log−ratio
Relative coverage
Allele frequency
Copy number
Position (Mb)
Position (Mb)
Position (Mb)

NB22_P1
Chromosome 8

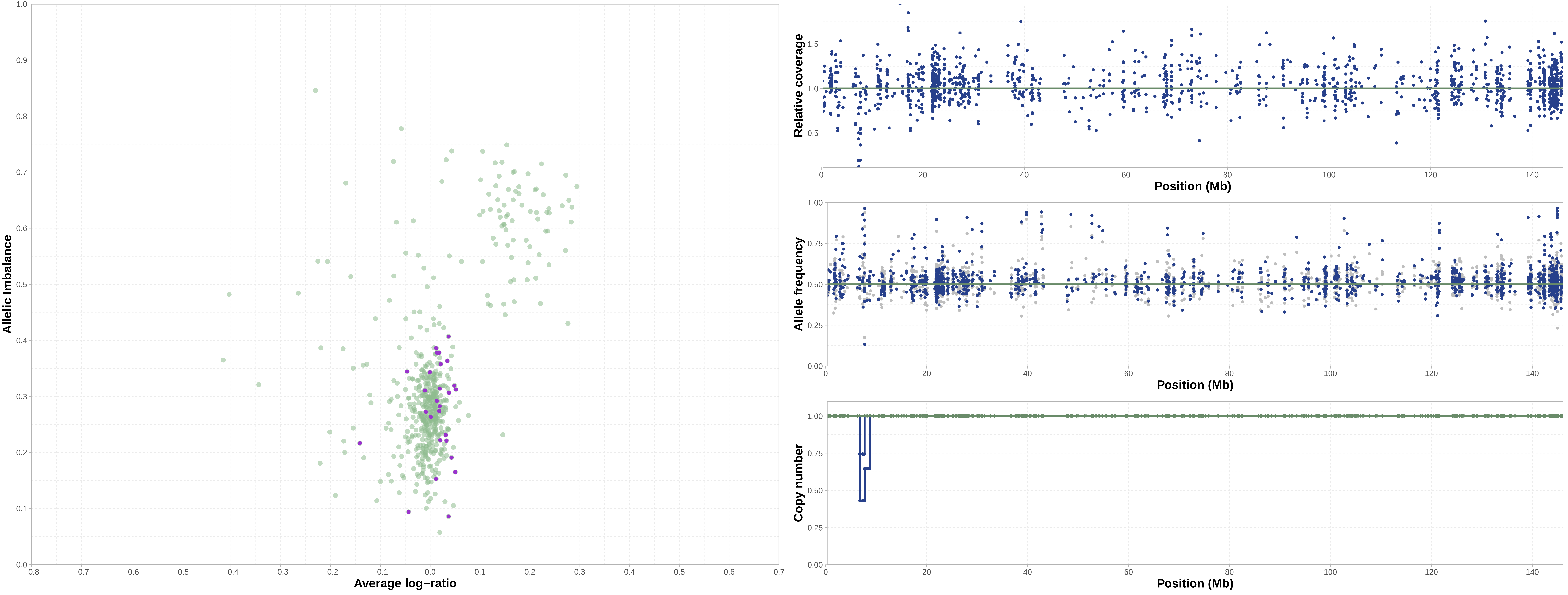

NB22_P1
Chromosome 9

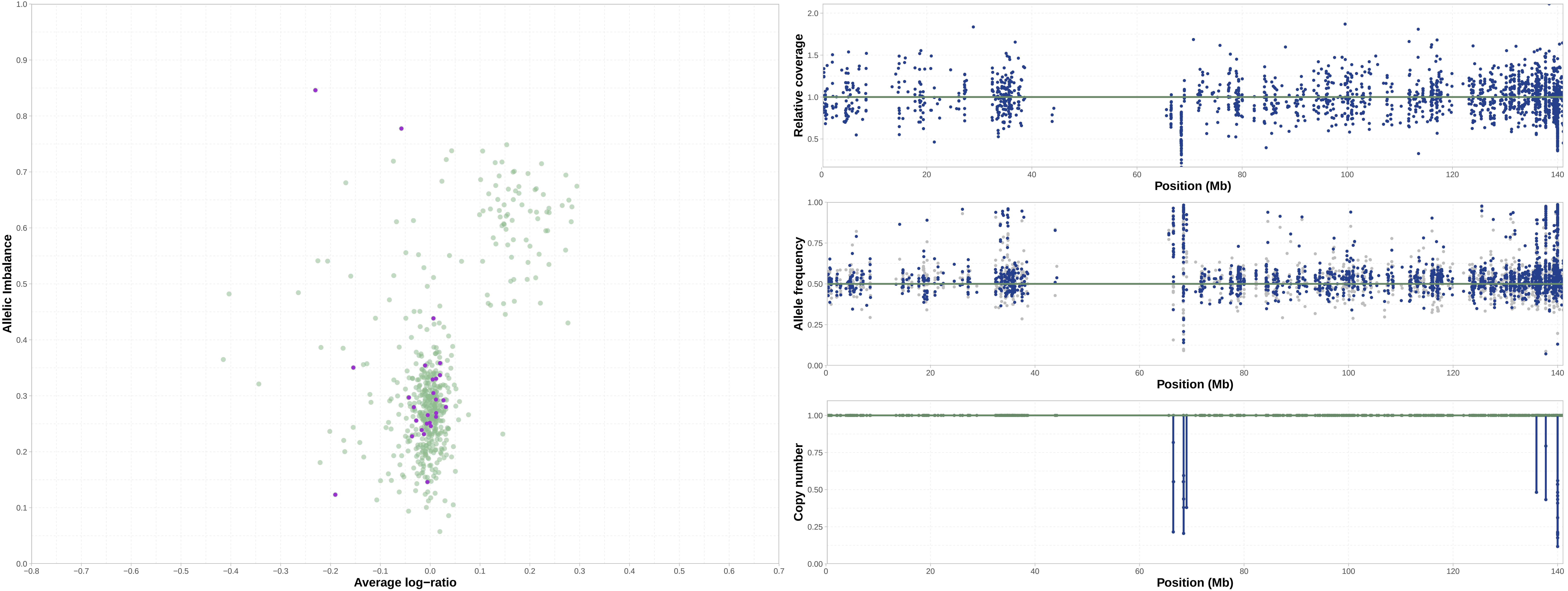

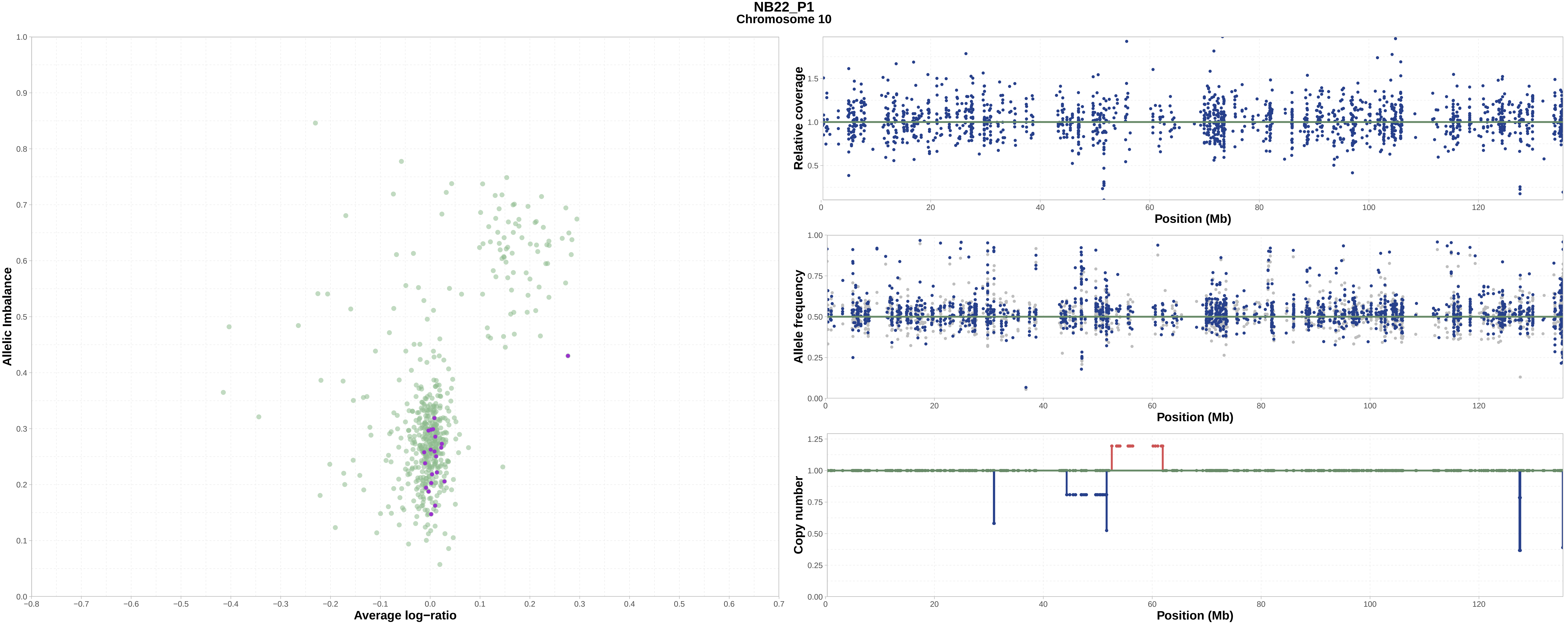
NB22_P1
Chromosome 10
Allelic Imbalance
Average log−ratio
Relative coverage
Position (Mb)
Allele frequency
Position (Mb)
Copy number
Position (Mb)

NB22_P1
Chromosome 11

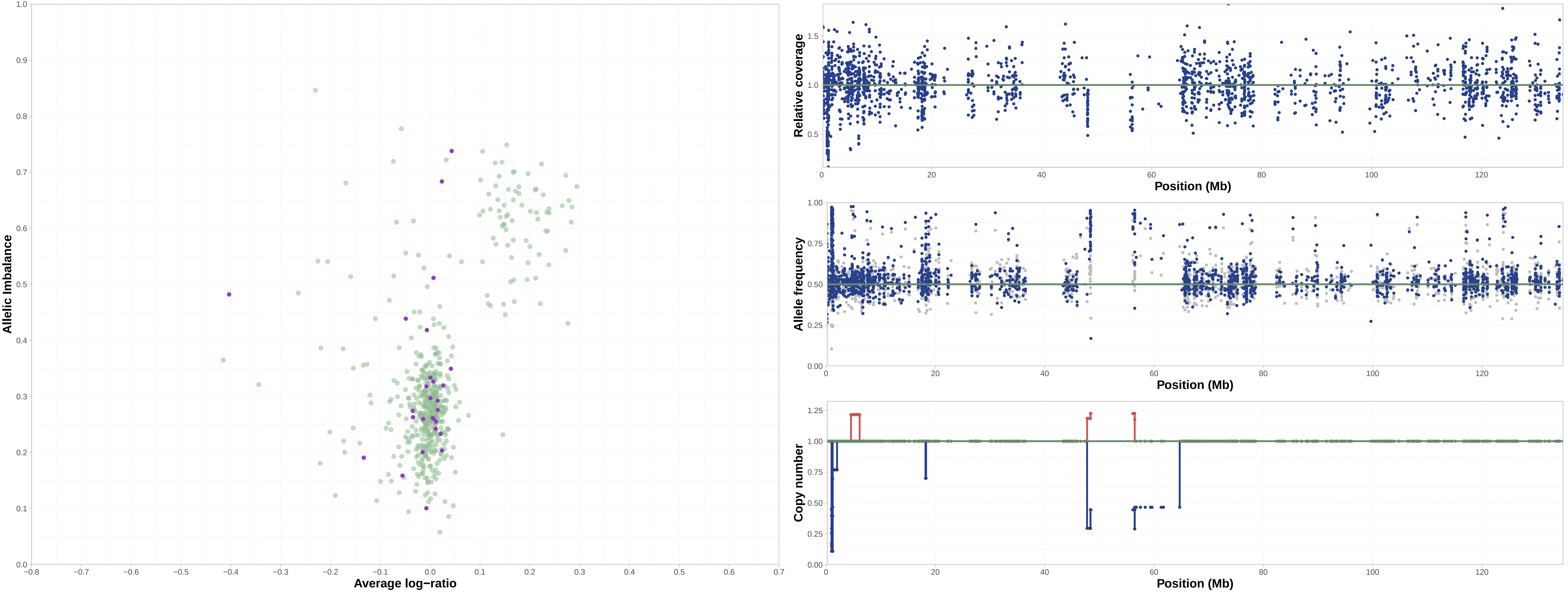

NB22_P1
Chromosome 12

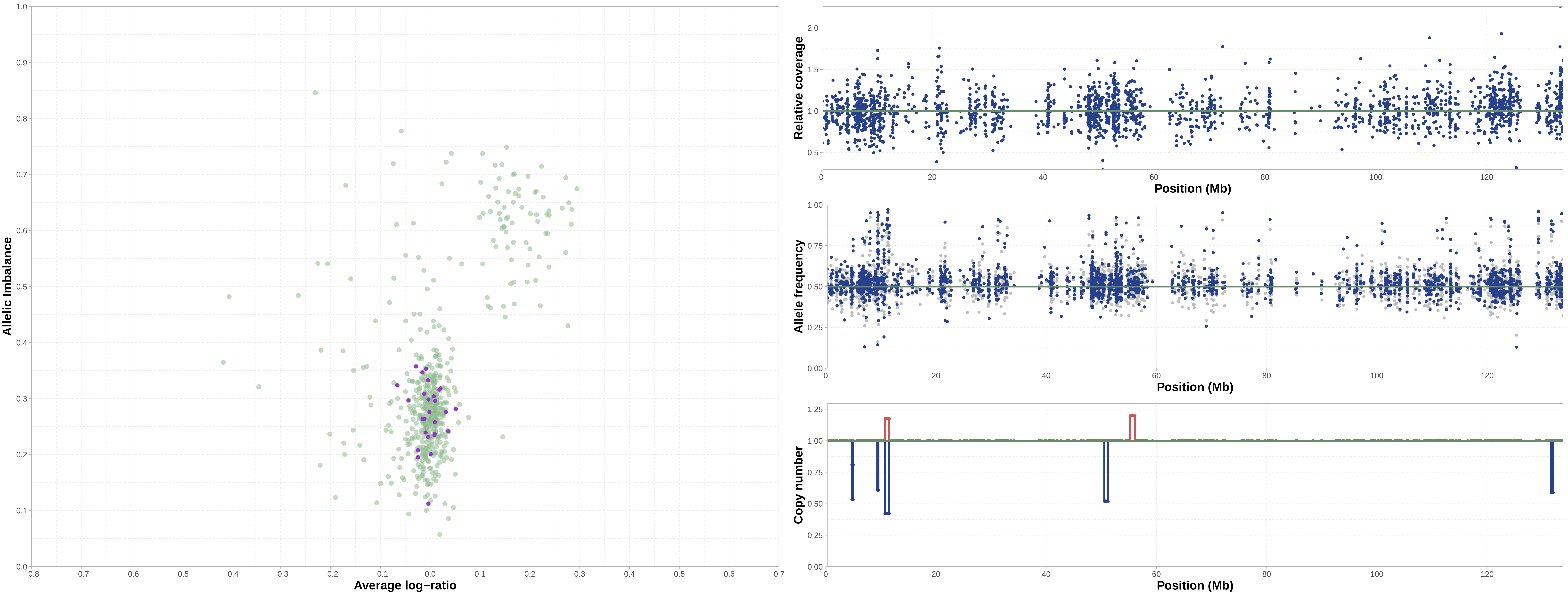

NB22_P1
Chromosome 13

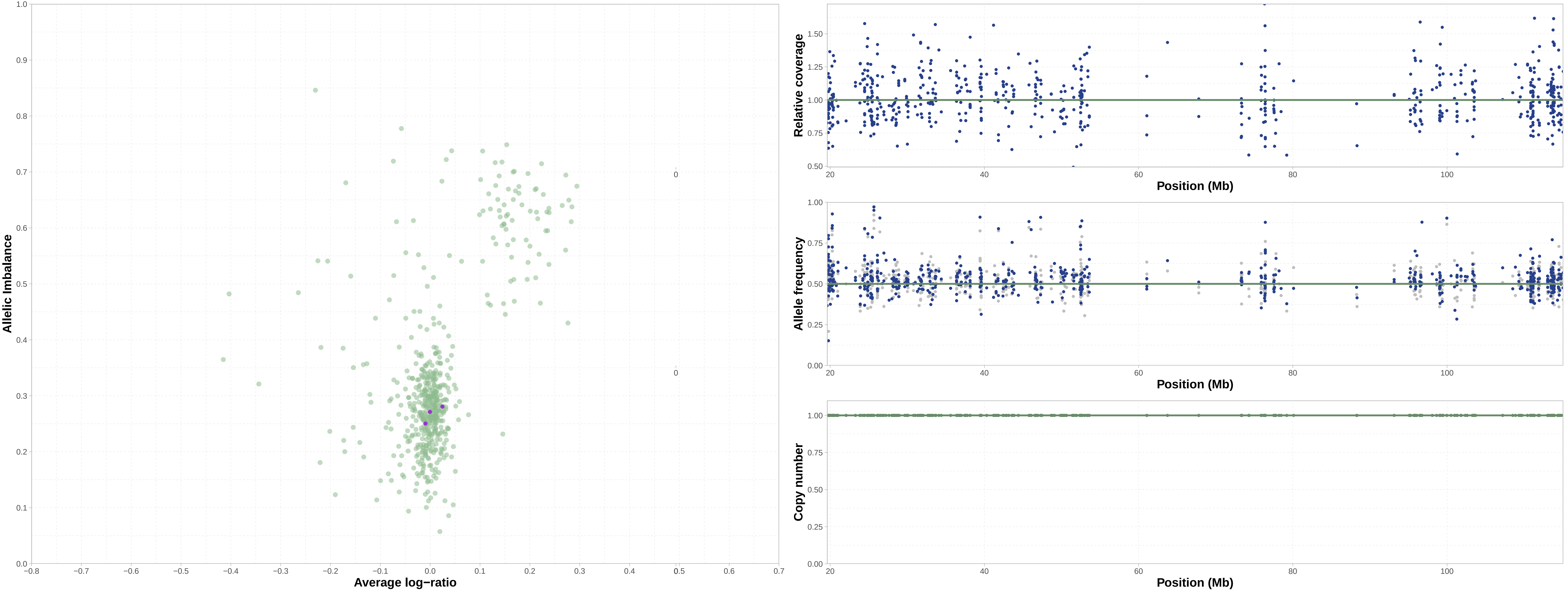

NB22_P1
Chromosome 14

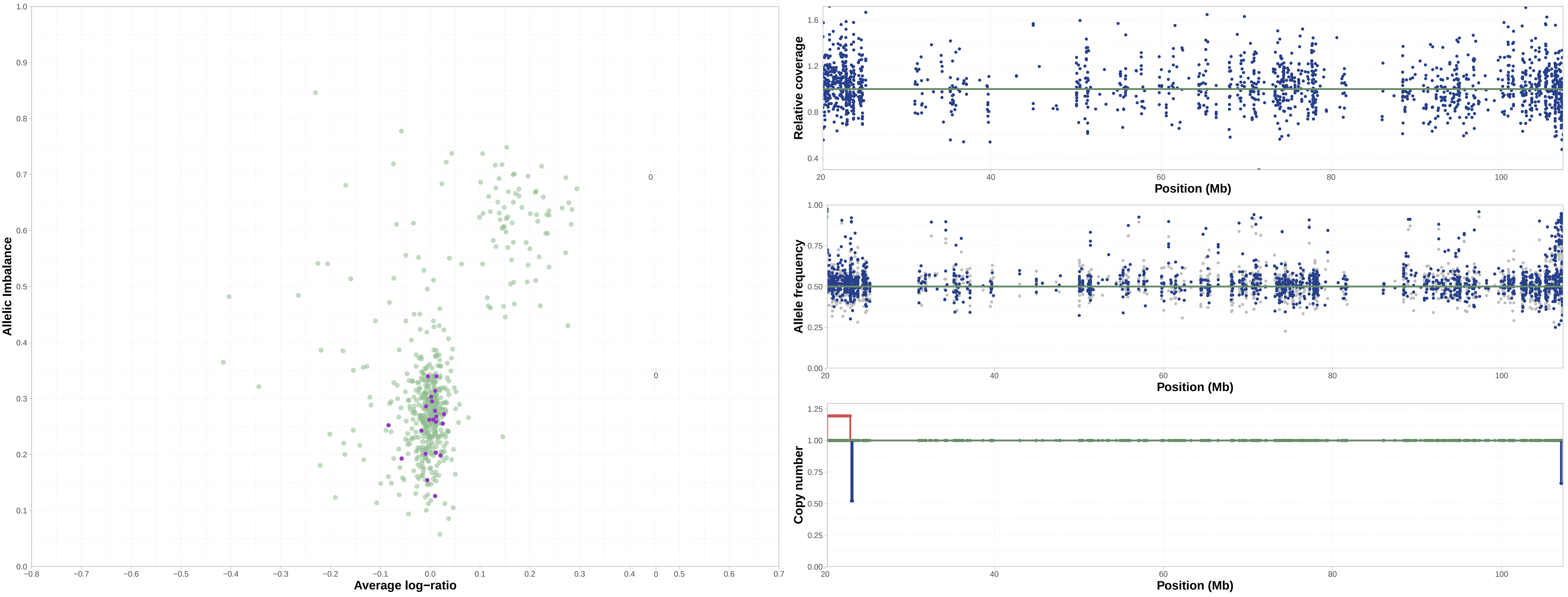

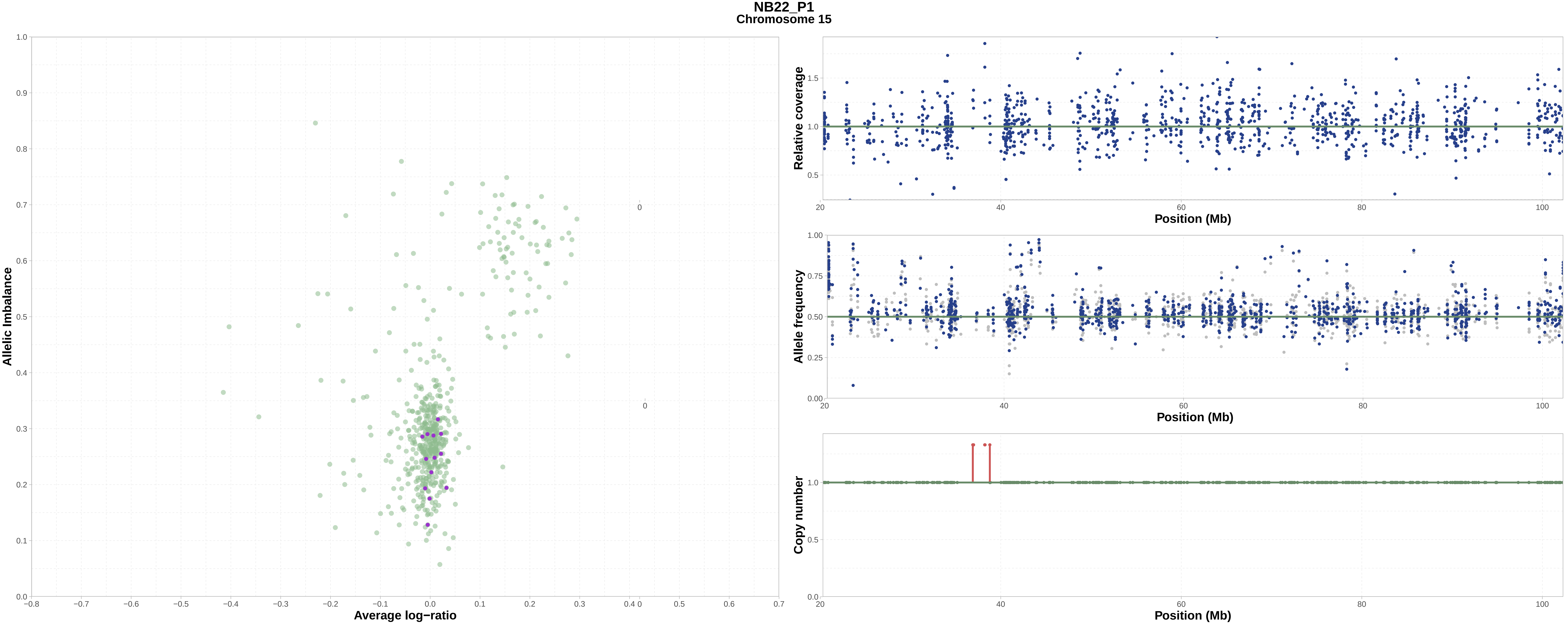
NB22_P1
Chromosome 15
Allelic Imbalance
Average log−ratio
Relative coverage
Position (Mb)
Allele frequency
Position (Mb)
Copy number
Position (Mb)

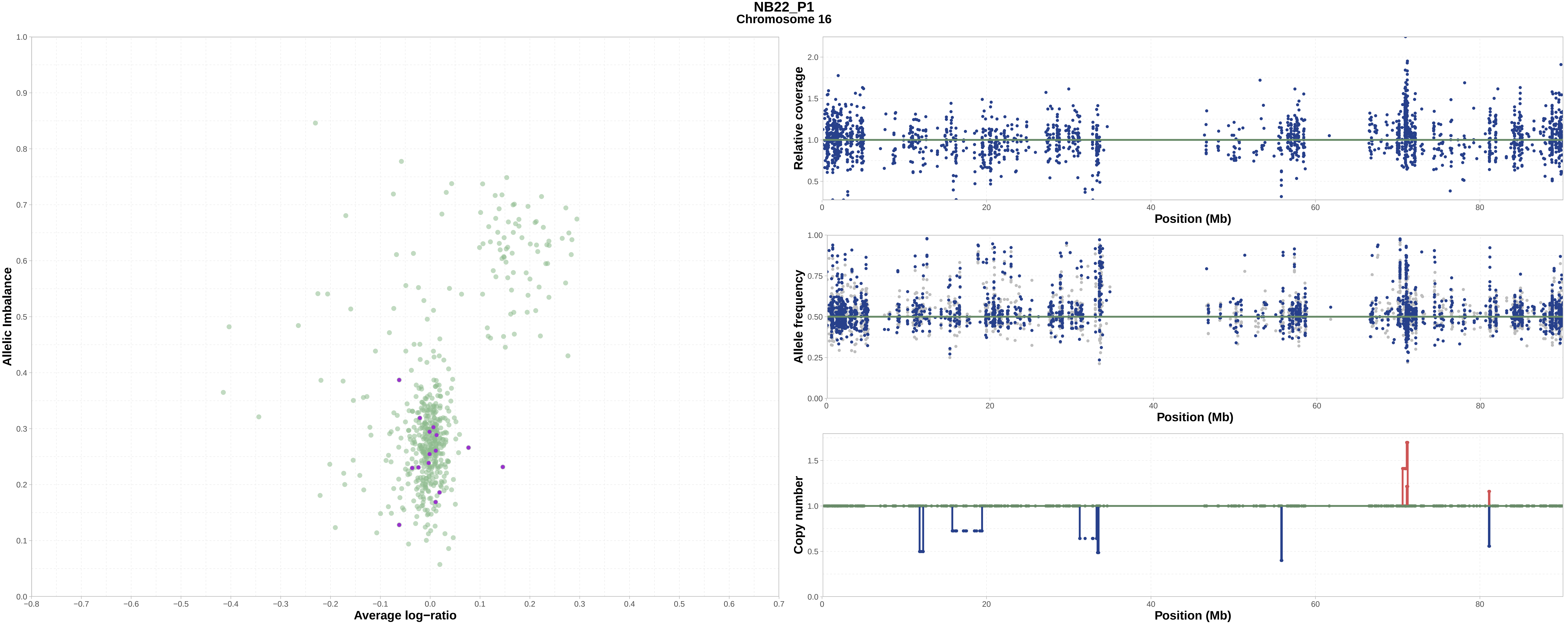

NB22_P1
Chromosome 16
Allelic Imbalance
Average log−ratio
Relative coverage
Position (Mb)
Allele frequency
Position (Mb)
Copy number
Position (Mb)

NB22_P1
Chromosome 17

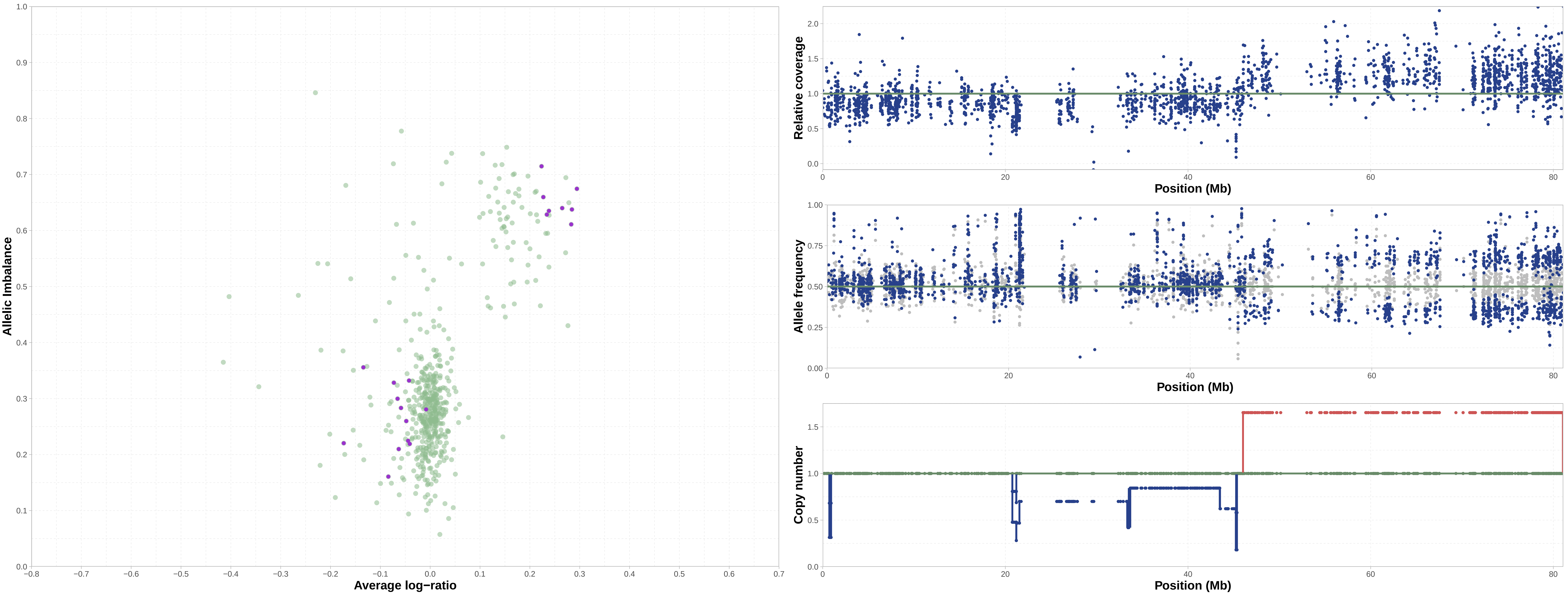

NB22_P1
Chromosome 18

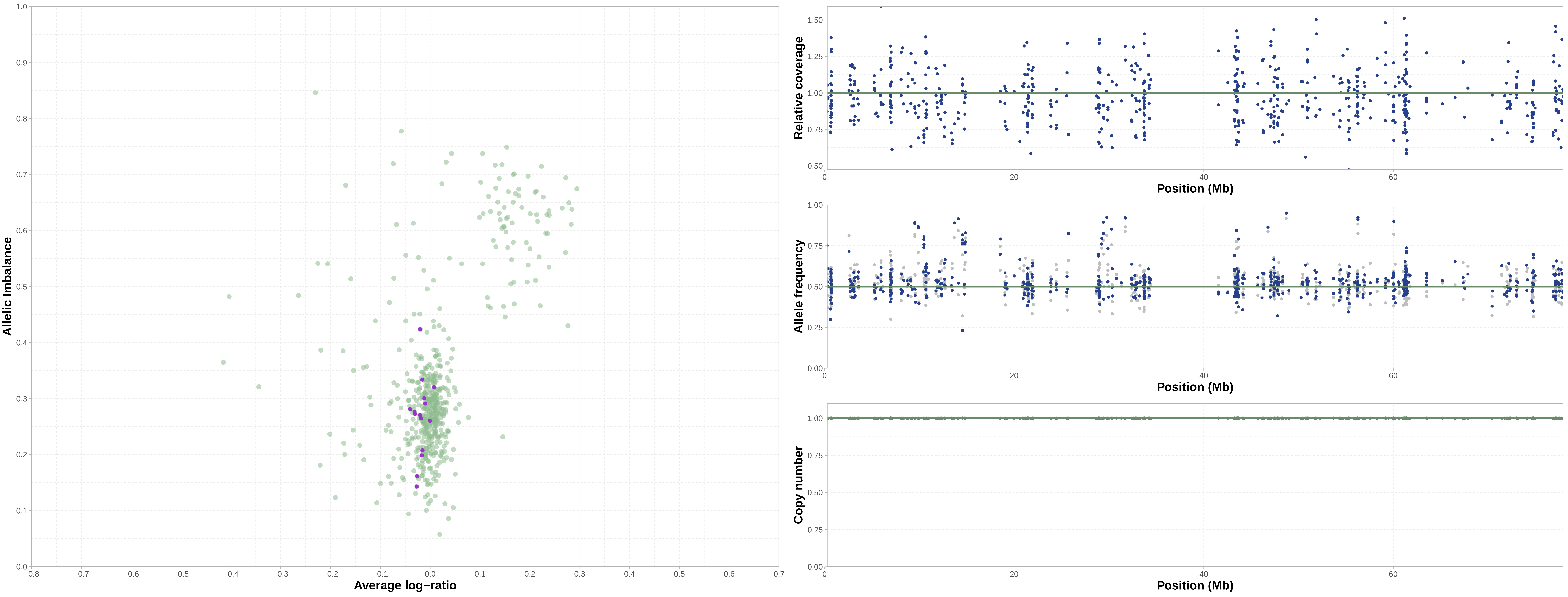

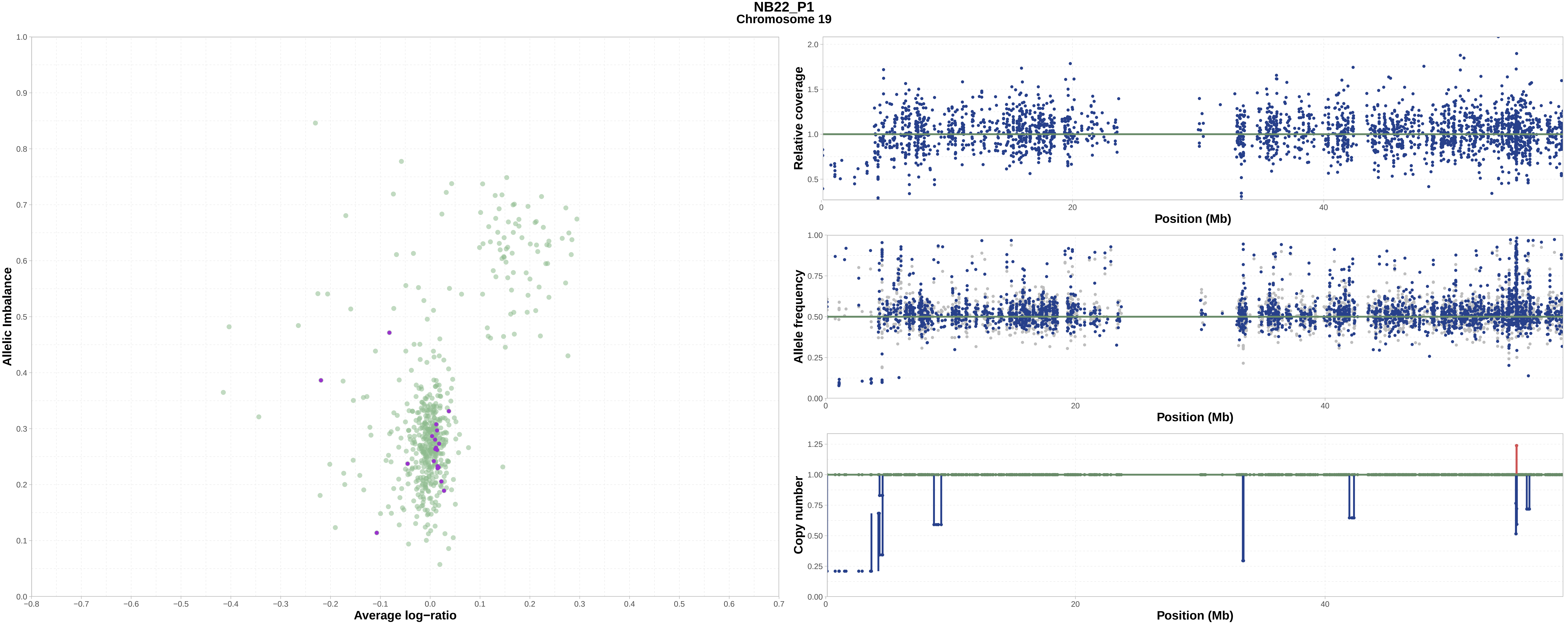
NB22_P1
Chromosome 19
Allelic Imbalance
Average log−ratio
Relative coverage
Position (Mb)
Allele frequency
Position (Mb)
Copy number
Position (Mb)

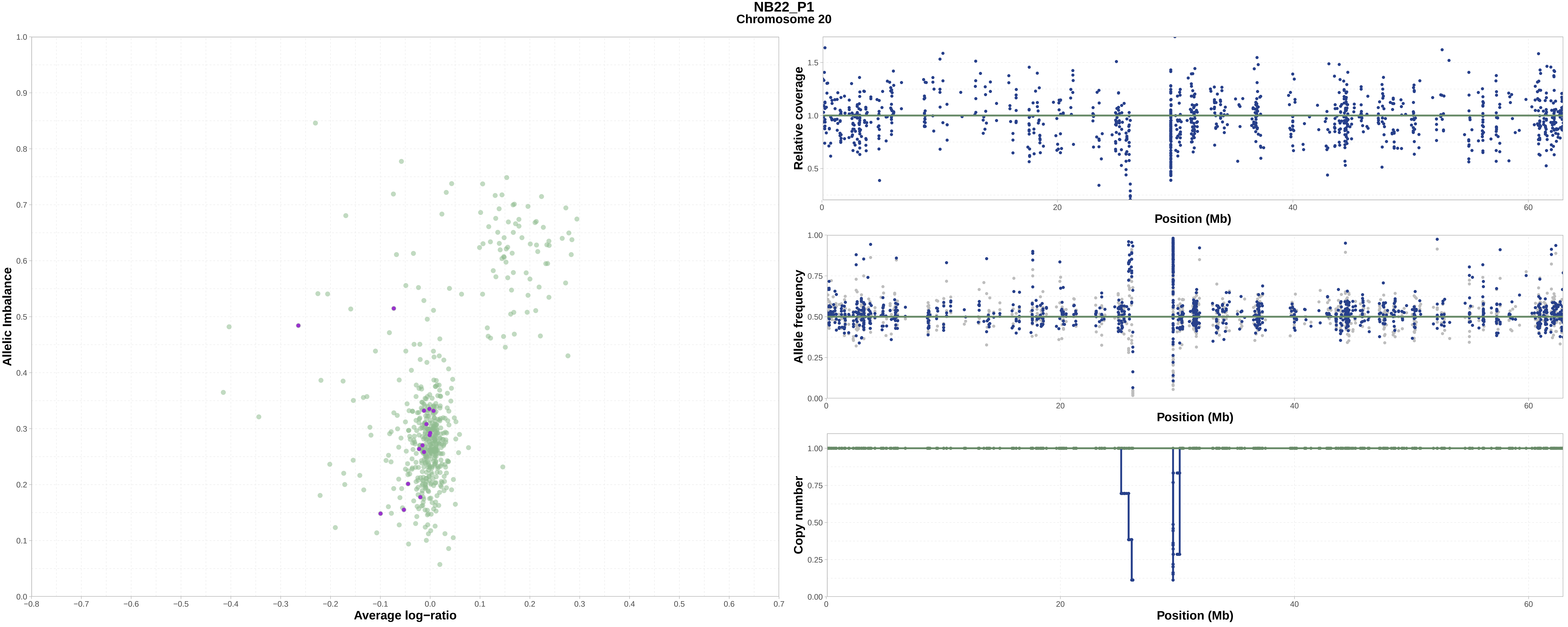

NB22_P1
Chromosome 20
Allelic Imbalance
Average log−ratio
Relative coverage
Allele frequency
Copy number
Position (Mb)

NB22_P1
Chromosome 21

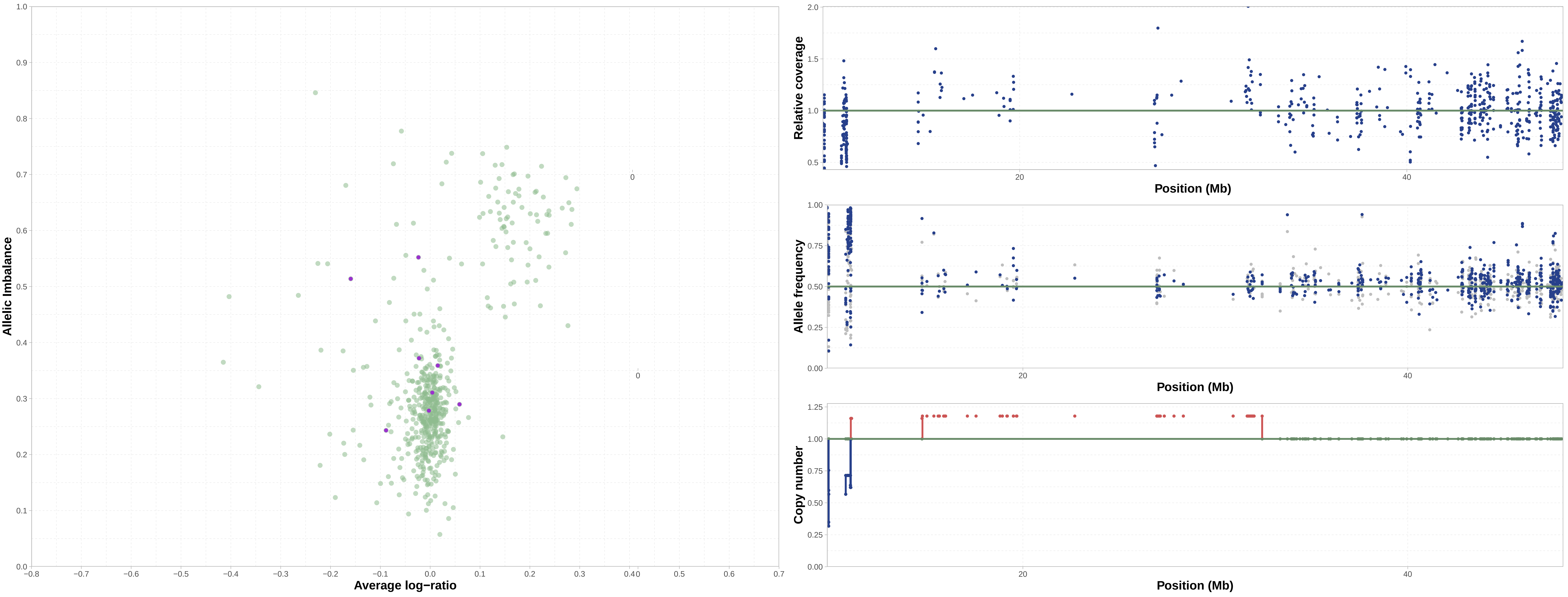

NB22_P1
Chromosome 22

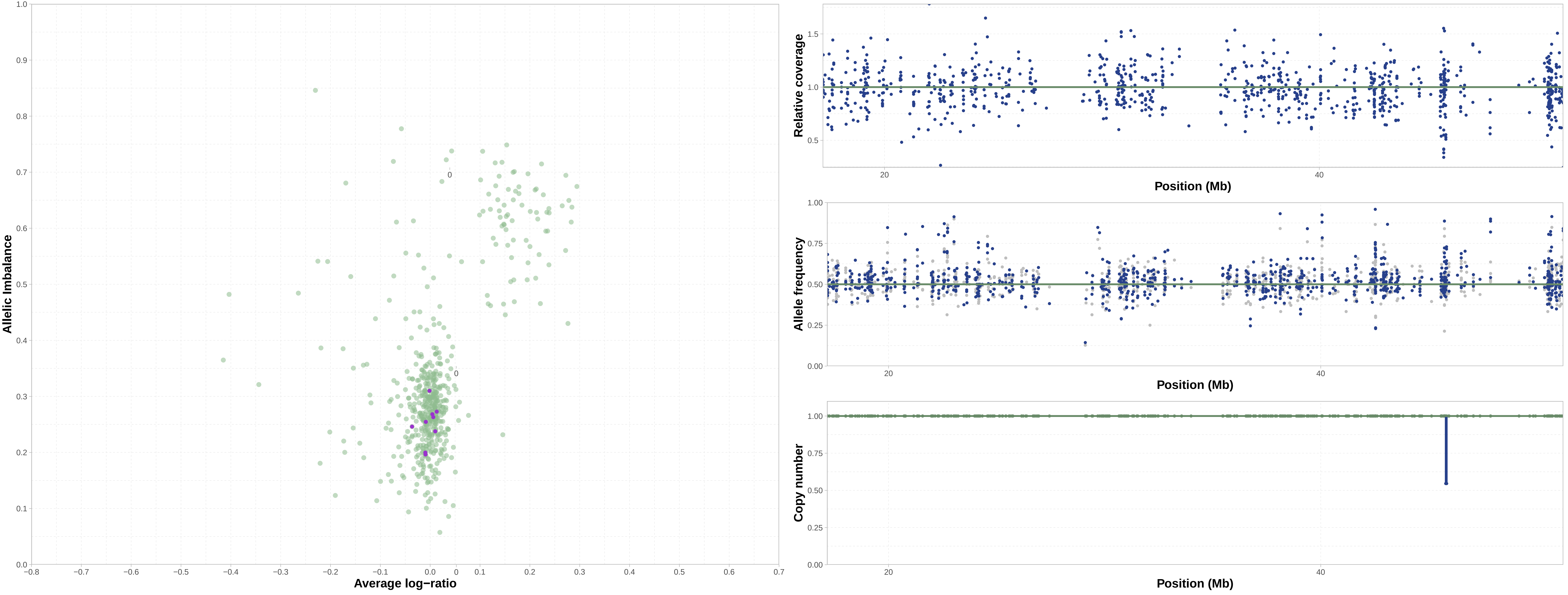

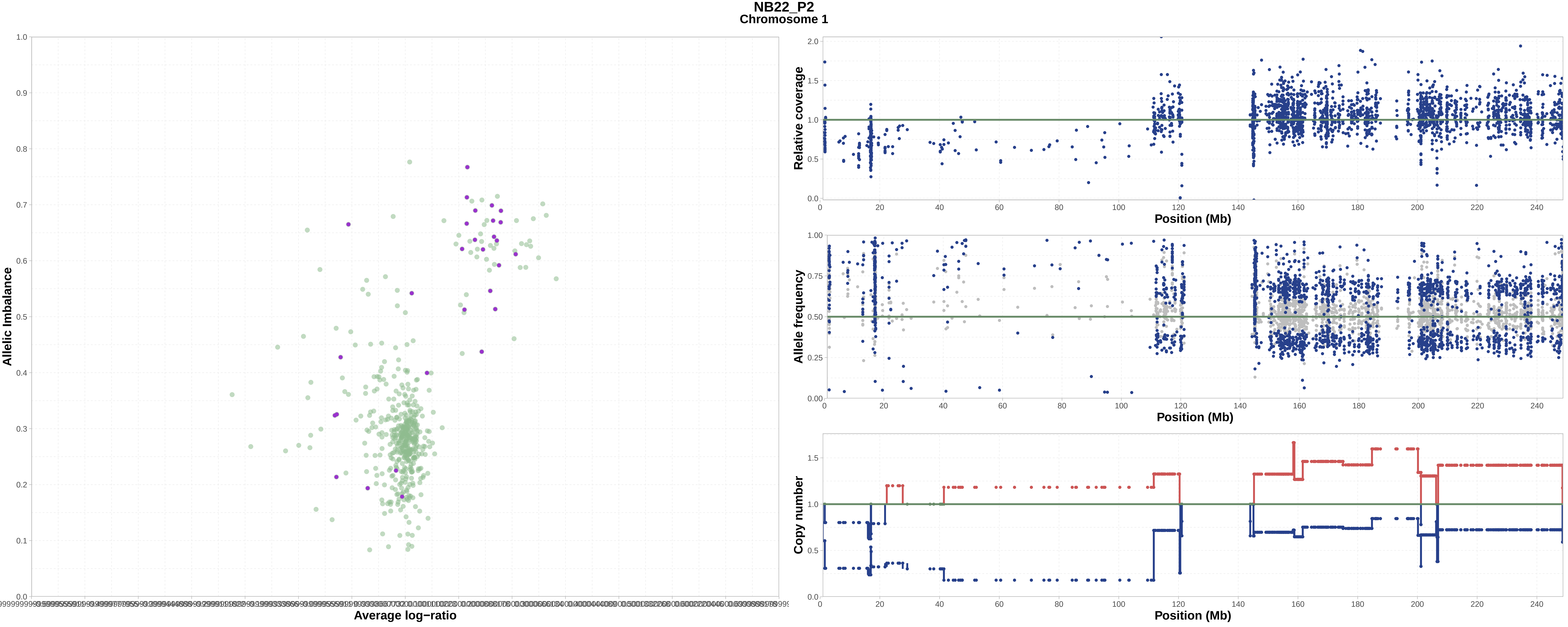
NB22_P2
Chromosome 1
Allelic Imbalance
Average log−ratio
Relative coverage
Position (Mb)
Allele frequency
Position (Mb)
Copy number
Position (Mb)

NB22_P2
Chromosome 2

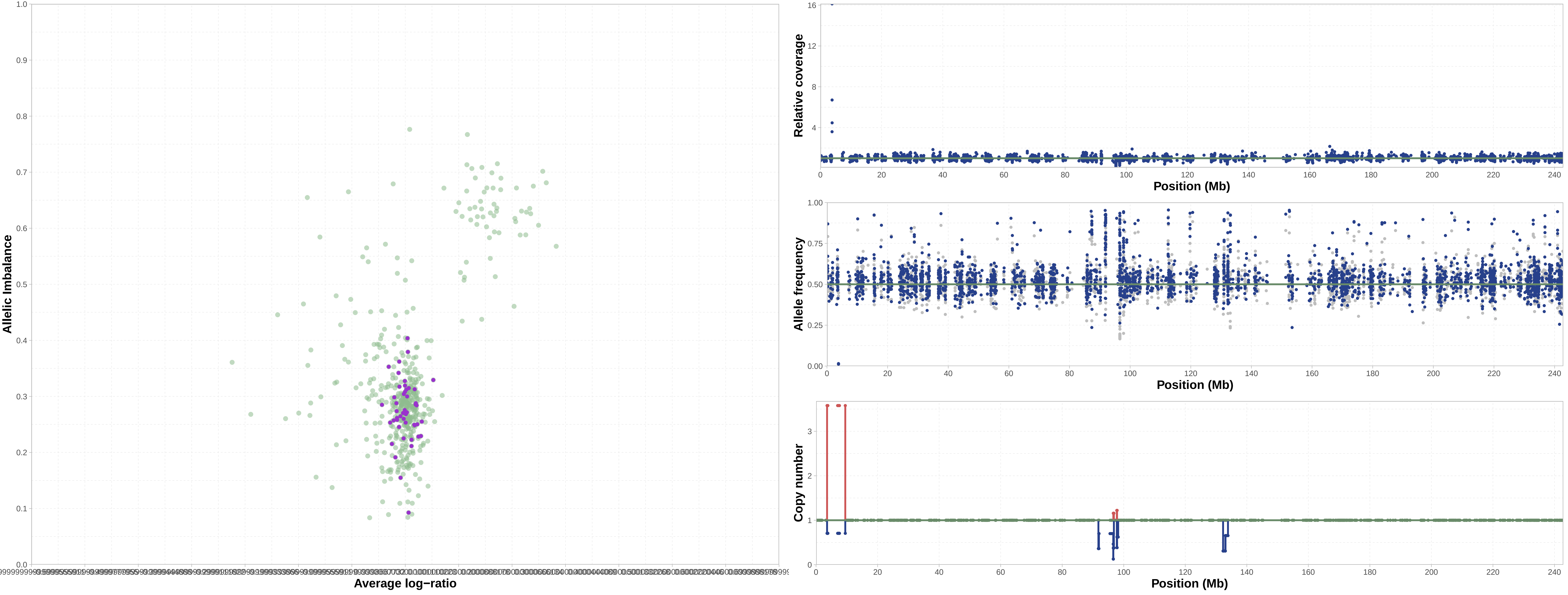

NB22_P2
Chromosome 3

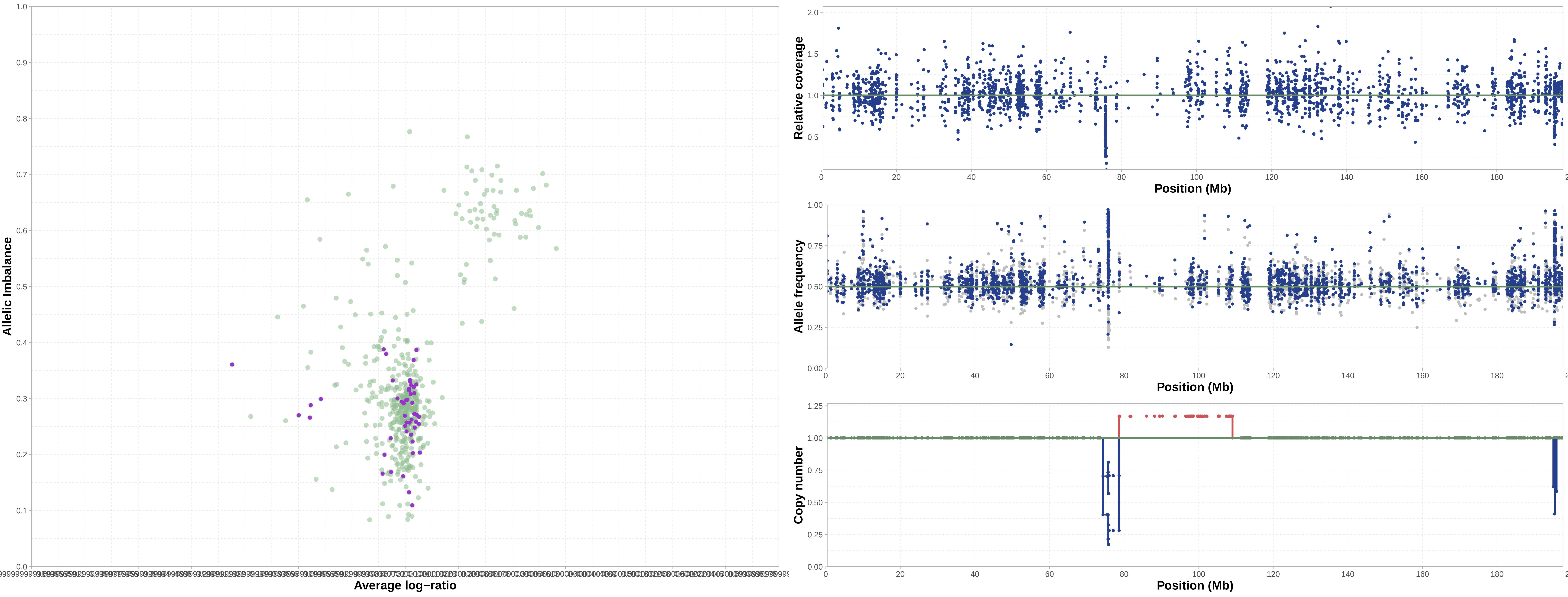

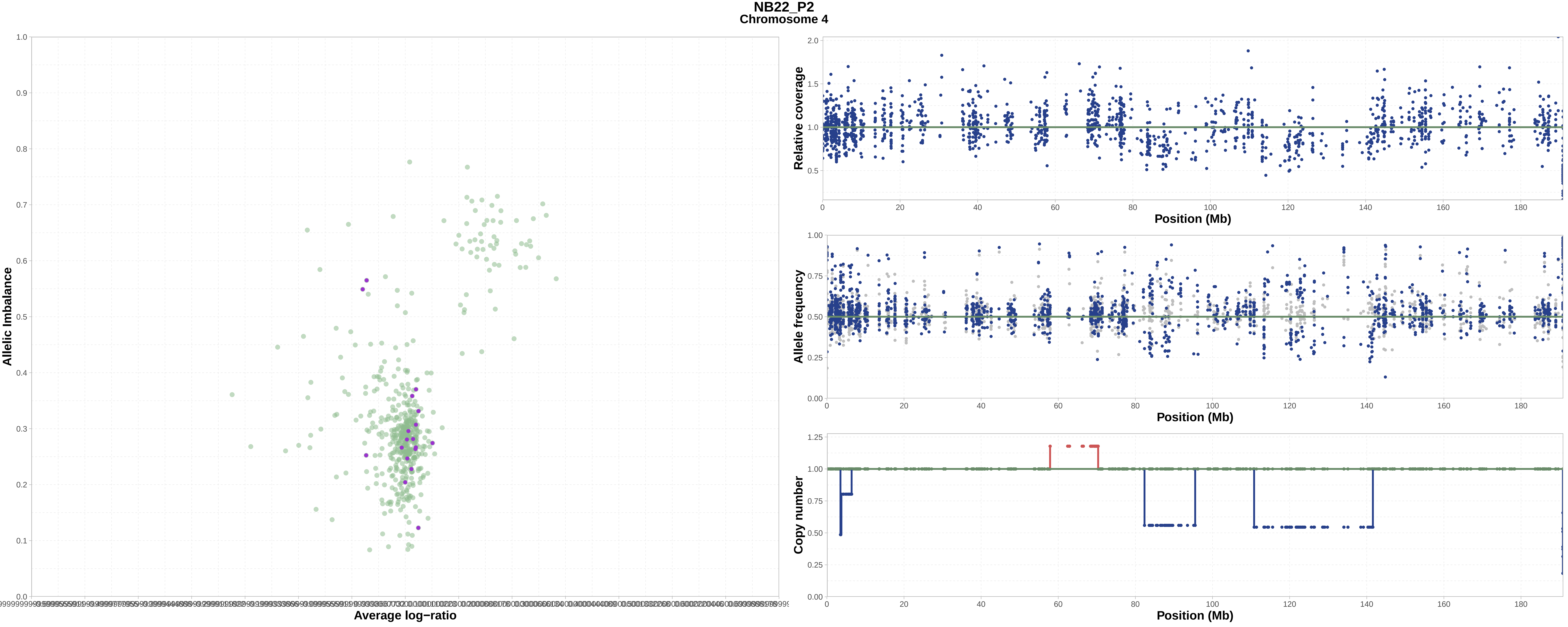
NB22_P2
Chromosome 4
Allelic Imbalance
Average log−ratio
Relative coverage
Position (Mb)
Allele frequency
Position (Mb)
Copy number
Position (Mb)

NB22_P2
Chromosome 5

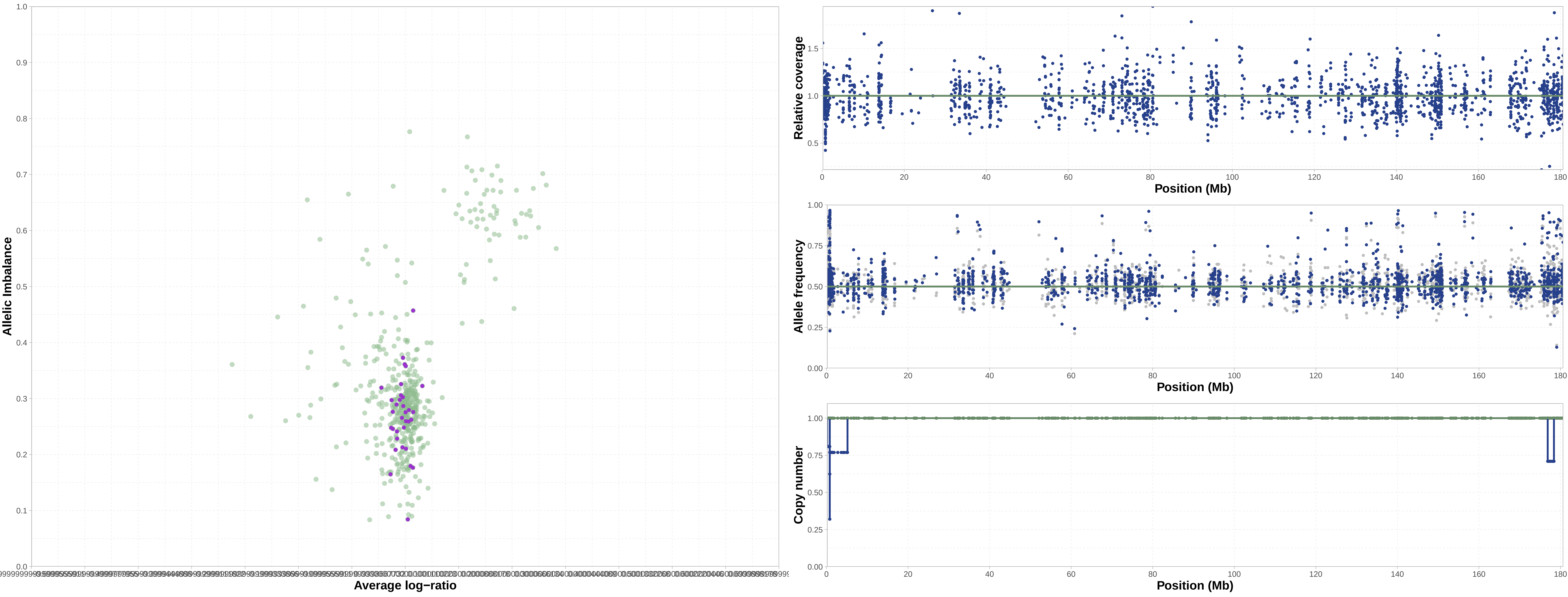

NB22_P2
Chromosome 6

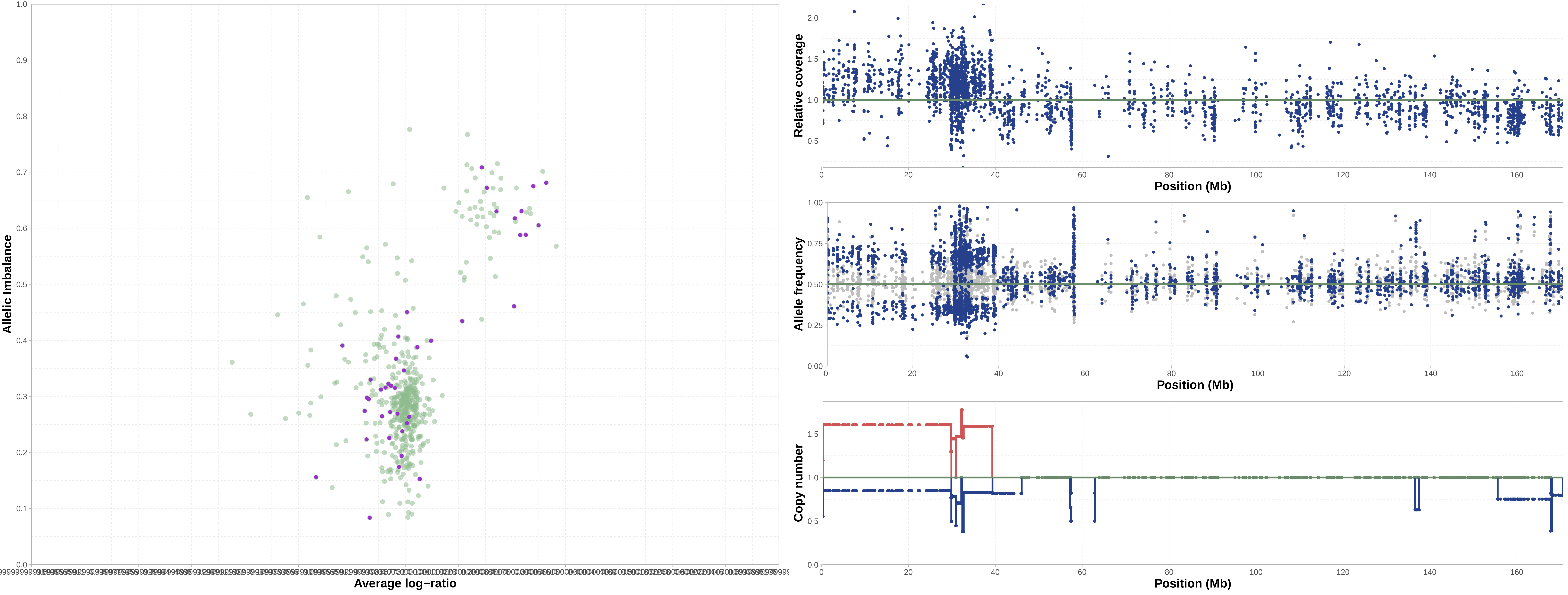

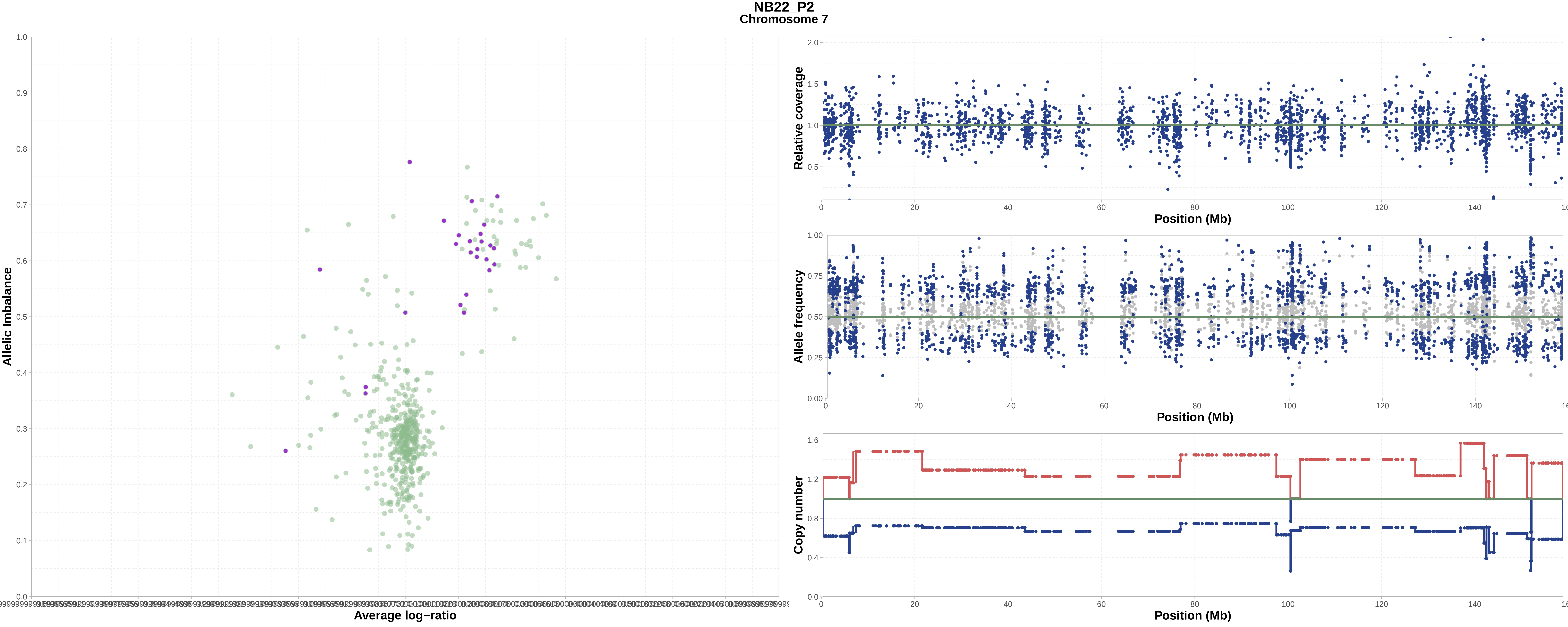
NB22_P2
Chromosome 7
Allelic Imbalance
Average log−ratio
Relative coverage
Position (Mb)
Allele frequency
Position (Mb)
Copy number
Position (Mb)

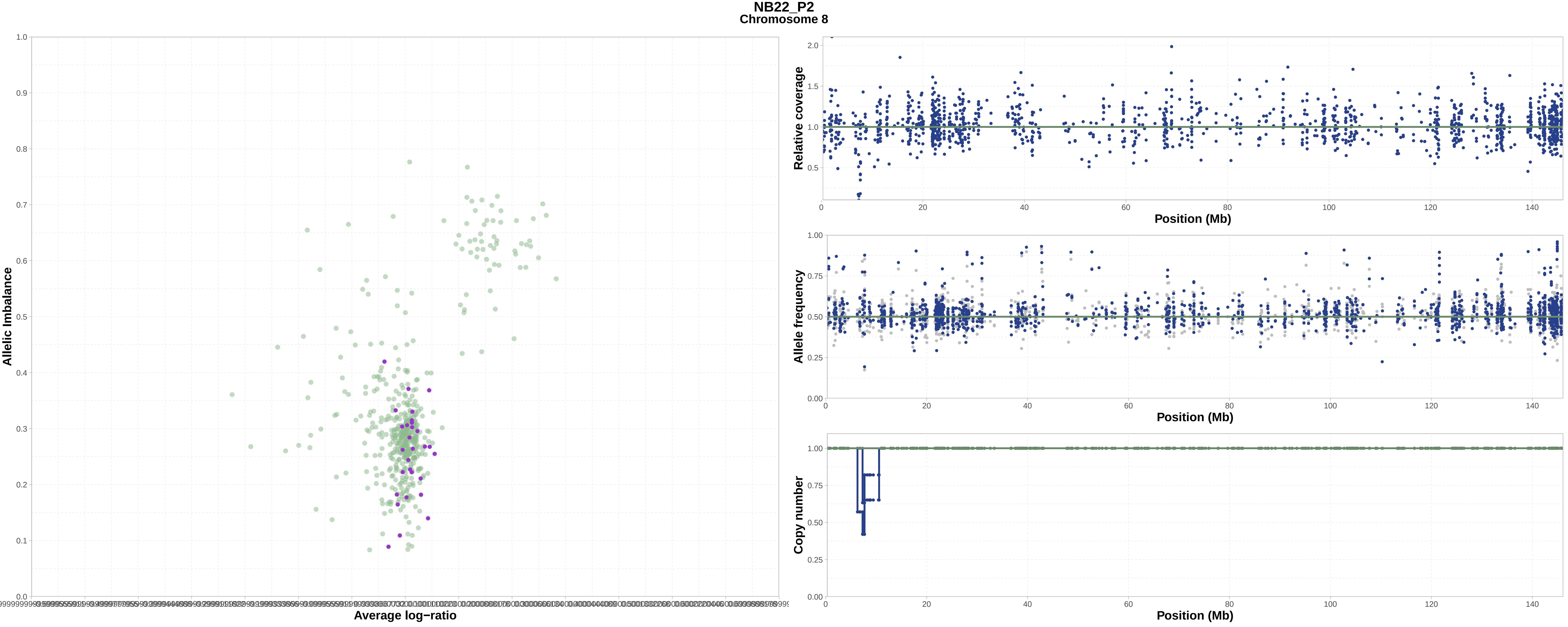
NB22_P2
Chromosome 8
Allelic Imbalance
Average log−ratio
Relative coverage
Position (Mb)
Allele frequency
Position (Mb)
Copy number
Position (Mb)

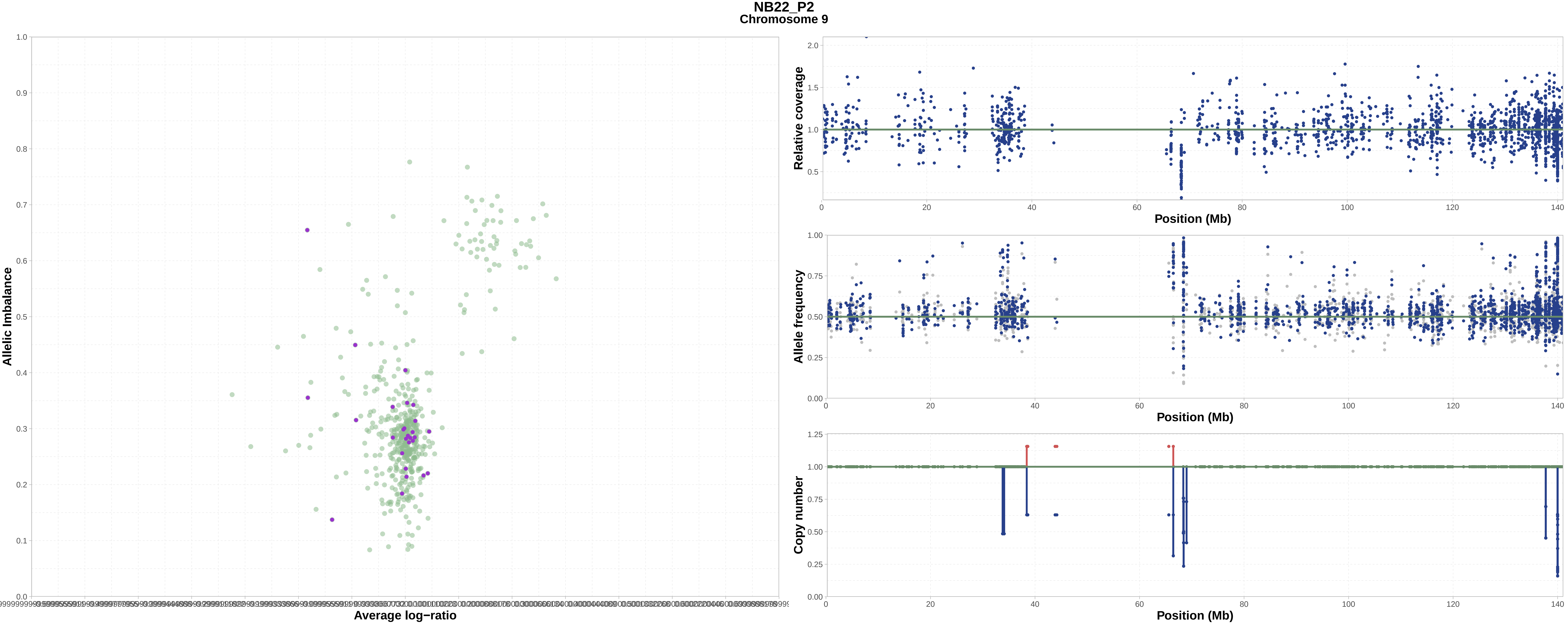
NB22_P2
Chromosome 9
Allelic Imbalance
Average log−ratio
Relative coverage
Position (Mb)
Allele frequency
Position (Mb)
Copy number
Position (Mb)

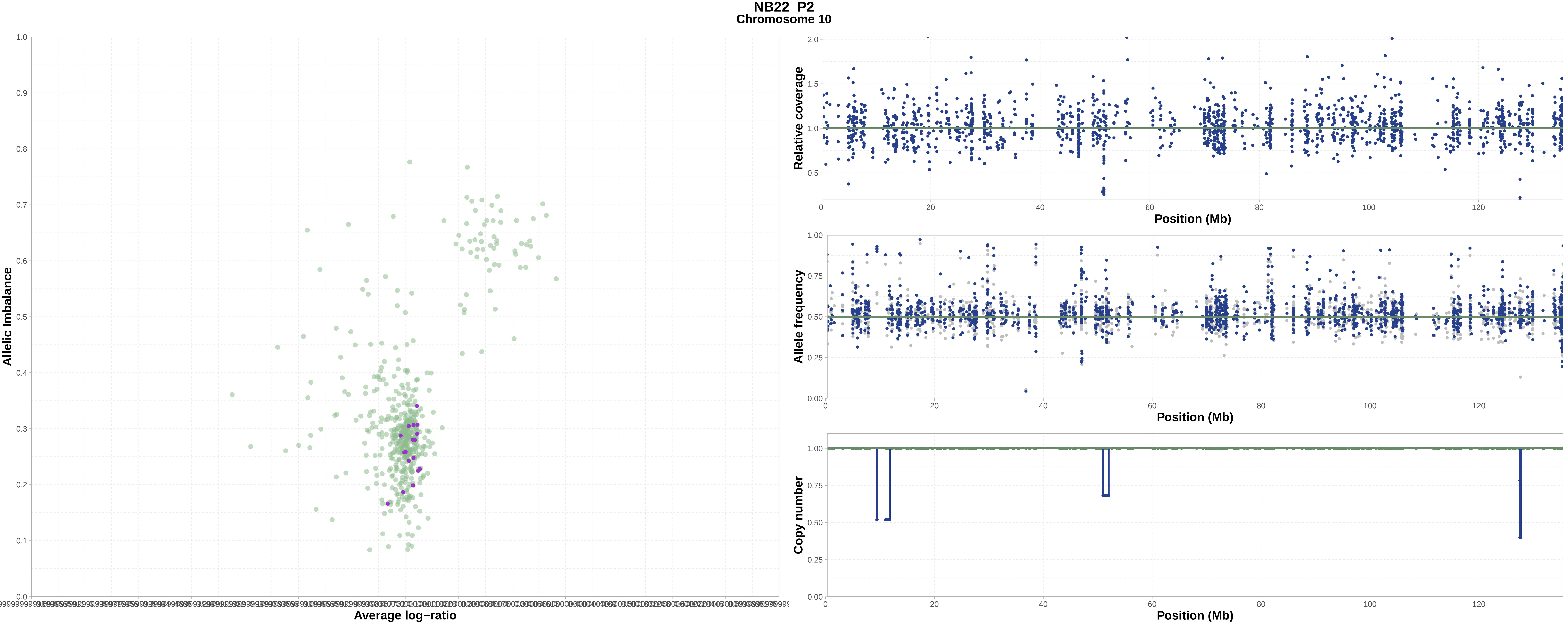
NB22_P2
Chromosome 10
Allelic Imbalance
Average log−ratio
Relative coverage
Position (Mb)
Allele frequency
Position (Mb)
Copy number
Position (Mb)

NB22_P2
Chromosome 11

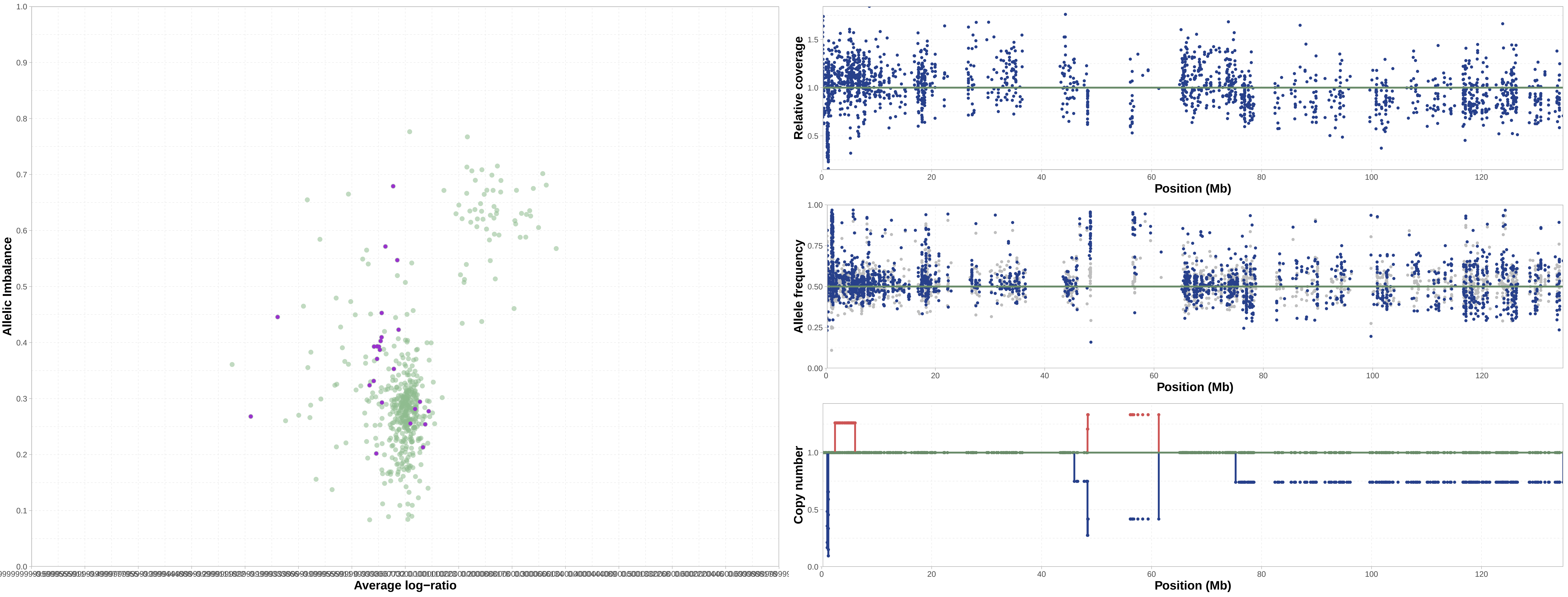

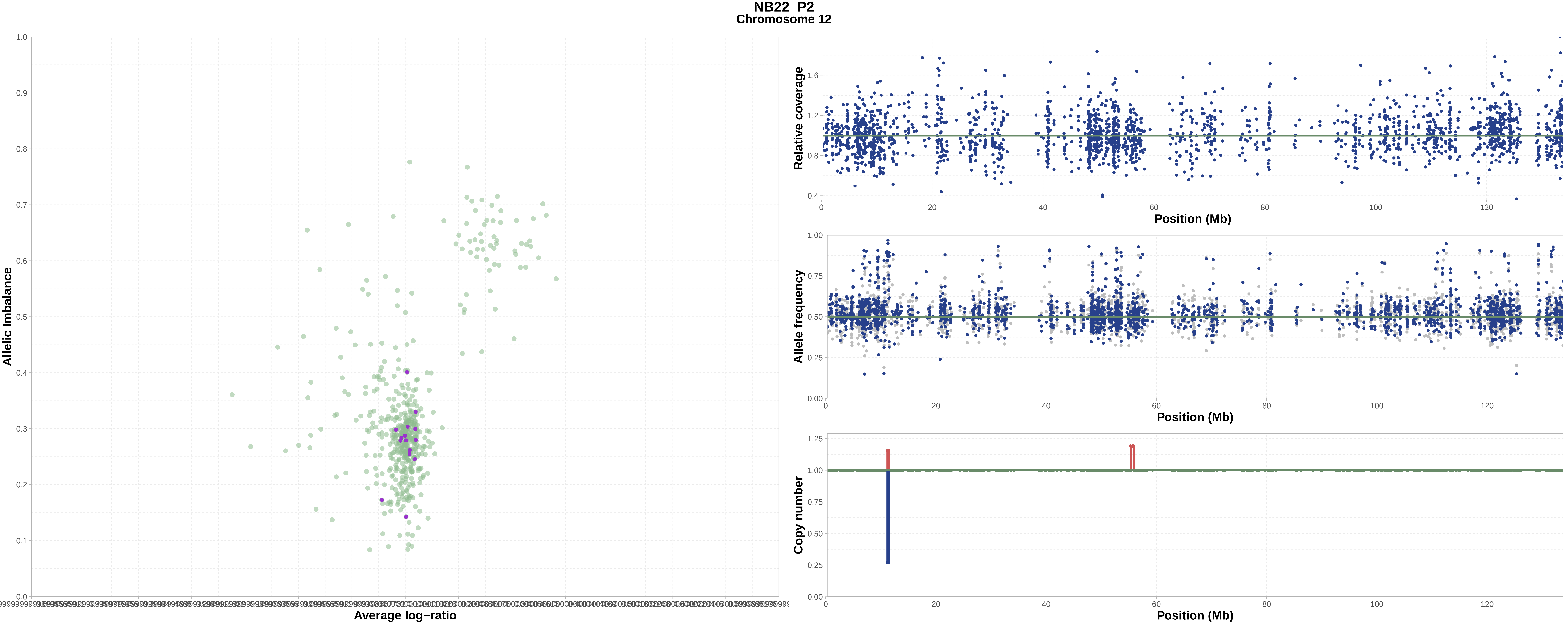

NB22_P2
Chromosome 12
Allelic Imbalance
Average log−ratio
Relative coverage
Position (Mb)
Allele frequency
Position (Mb)
Copy number
Position (Mb)

NB22_P2
Chromosome 13

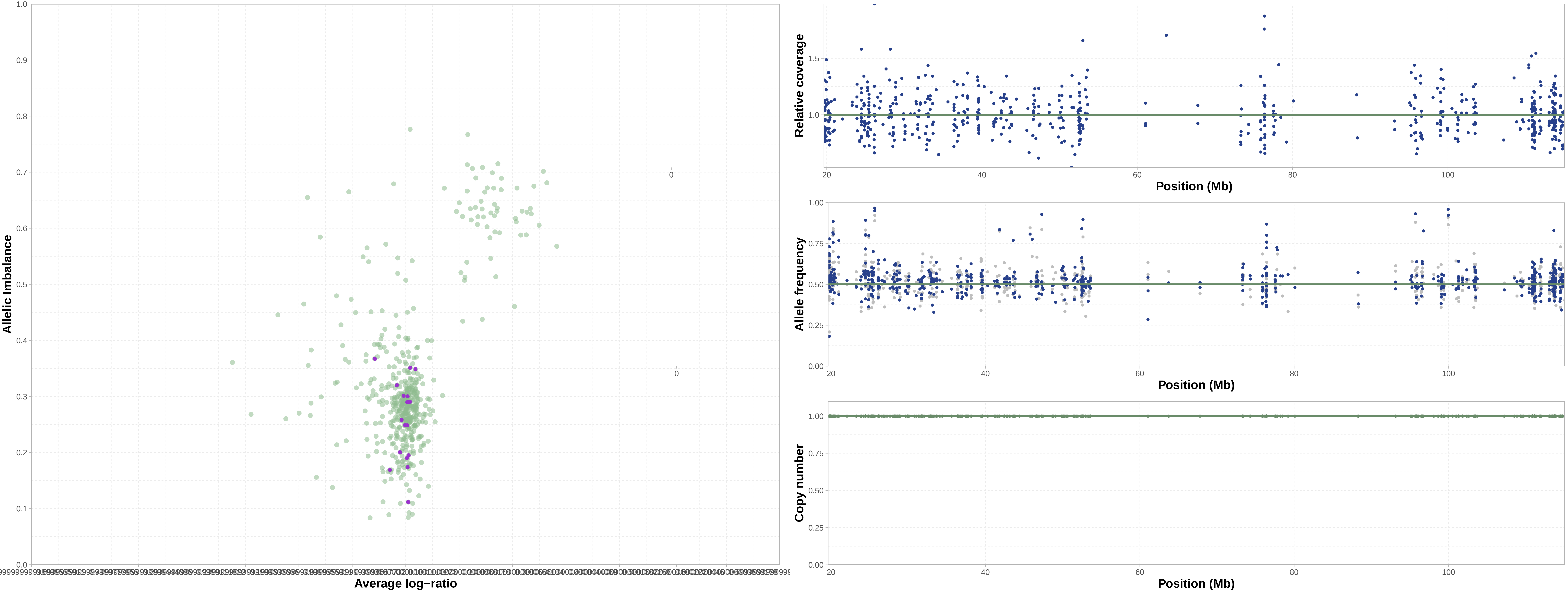

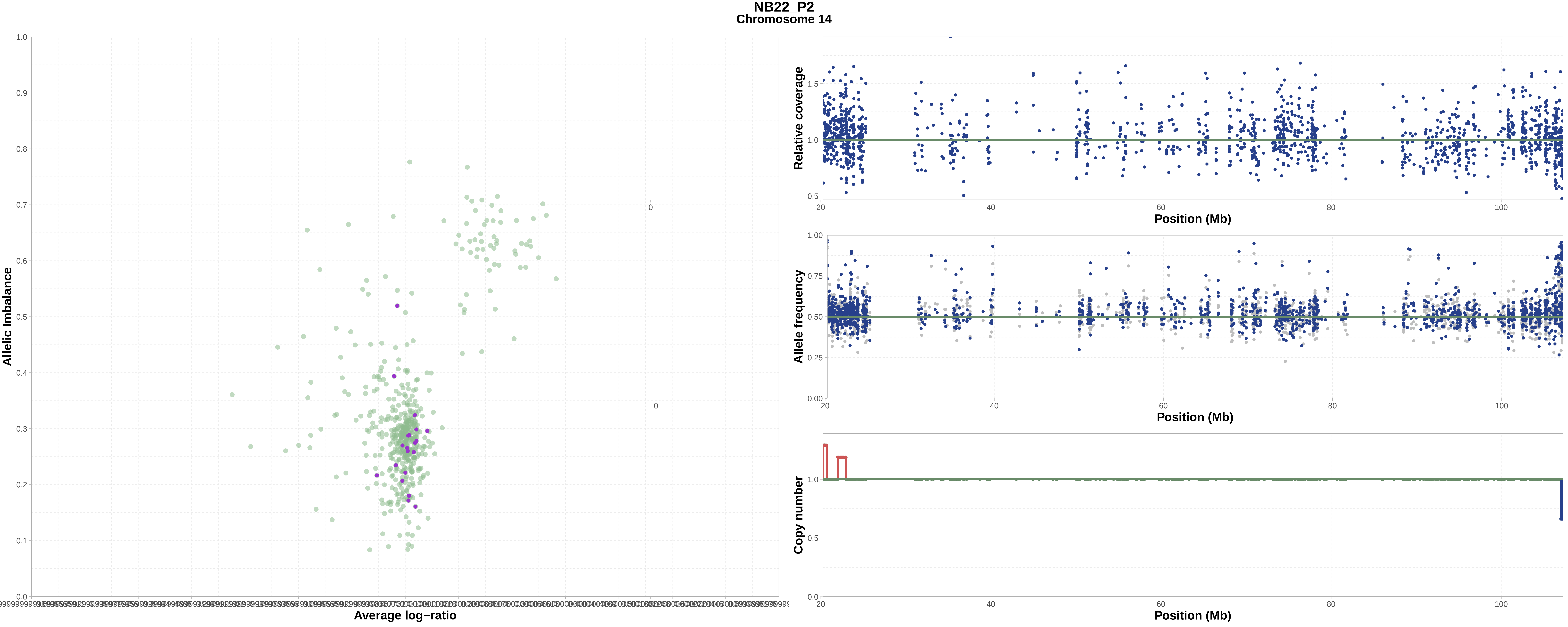
NB22_P2
Chromosome 14
Allelic Imbalance
Average log−ratio
Relative coverage
Position (Mb)
Allele frequency
Position (Mb)
Copy number
Position (Mb)

NB22_P2
Chromosome 15

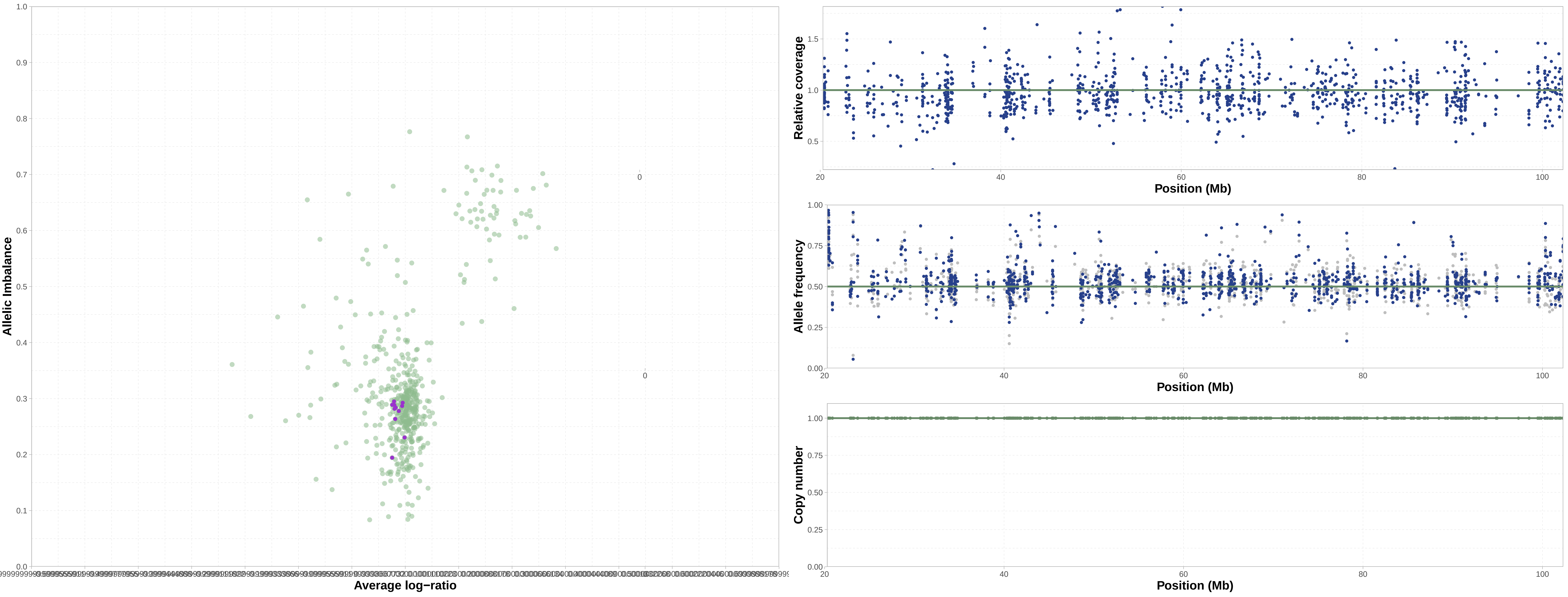

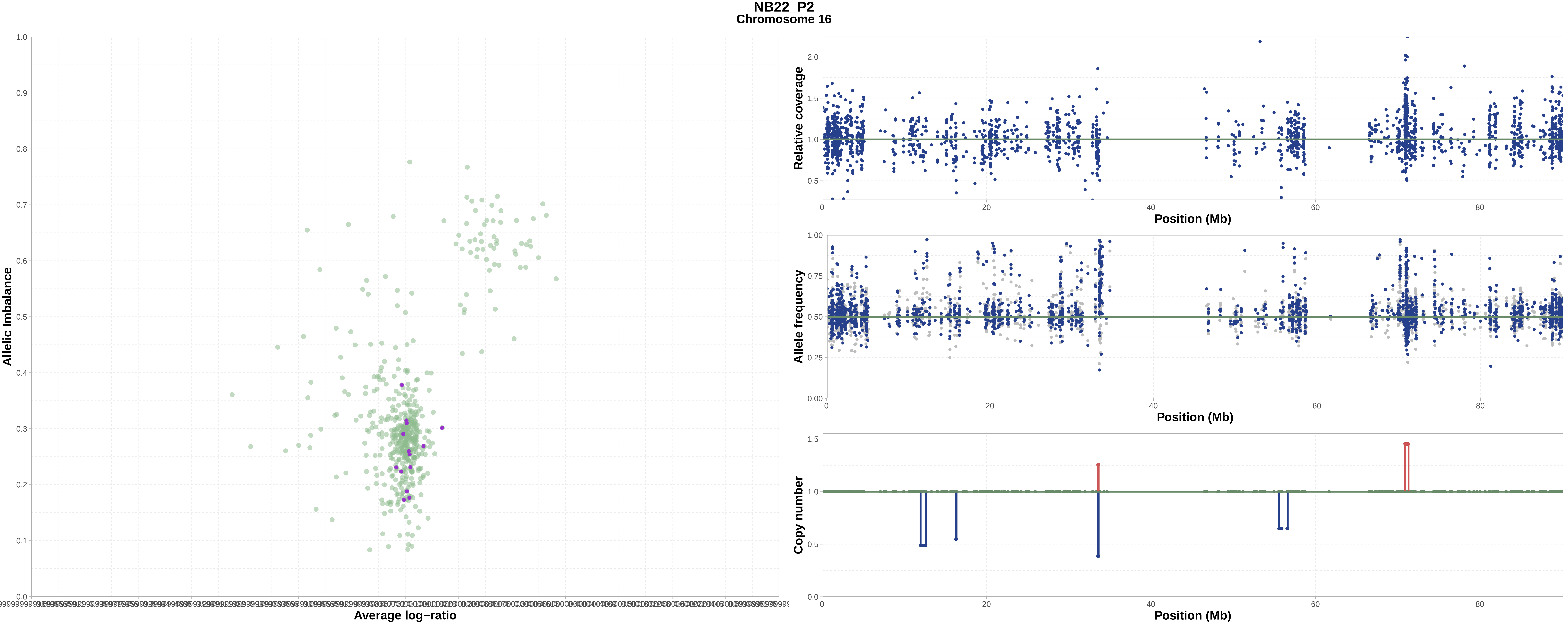
NB22_P2
Chromosome 16
Allelic Imbalance
Average log−ratio
Relative coverage
Allele frequency
Copy number
Position (Mb)
Position (Mb)
Position (Mb)

NB22_P2
Chromosome 17

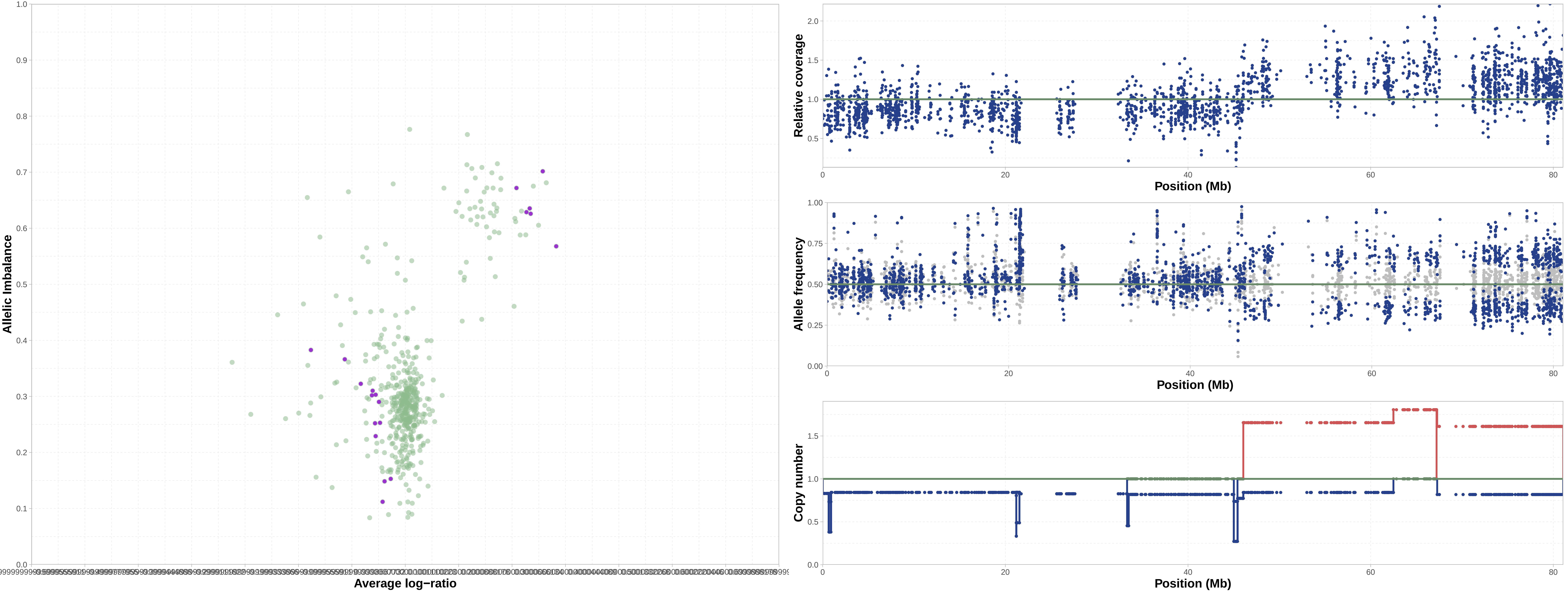

NB22_P2
Chromosome 18

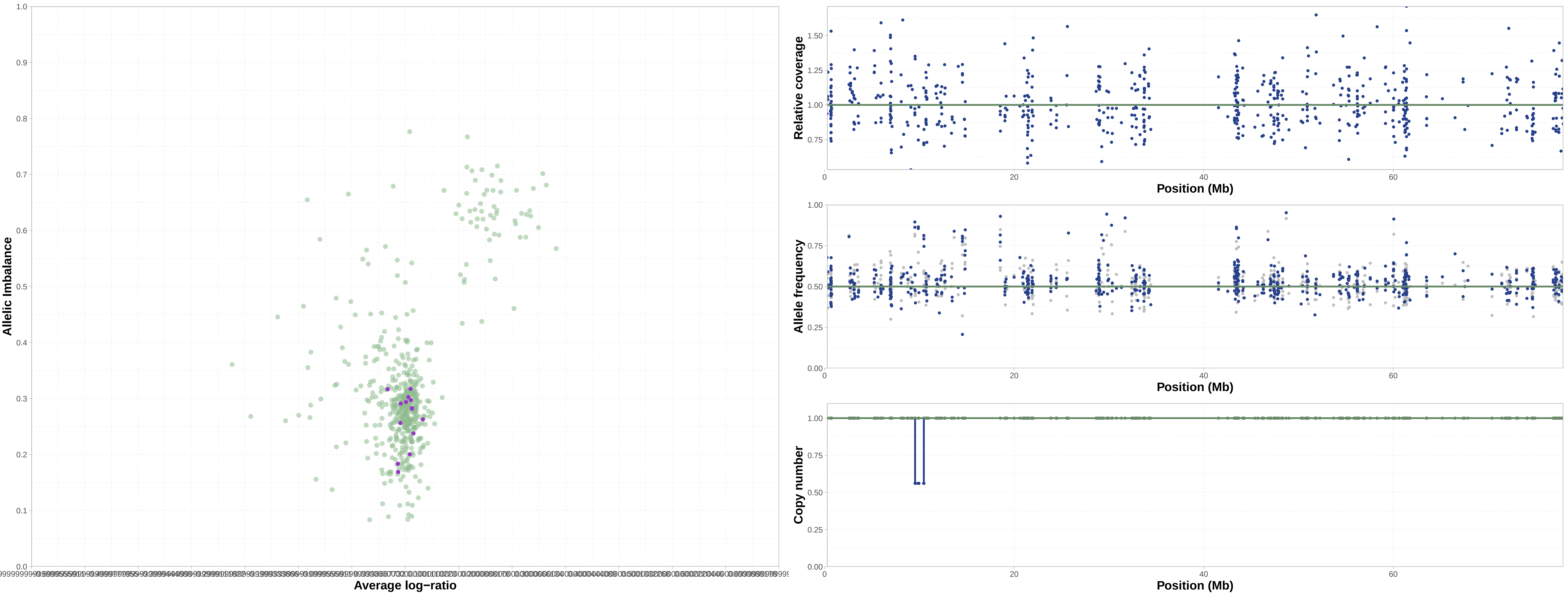

NB22_P2
Chromosome 19

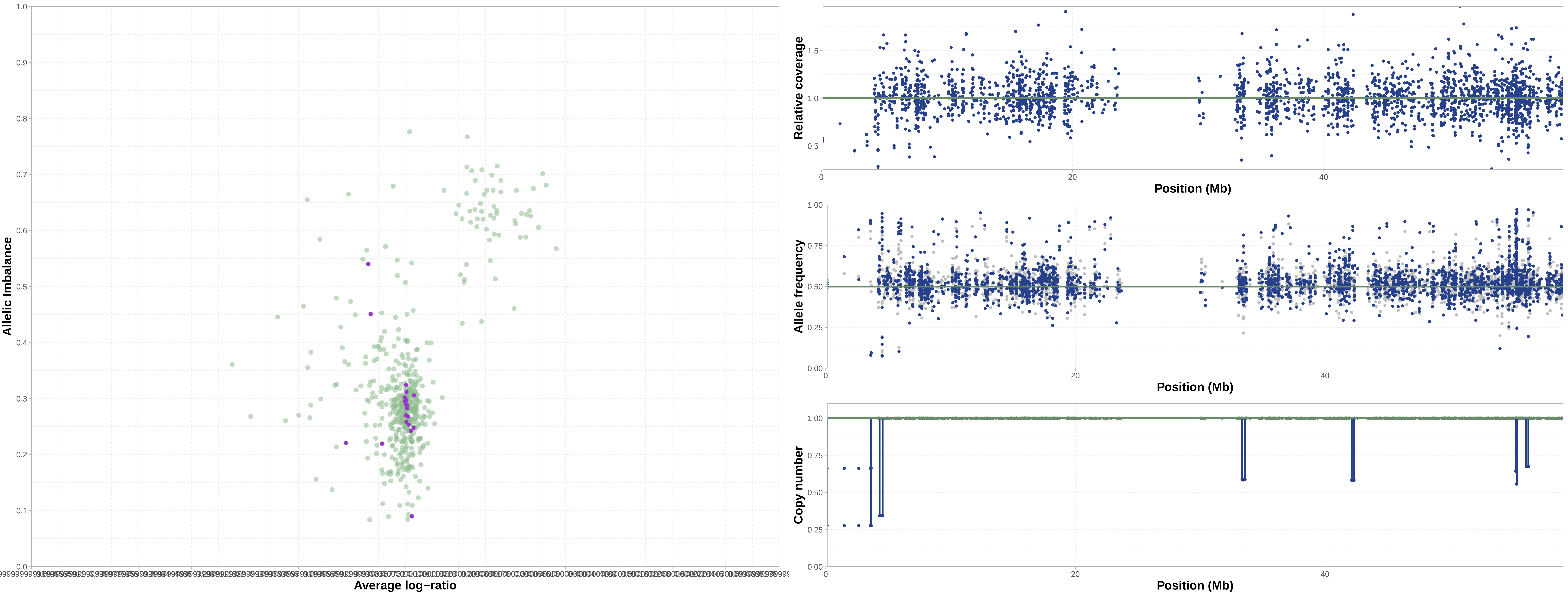

NB22_P2
Chromosome 20

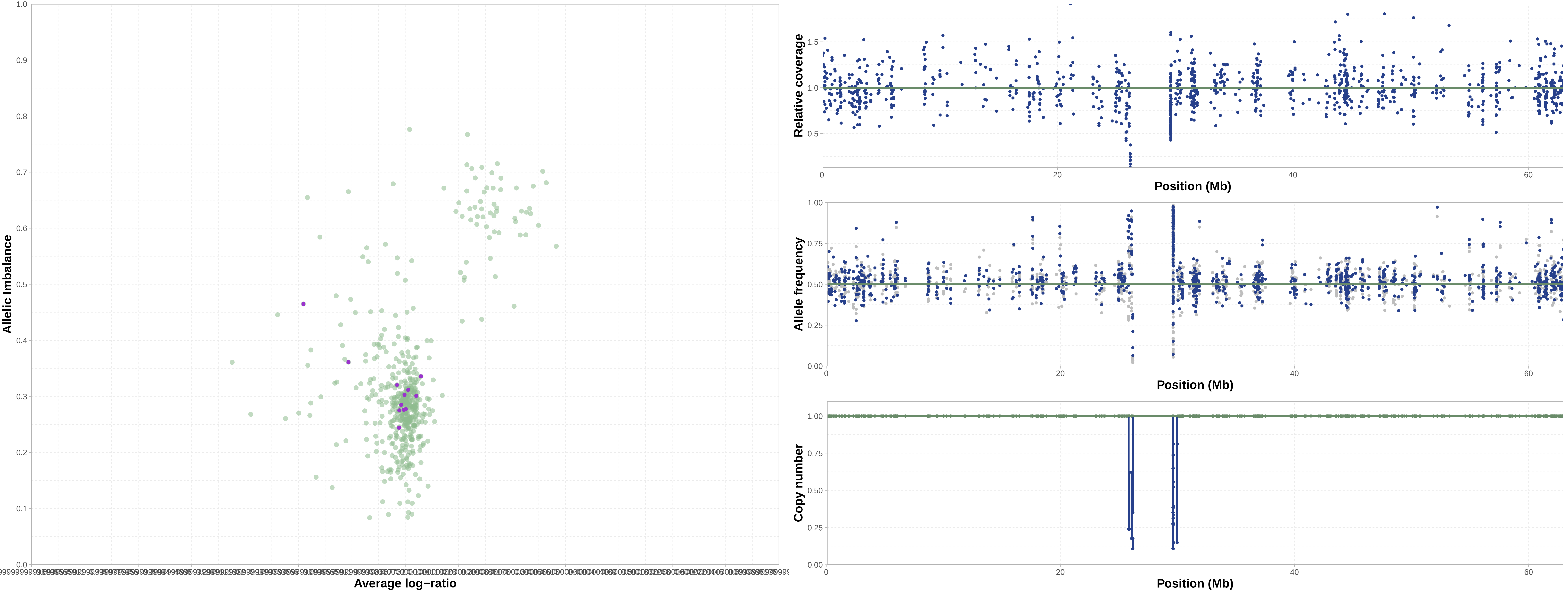

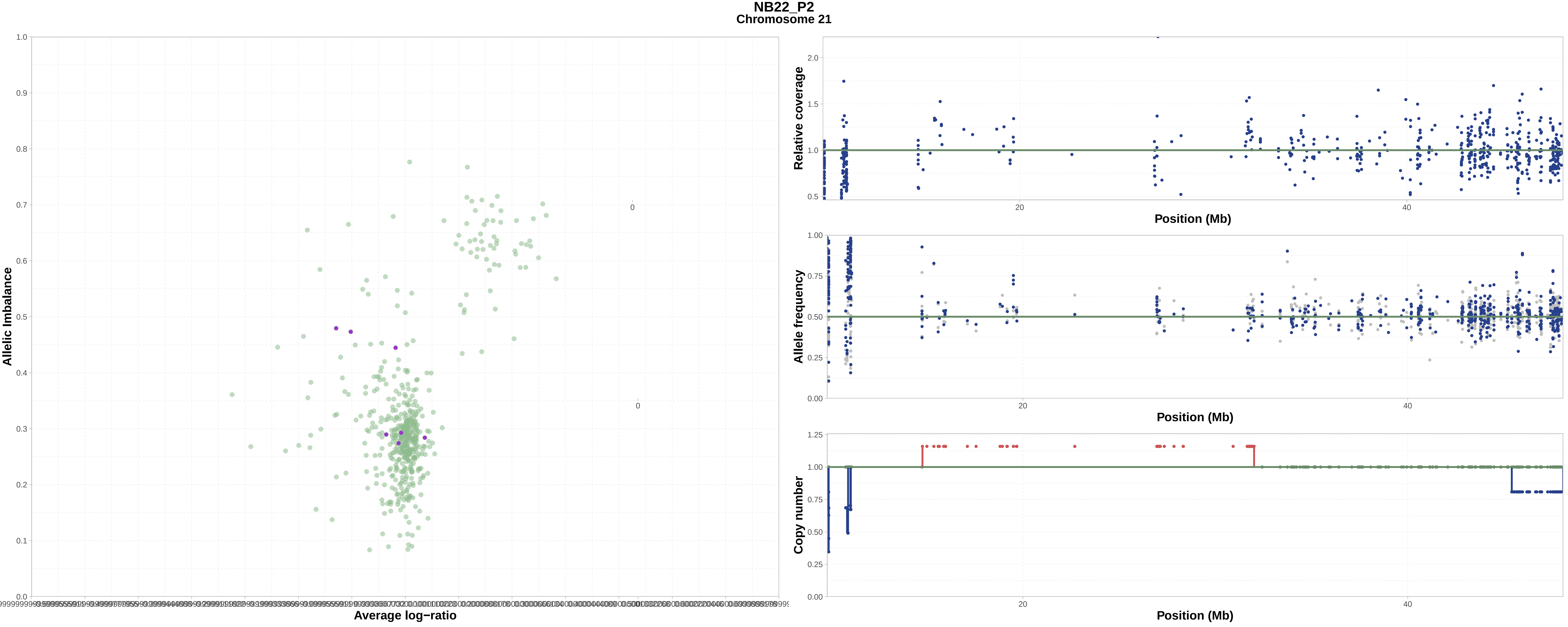

NB22_P2
Chromosome 21
Allelic Imbalance
Average log−ratio
Relative coverage
Position (Mb)
Allele frequency
Position (Mb)
Copy number
Position (Mb)

NB22_P2
Chromosome 22

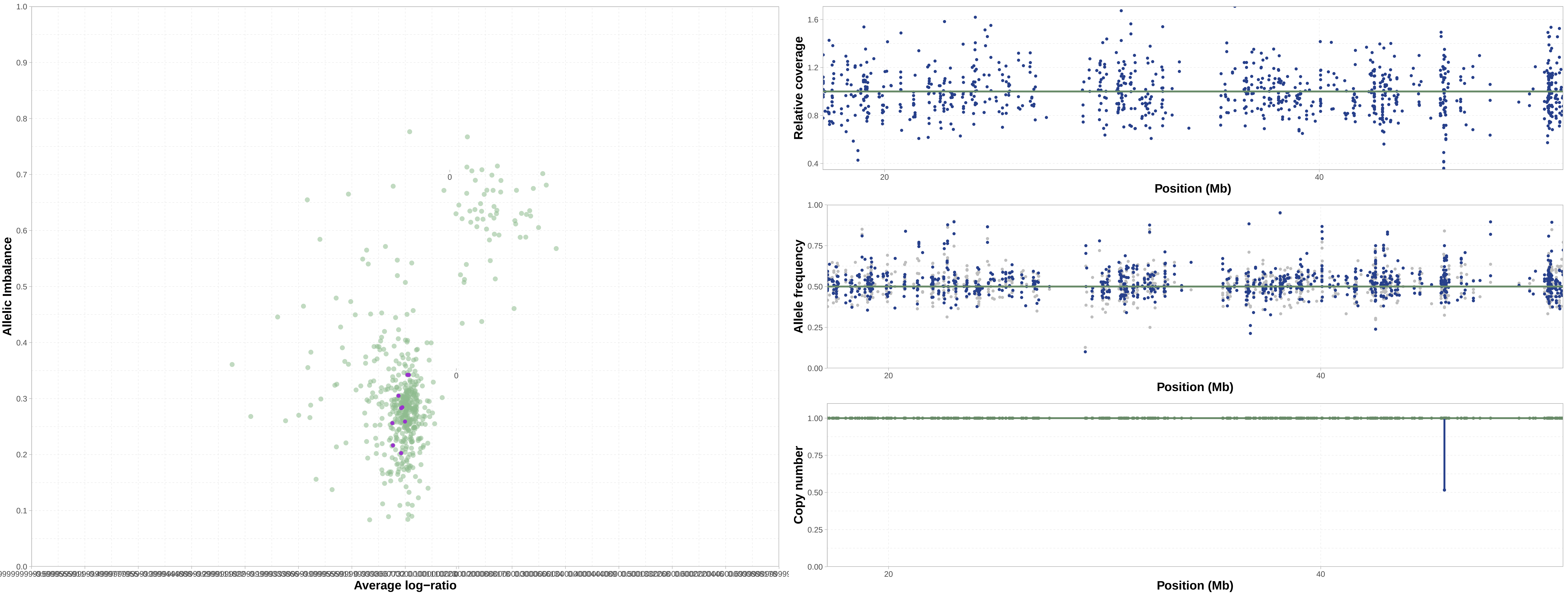

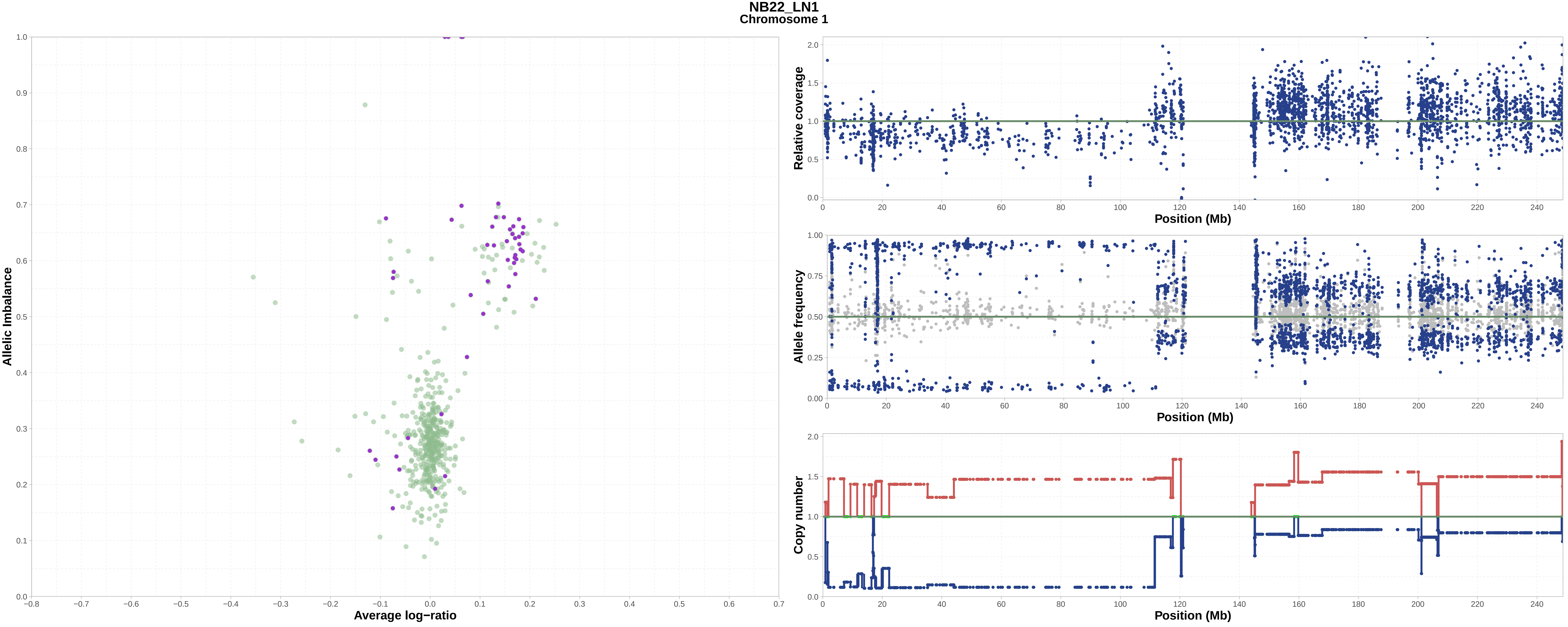
NB22_LN1
Chromosome 1
Allelic Imbalance
Average log−ratio
Relative coverage
Position (Mb)
Allele frequency
Position (Mb)
Copy number
Position (Mb)

NB22_LN1
Chromosome 2

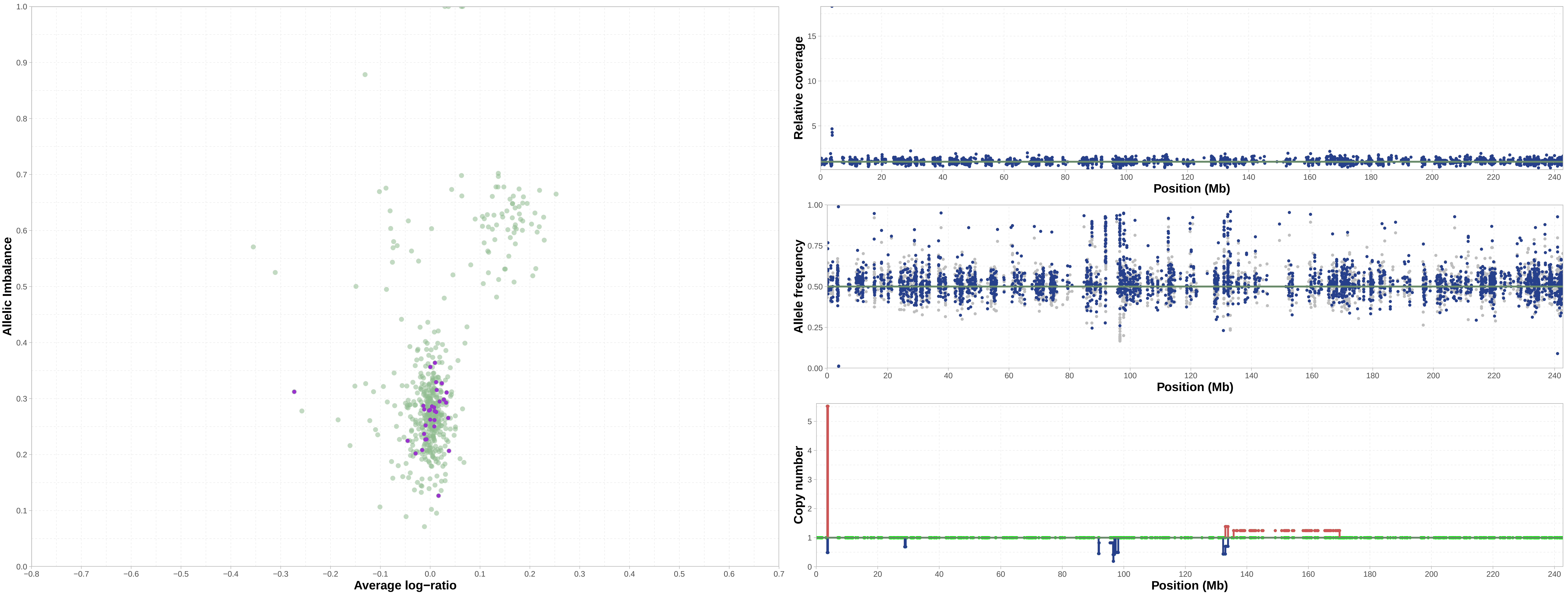

NB22_LN1
Chromosome 3

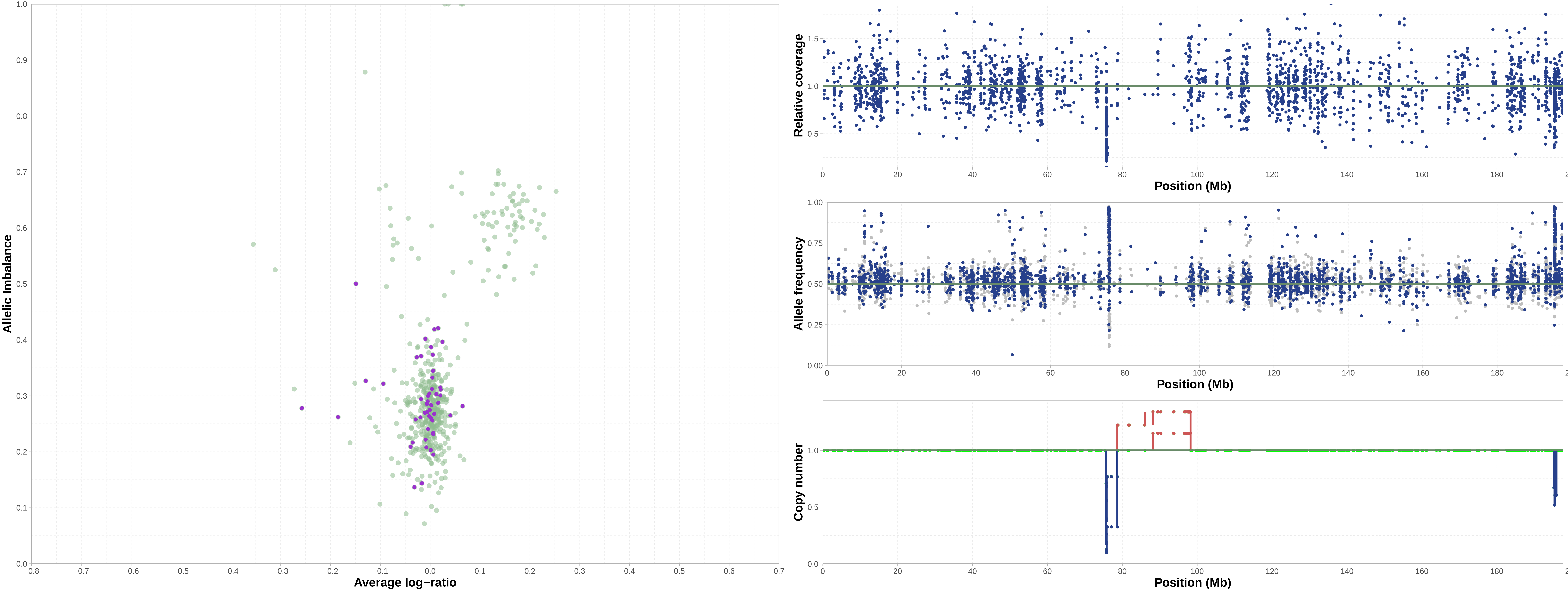

NB22_LN1
Chromosome 4

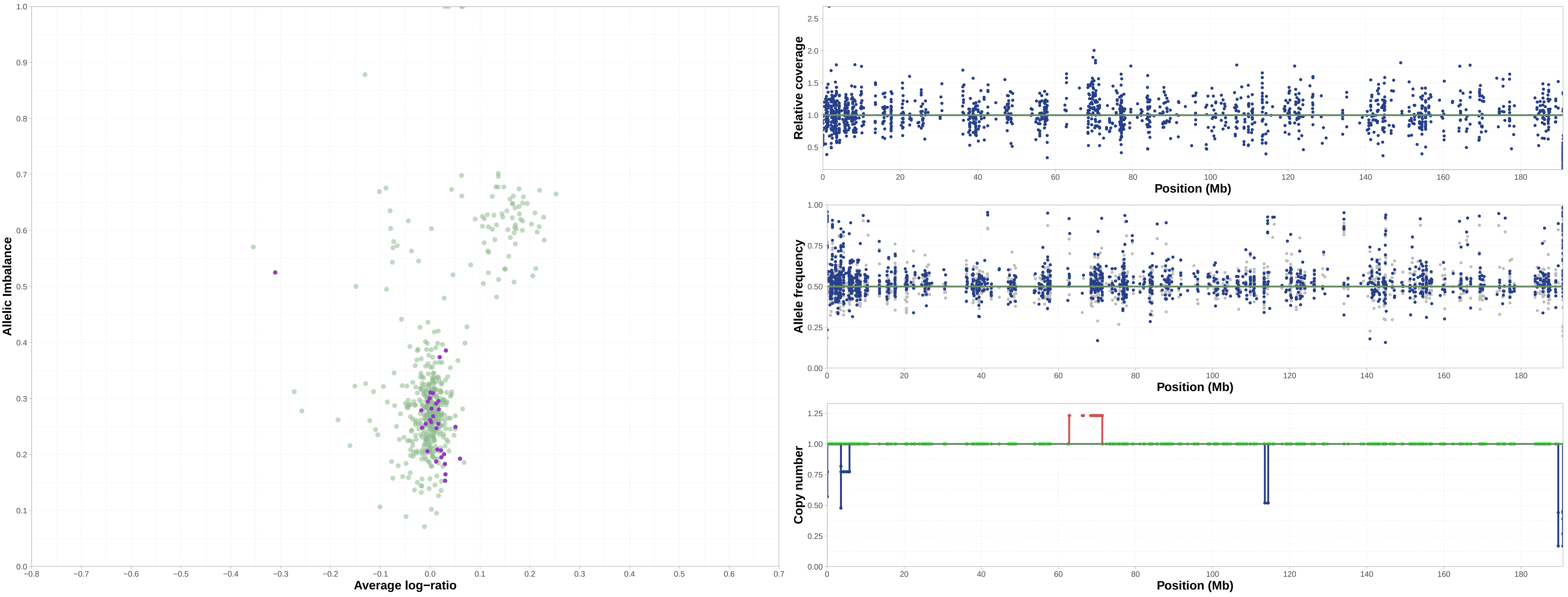

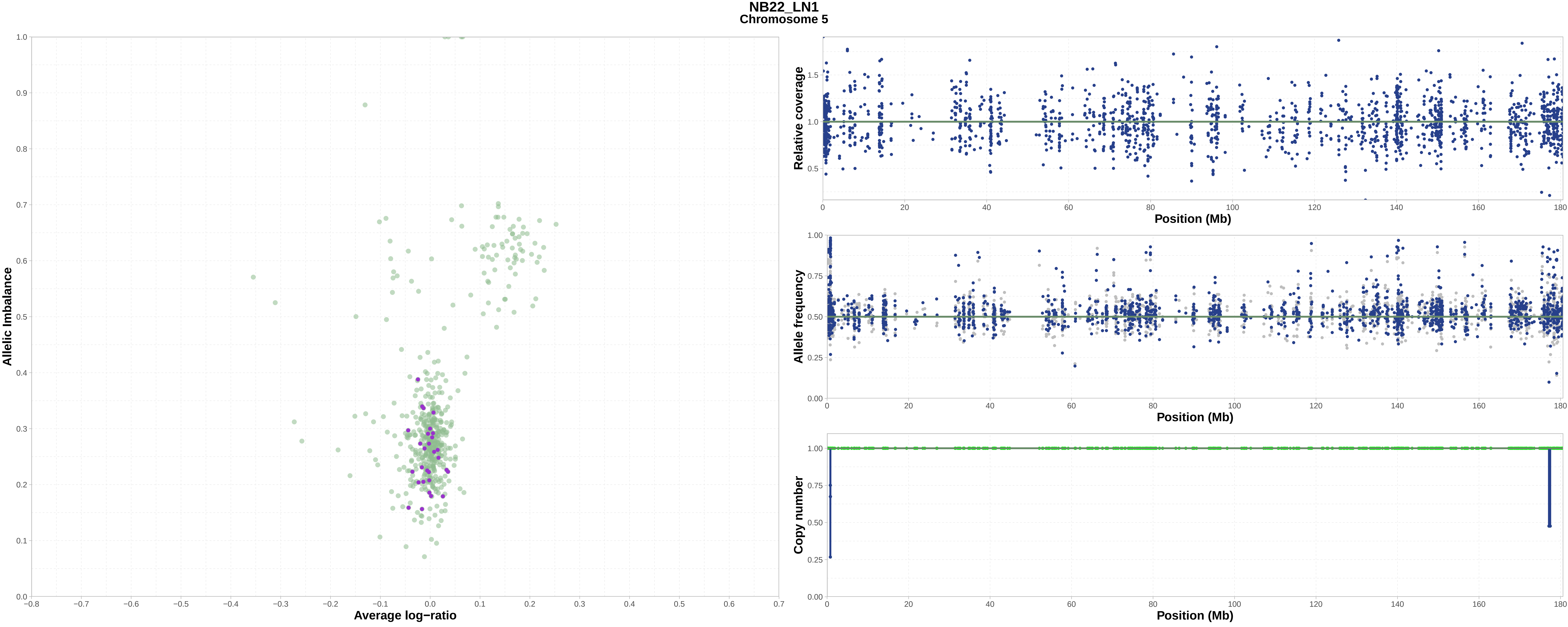
NB22_LN1
Chromosome 5
Allelic Imbalance
Average log−ratio
Relative coverage
Position (Mb)
Allele frequency
Position (Mb)
Copy number
Position (Mb)

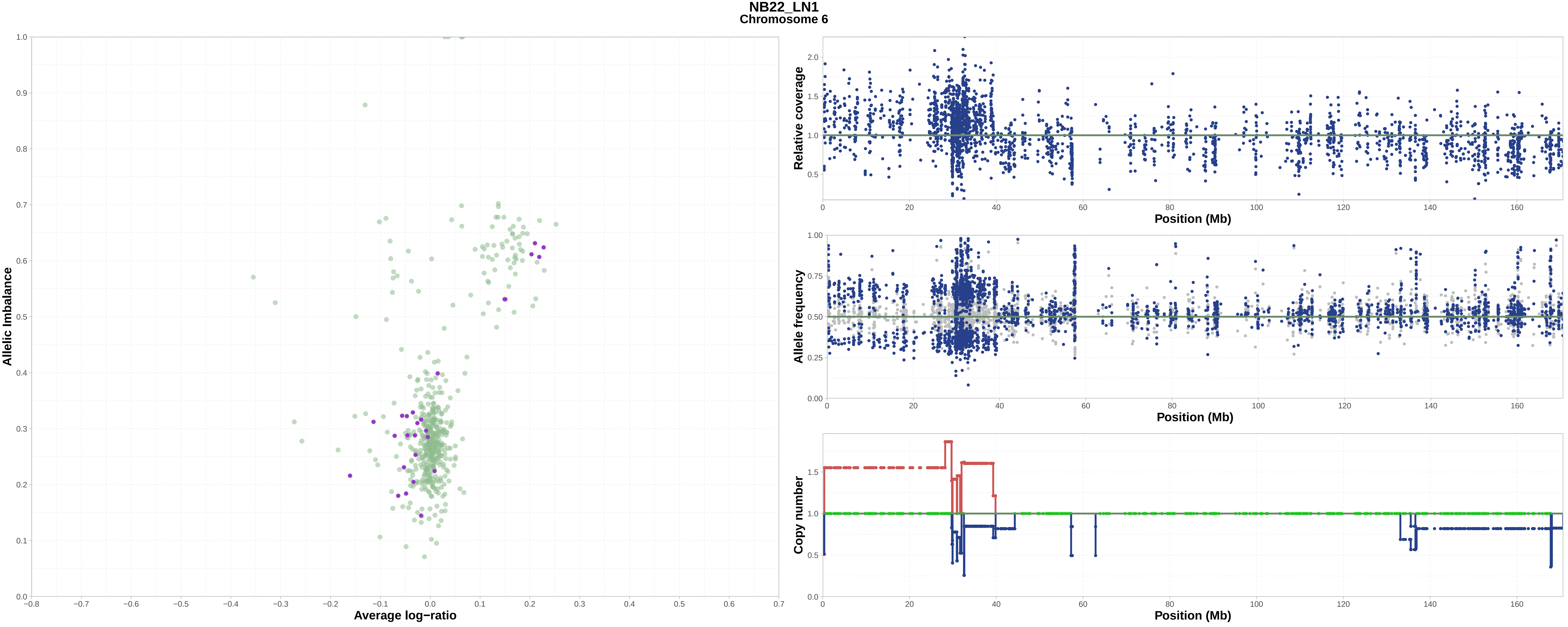
NB22_LN1
Chromosome 6
Allelic Imbalance
Average log−ratio
Relative coverage
Position (Mb)
Allele frequency
Position (Mb)
Copy number
Position (Mb)

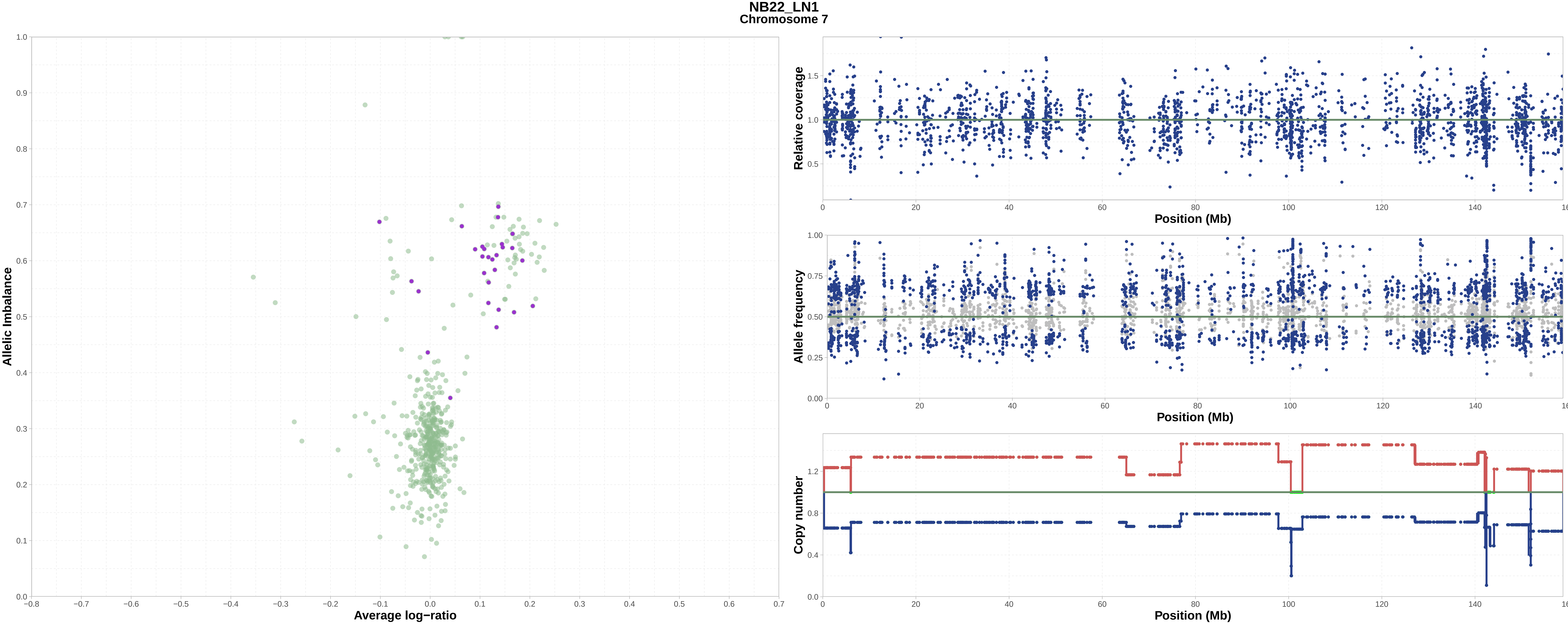
NB22_LN1
Chromosome 7
Allelic Imbalance
Average log−ratio
Relative coverage
Allele frequency
Copy number
Position (Mb)
Position (Mb)
Position (Mb)

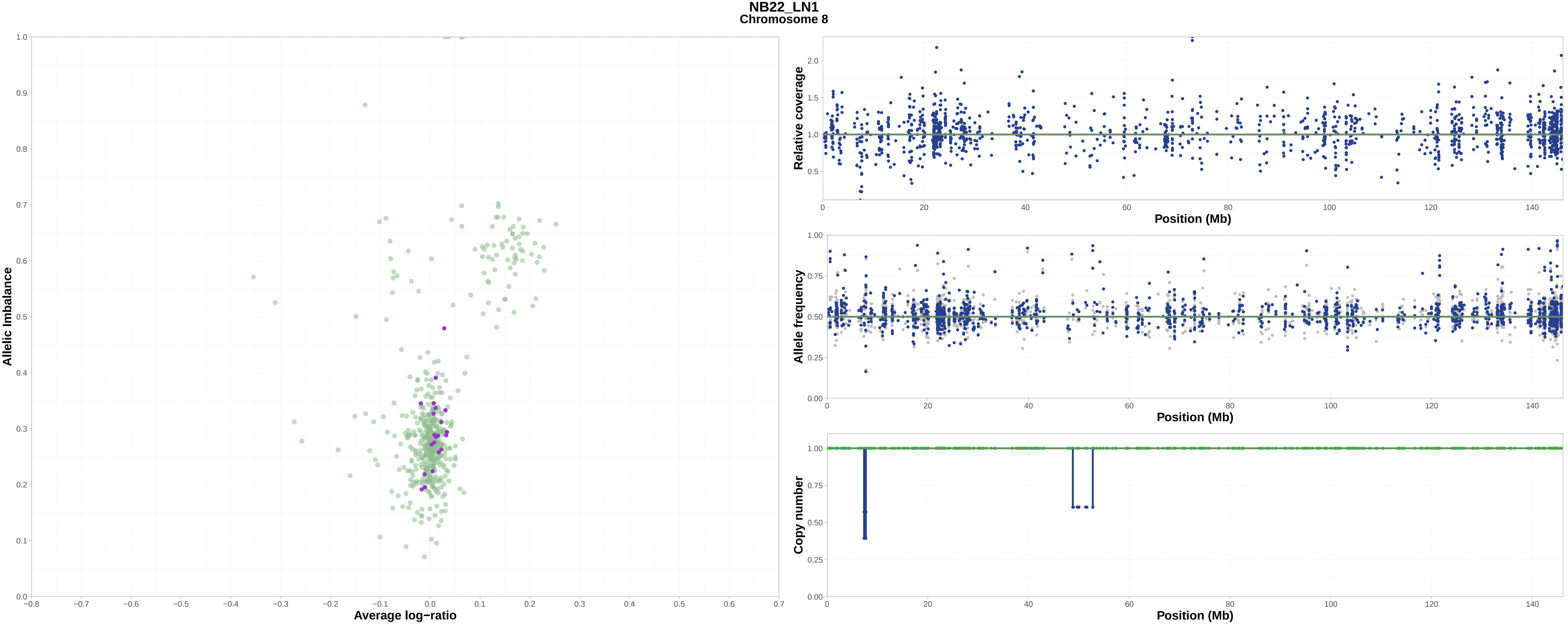
NB22_LN1
Chromosome 8
Allelic Imbalance
Average log−ratio
Relative coverage
Position (Mb)
Allele frequency
Position (Mb)
Copy number
Position (Mb)

NB22_LN1
Chromosome 9

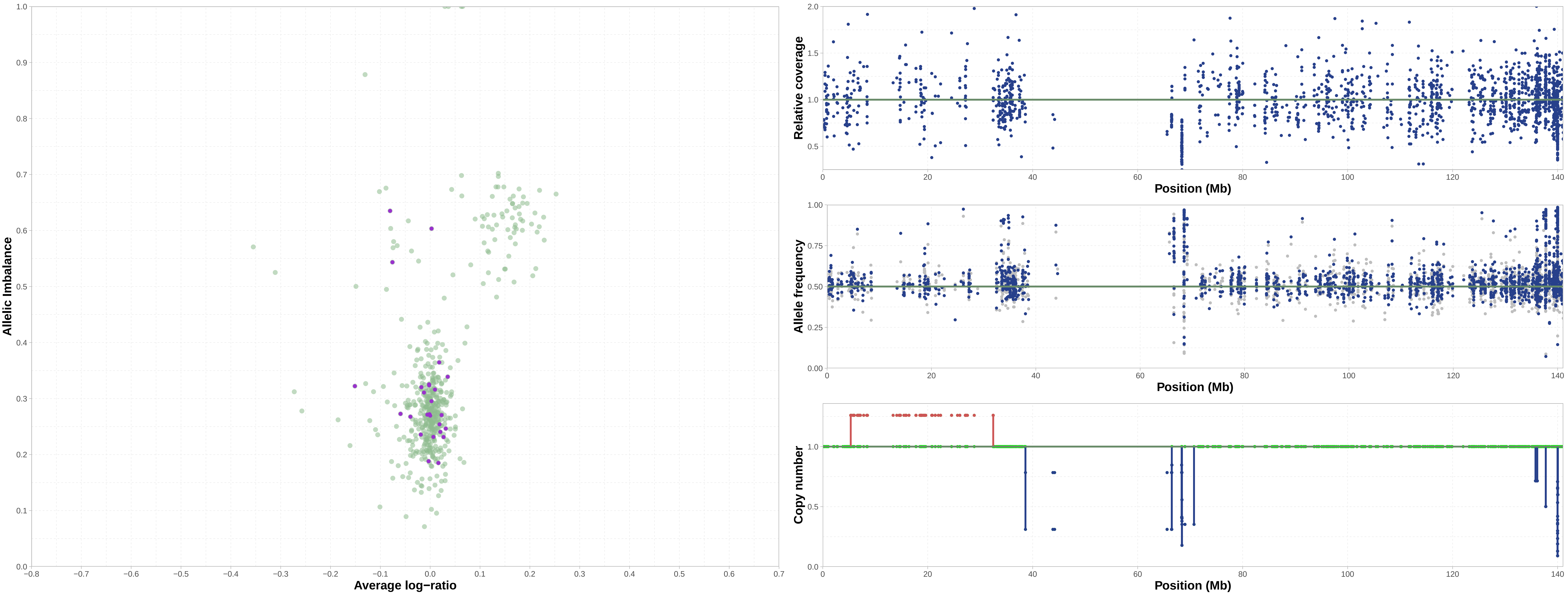

NB22_LN1
Chromosome 10

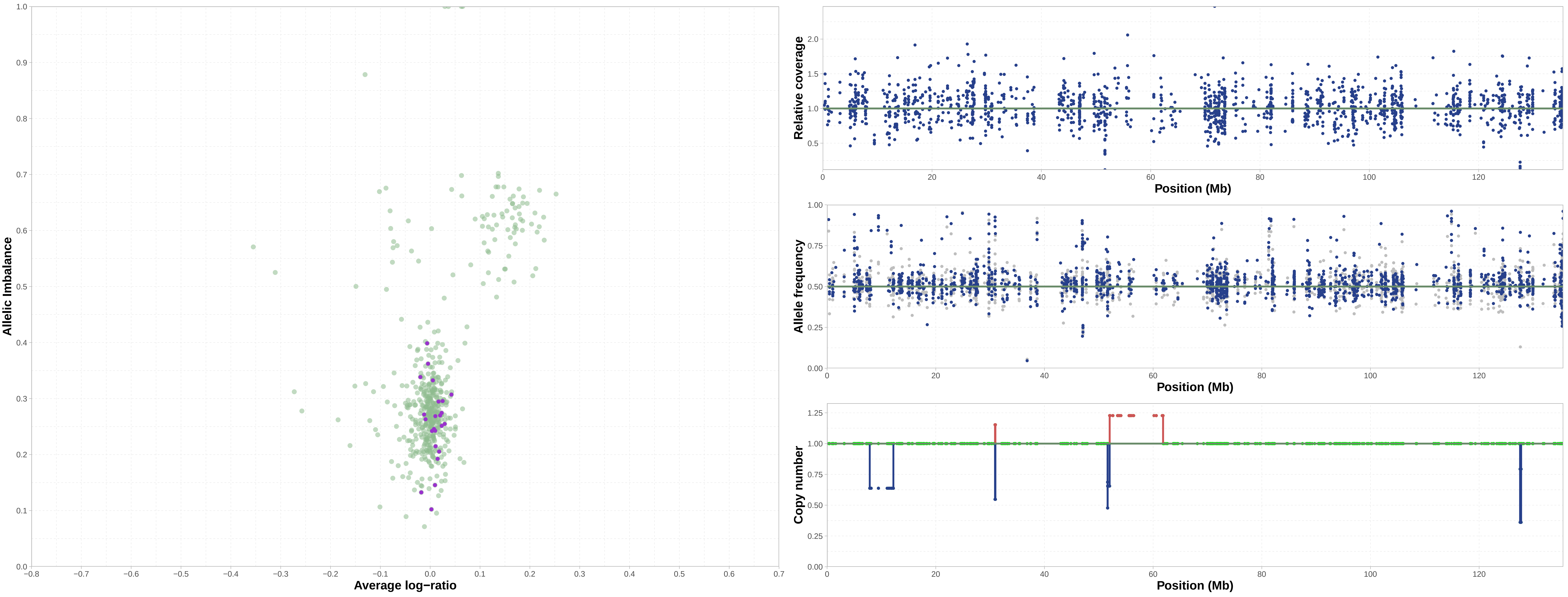

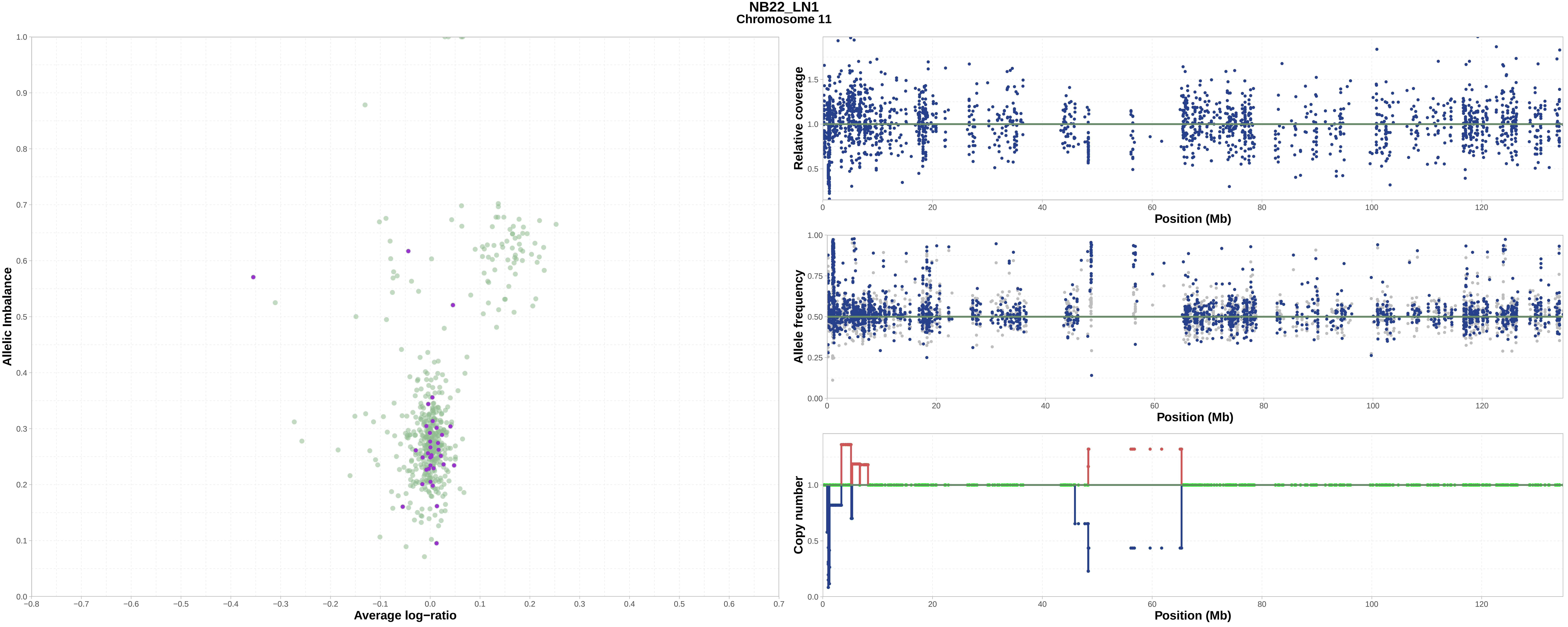
NB22_LN1
Chromosome 11
Allelic Imbalance
Average log−ratio
Relative coverage
Position (Mb)
Allele frequency
Position (Mb)
Copy number
Position (Mb)

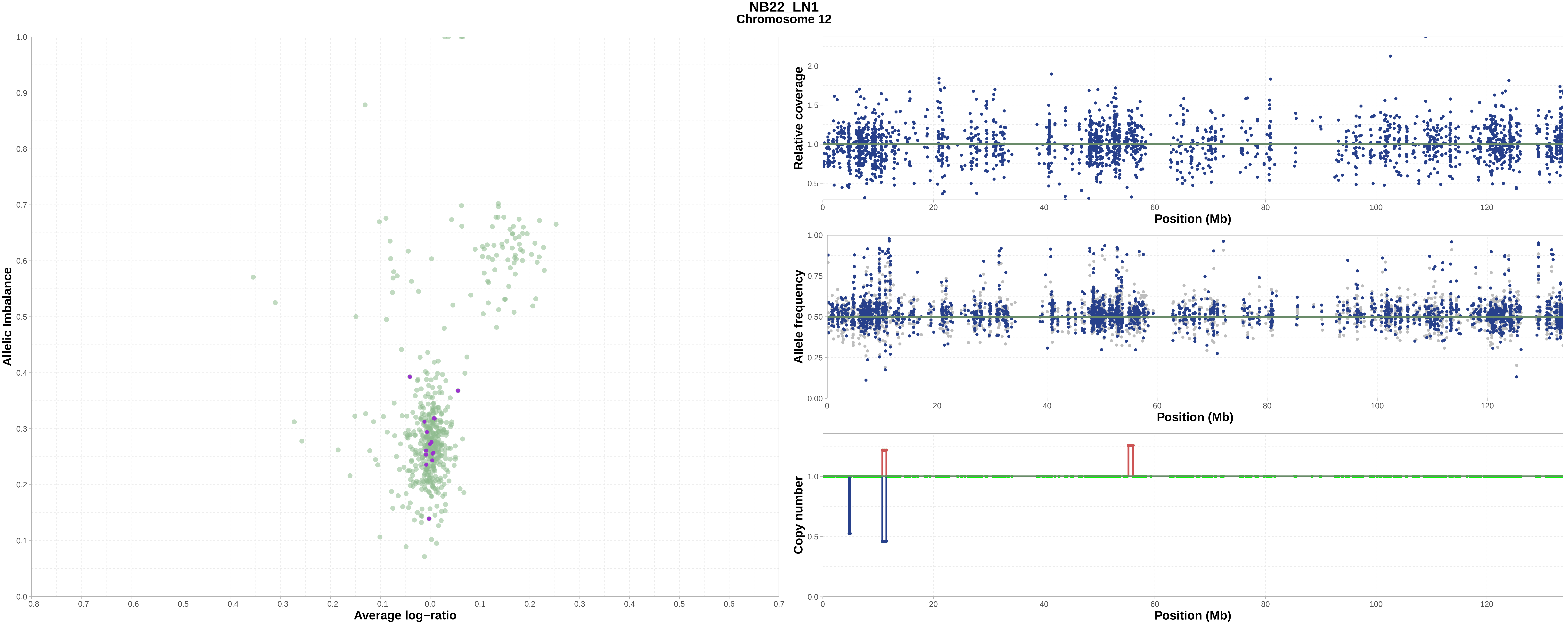

NB22_LN1
Chromosome 12
Allelic Imbalance
Average log−ratio
Relative coverage
Position (Mb)
Allele frequency
Position (Mb)
Copy number
Position (Mb)

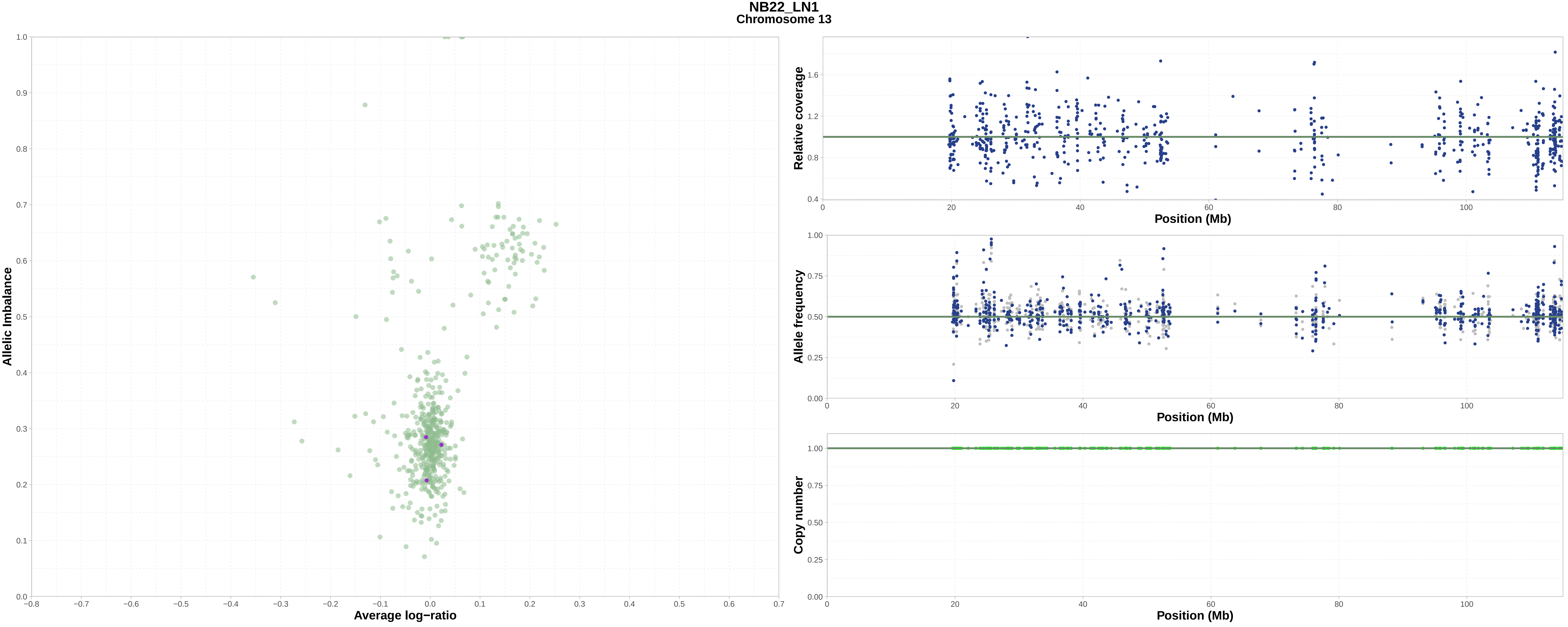
NB22_LN1
Chromosome 13
Allelic Imbalance
Average log−ratio
Relative coverage
Position (Mb)
Allele frequency
Position (Mb)
Copy number
Position (Mb)

NB22_LN1
Chromosome 14

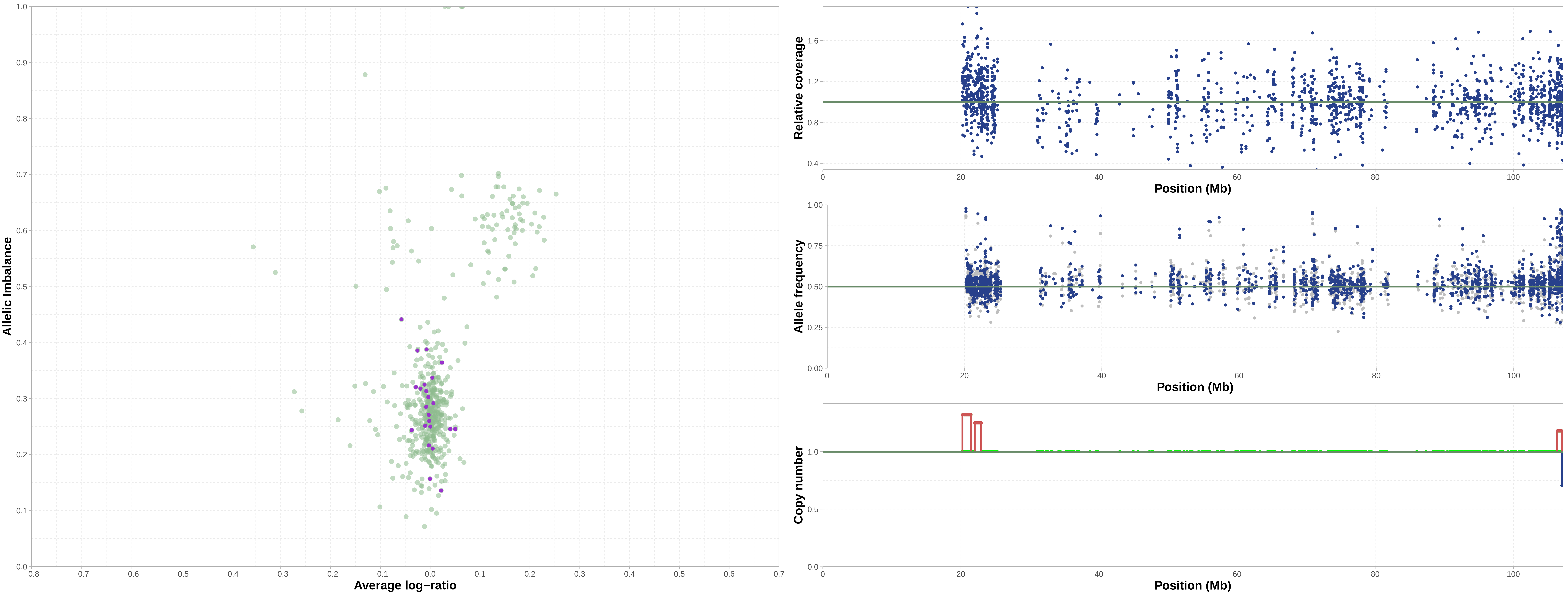

NB22_LN1
Chromosome 15

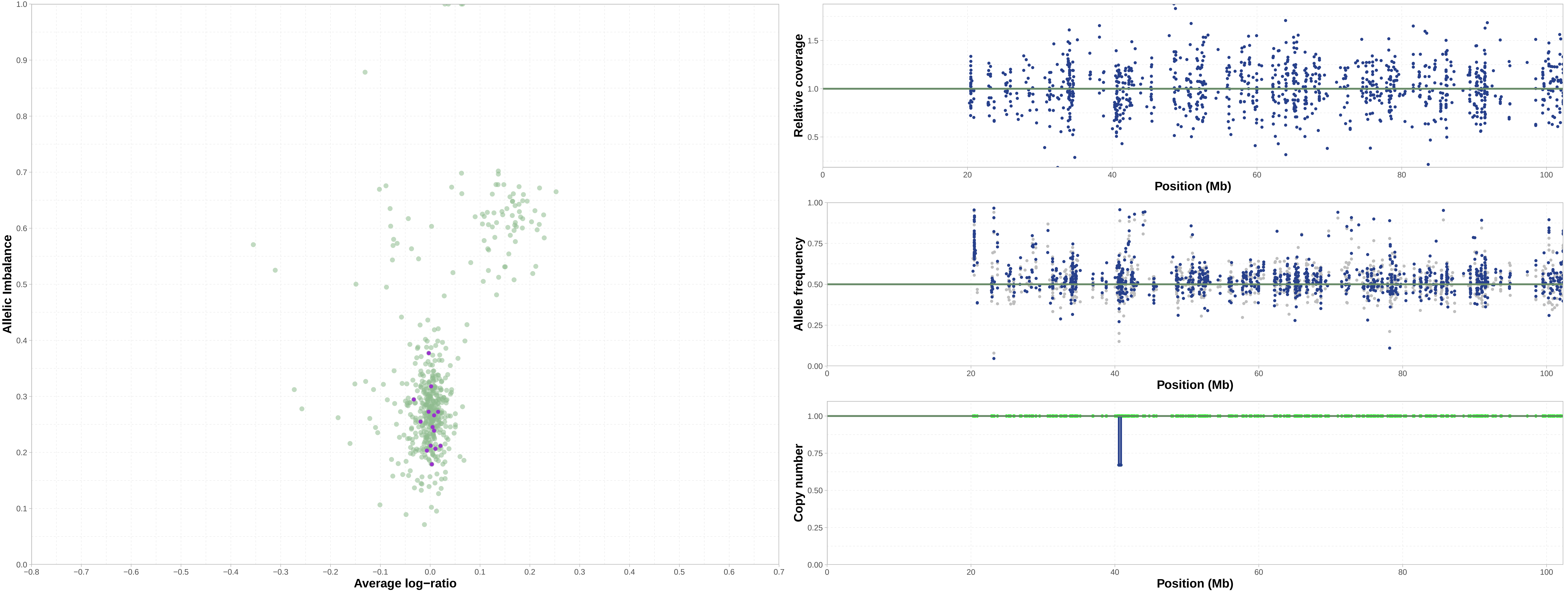

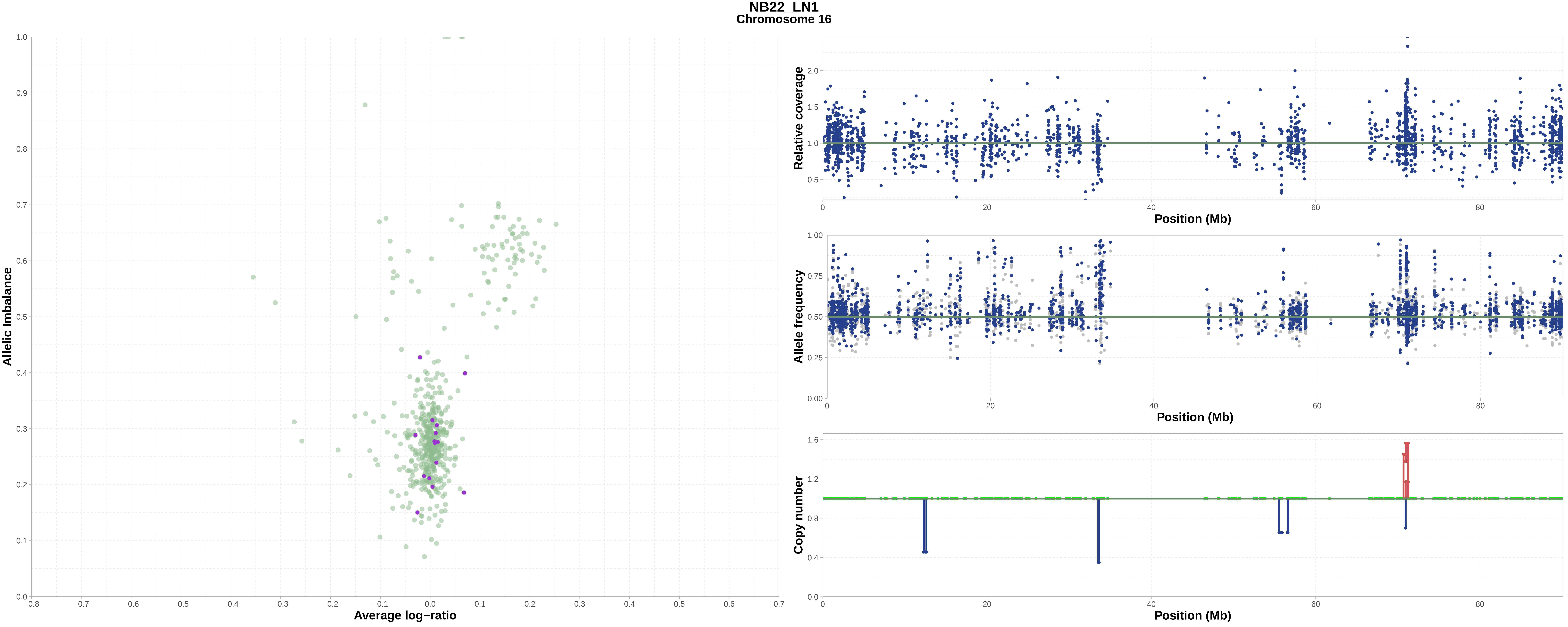

NB22_LN1
Chromosome 16
Allelic Imbalance
Average log−ratio
Relative coverage
Position (Mb)
Allele frequency
Position (Mb)
Copy number
Position (Mb)

NB22_LN1
Chromosome 17

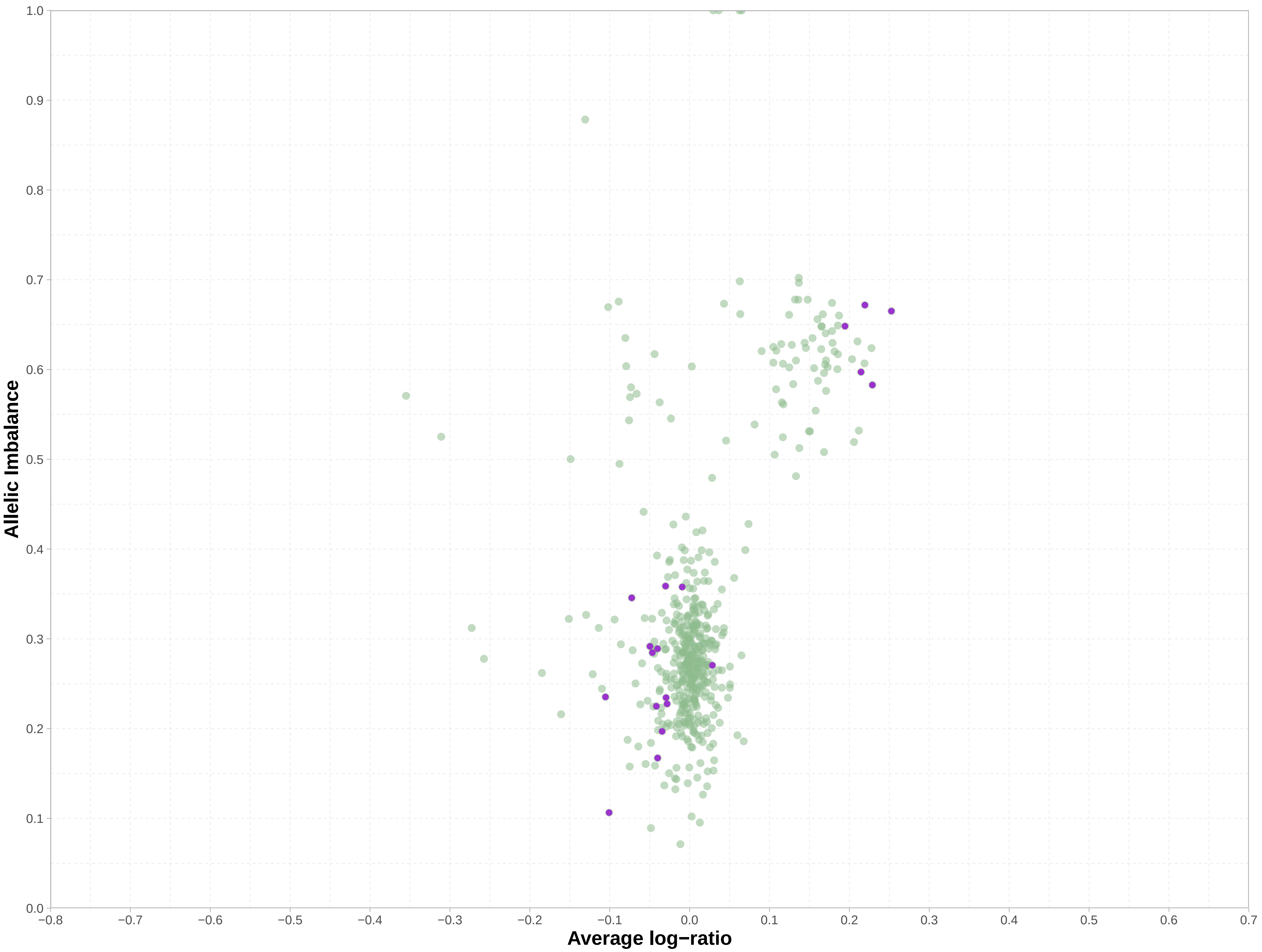

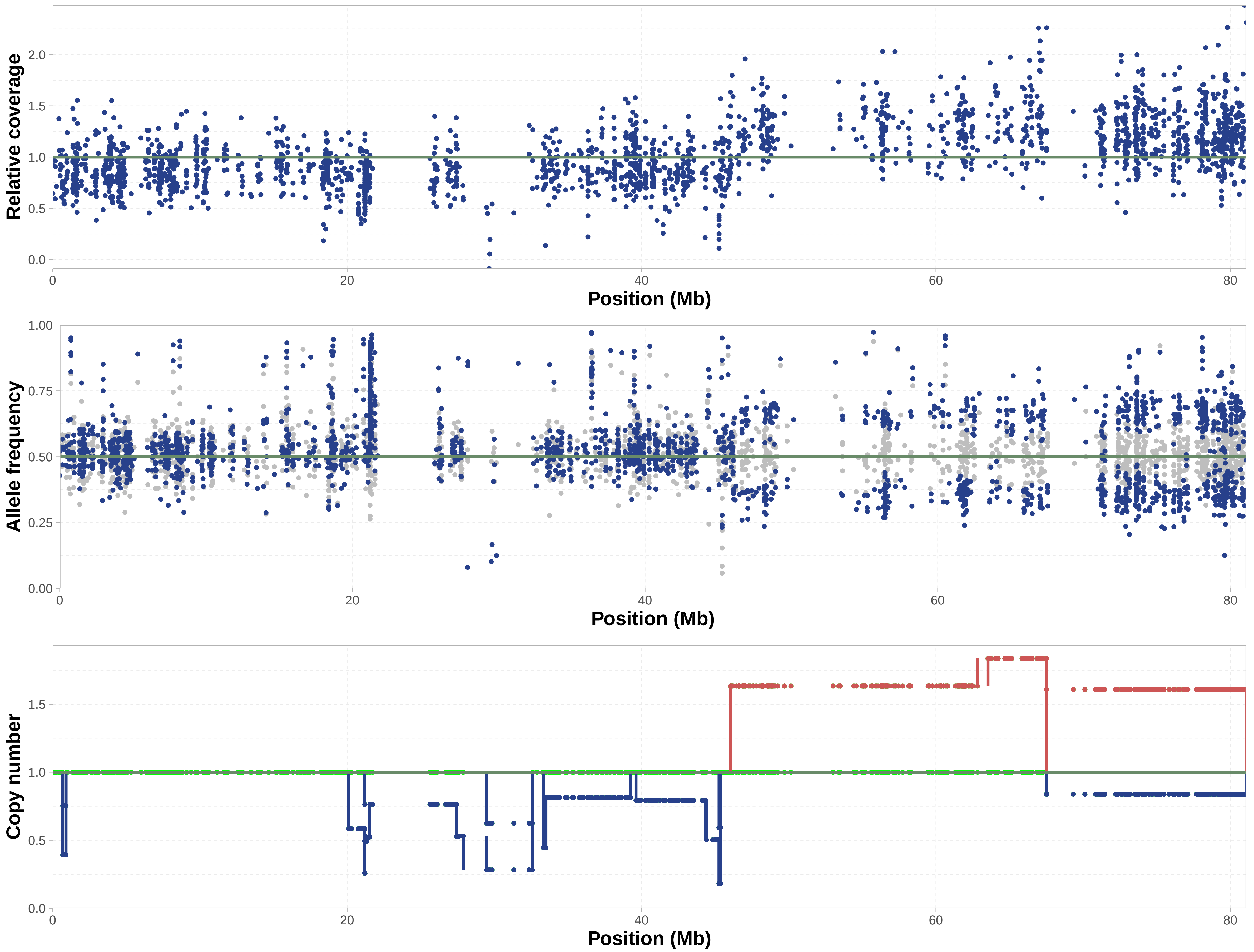

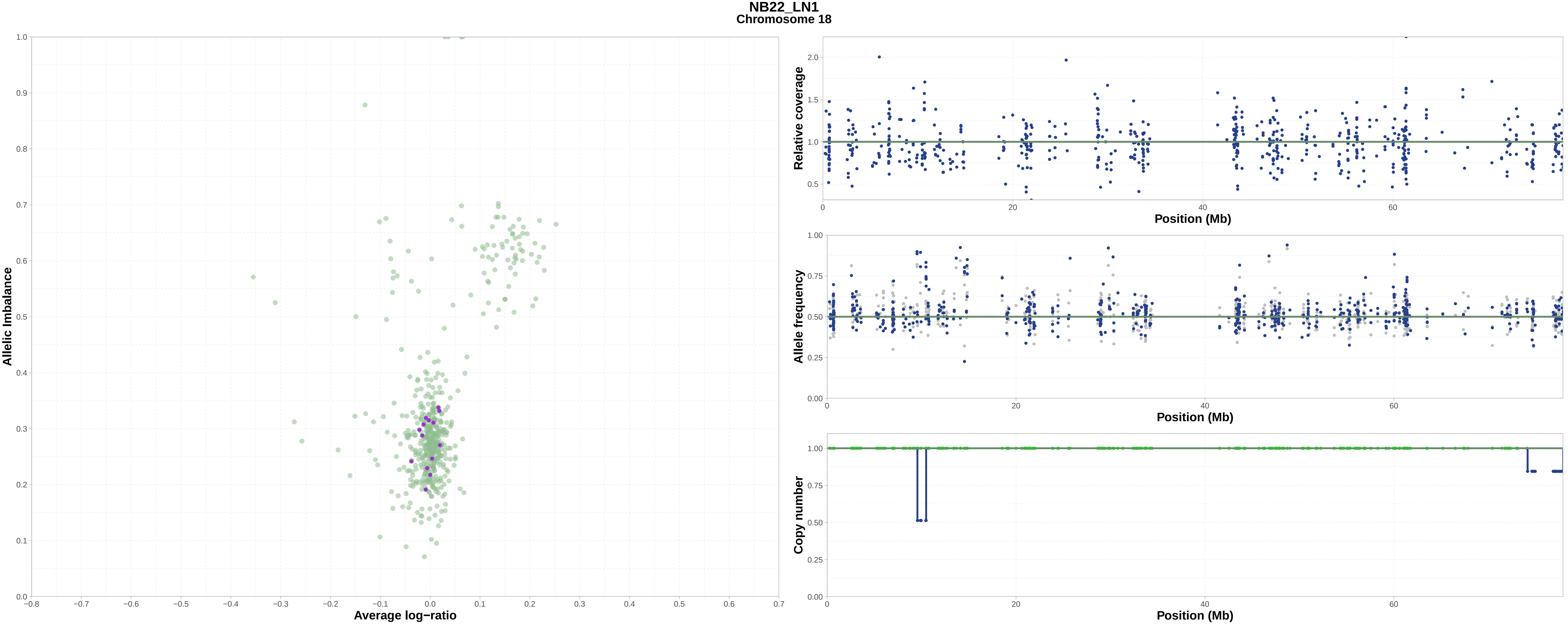

NB22_LN1
Chromosome 18
Allelic Imbalance
Average log−ratio
Relative coverage
Position (Mb)
Allele frequency
Position (Mb)
Copy number
Position (Mb)

NB22_LN1
Chromosome 19

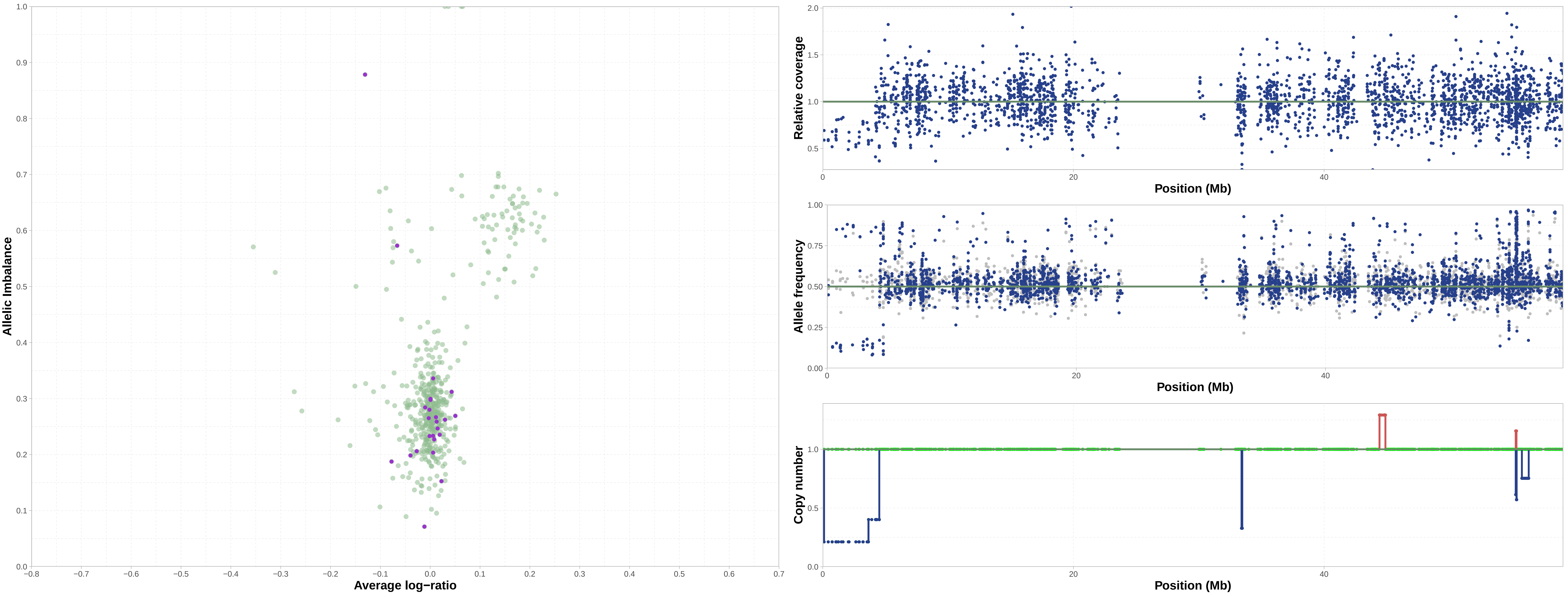

NB22_LN1
Chromosome 20

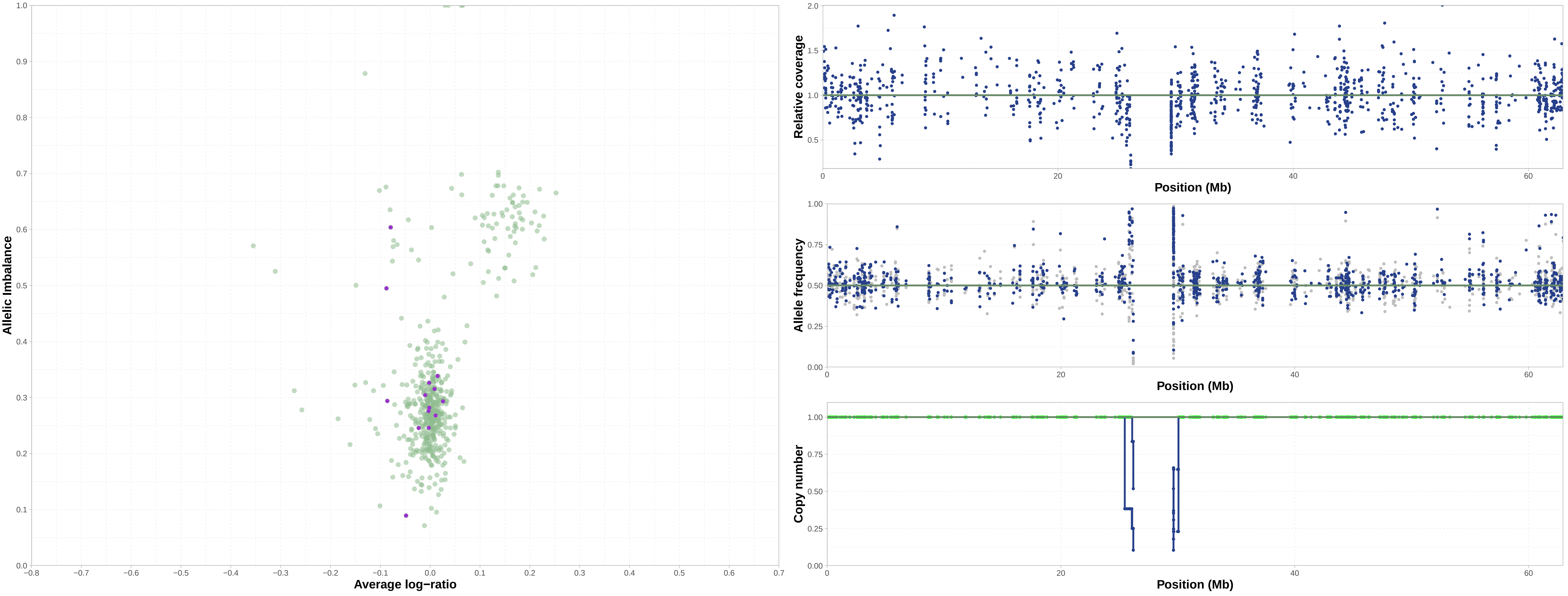

NB22_LN1
Chromosome 21

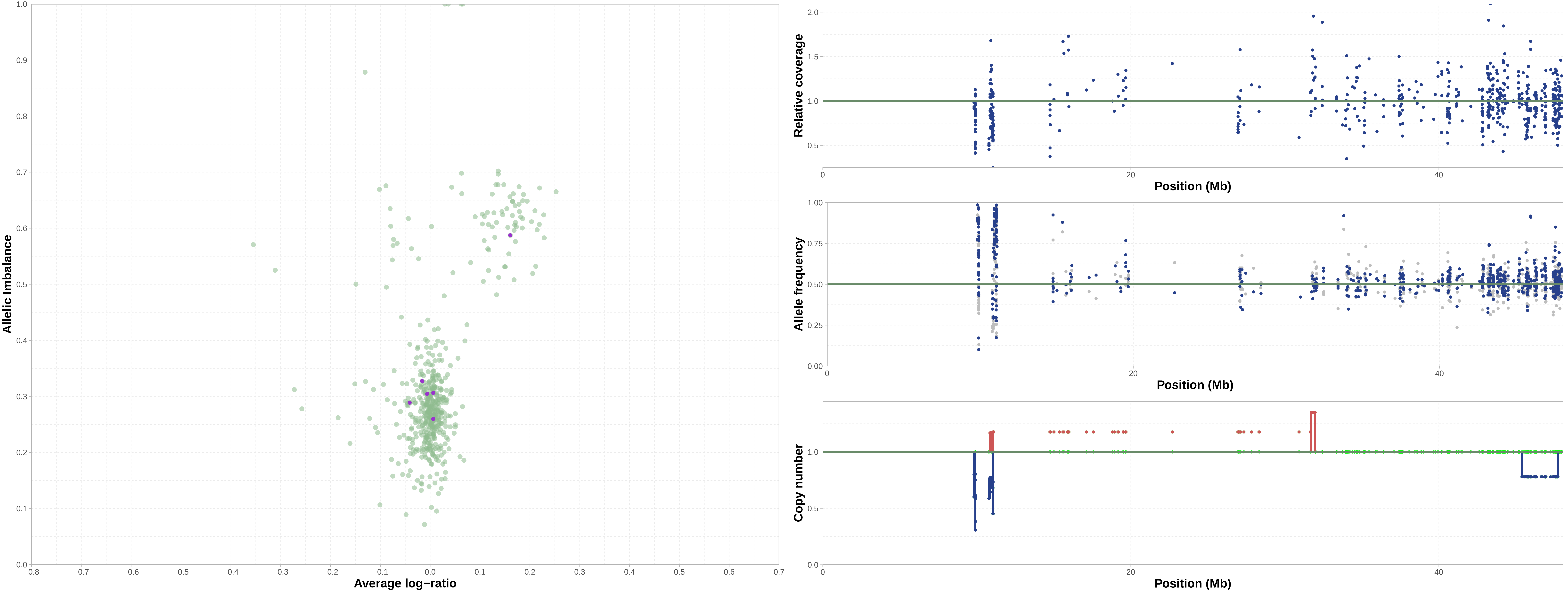

NB22_LN1
Chromosome 22

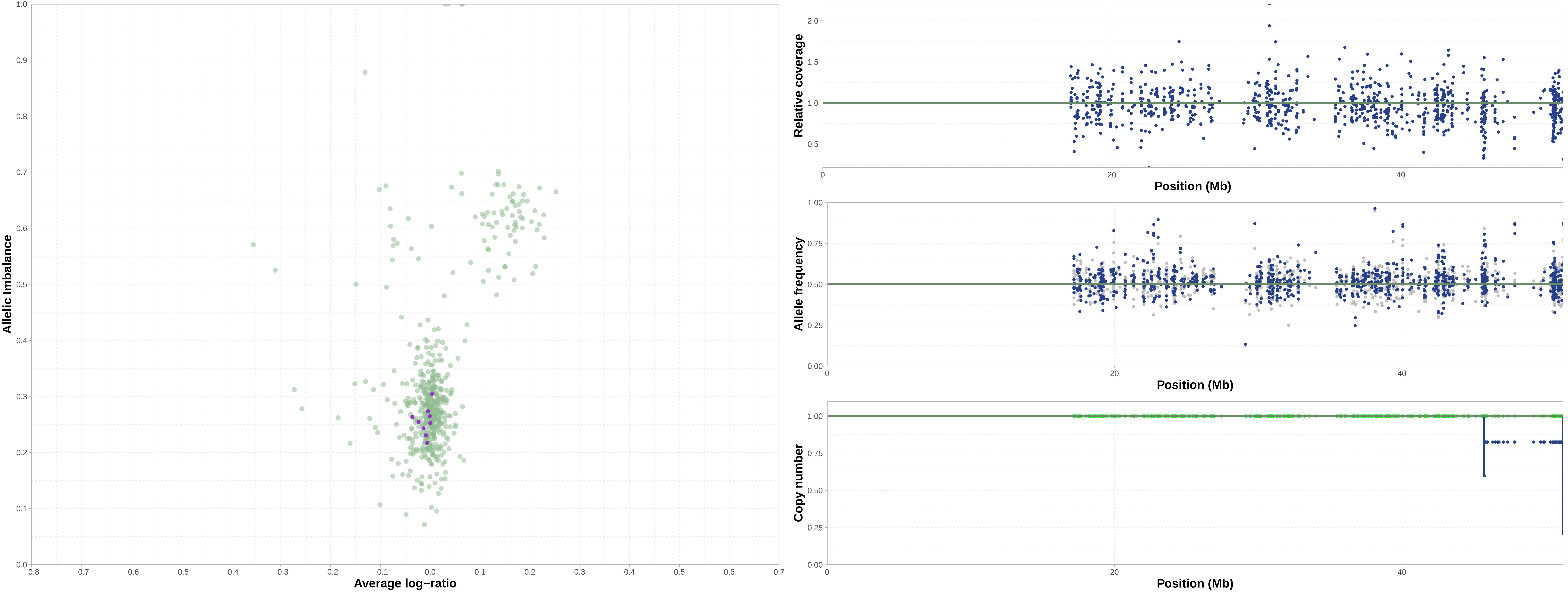

NB22_LN2
Chromosome 1

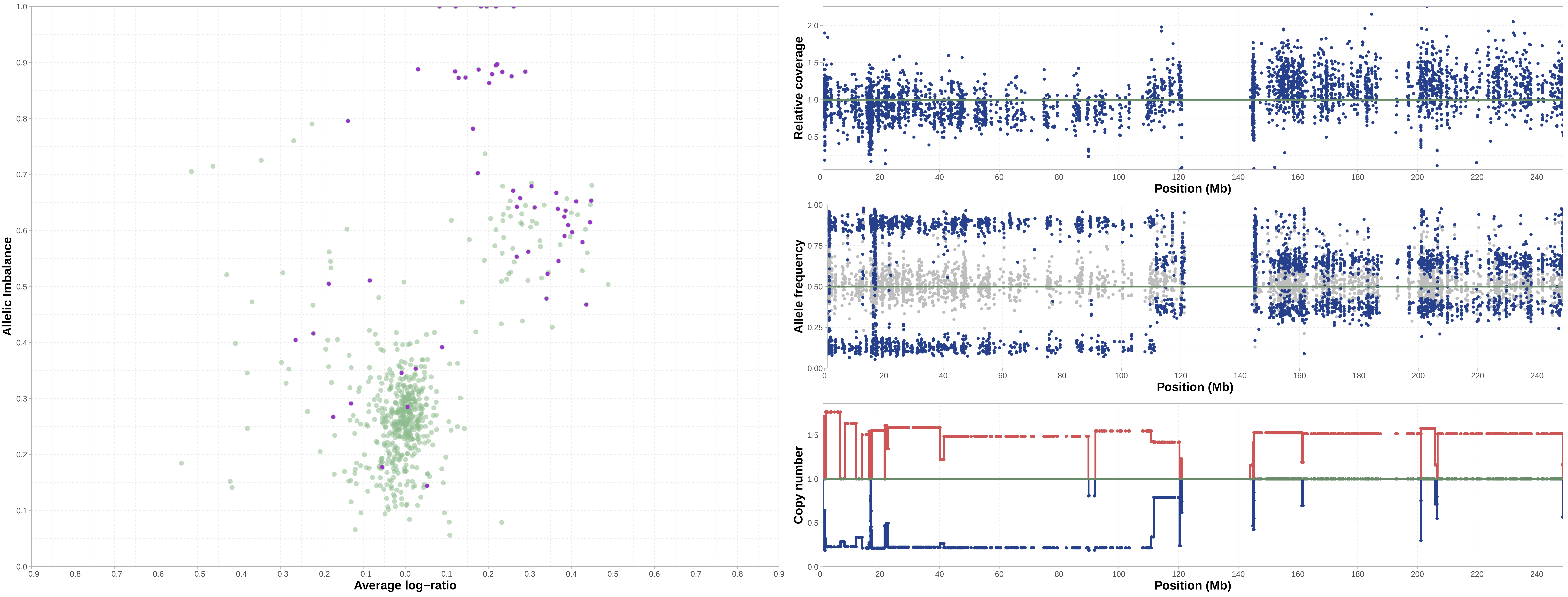

NB22_LN2
Chromosome 2

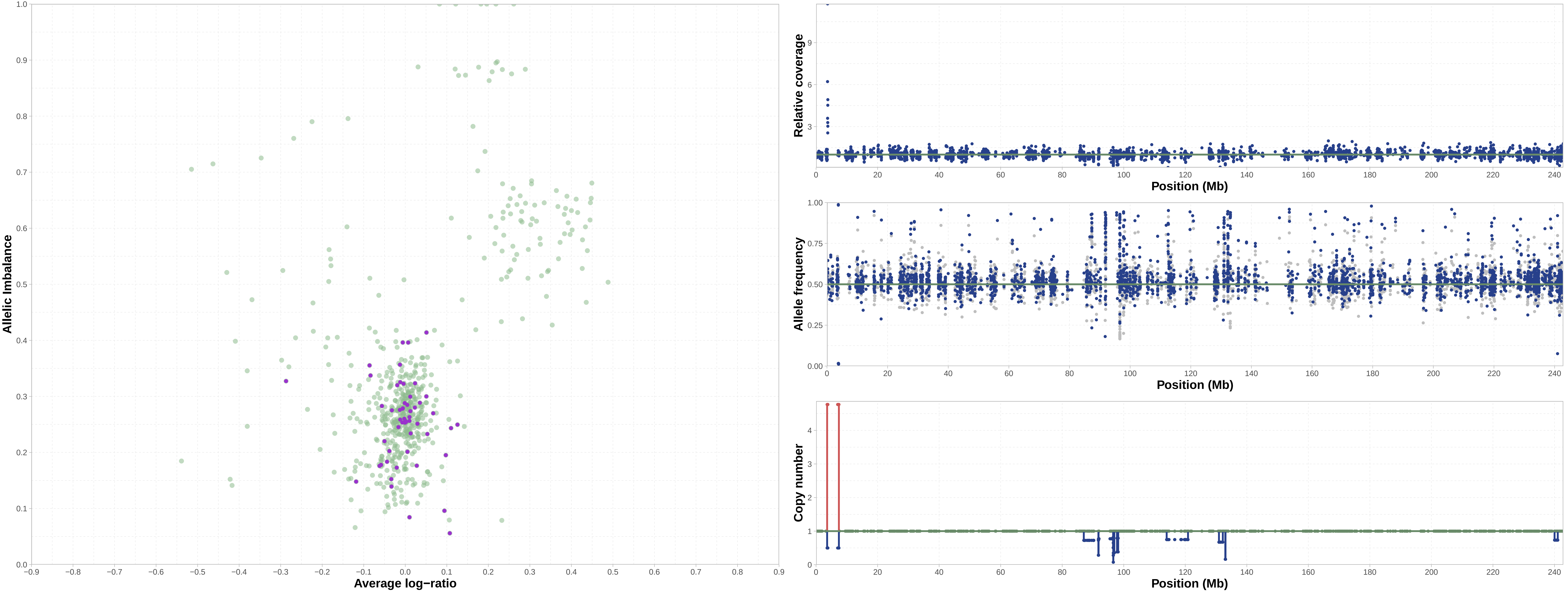

NB22_LN2
Chromosome 3

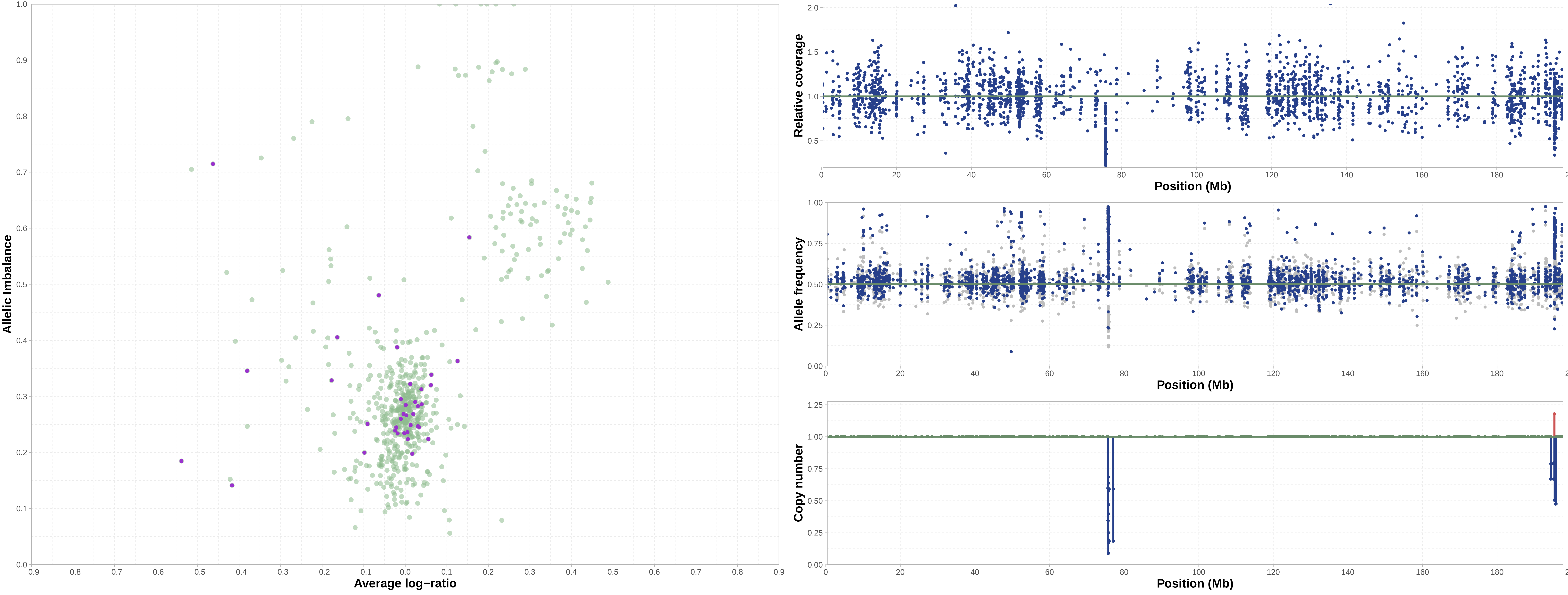

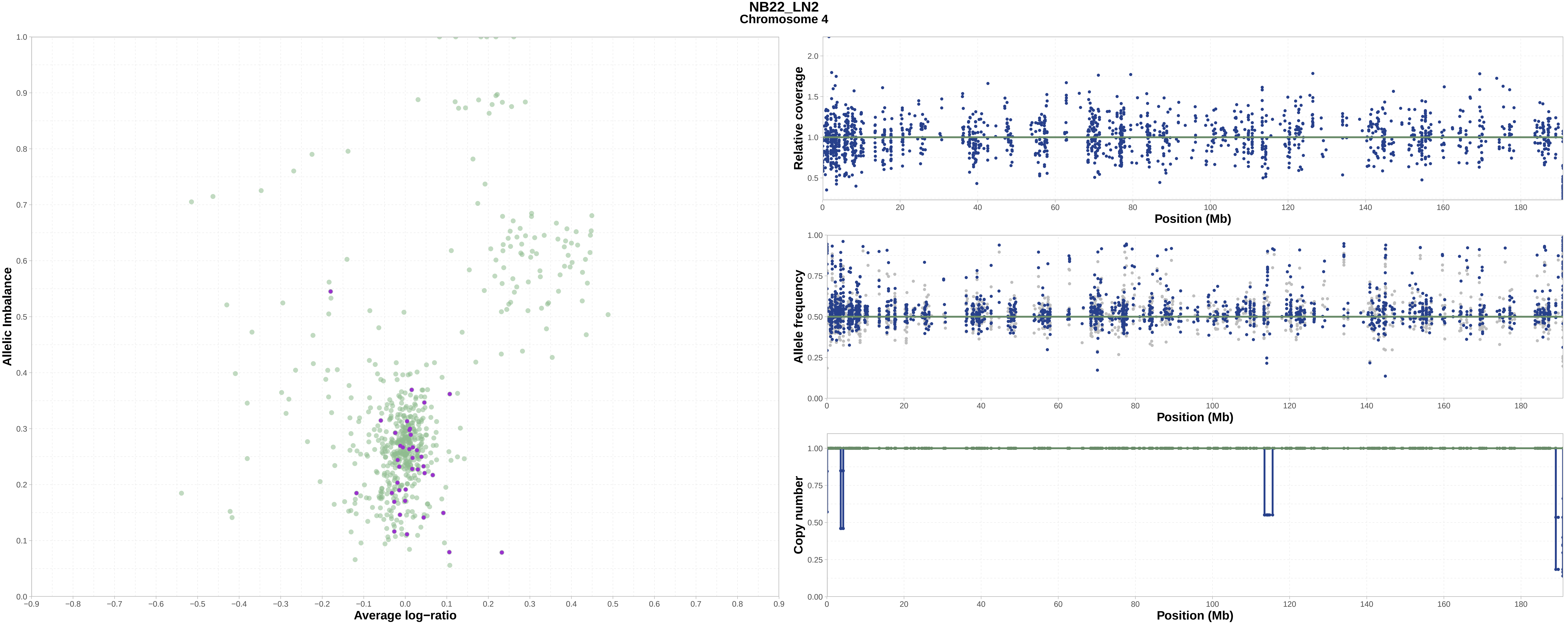

NB22_LN2
Chromosome 4
Allelic Imbalance
Average log−ratio
Relative coverage
Position (Mb)
Allele frequency
Position (Mb)
Copy number
Position (Mb)

NB22_LN2
Chromosome 5

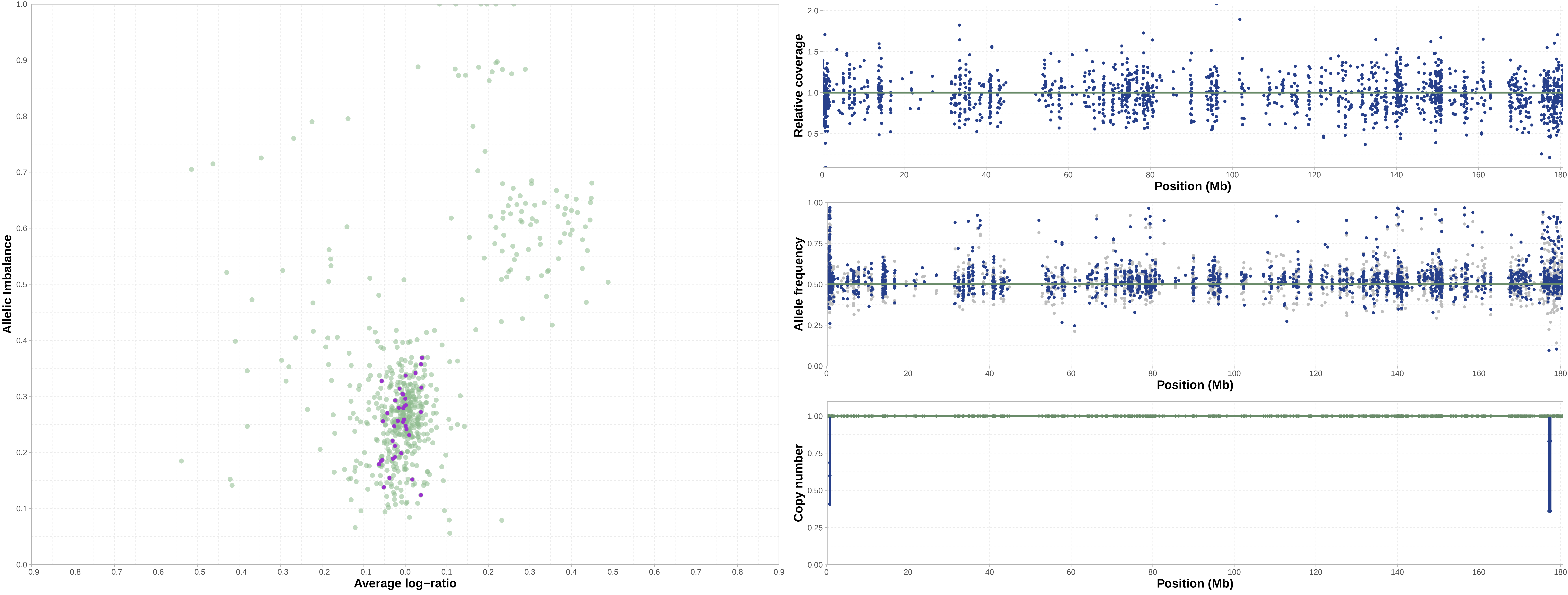

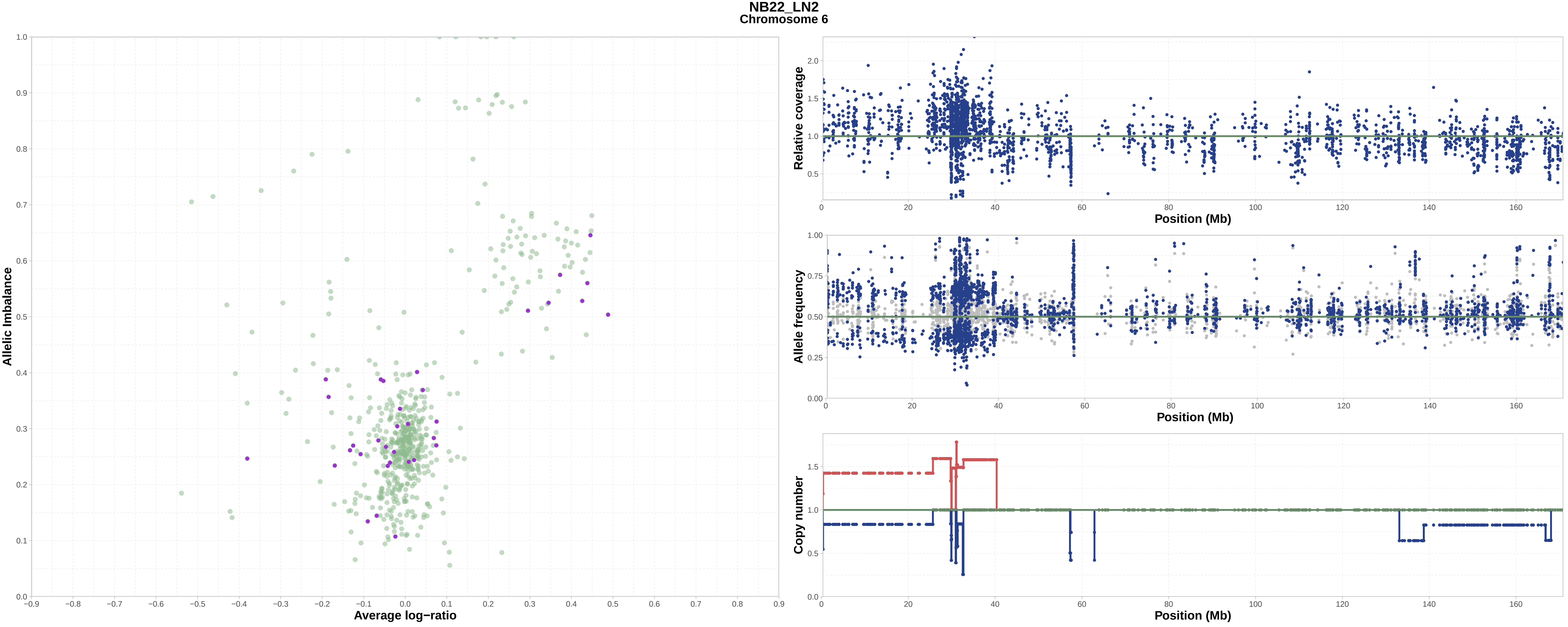
NB22_LN2
Chromosome 6
Allelic Imbalance
Average log−ratio
1.0
0.9
0.8
0.7
0.6
0.5
0.4
0.3
0.2
0.1
0.0
−0.9
−0.8
−0.7
−0.6
−0.5
−0.4
−0.3
−0.2
−0.1
0.0
0.1
0.2
0.3
0.4
0.5
0.6
0.7
0.8
0.9
Relative coverage
2.0
1.5
1.0
0.5
Position (Mb)
0
20
40
60
80
100
120
140
160
Allele frequency
1.00
0.75
0.50
0.25
0.00
Position (Mb)
Copy number
1.5
1.0
0.5
0.0
Position (Mb)

NB22_LN2
Chromosome 7

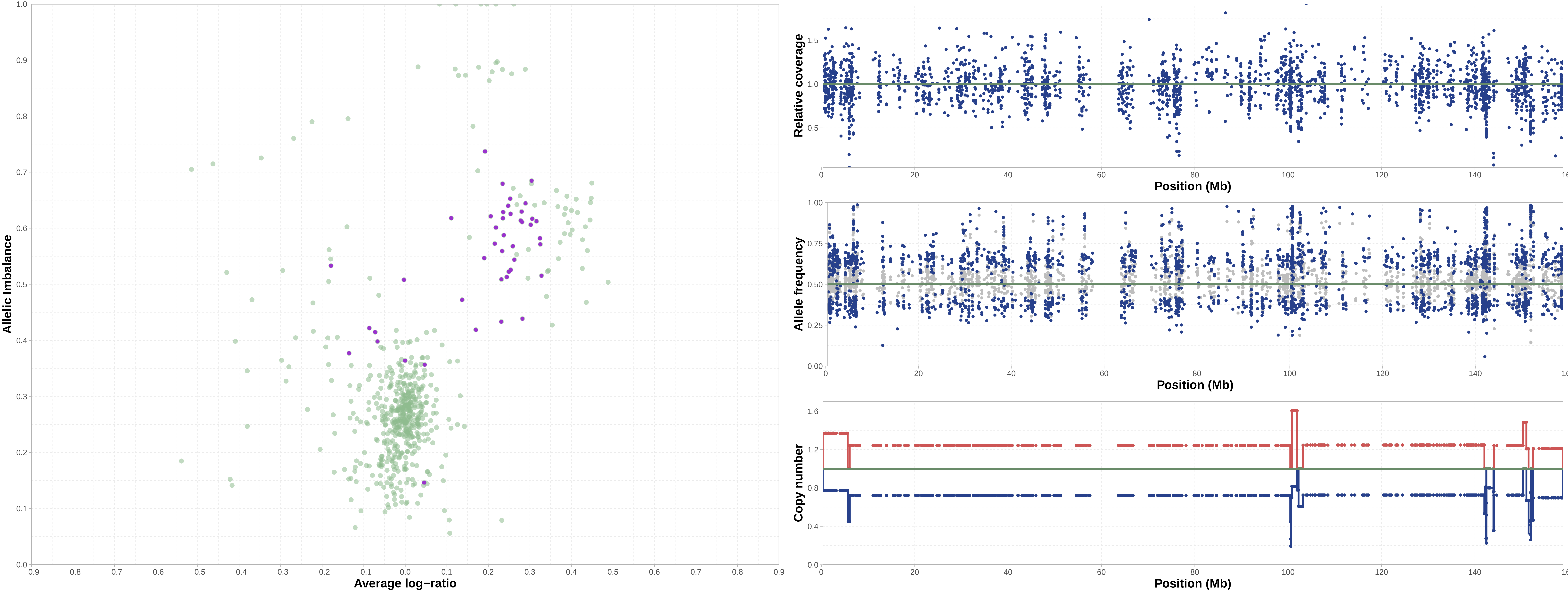

NB22_LN2
Chromosome 8

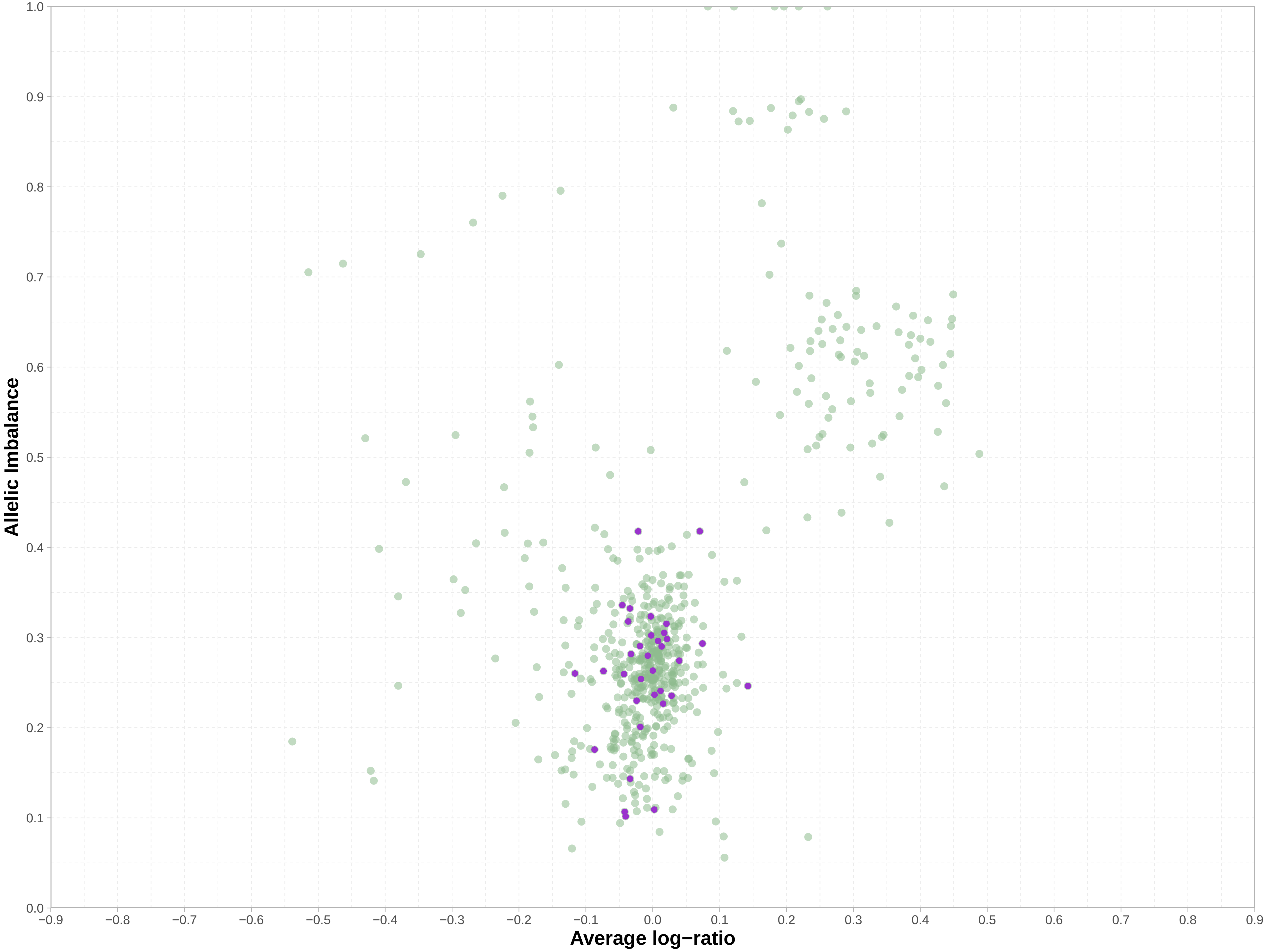

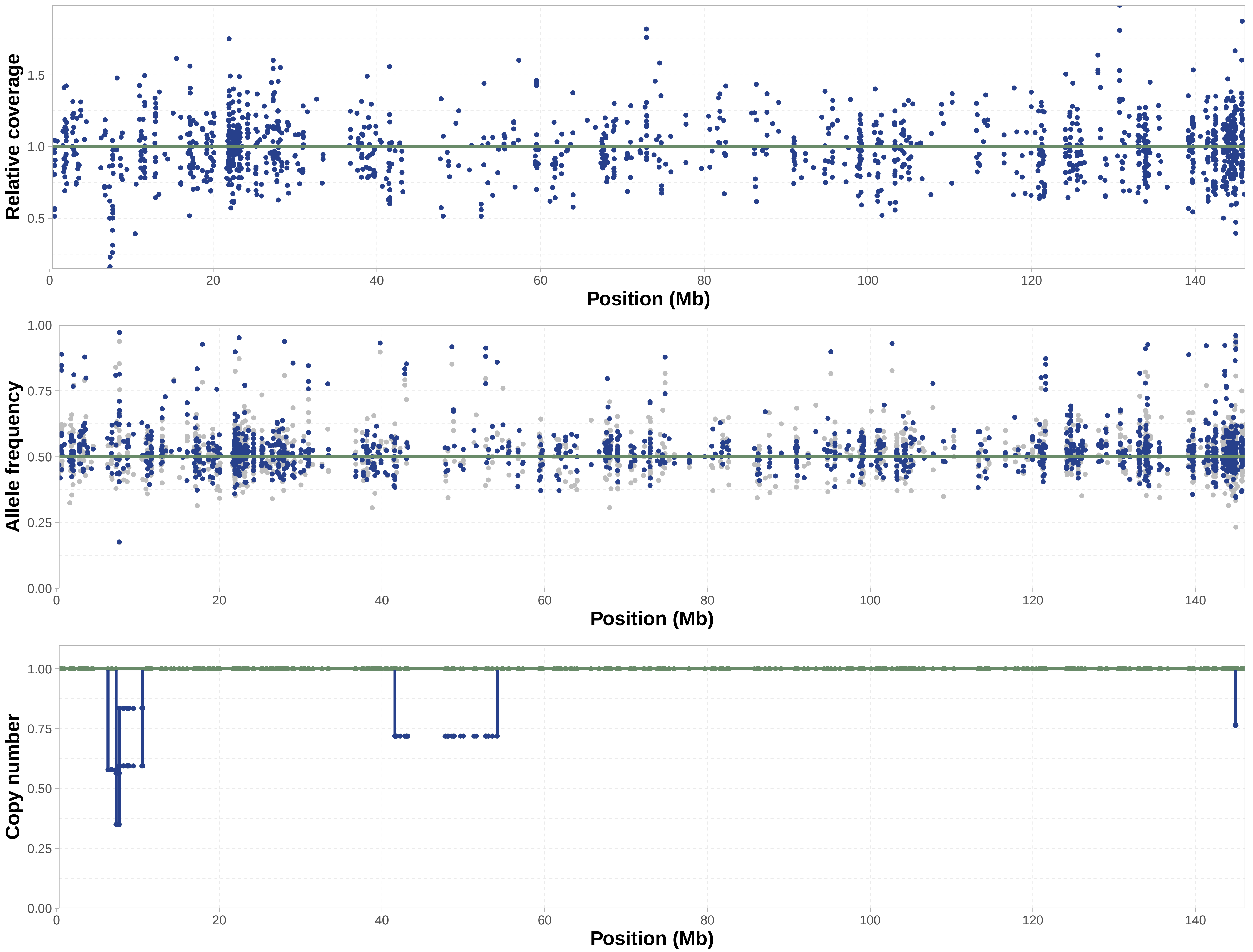

NB22_LN2
Chromosome 9

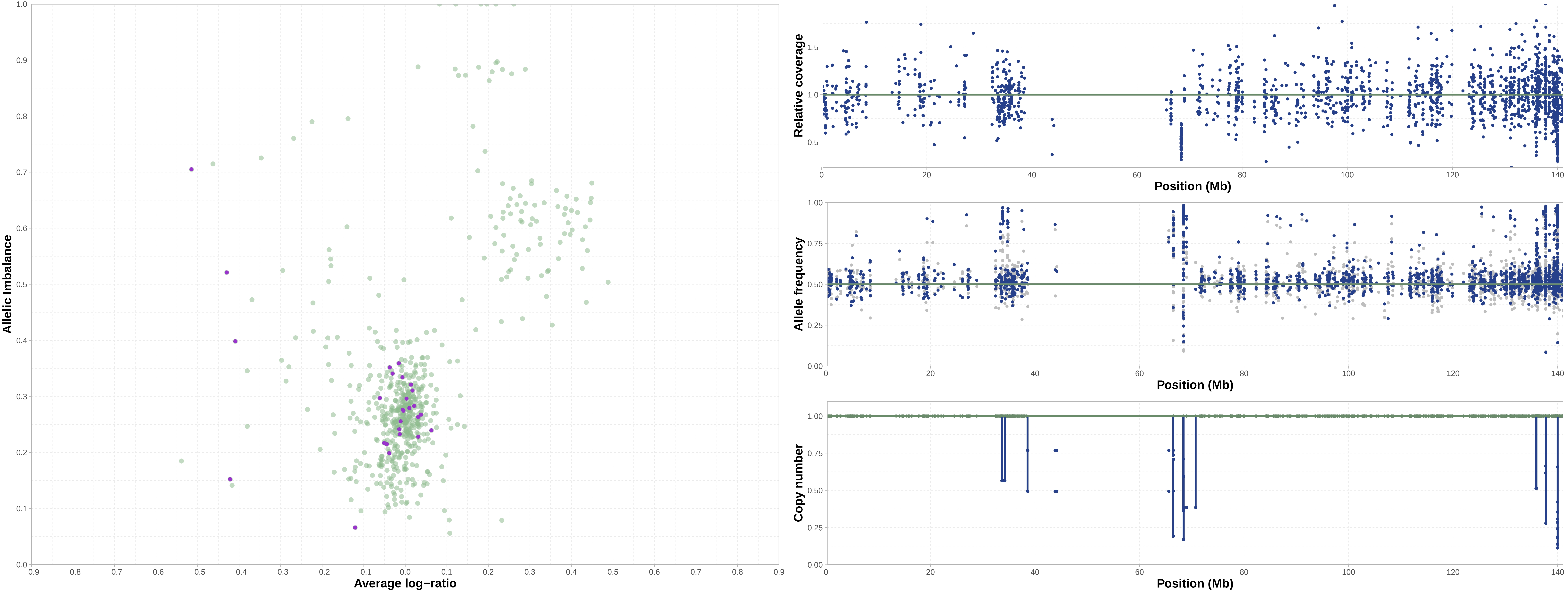

NB22_LN2
Chromosome 10

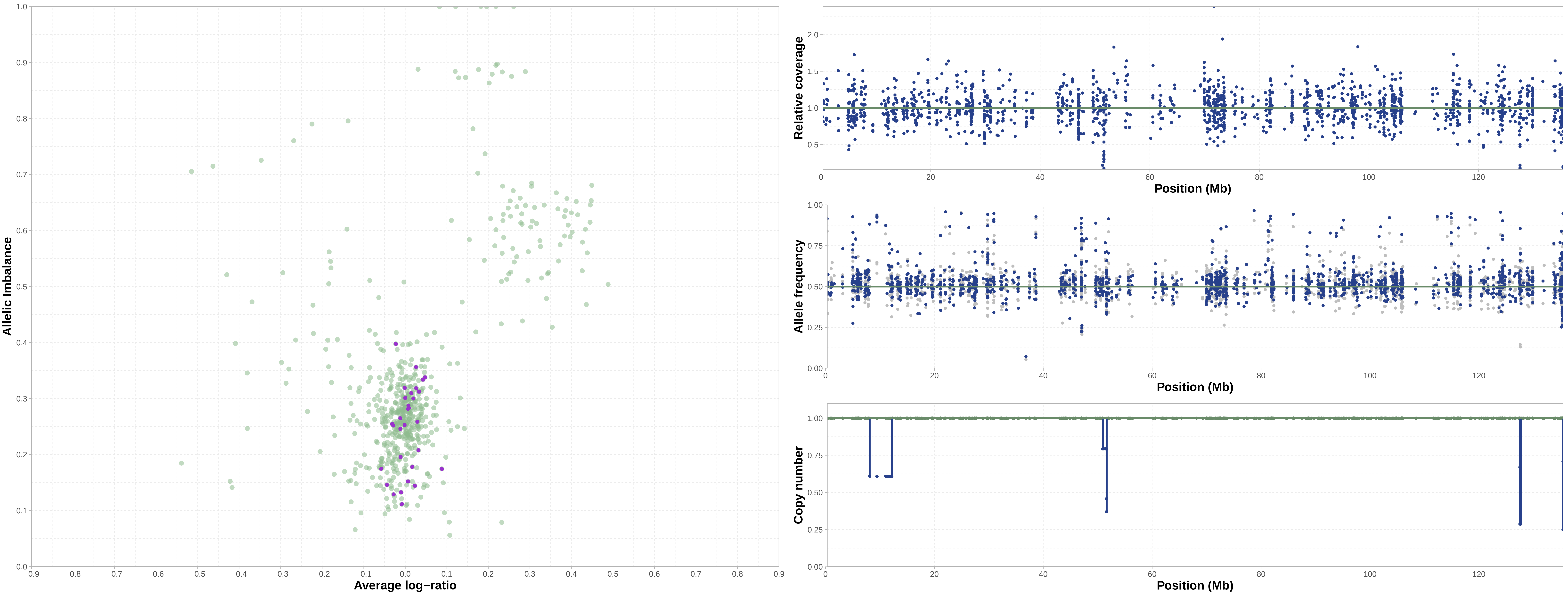

NB22_LN2
Chromosome 11

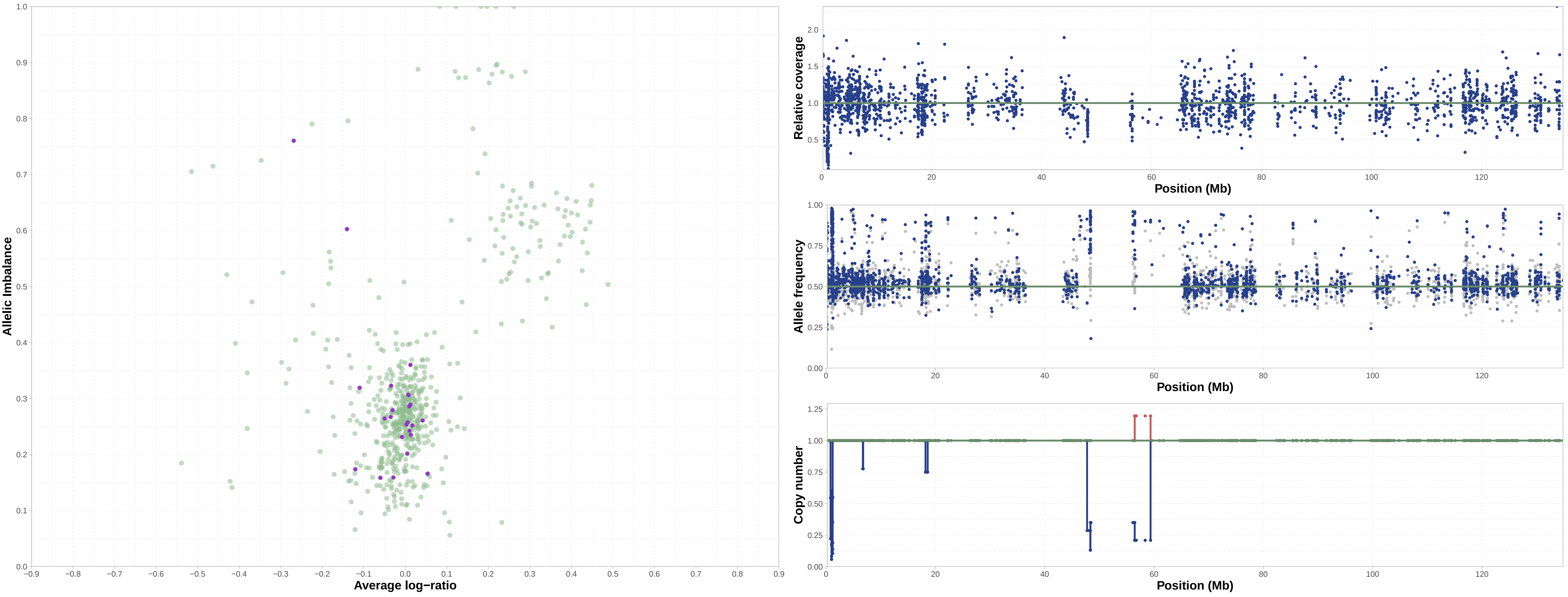

NB22_LN2
Chromosome 12

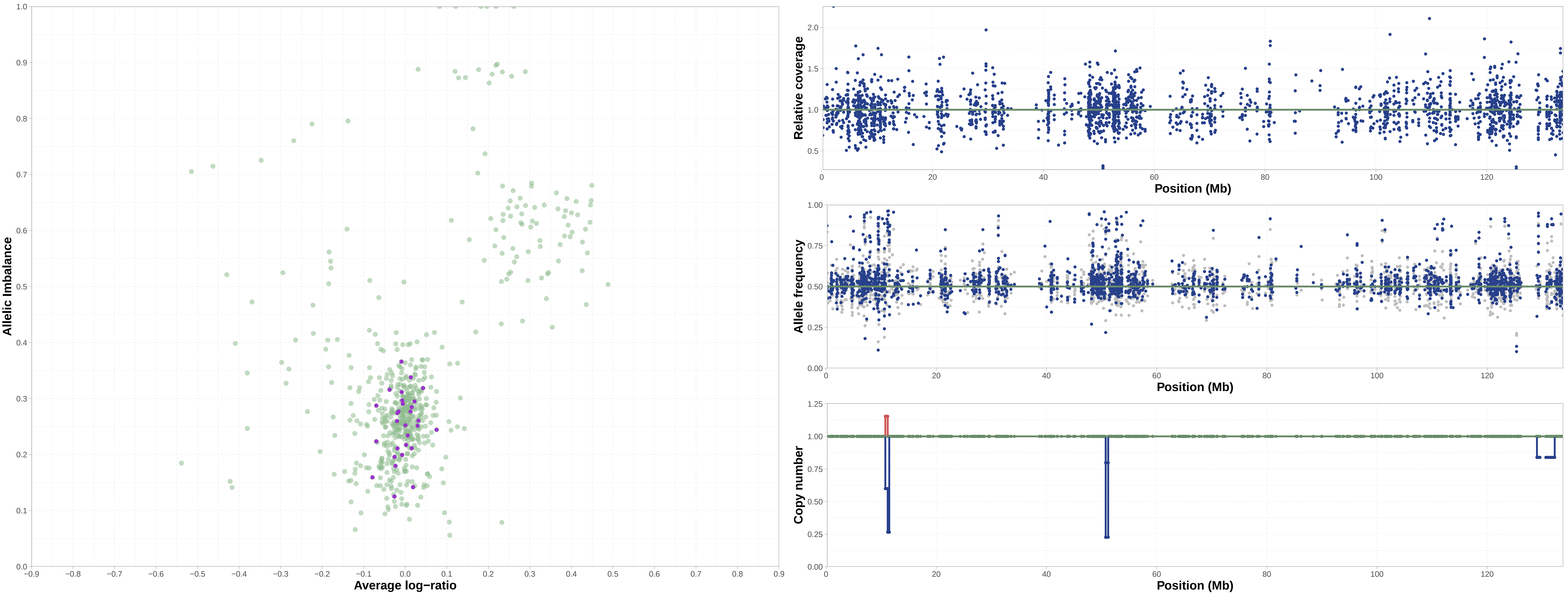

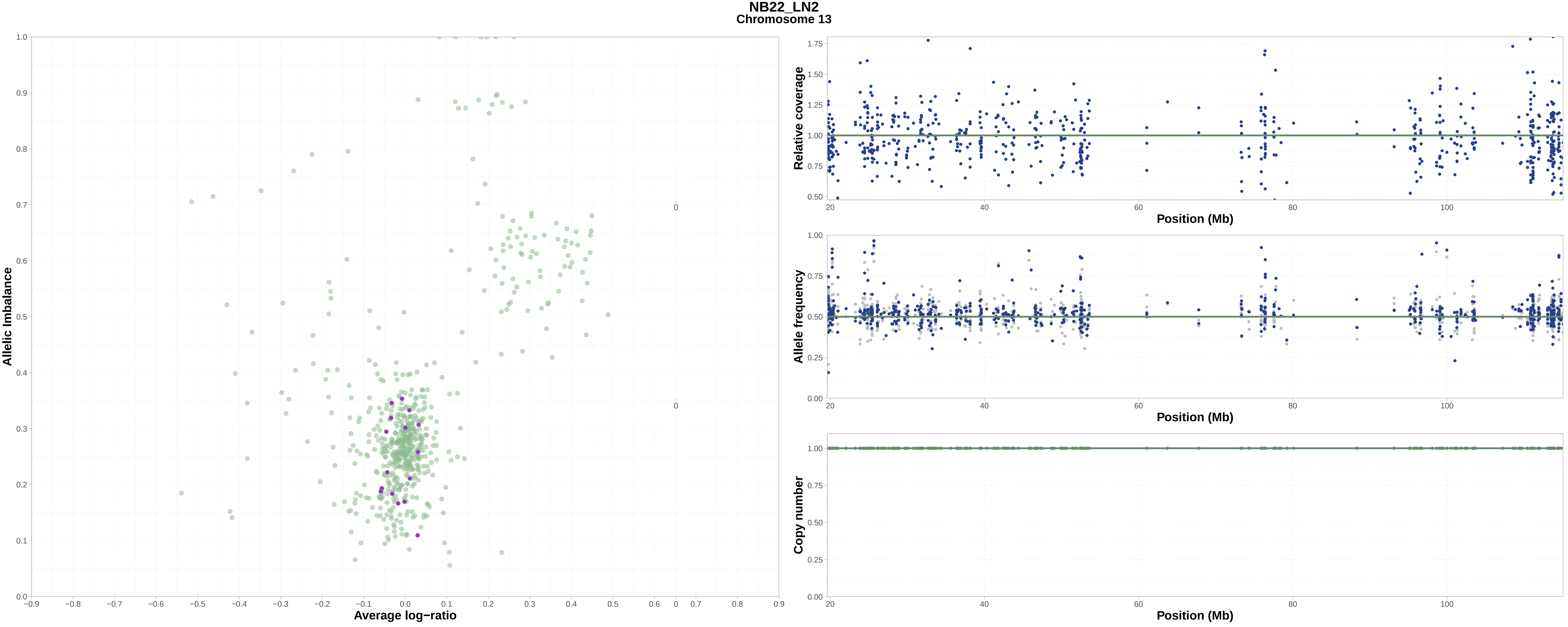

NB22_LN2
Chromosome 13
Allelic Imbalance
Average log−ratio
Relative coverage
Position (Mb)
Allele frequency
Position (Mb)
Copy number
Position (Mb)

NB22_LN2
Chromosome 14

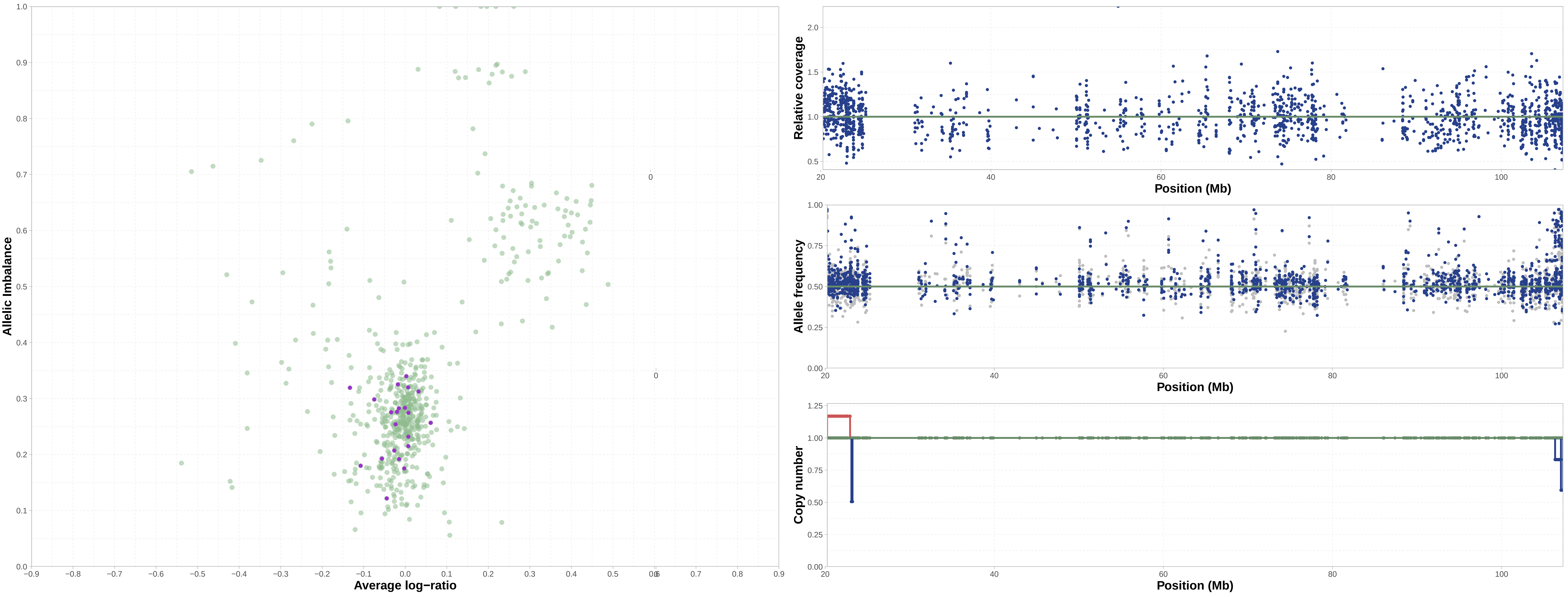

NB22_LN2
Chromosome 15

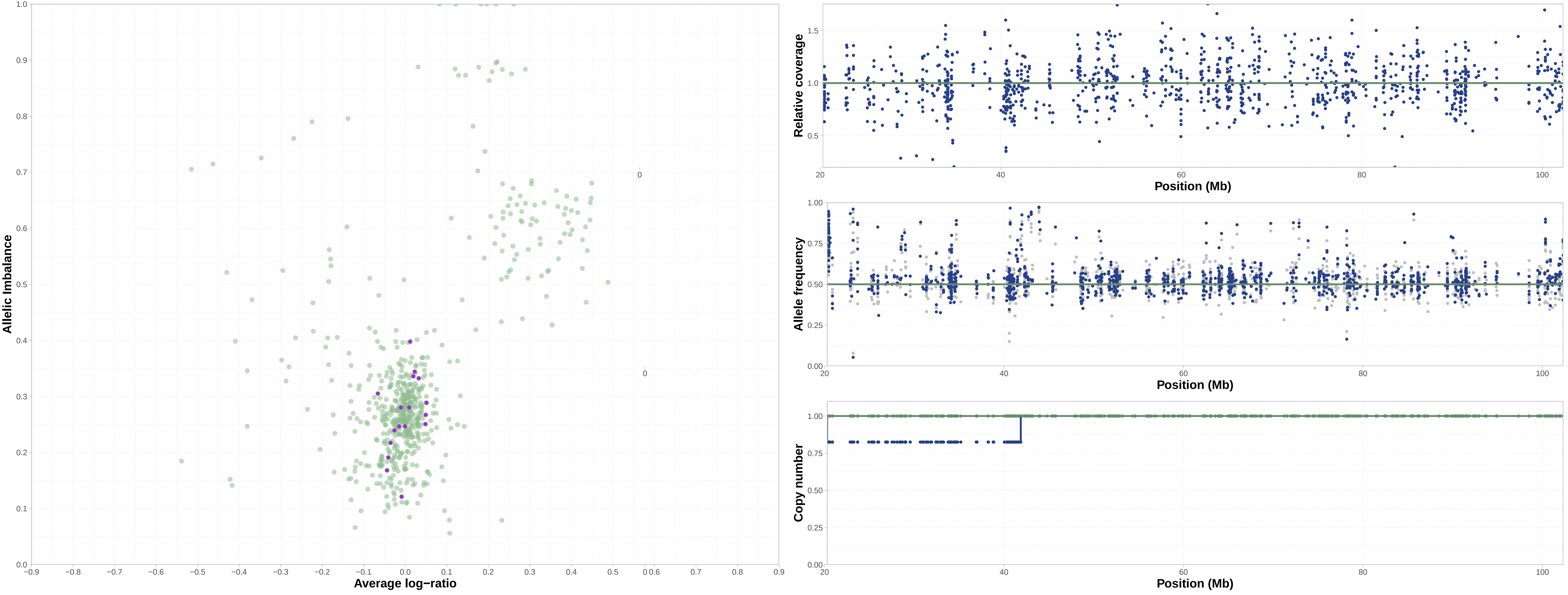

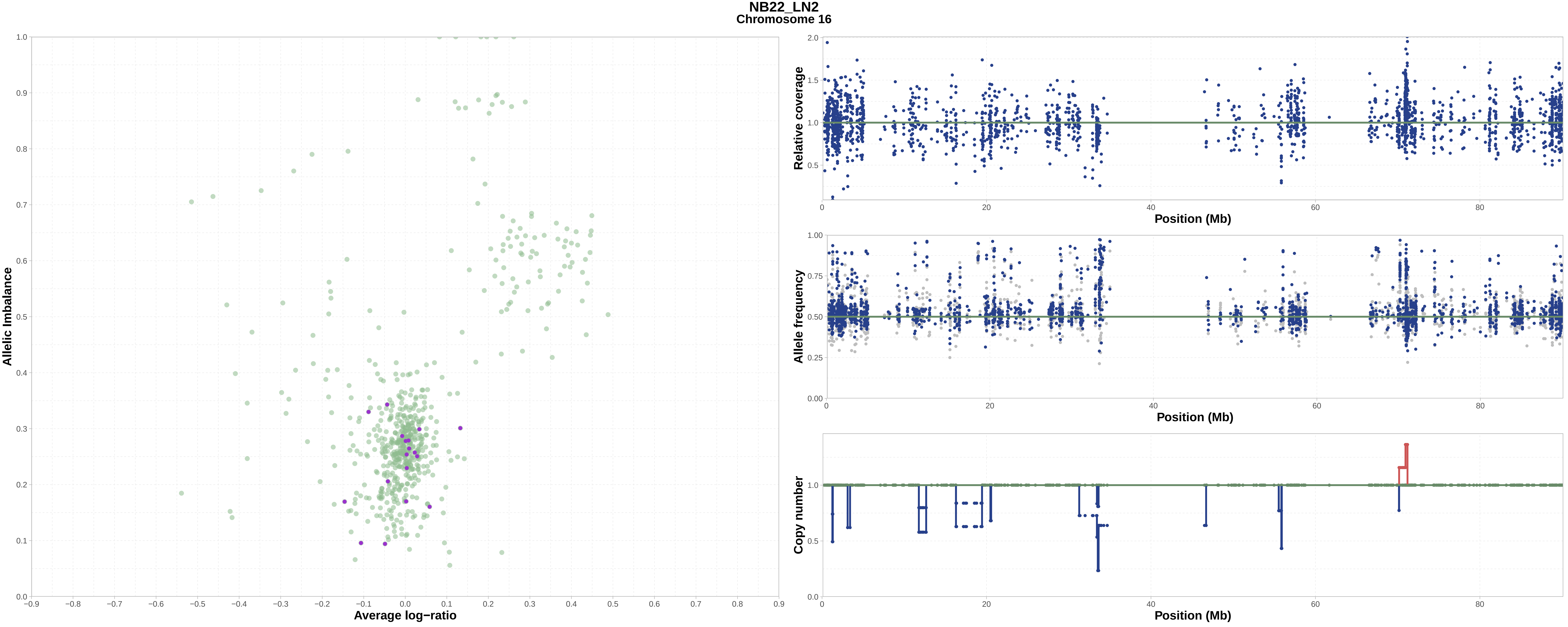
NB22_LN2
Chromosome 16
Allelic Imbalance
Average log−ratio
Relative coverage
Position (Mb)
Allele frequency
Position (Mb)
Copy number
Position (Mb)

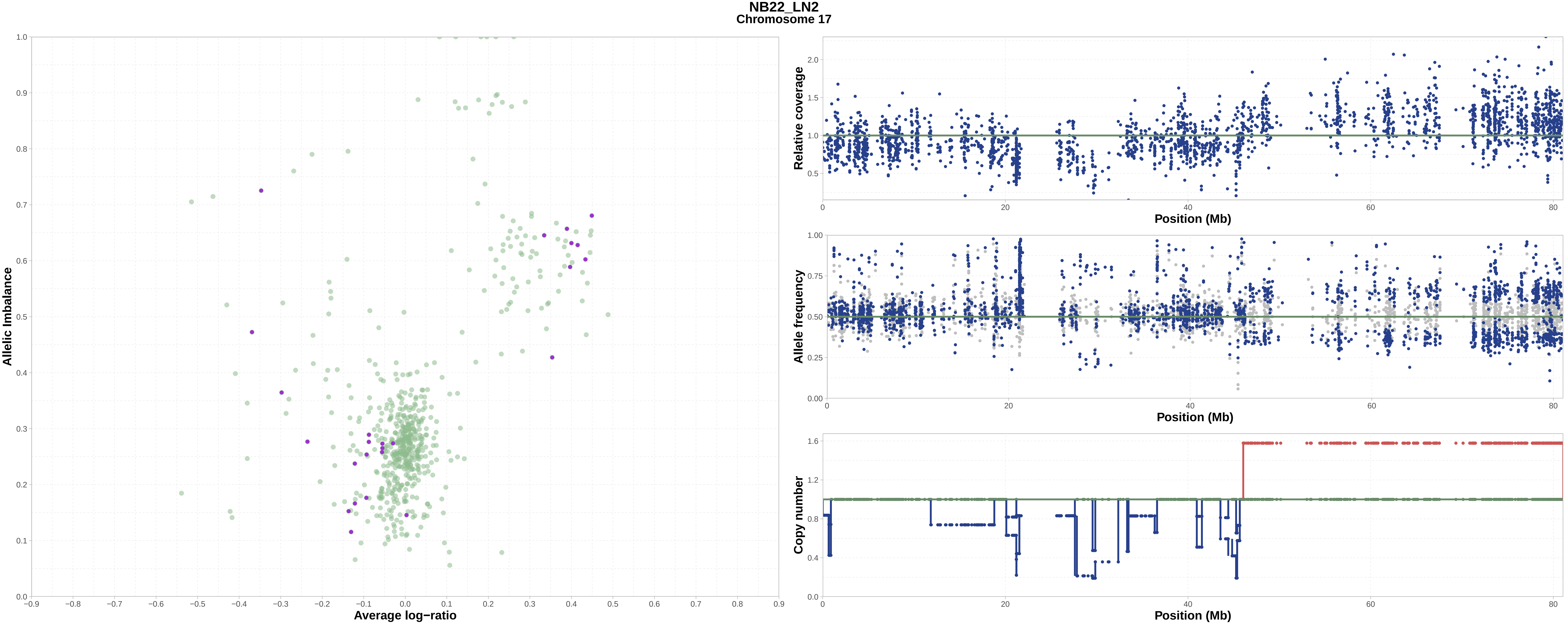
NB22_LN2
Chromosome 17
Allelic Imbalance
Average log−ratio
Relative coverage
Position (Mb)
Allele frequency
Position (Mb)
Copy number
Position (Mb)

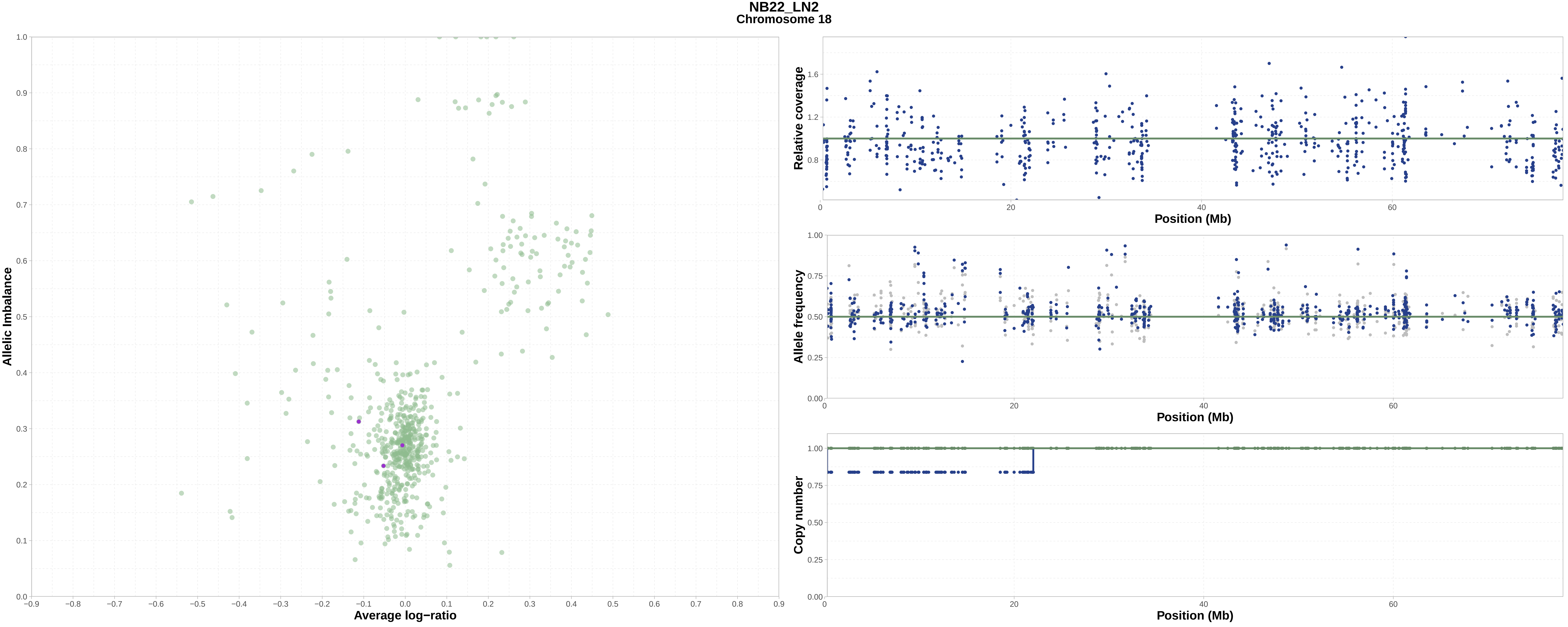

NB22_LN2
Chromosome 18
Allelic Imbalance
Average log−ratio
Relative coverage
Position (Mb)
Allele frequency
Position (Mb)
Copy number
Position (Mb)

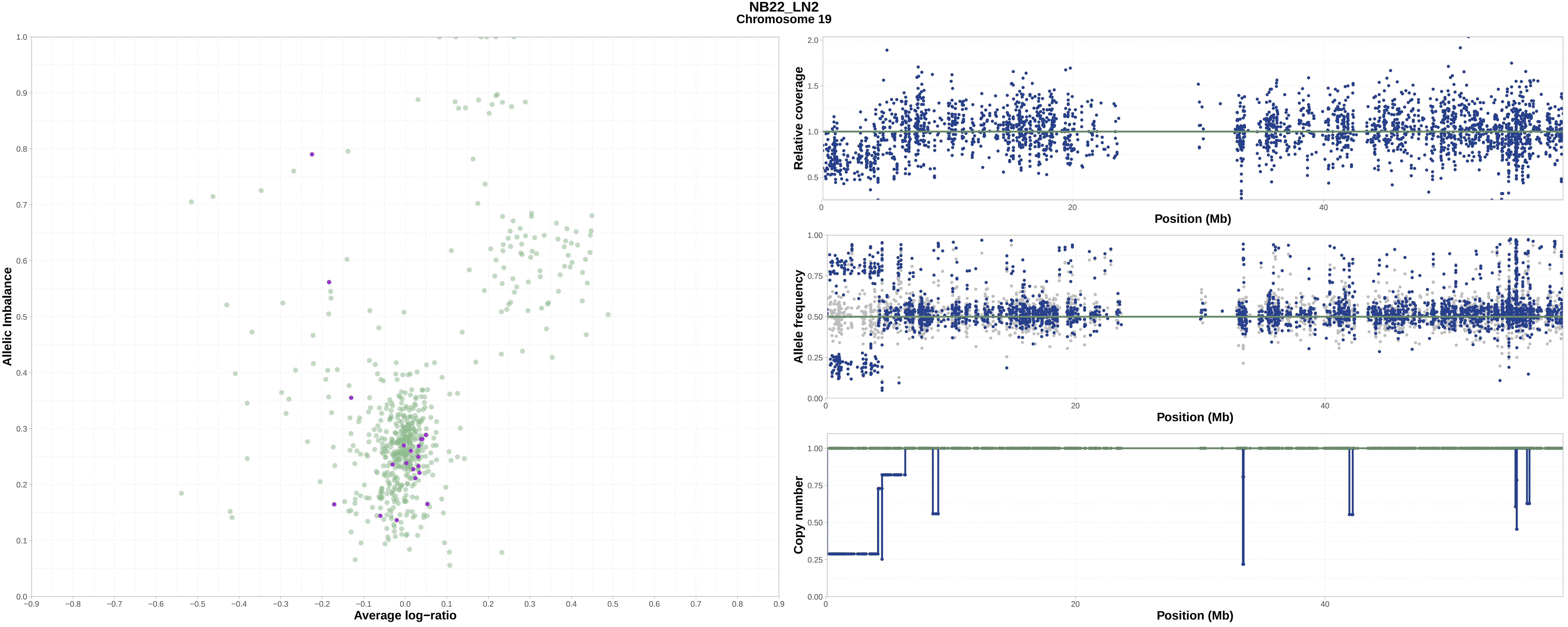
NB22_LN2
Chromosome 19
Allelic Imbalance
Average log−ratio
Relative coverage
Position (Mb)
Allele frequency
Position (Mb)
Copy number
Position (Mb)

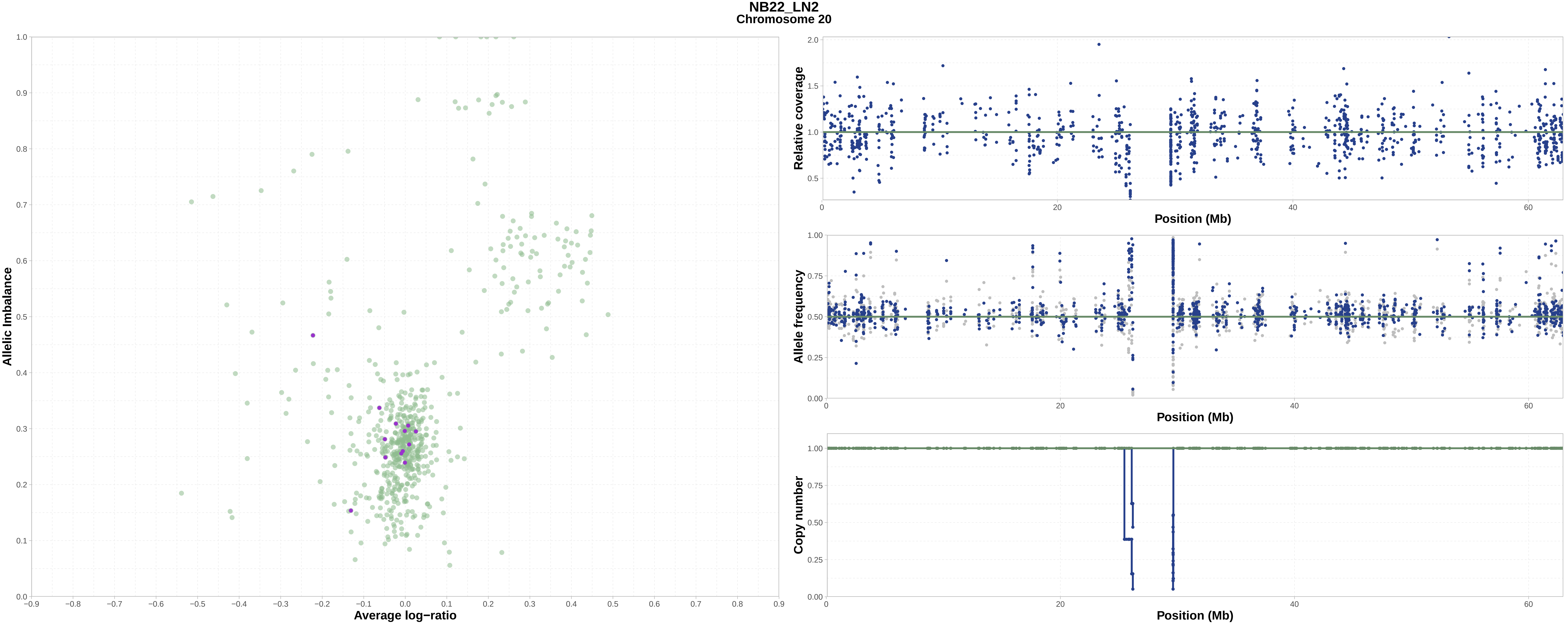

NB22_LN2
Chromosome 20
Allelic Imbalance
Average log−ratio
Relative coverage
Position (Mb)
Allele frequency
Position (Mb)
Copy number
Position (Mb)

NB22_LN2
Chromosome 21

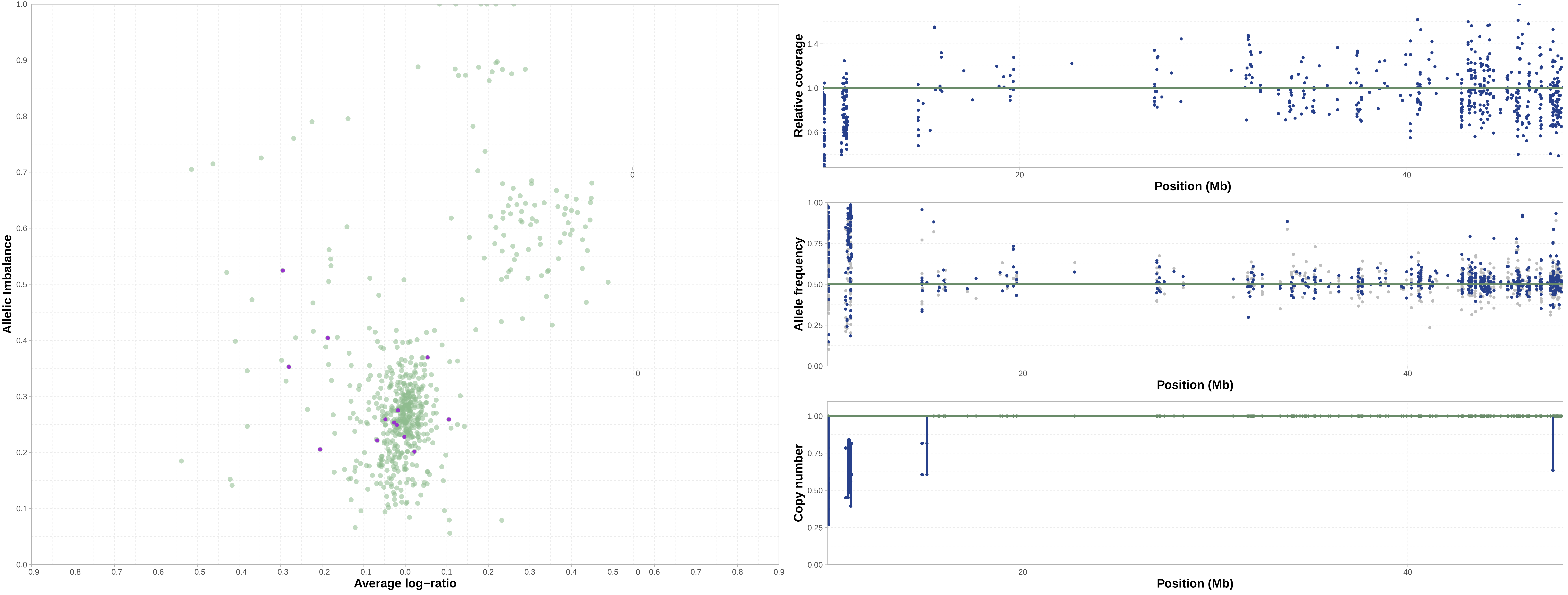

NB22_LN2
Chromosome 22

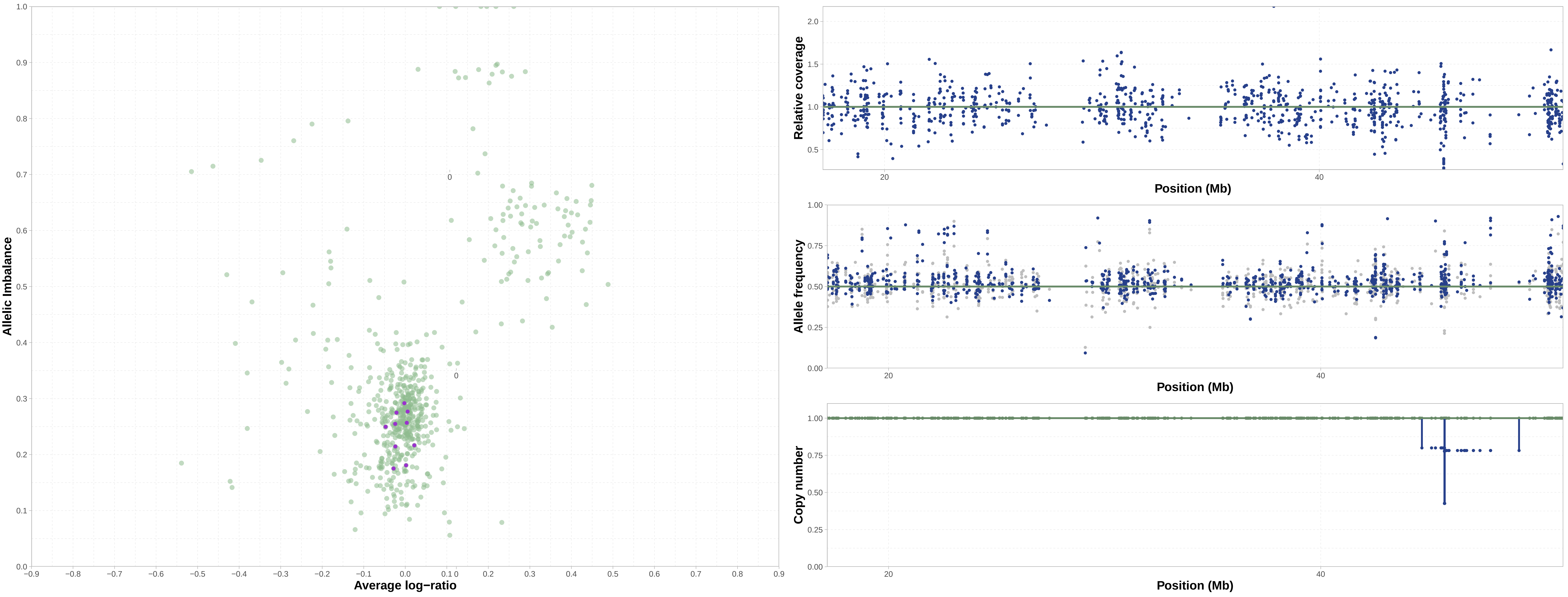

Supplement: Supplementary_Figure_5_bbab292 [file supplementary_figure_5_bbab292.pdf]
