# Supplementary material for: Tracing the evolution of aneuploid cancers by multiregional sequencing with CRUST
Source: Brief Bioinform. 2021 Aug 3;22(6):bbab292. doi: 10.1093/bib/bbab292 (PMC8981300; doi:10.1093/bib/bbab292)
Supplement: Supplementary_Figure_8_bbab292 [file supplementary_figure_8_bbab292.pdf]

WES_CRUK0001 2
WES_CRUK0002 13
WES_CRUK0003 16
WES_CRUK0004 20
WES_CRUK0005 23
WES_CRUK0009 27
WES_CRUK0011 38
WES_CRUK0017 43
WES_CRUK0018 53
WES_CRUK0023 61
WES_CRUK0024 63
WES_CRUK0029 69
WES_CRUK0031 72
WES_CRUK0034 80
WES_CRUK0037 86
WES_CRUK0046 89
WES_CRUK0050 91
WES_CRUK0051 94
WES_CRUK0052 103
WES_CRUK0094 109

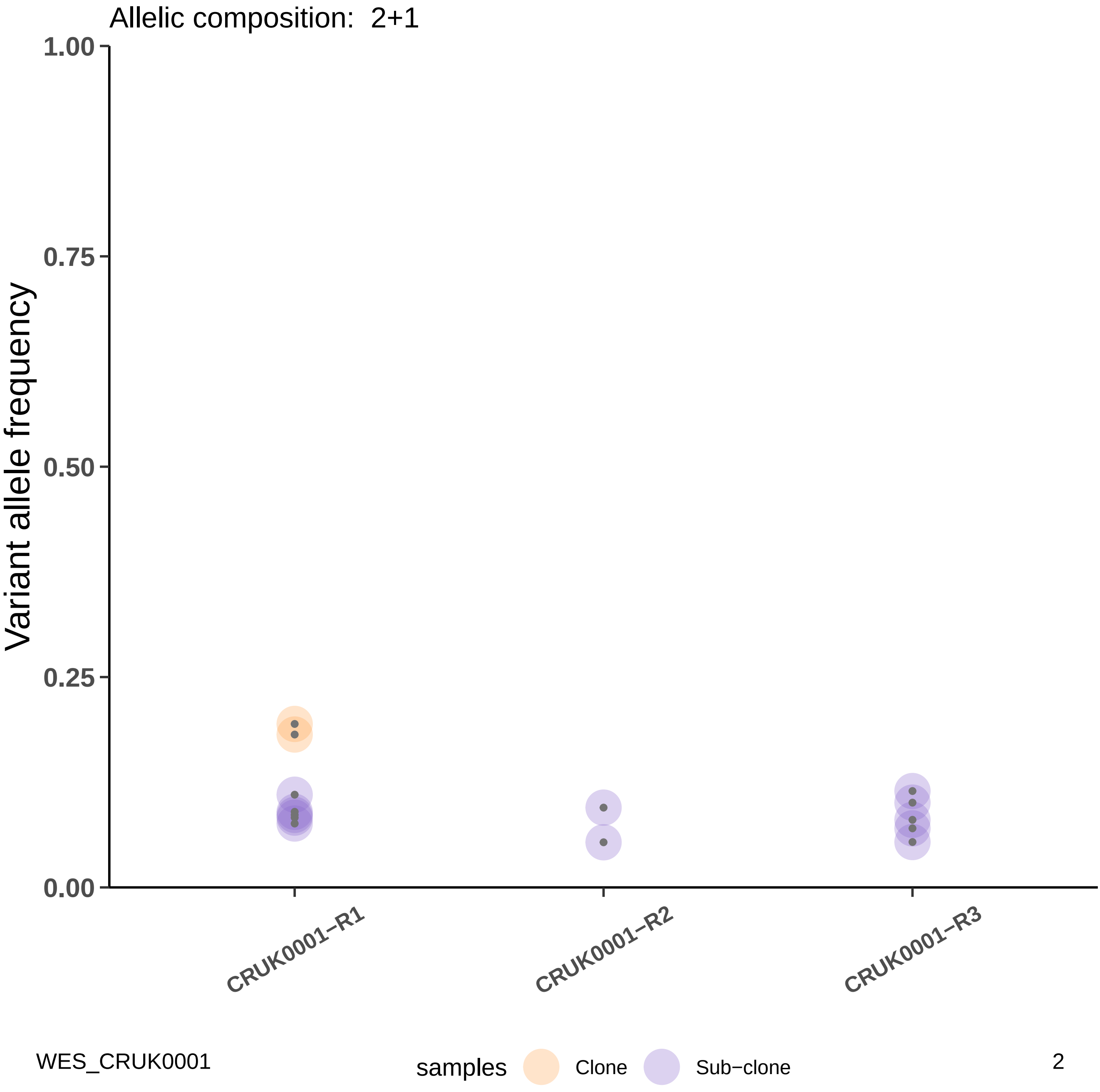

WES_CRUK0001

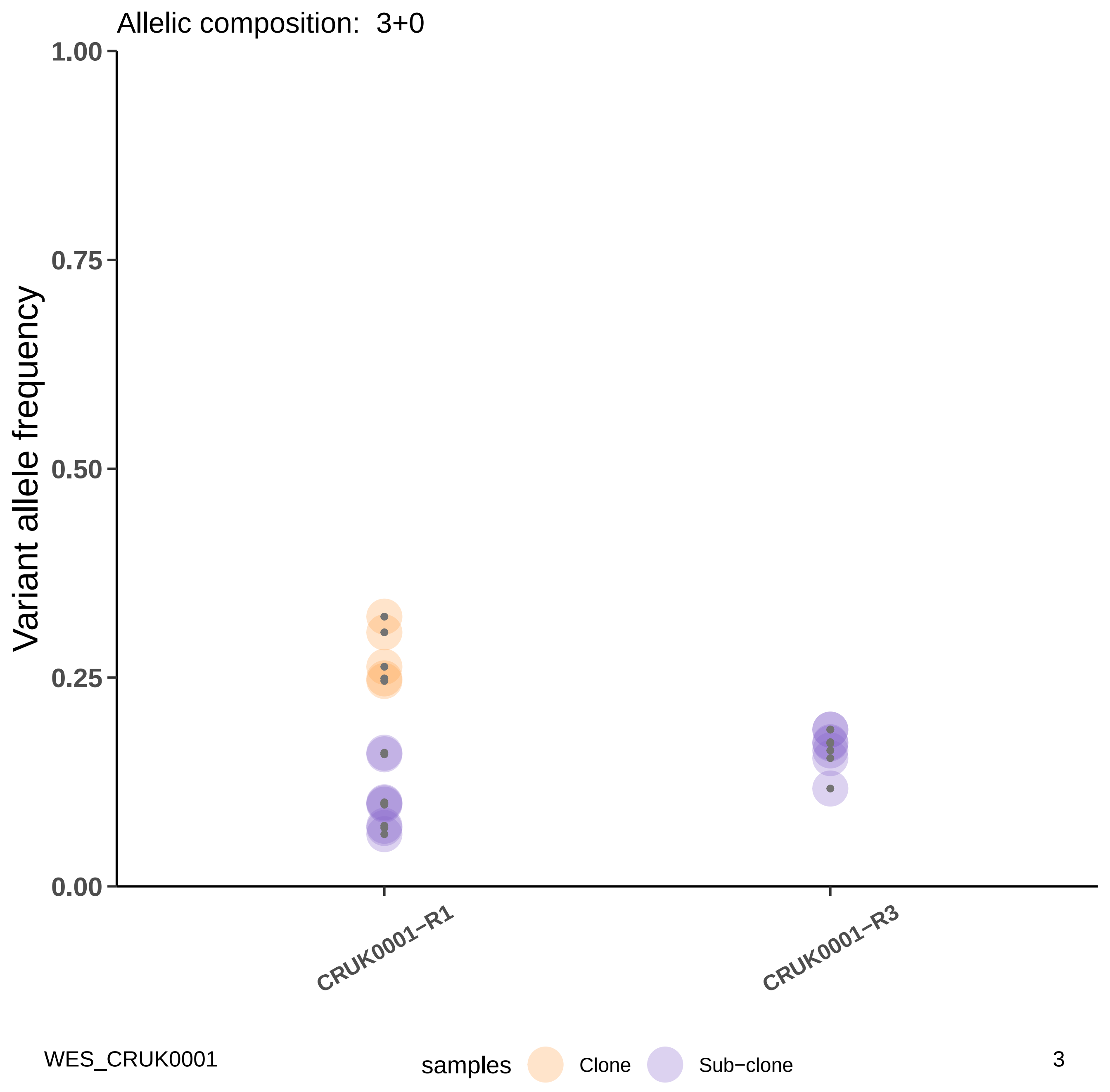

WES_CRUK0001

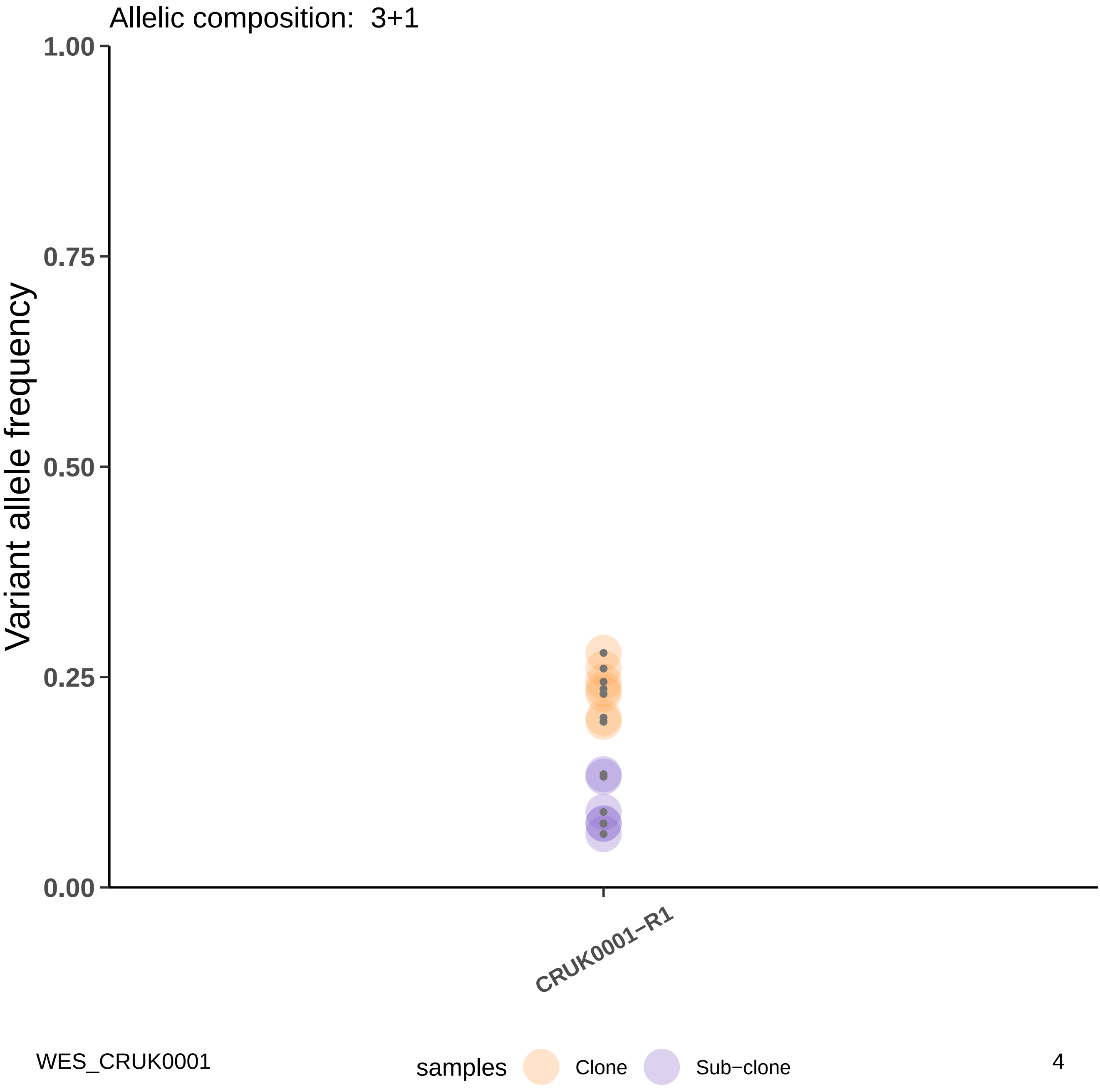

WES_CRUK0001

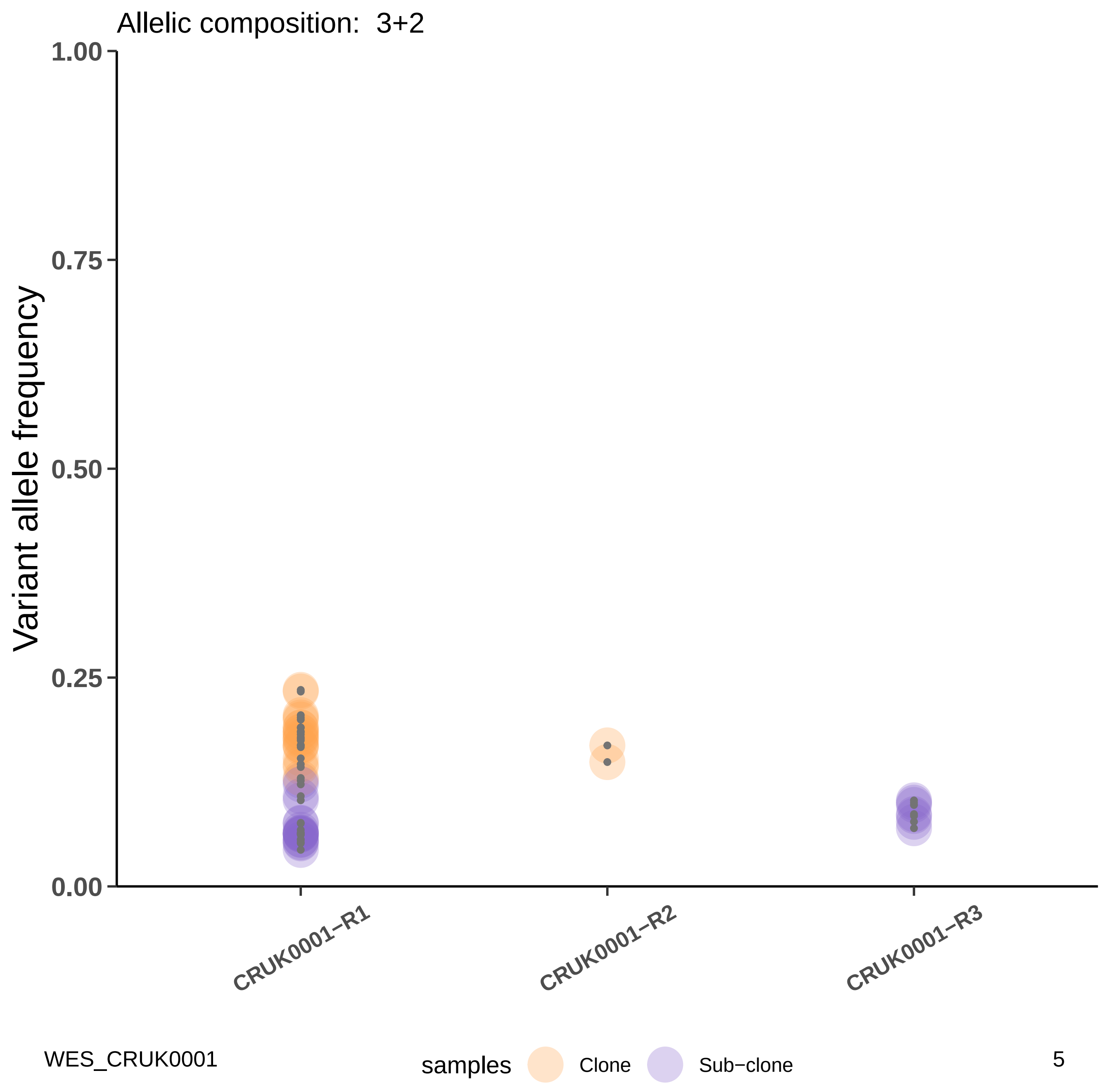

WES_CRUK0001

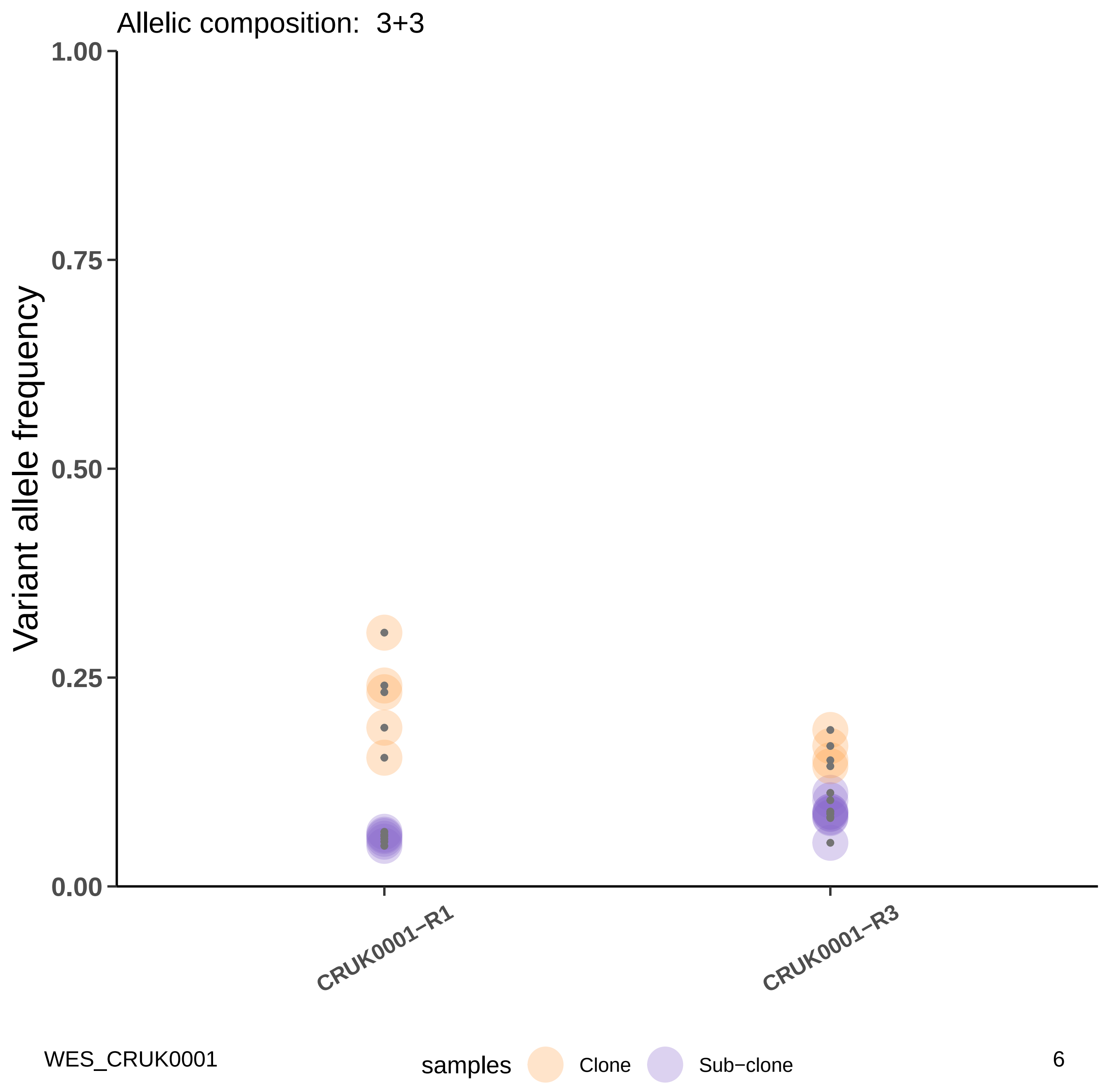

WES_CRUK0001

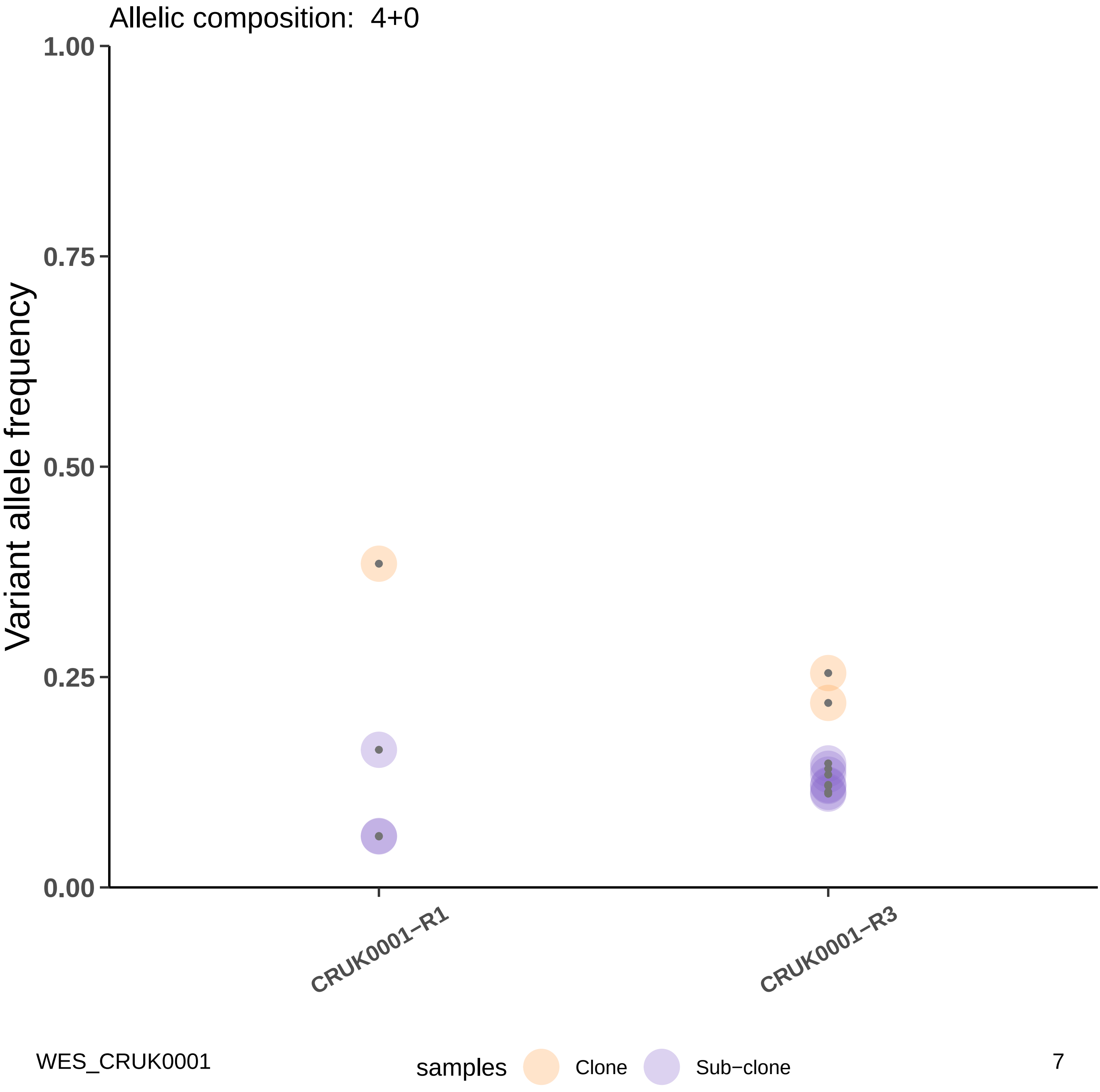

WES_CRUK0001

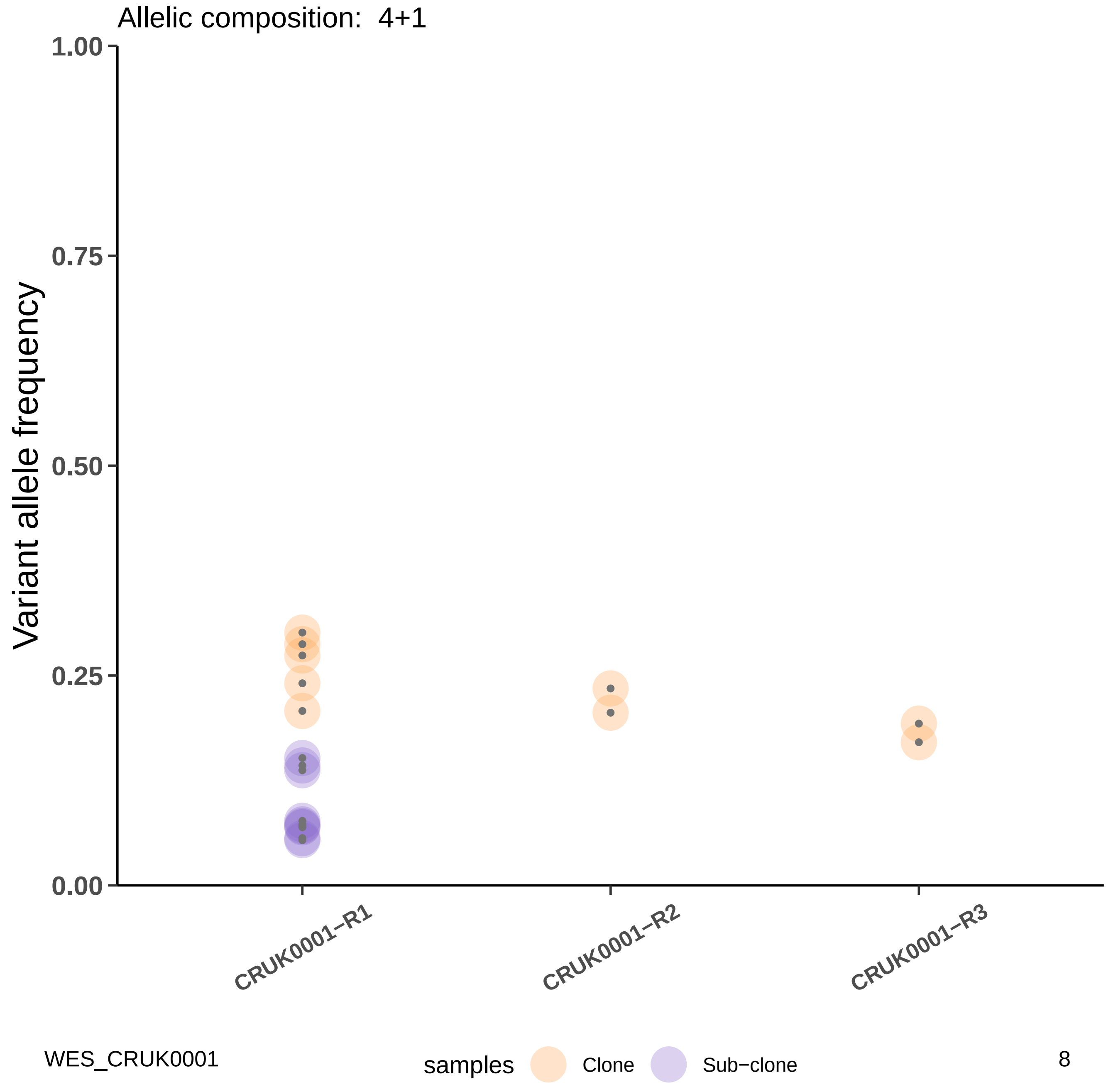

WES_CRUK0001

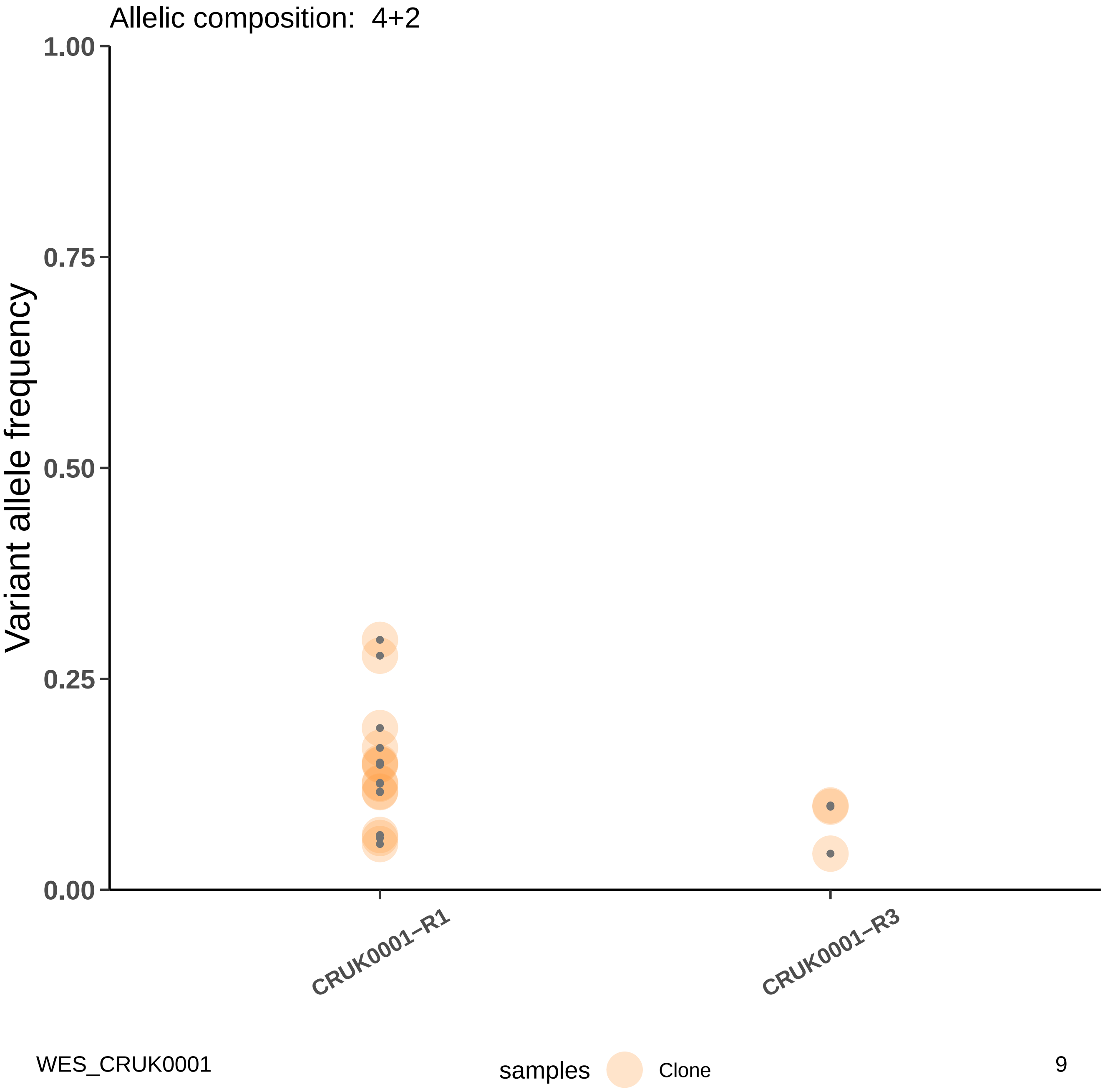

WES_CRUK0001

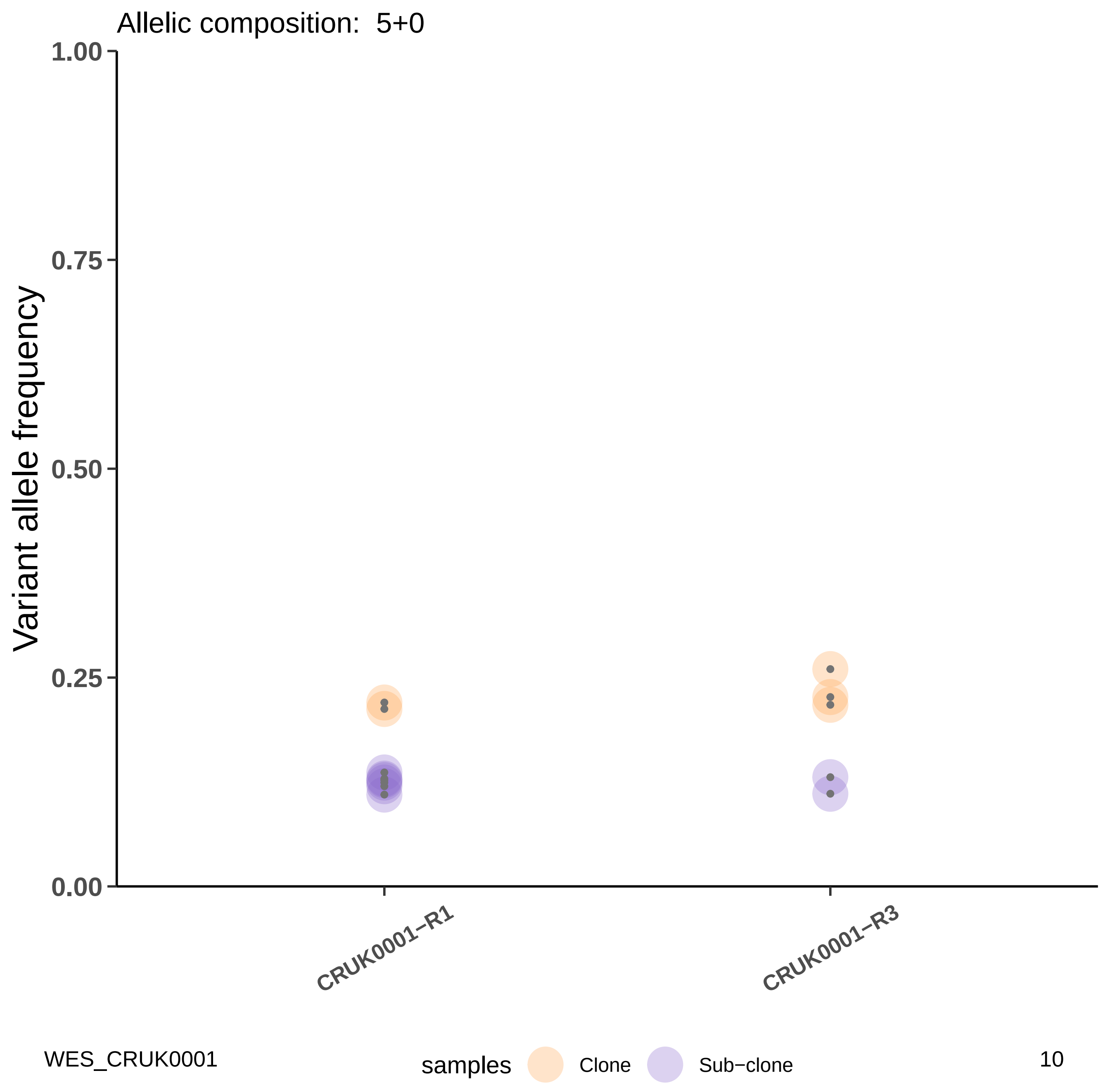

WES_CRUK0001

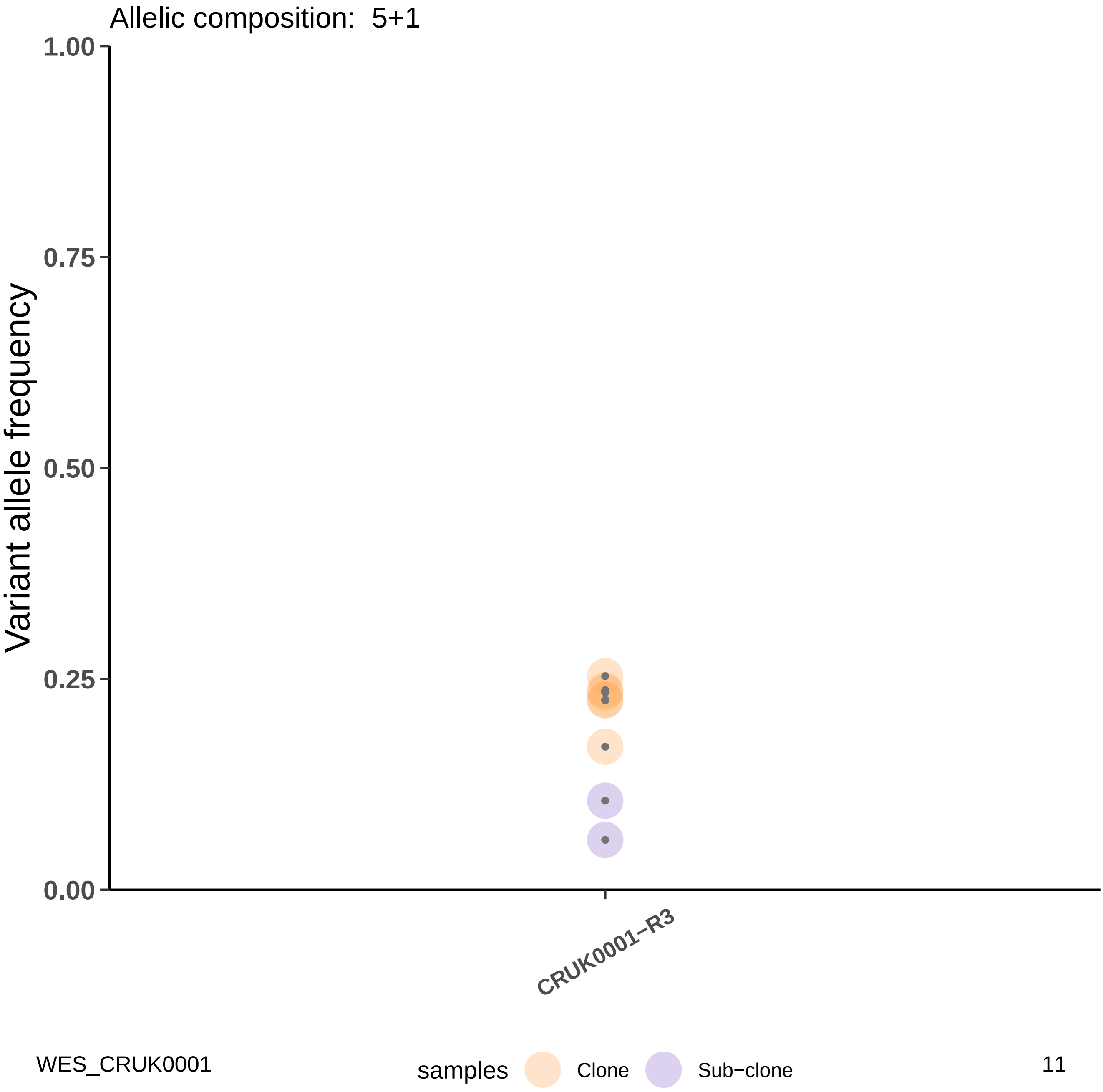

Allelic composition: 5+1
Variant allele frequency
1.00
0.75
0.50
0.25
0.00
CRUK0001−R3
samples
Clone
Sub−clone

Allelic composition: 5+2

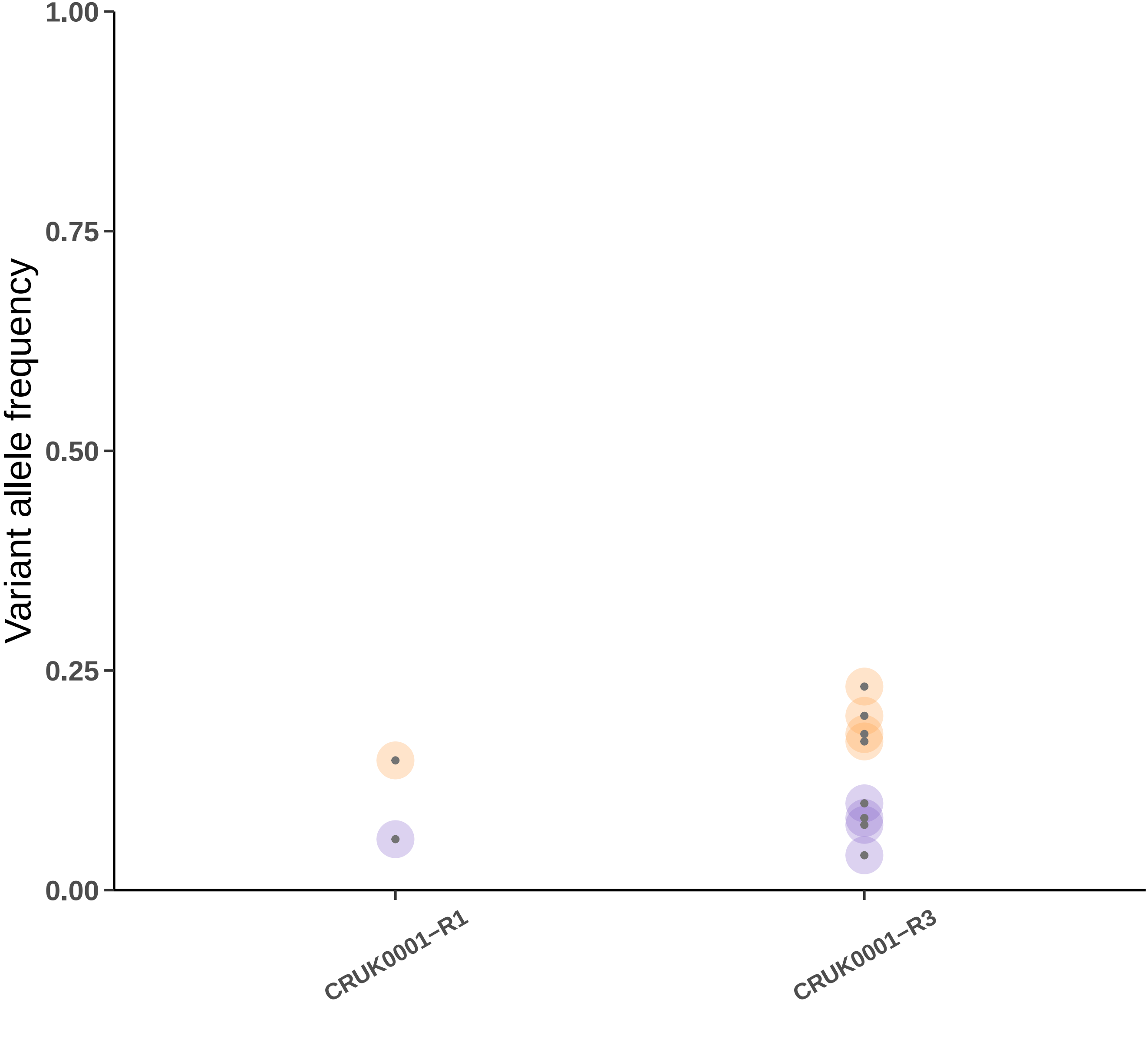

WES_CRUK0001

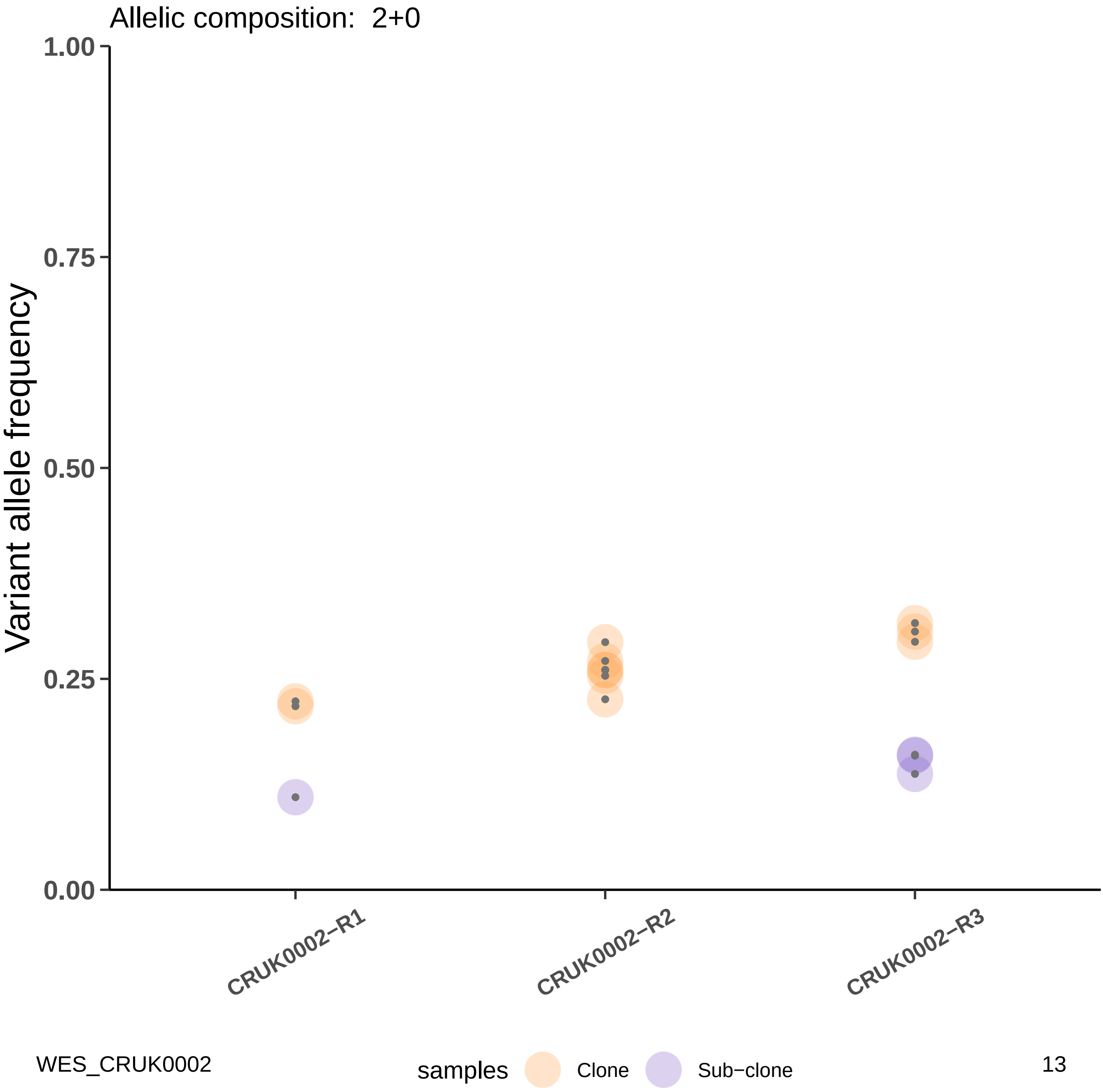

WES_CRUK0002

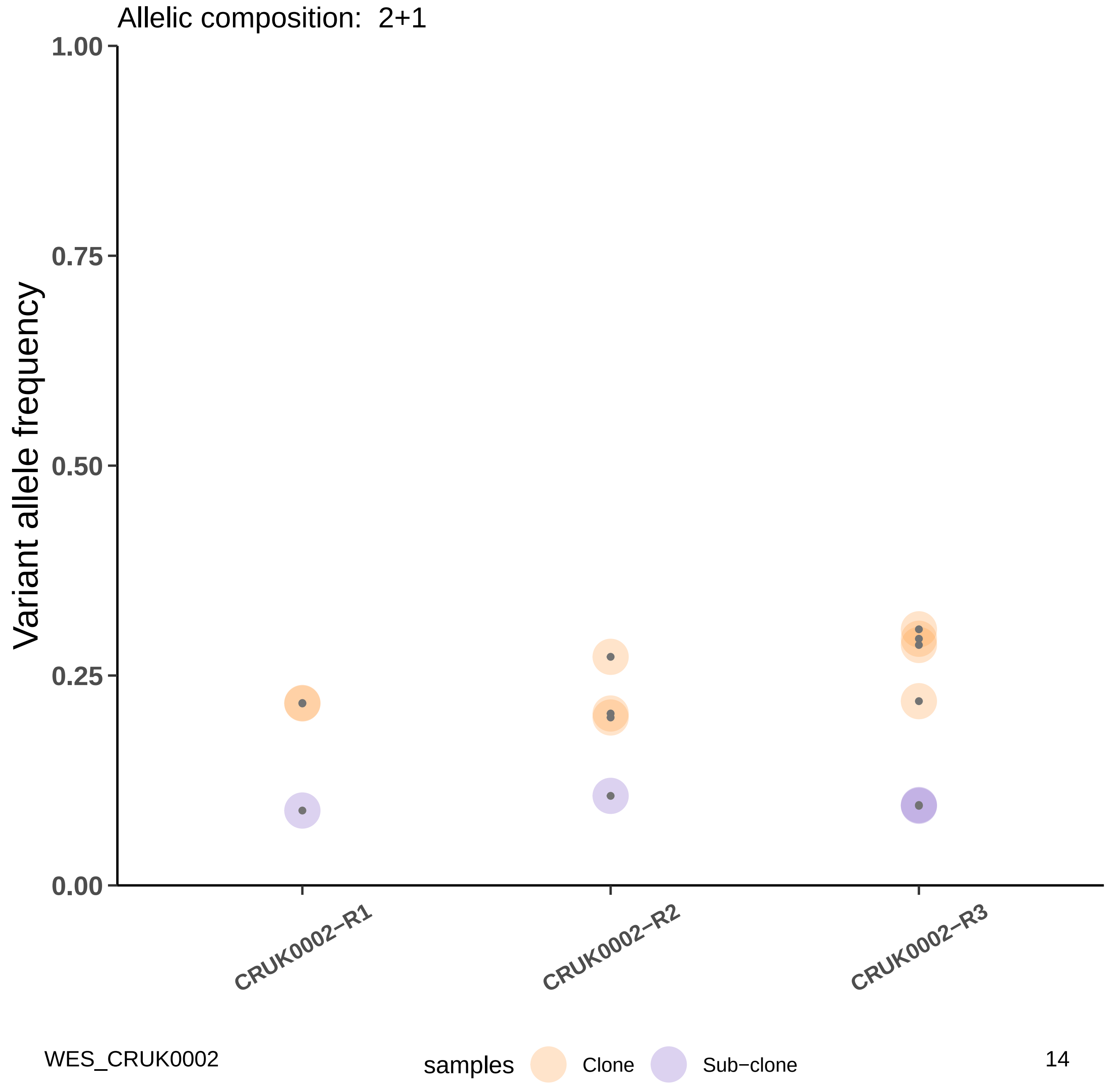

WES_CRUK0002

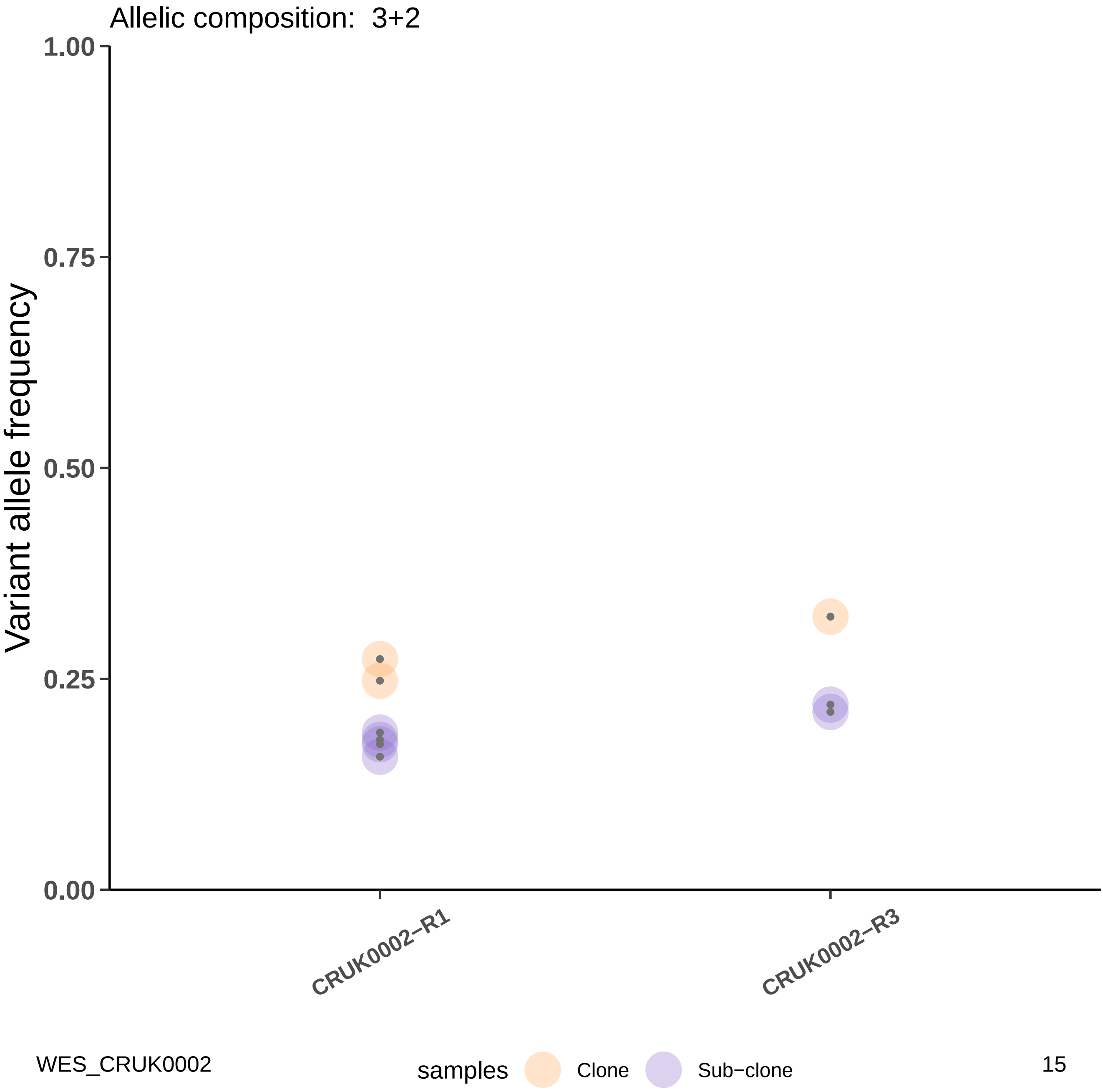

WES_CRUK0002

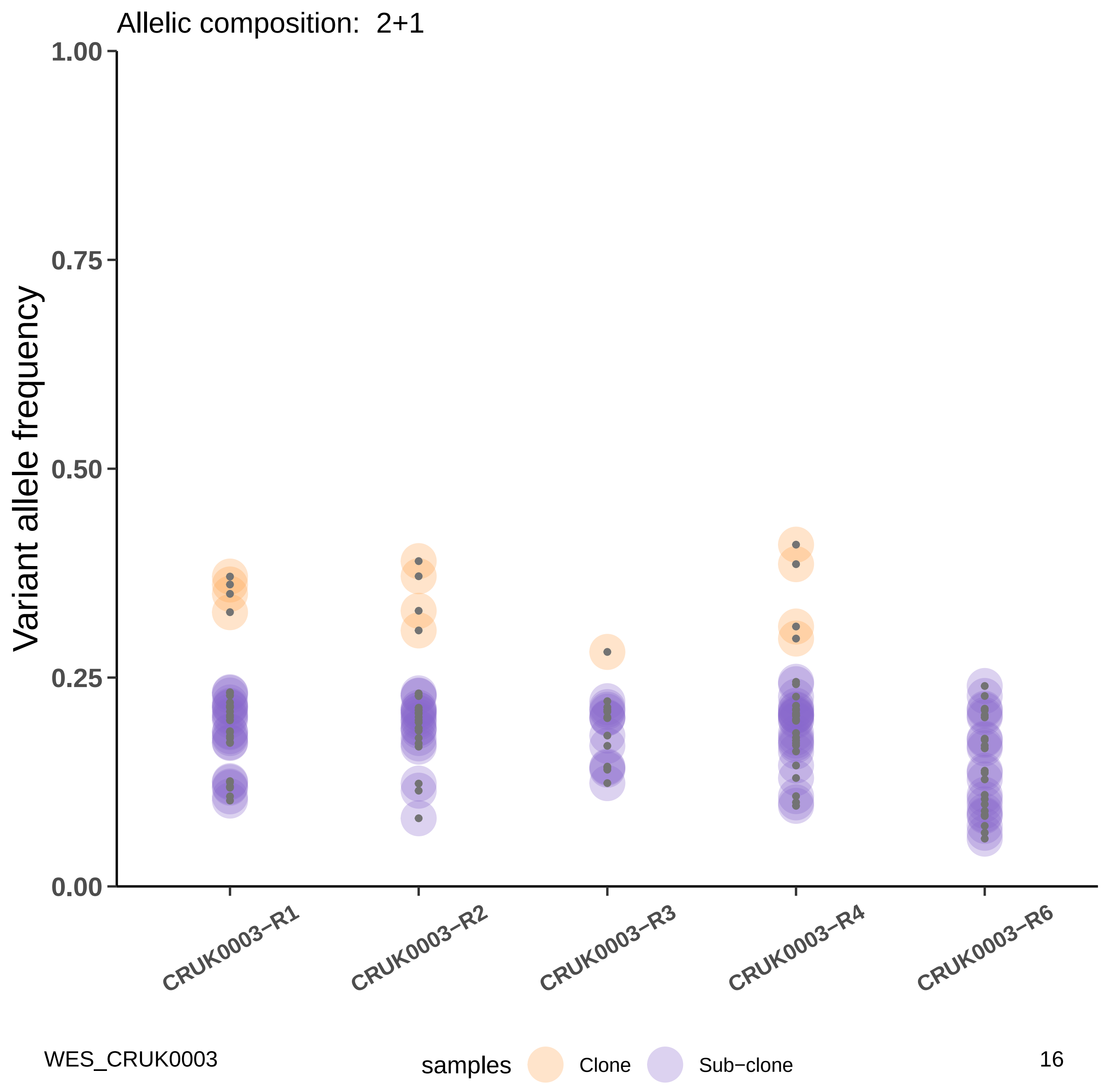

WES_CRUK0003

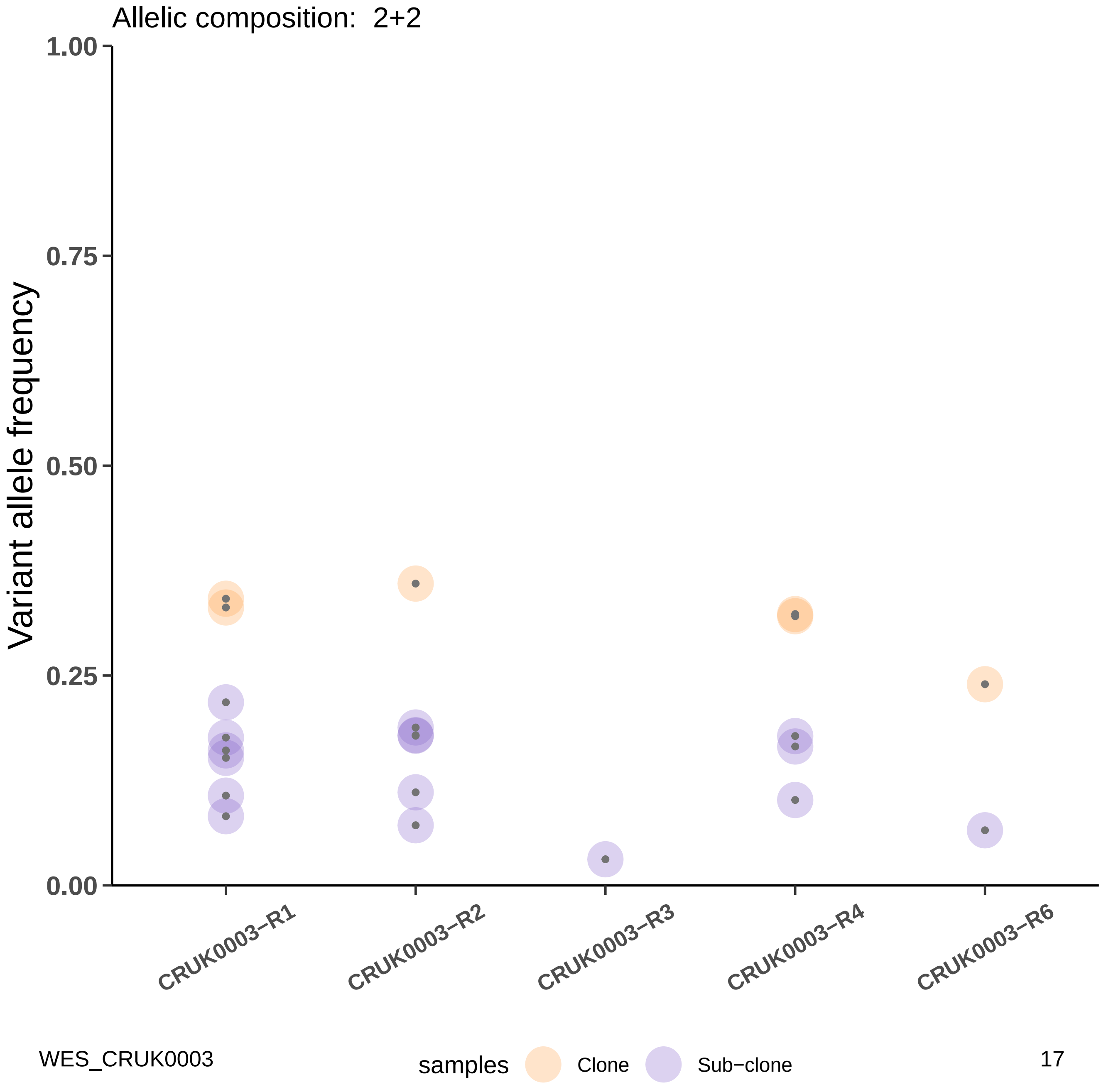

WES_CRUK0003

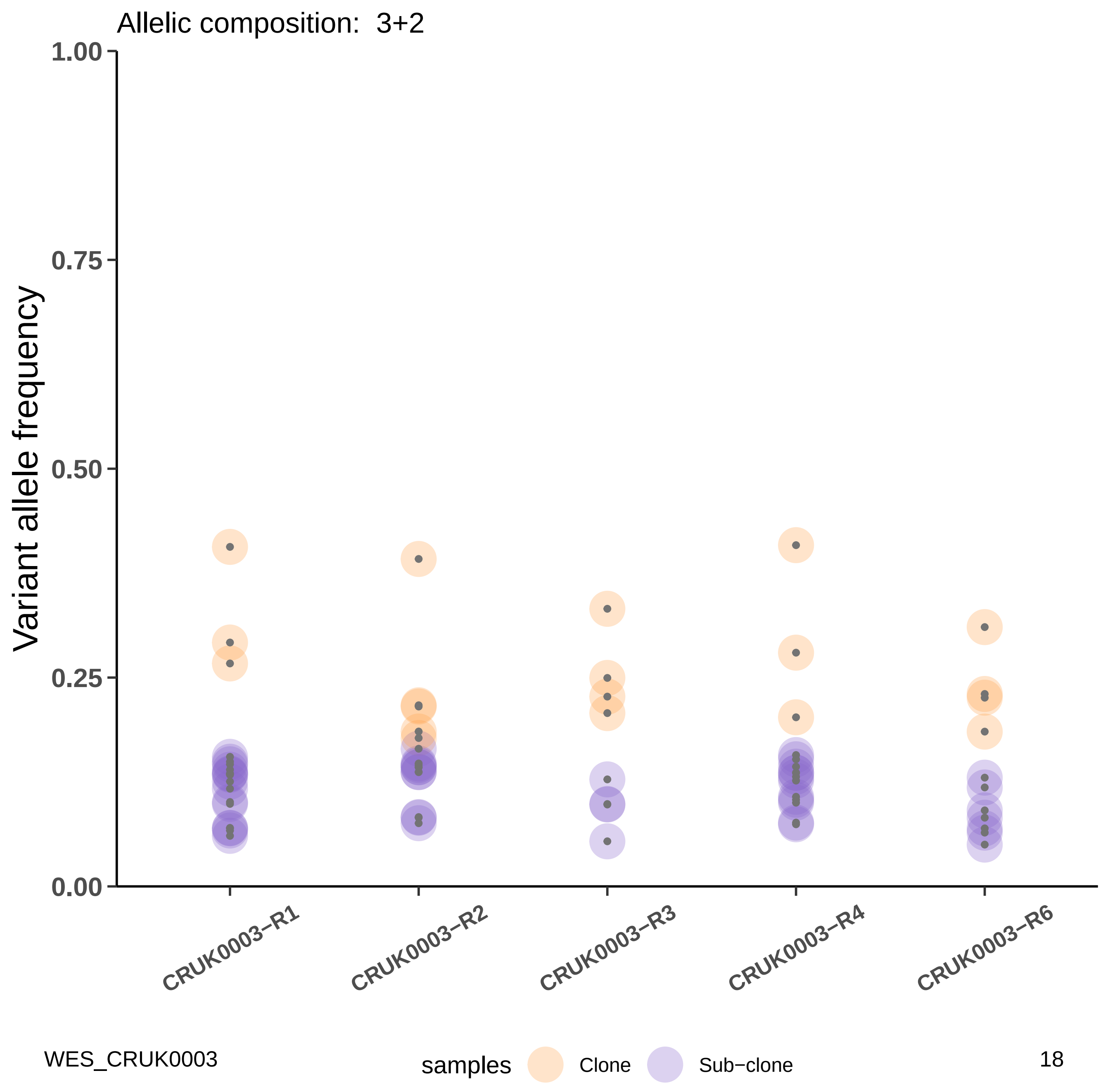

WES_CRUK0003

Allelic composition: 4+2

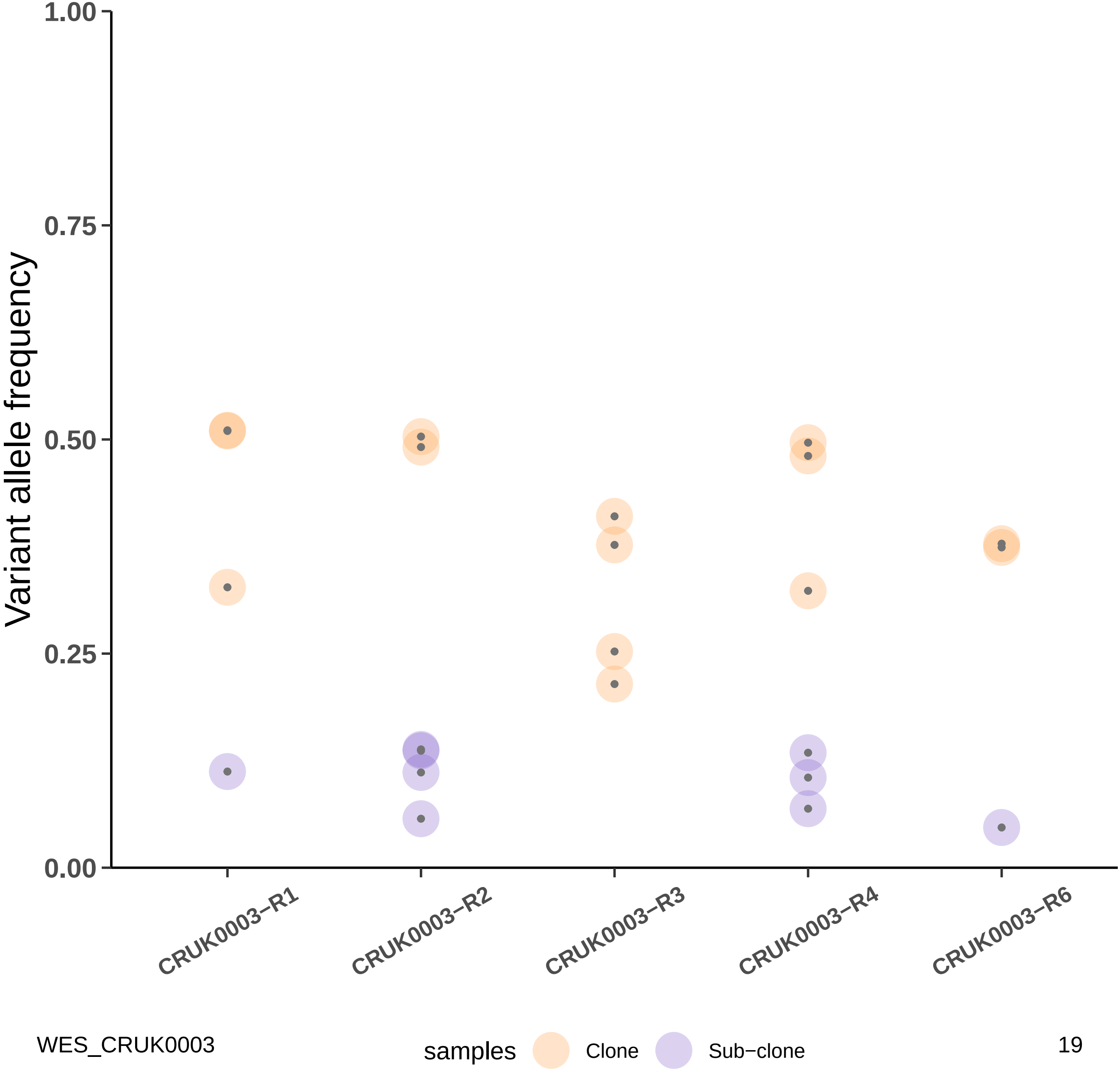

WES_CRUK0003

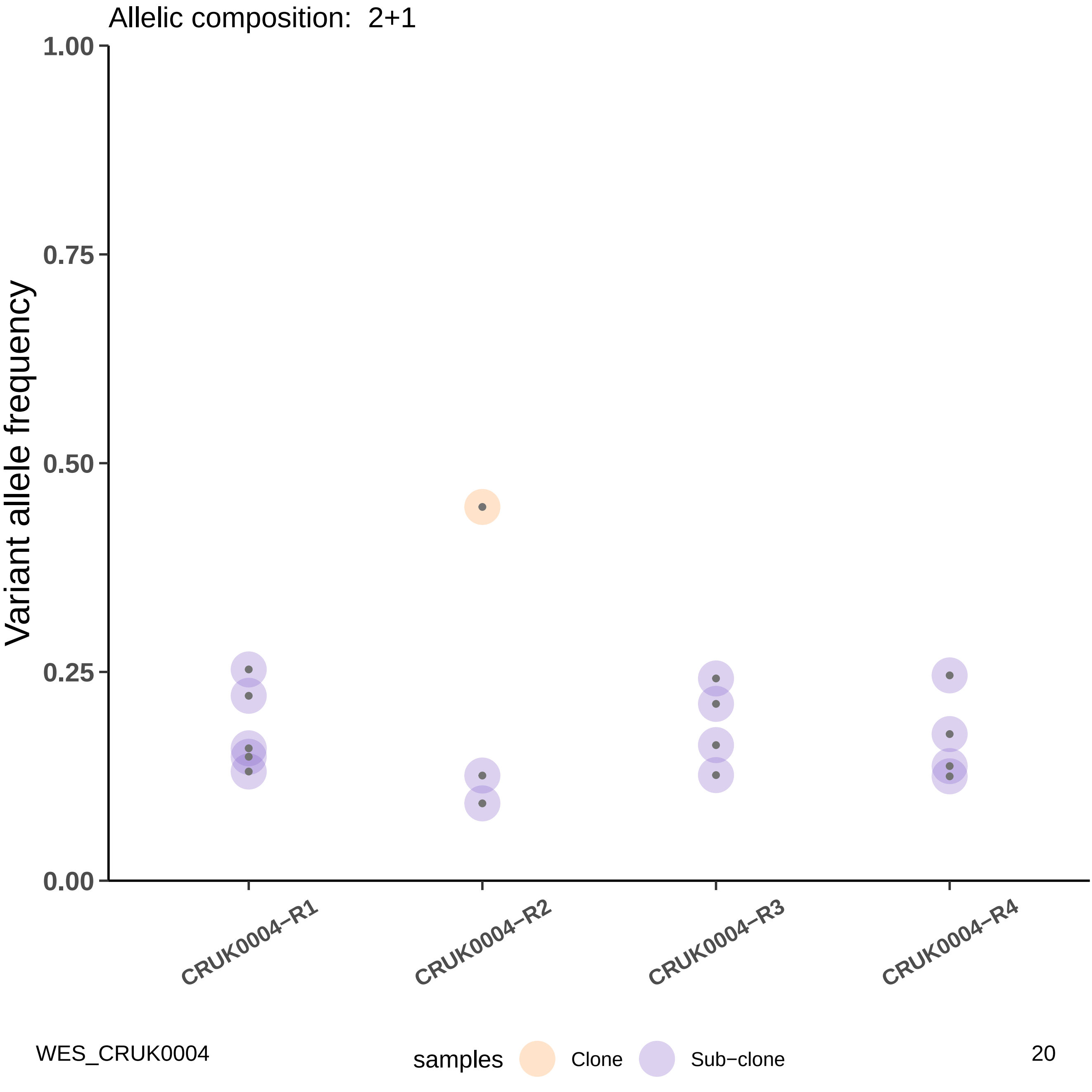
Allelic composition: 2+1
Variant allele frequency
1.00
0.75
0.50
0.25
0.00
CRUK0004−R1
CRUK0004−R2
CRUK0004−R3
CRUK0004−R4
samples
Clone
Sub−clone

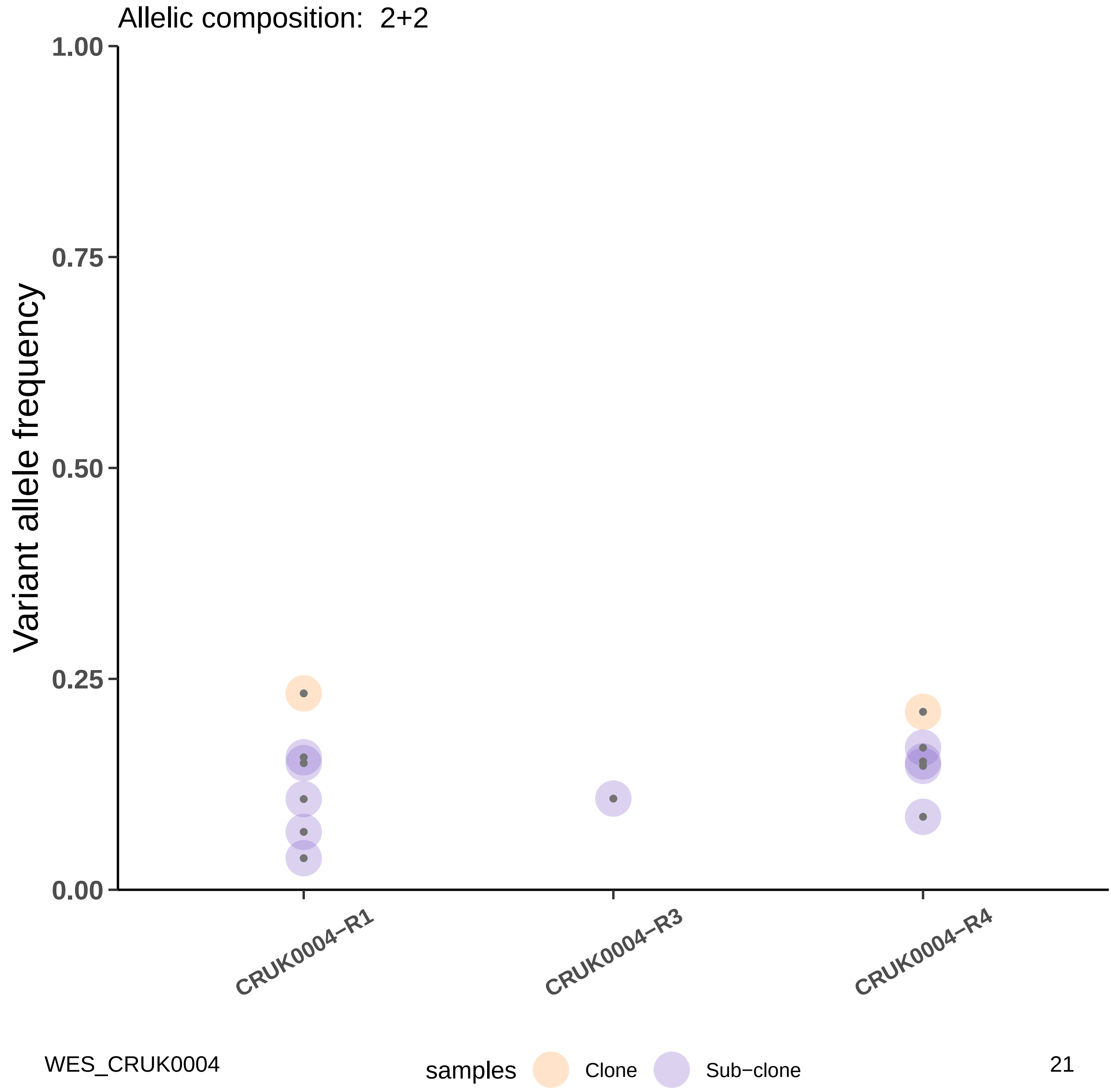
Allelic composition: 2+2
Variant allele frequency
1.00
0.75
0.50
0.25
0.00
CRUK0004−R1
CRUK0004−R3
CRUK0004−R4
samples
Clone
Sub−clone

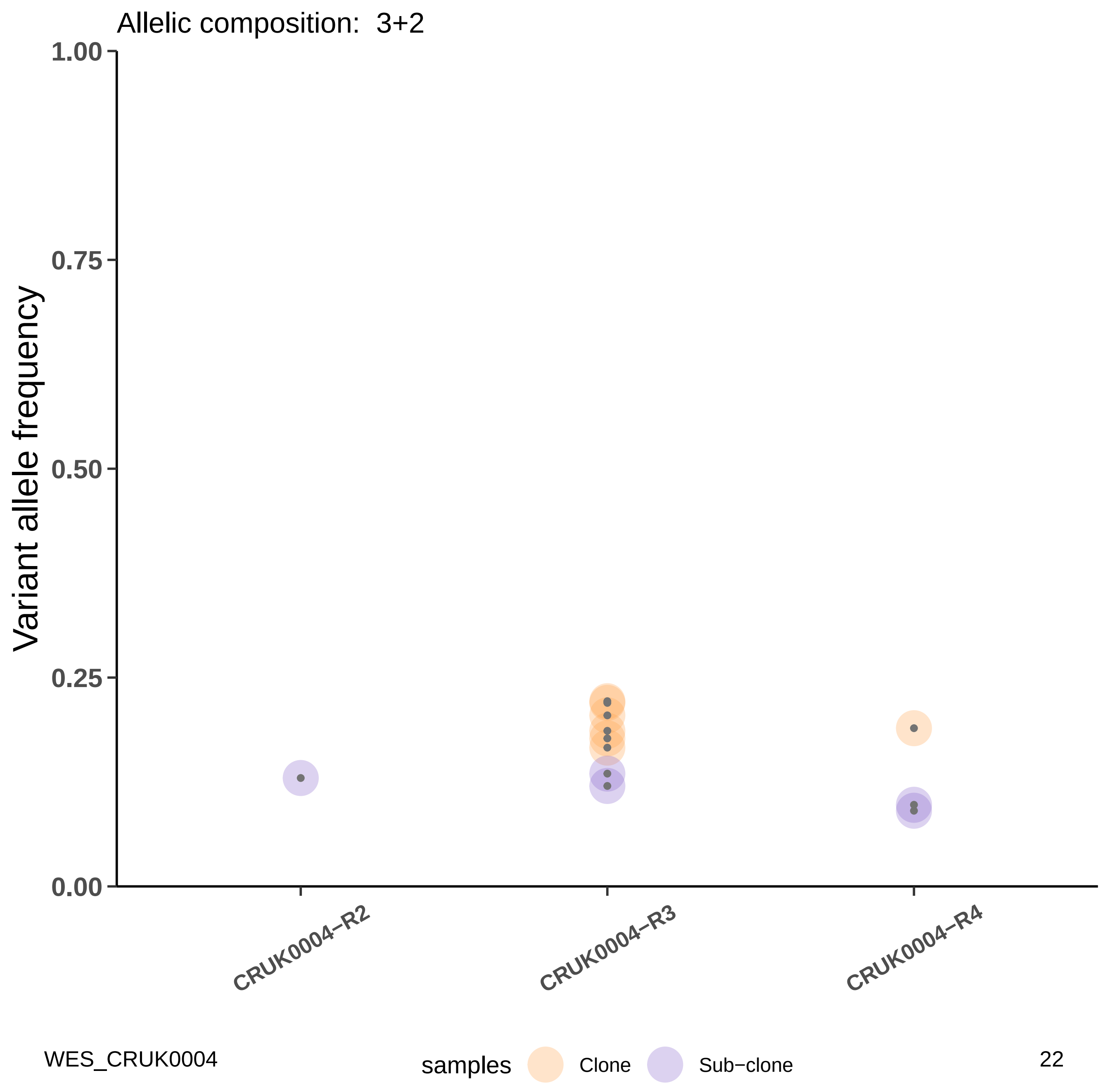

WES_CRUK0004

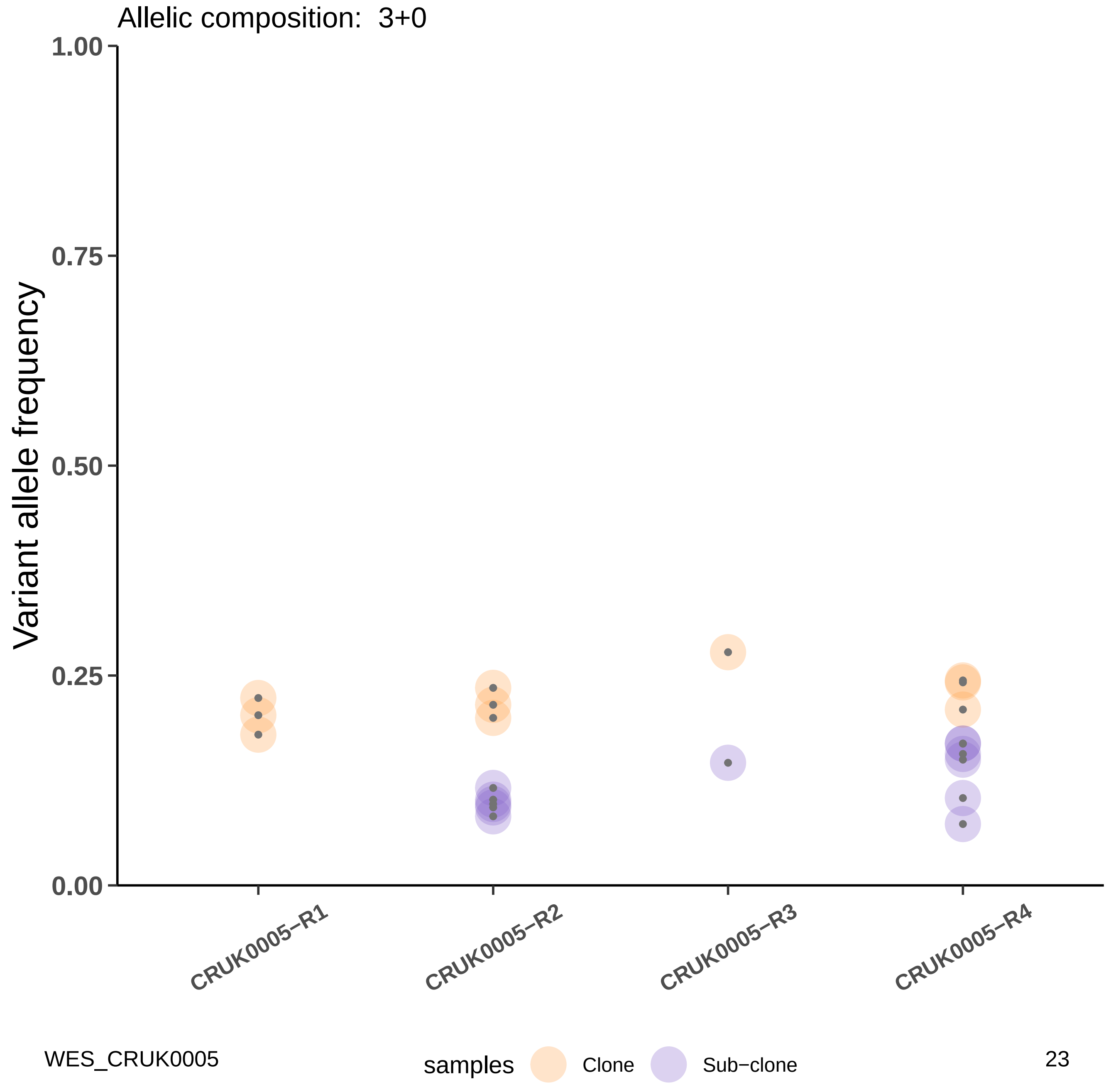
Allelic composition: 3+0
Variant allele frequency
1.00
0.75
0.50
0.25
0.00
CRUK0005−R1
CRUK0005−R2
CRUK0005−R3
CRUK0005−R4
samples
Clone
Sub−clone

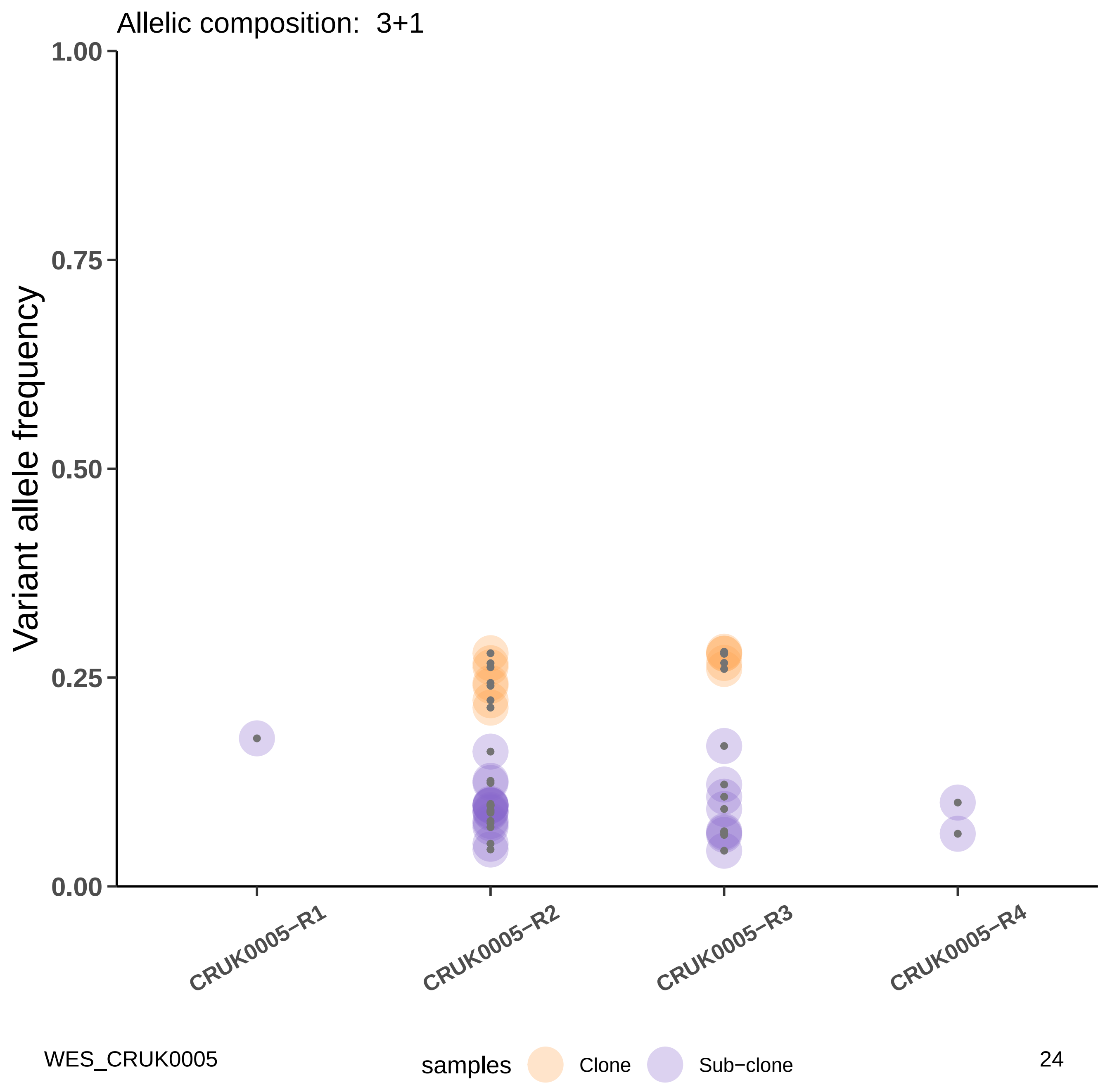

WES_CRUK0005

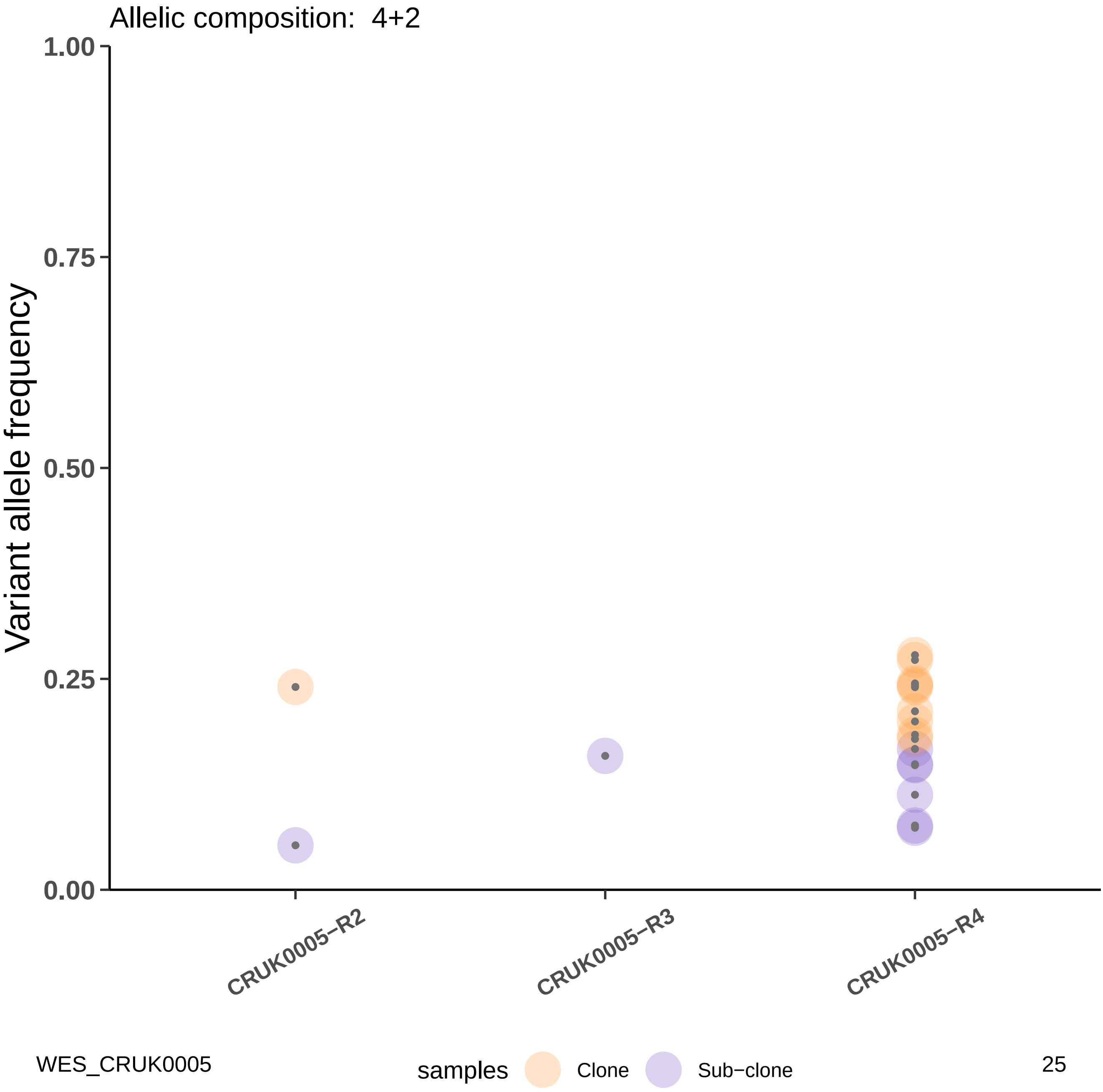

WES_CRUK0005

Allelic composition: 5+2

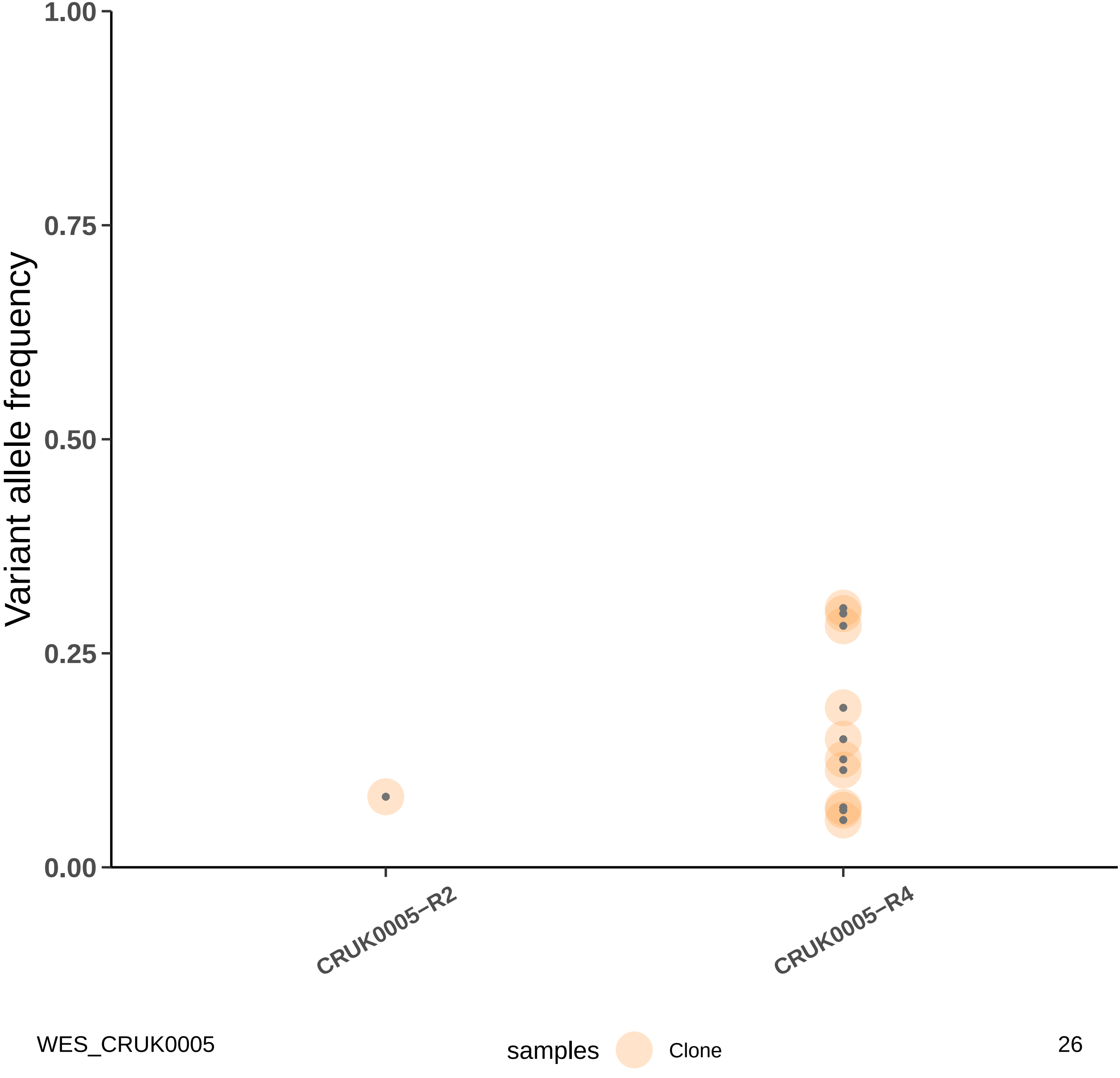

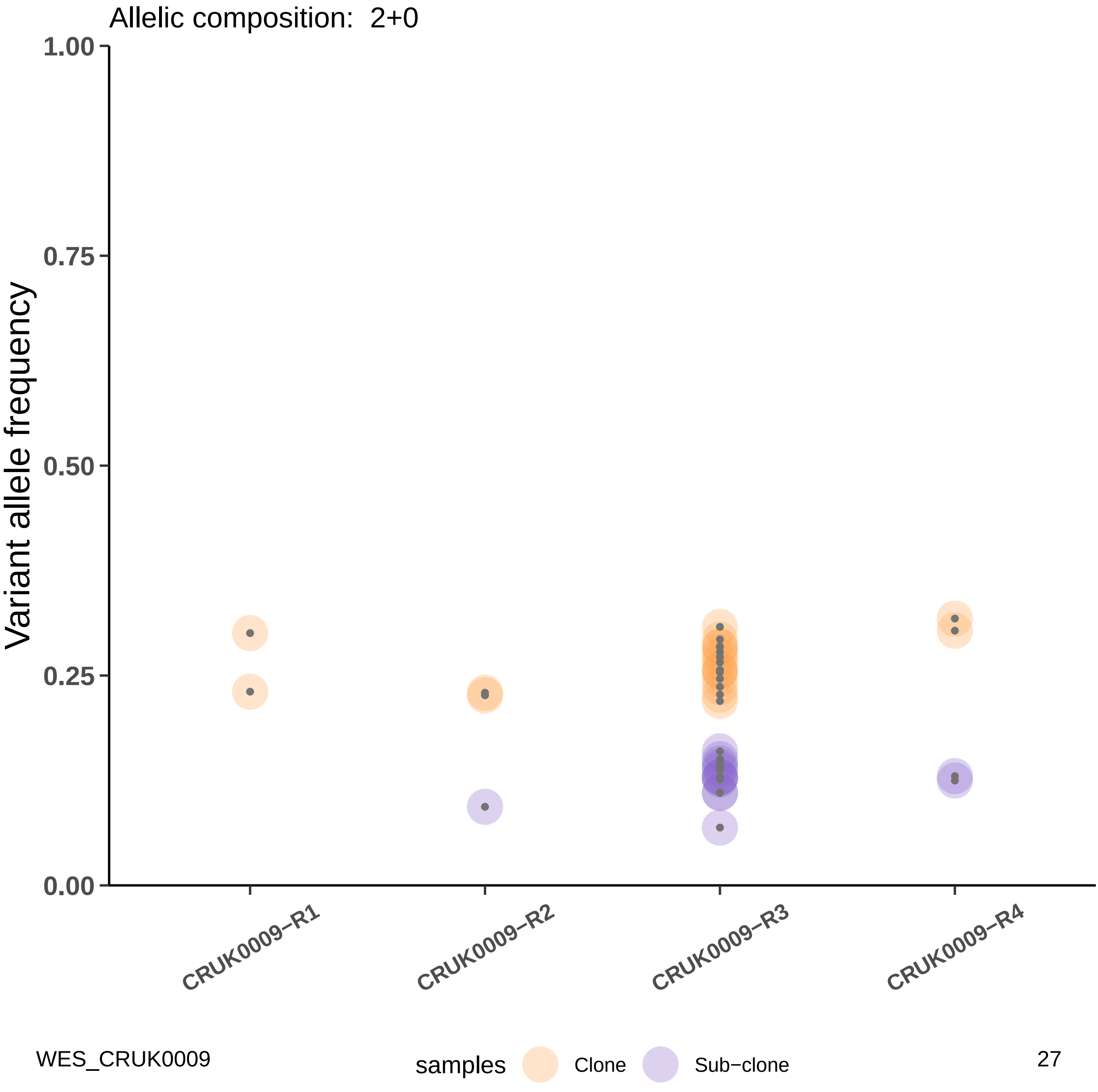

WES_CRUK0009

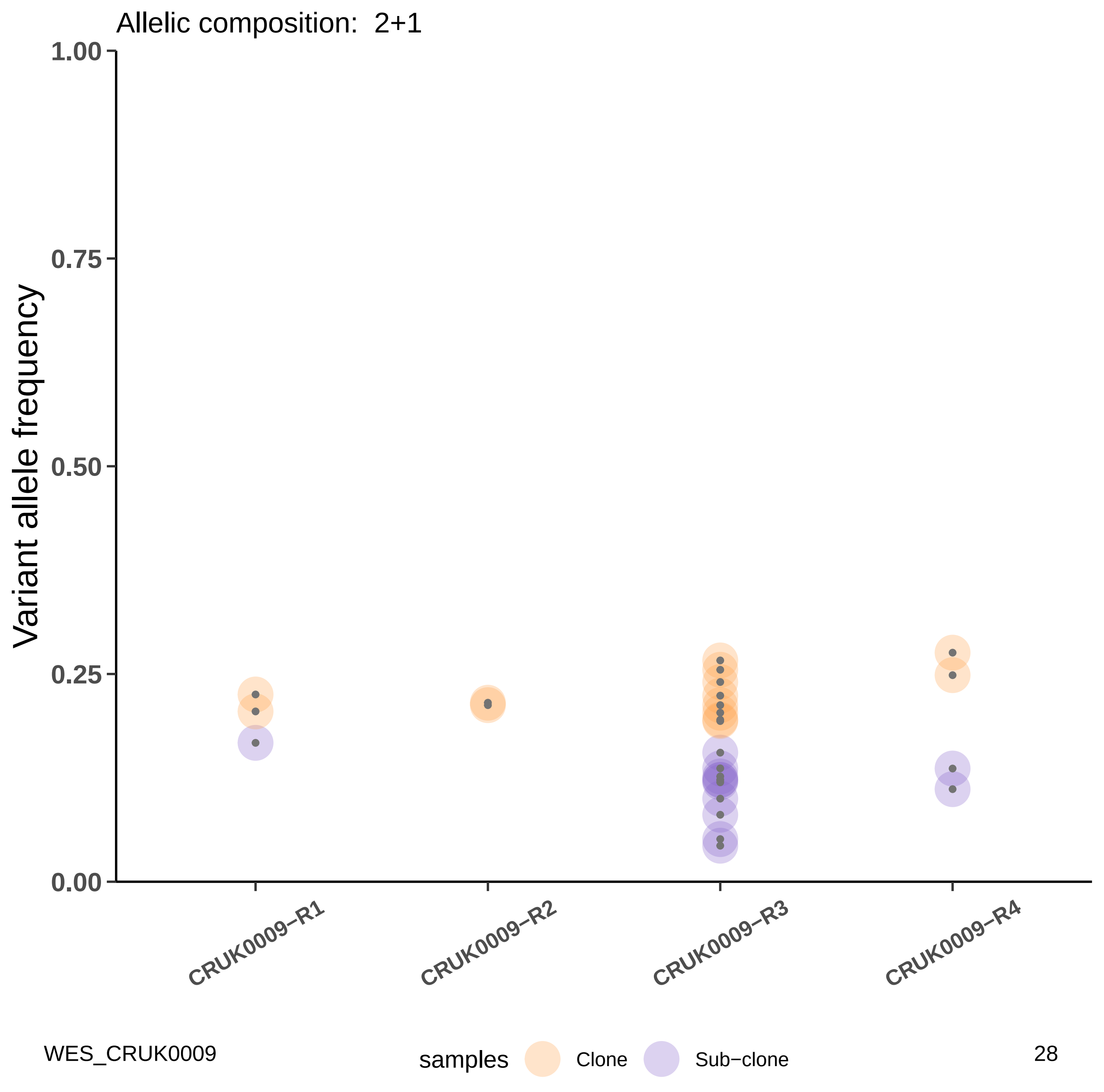
Allelic composition: 2+1
Variant allele frequency
1.00
0.75
0.50
0.25
0.00
CRUK0009−R1
CRUK0009−R2
CRUK0009−R3
CRUK0009−R4
samples
Clone
Sub−clone

Allelic composition: 2+2

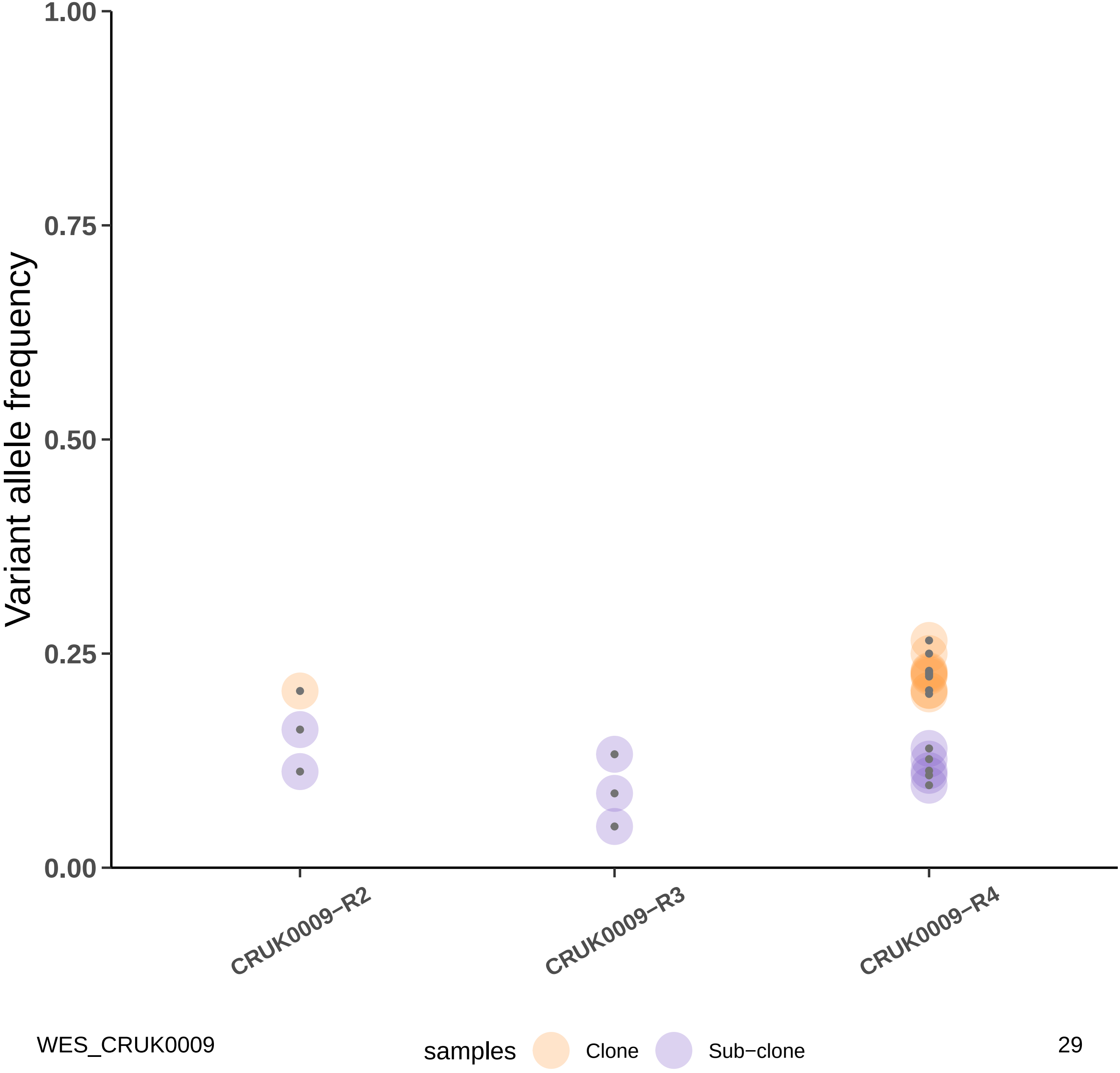

WES_CRUK0009

Allelic composition: 3+0

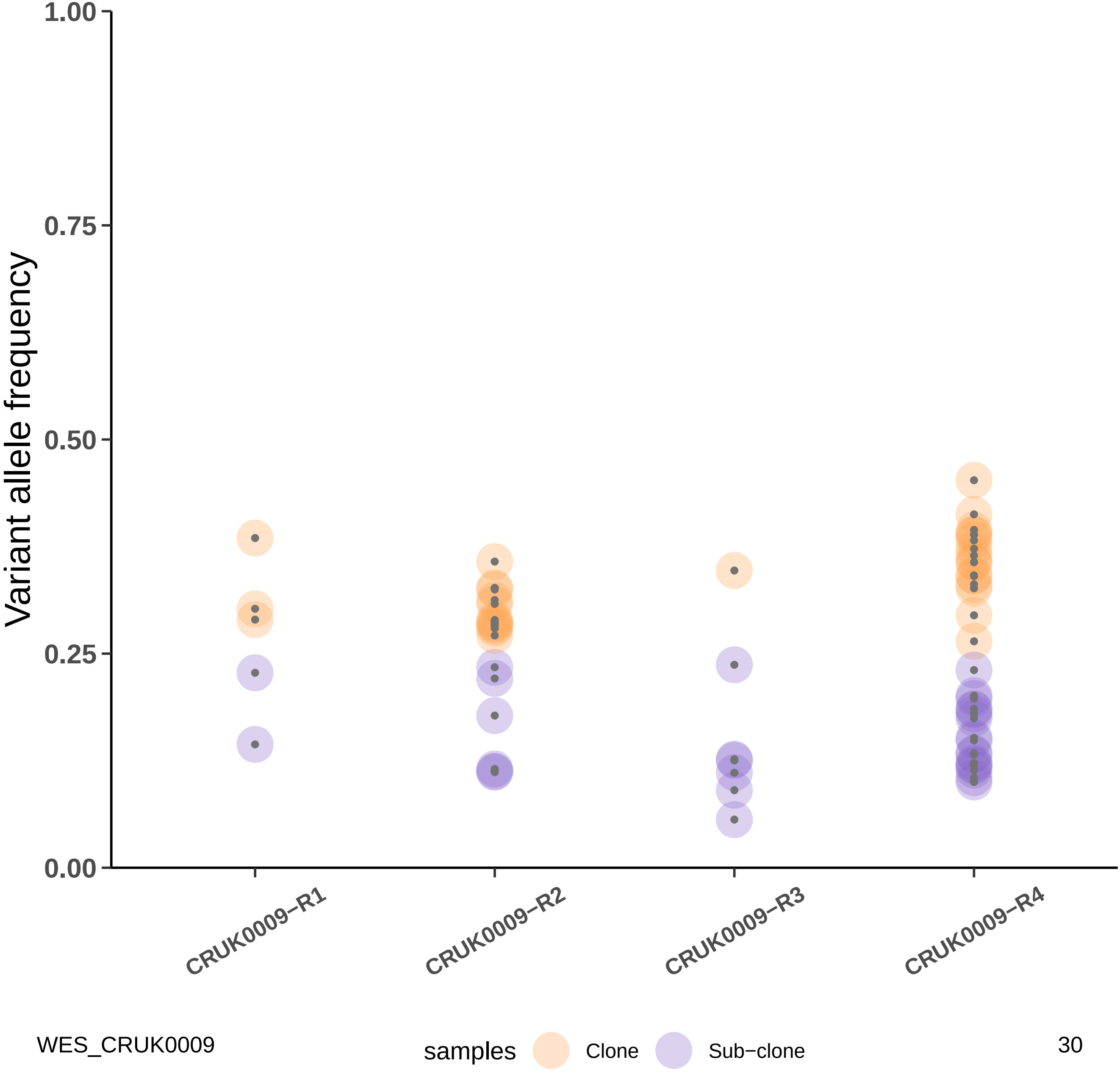

WES_CRUK0009

Allelic composition: 3+1

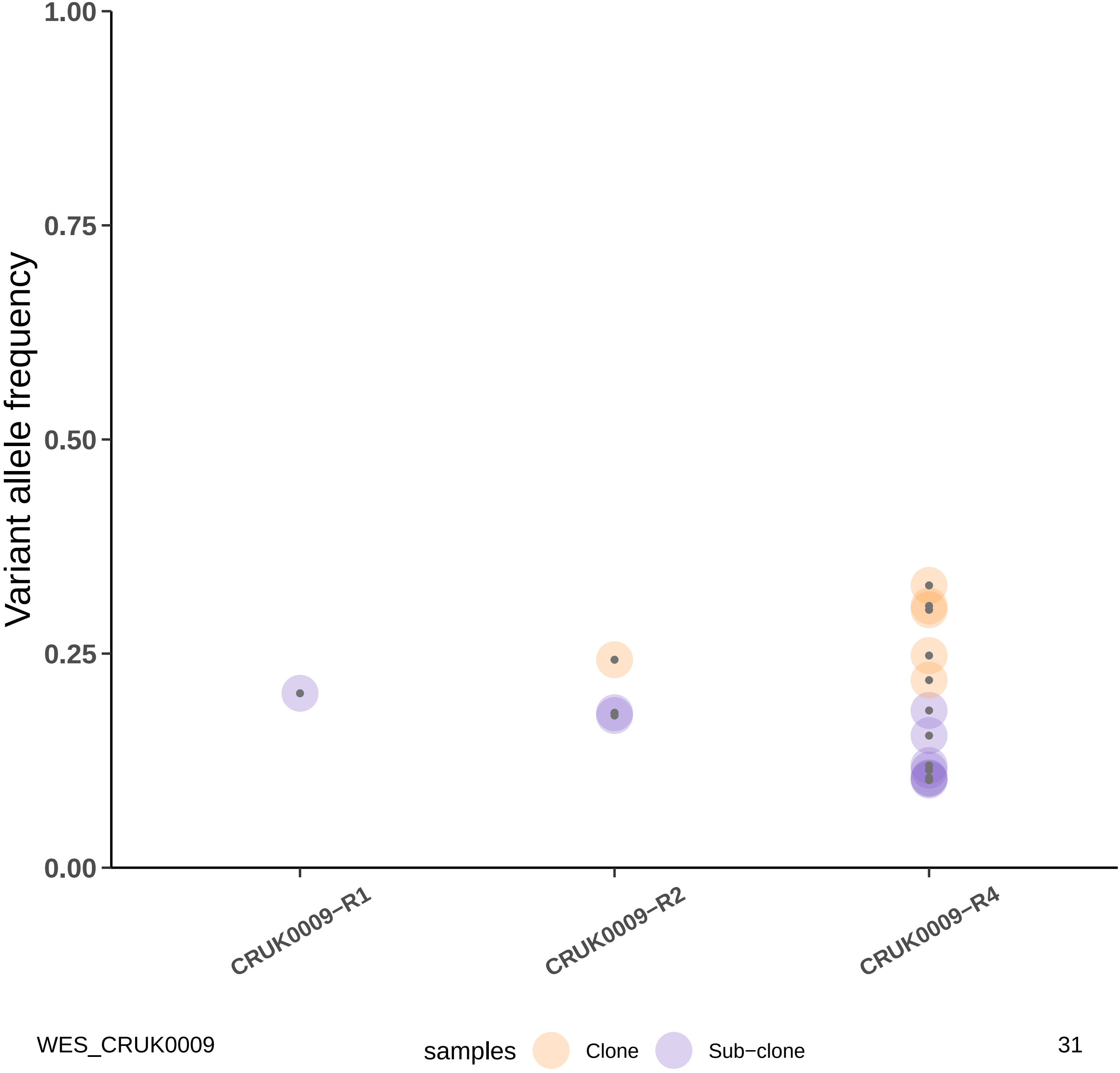

WES_CRUK0009

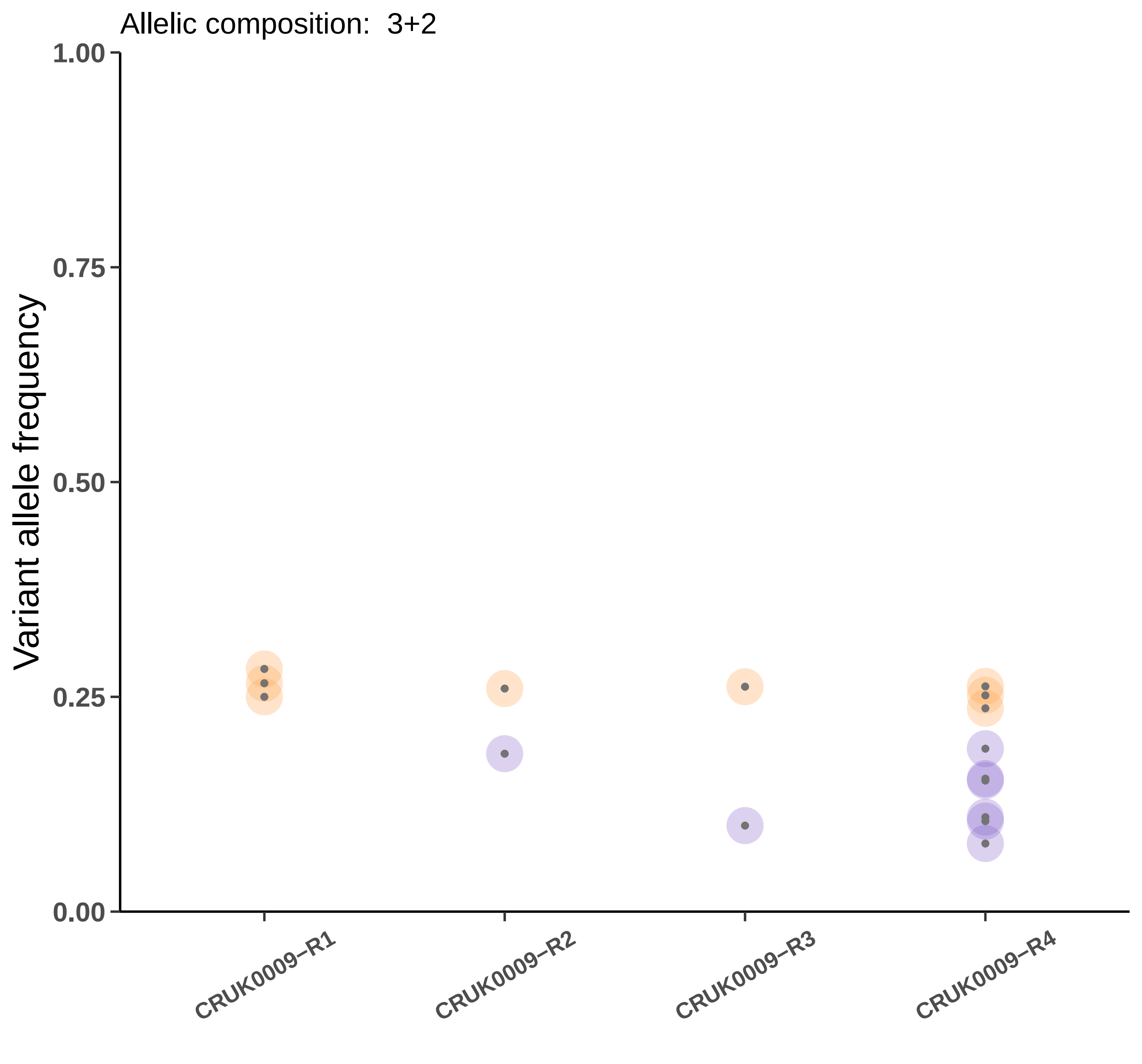
Allelic composition: 3+2
Variant allele frequency
1.00
0.75
0.50
0.25
0.00
CRUK0009−R1
CRUK0009−R2
CRUK0009−R3
CRUK0009−R4
samples
Clone
Sub−clone

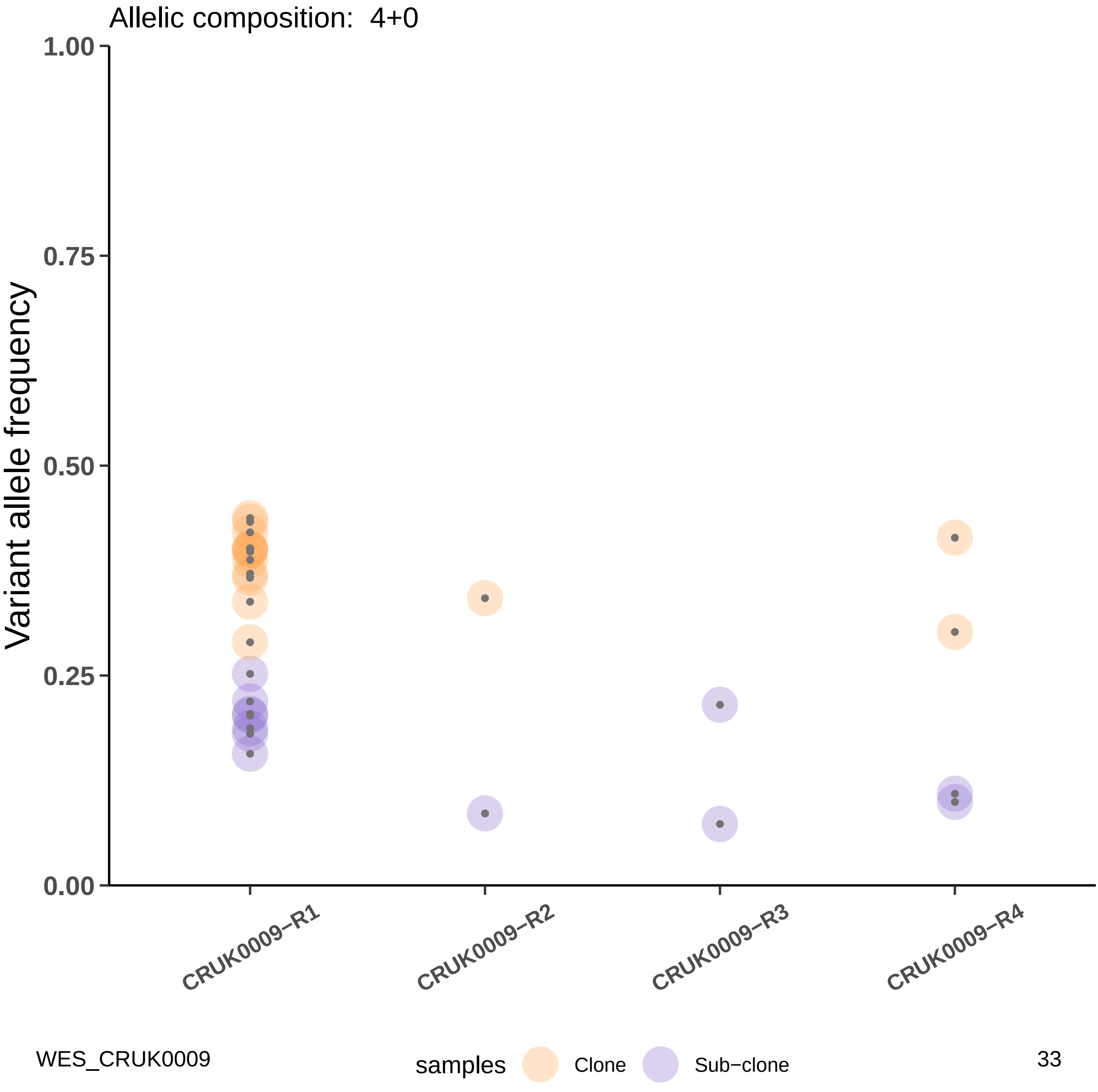

WES_CRUK0009

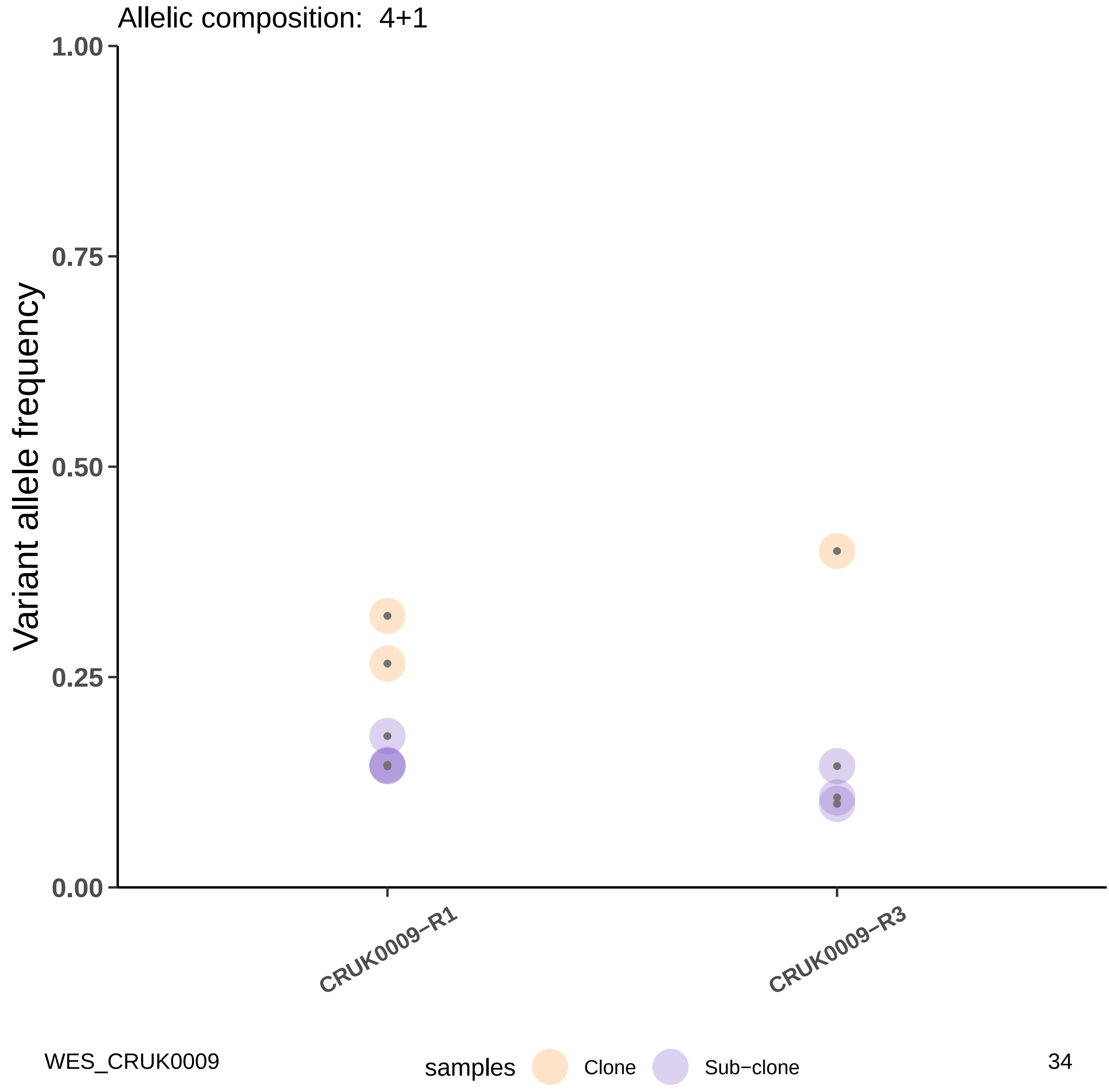

WES_CRUK0009

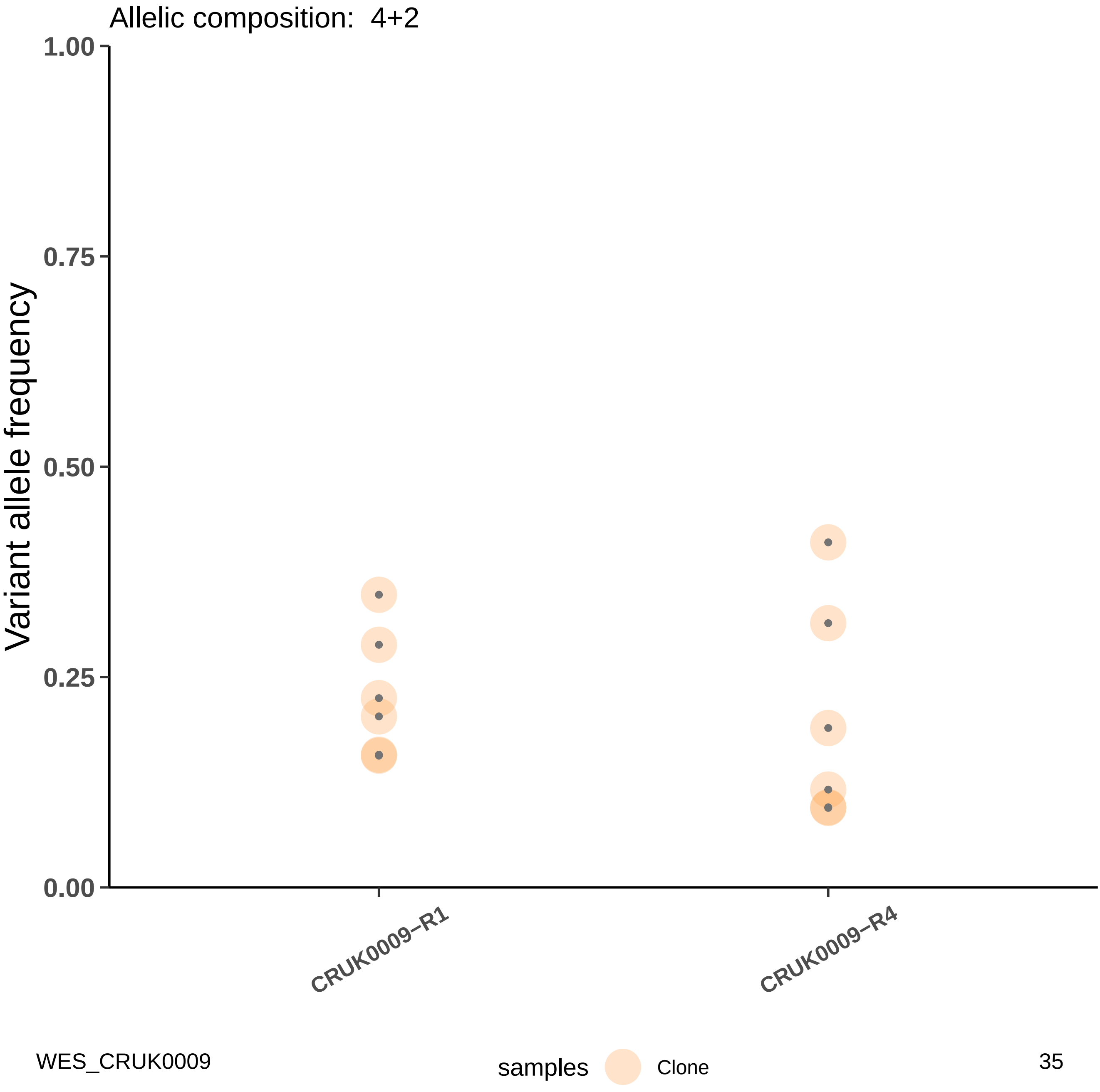
Allelic composition: 4+2
Variant allele frequency
1.00
0.75
0.50
0.25
0.00
CRUK0009−R1
CRUK0009−R4
samples
Clone

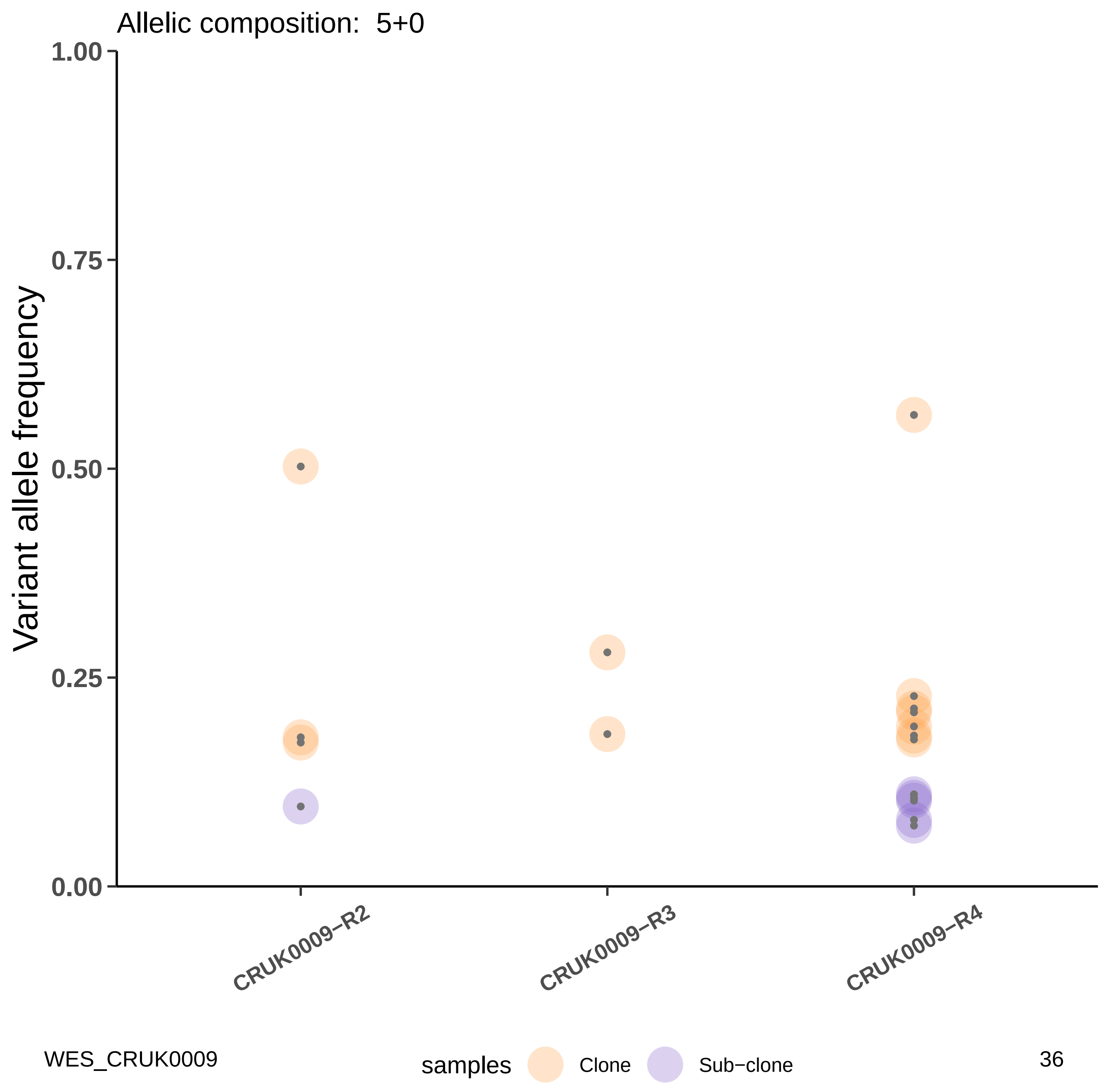

WES_CRUK0009

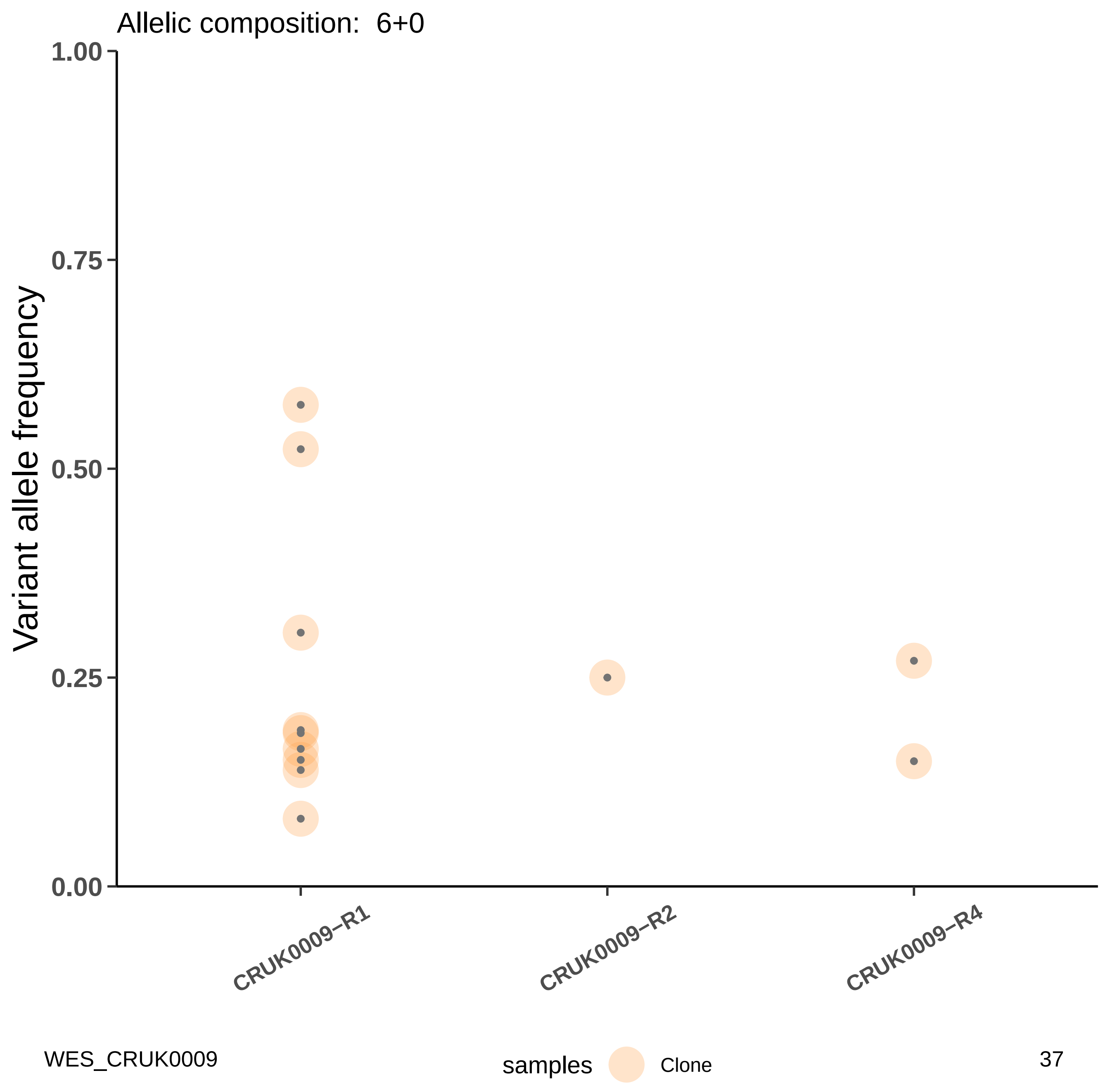

Allelic composition: 6+0
Variant allele frequency
1.00
0.75
0.50
0.25
0.00
CRUK0009−R1
CRUK0009−R2
CRUK0009−R4
samples
Clone

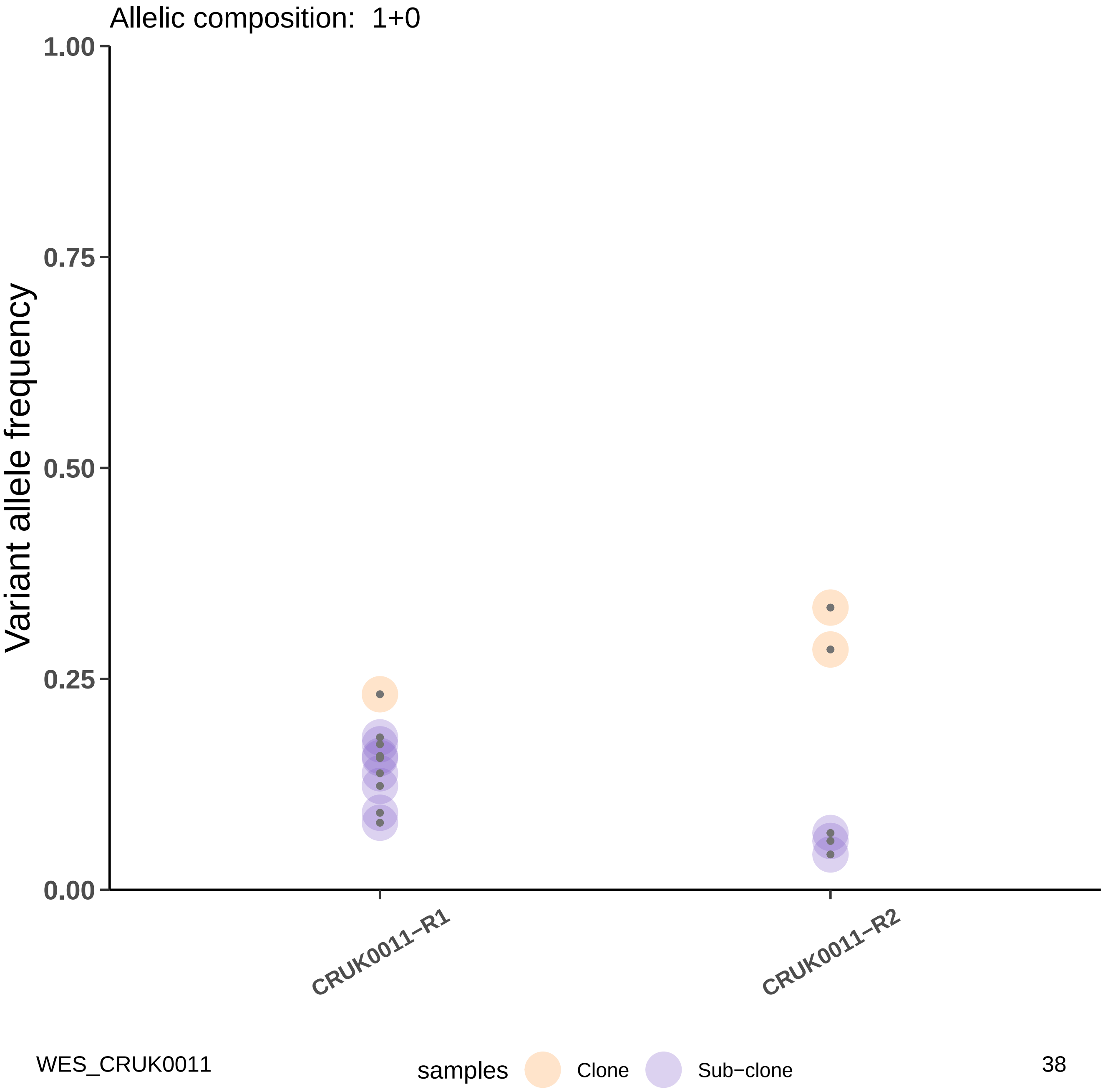
Allelic composition: 1+0
Variant allele frequency
1.00
0.75
0.50
0.25
0.00
CRUK0011−R1
CRUK0011−R2
samples
Clone
Sub−clone

Allelic composition: 2+1

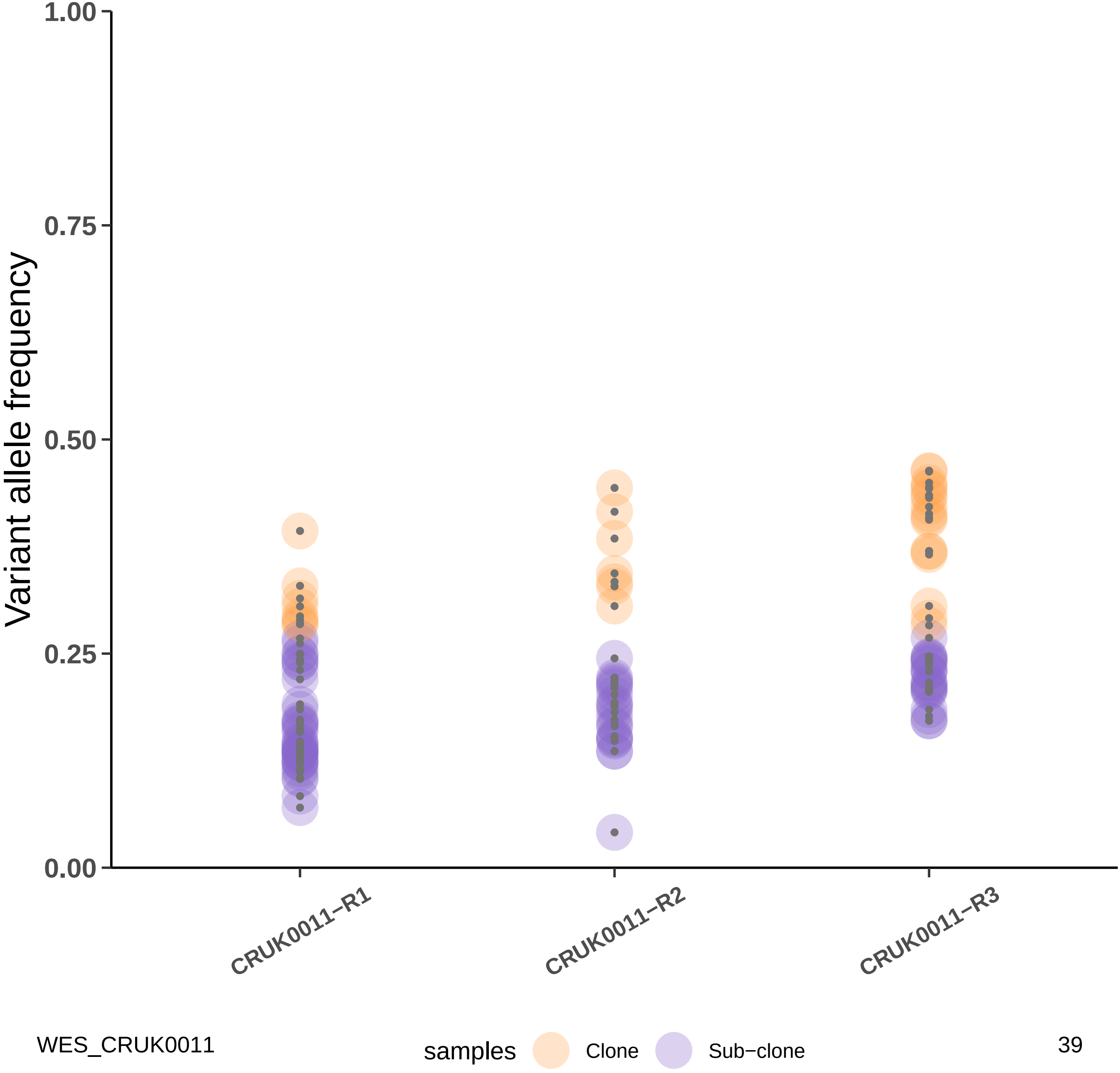

WES_CRUK0011

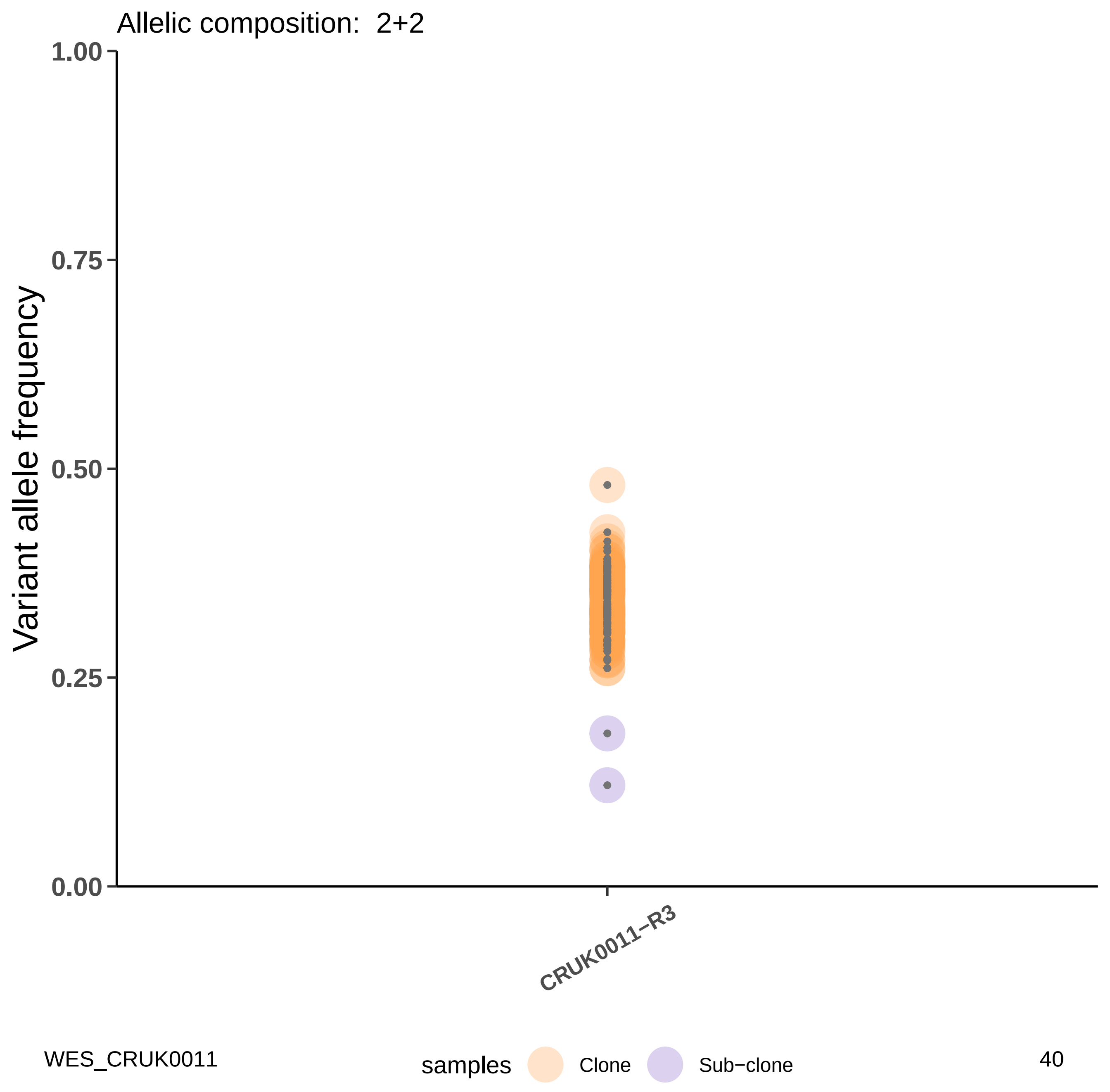

WES_CRUK0011

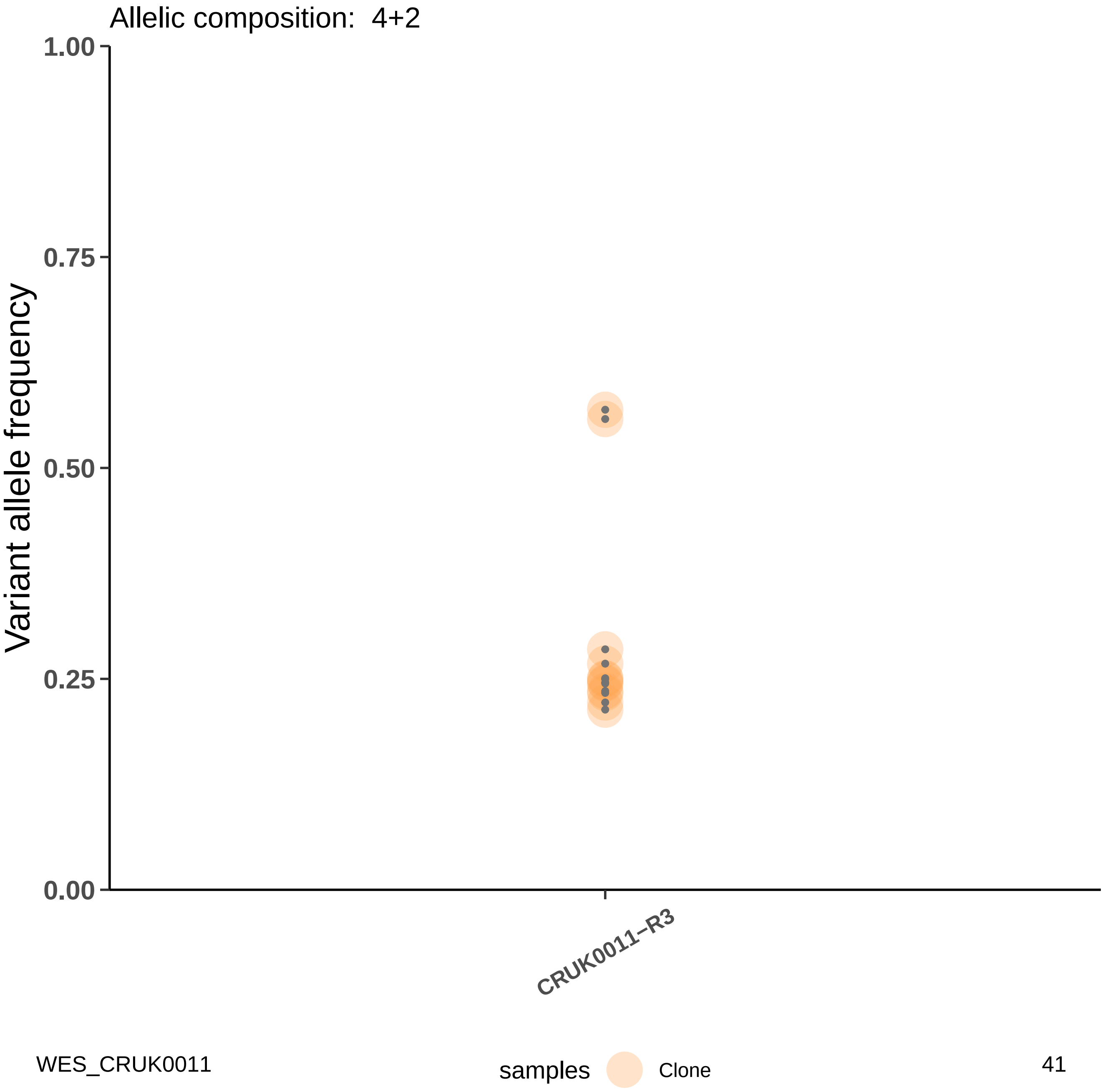

WES_CRUK0011

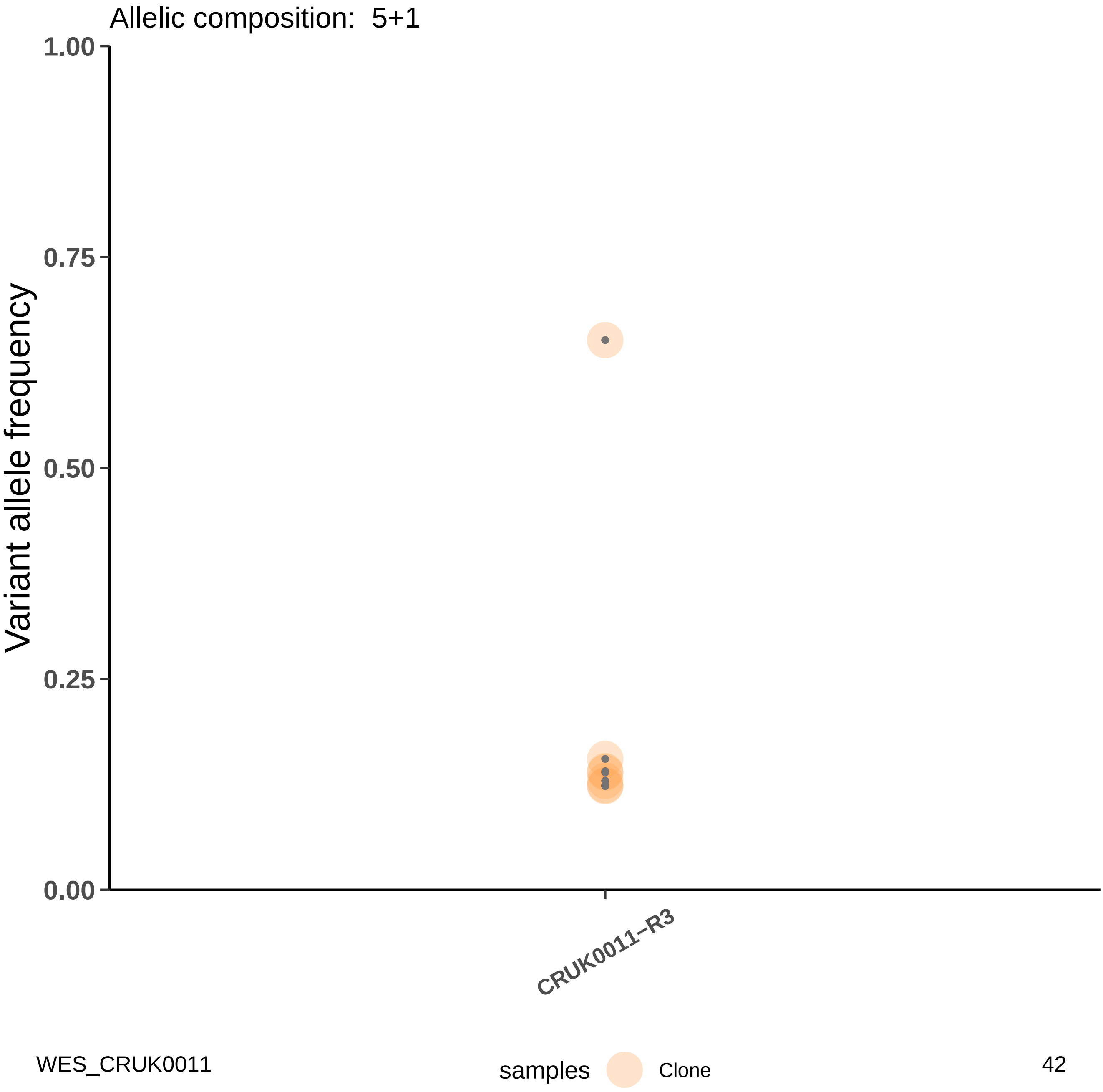
Allelic composition: 5+1
1.00
0.75
0.50
0.25
0.00
Variant allele frequency
CRUK0011−R3
samples
Clone

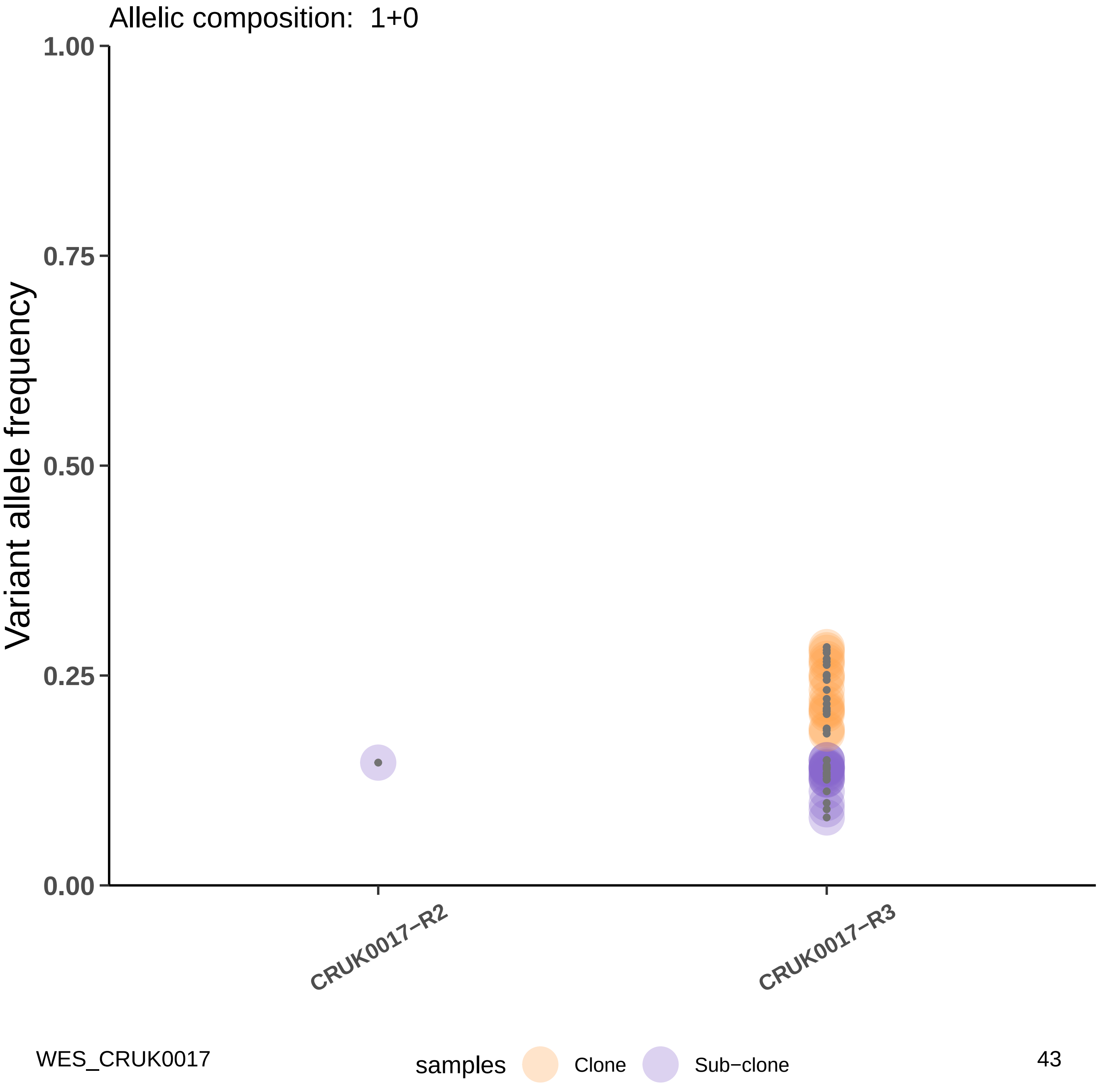

WES_CRUK0017

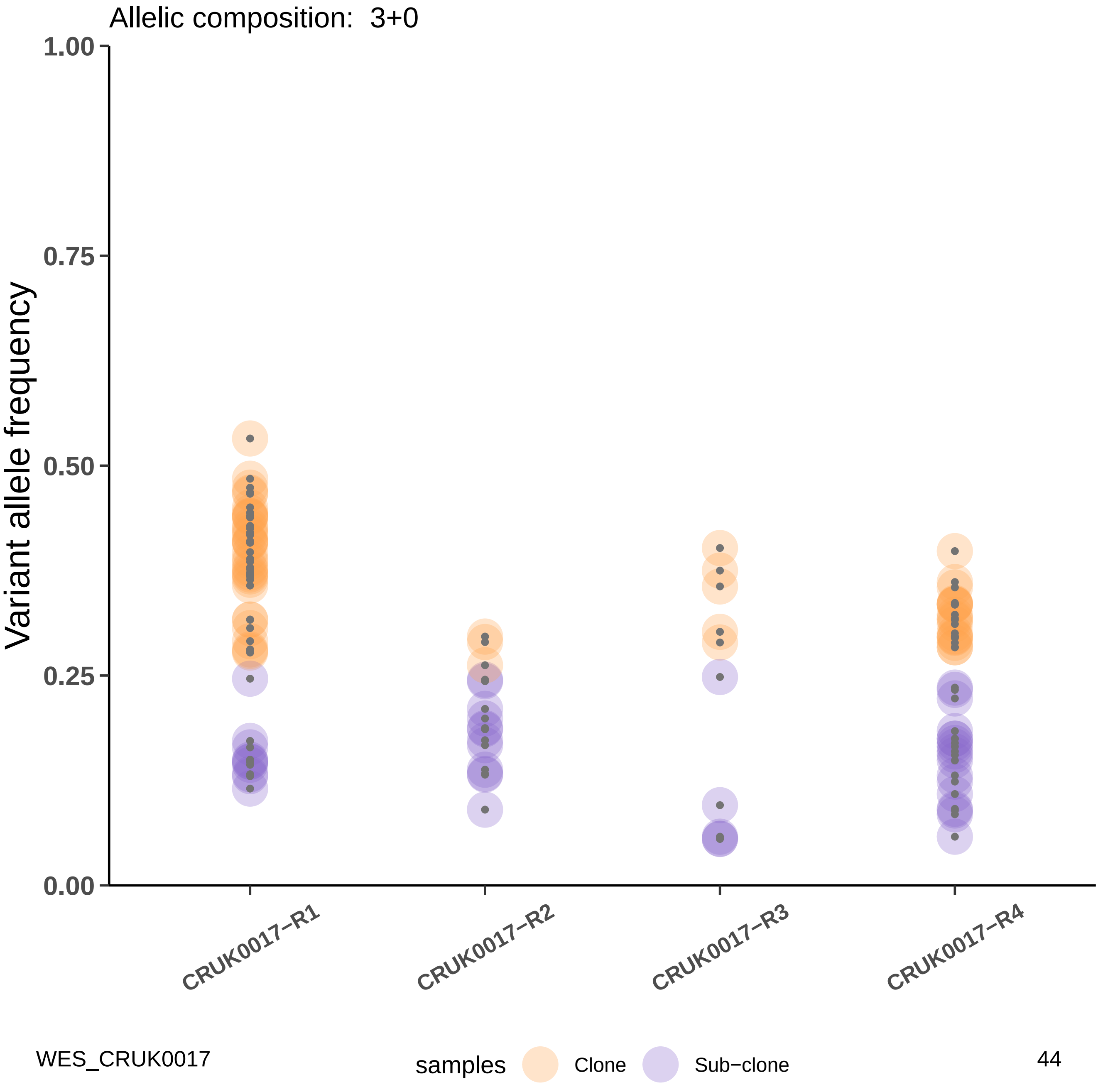

WES_CRUK0017

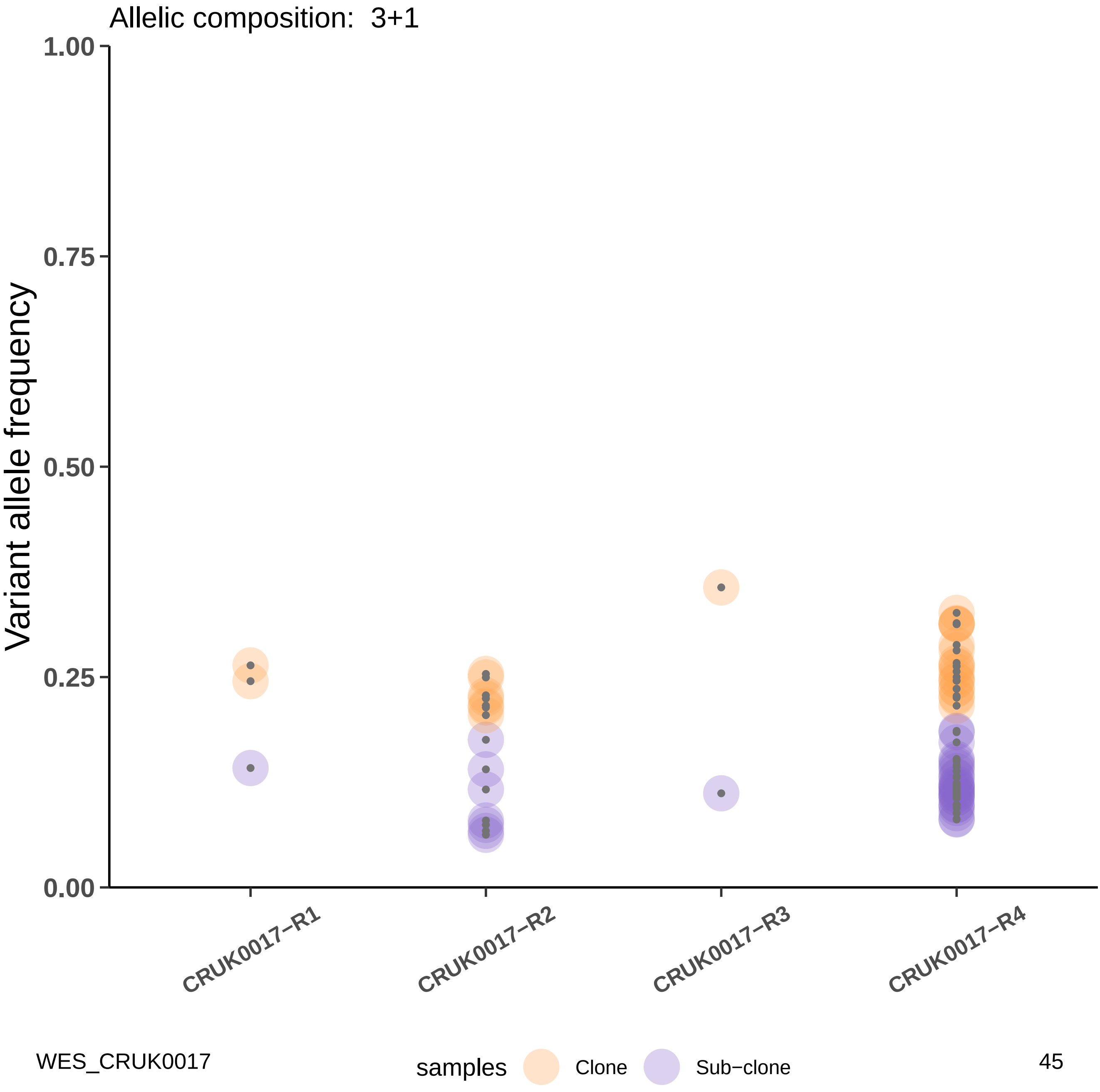

WES_CRUK0017

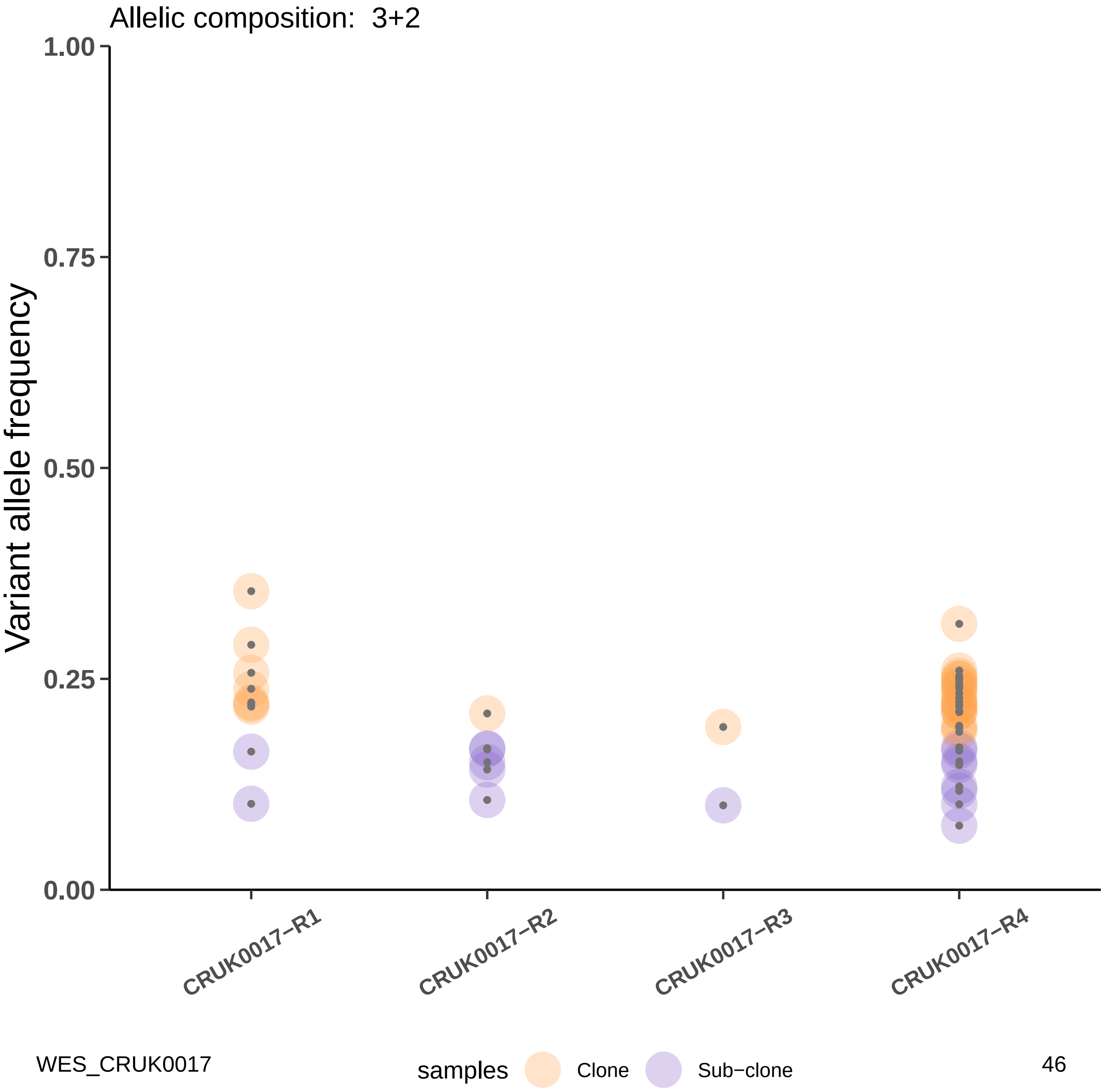

WES_CRUK0017

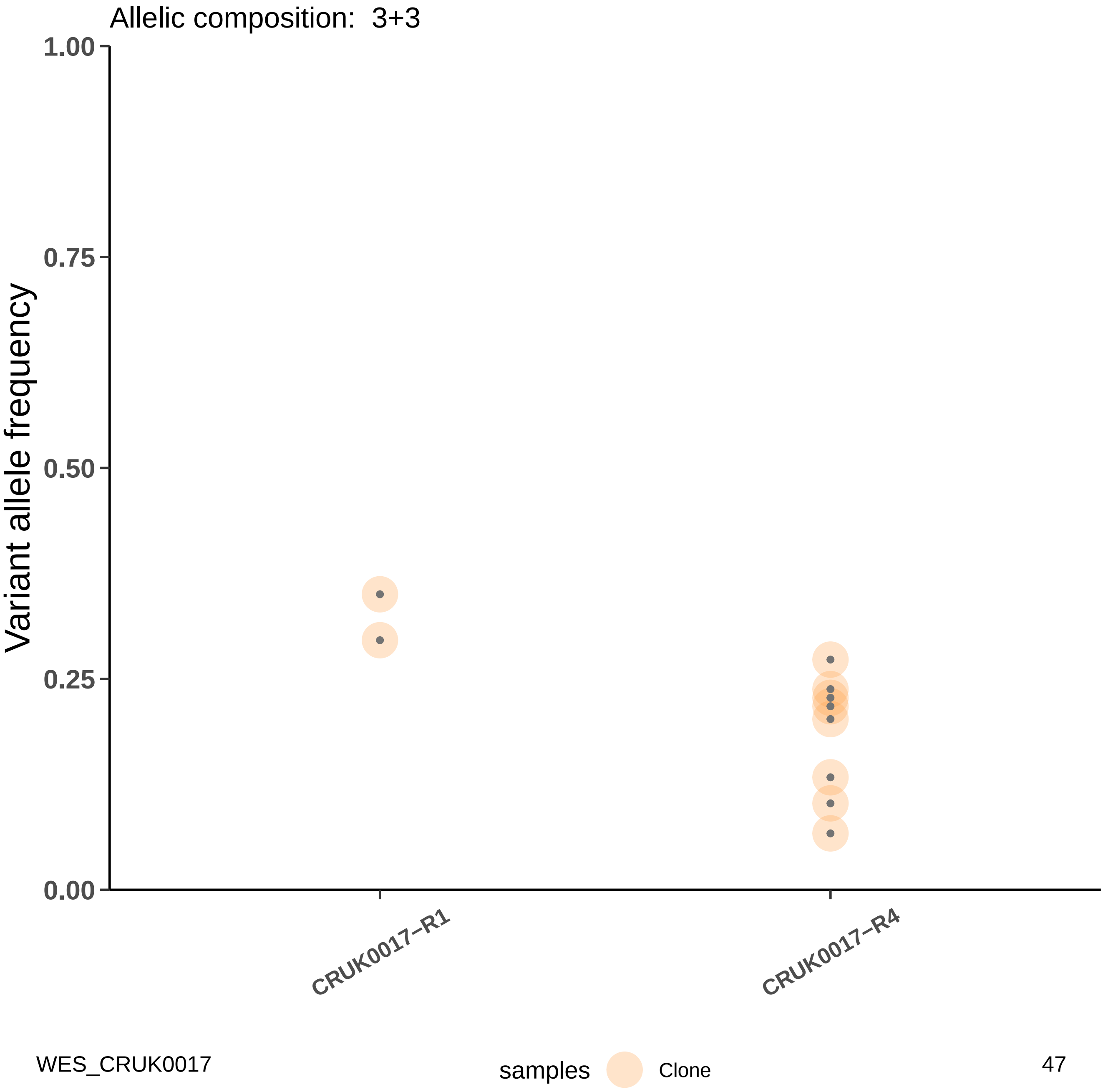

WES_CRUK0017

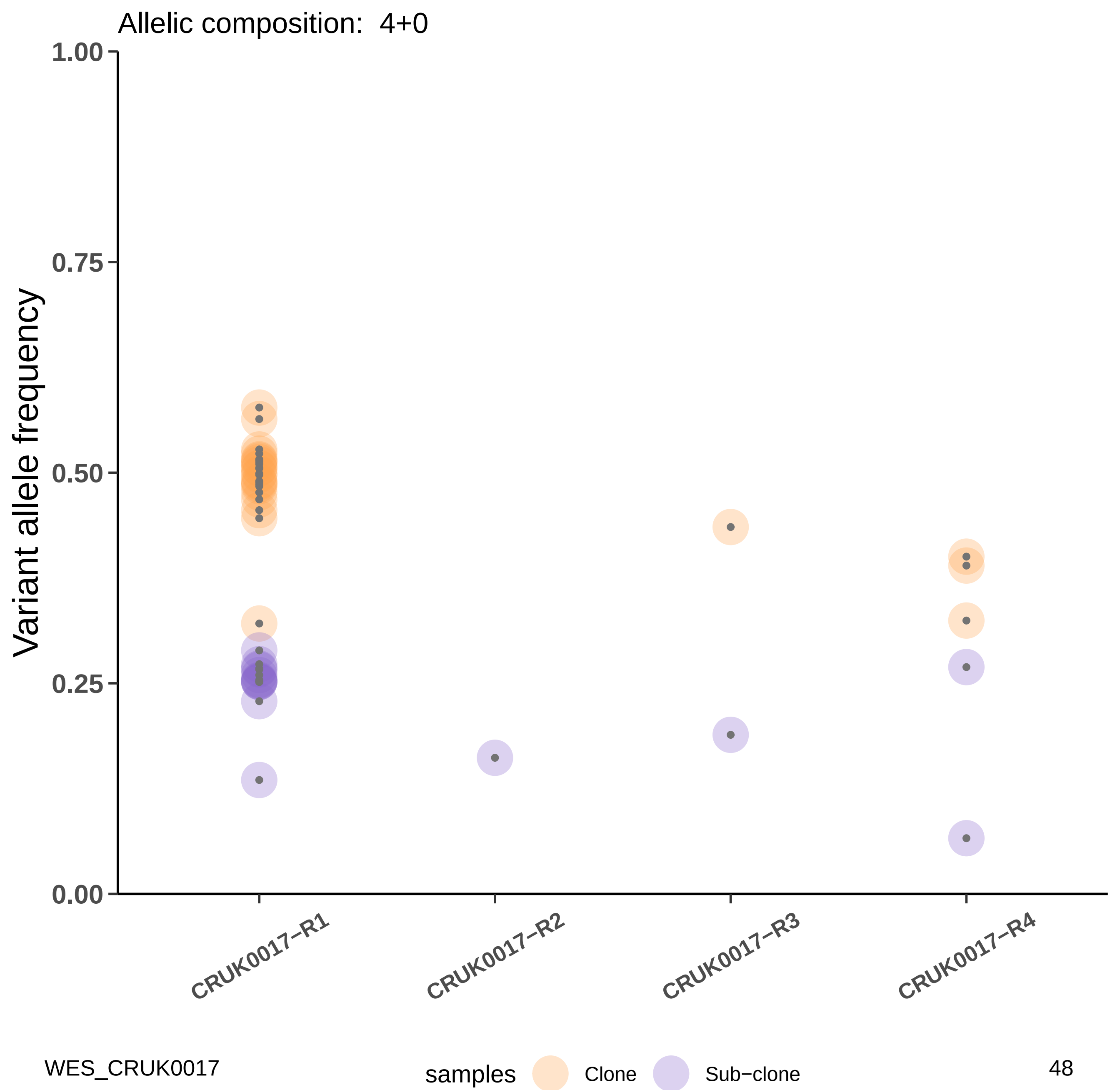

WES_CRUK0017

Allelic composition: 4+1

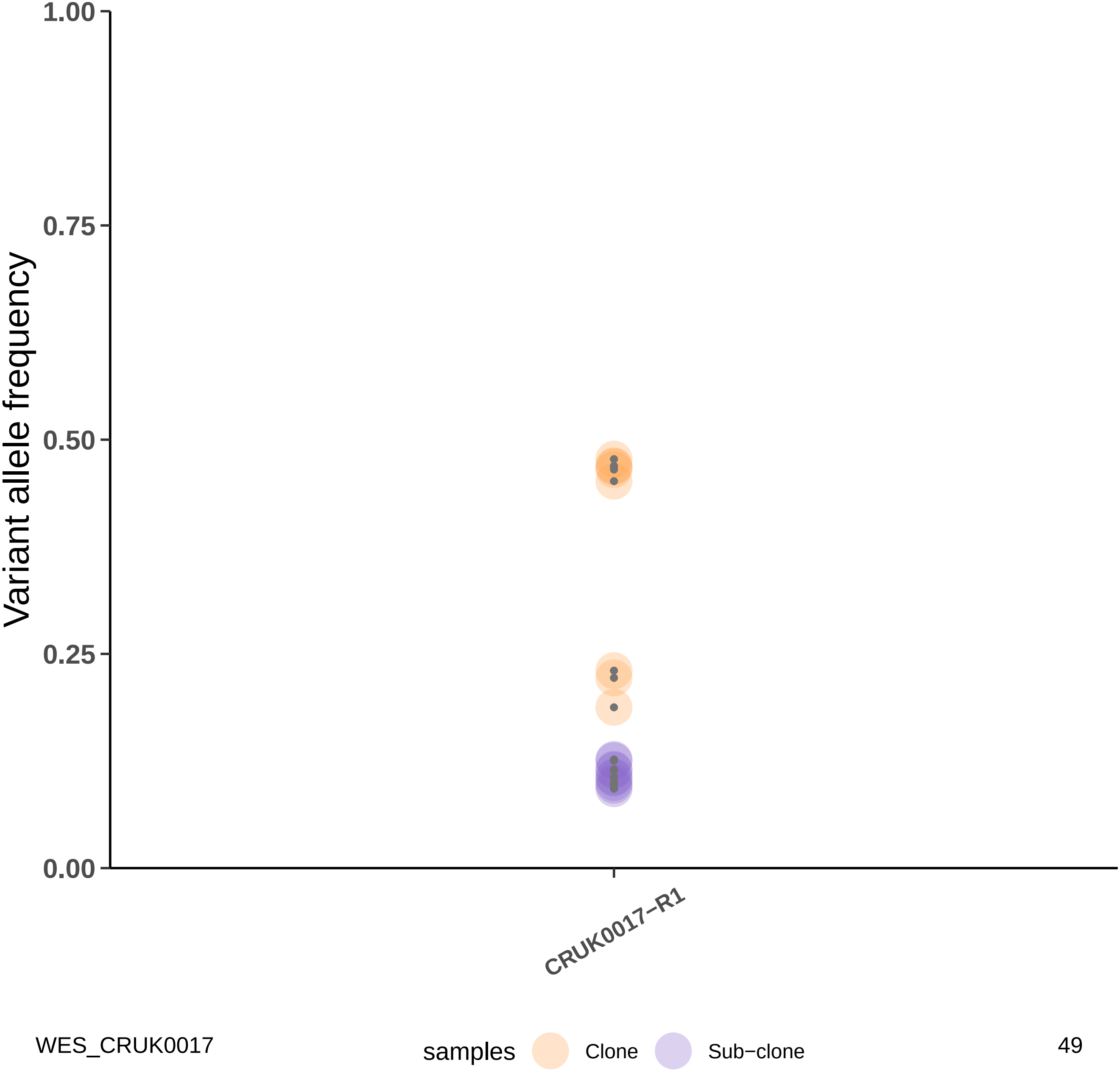

WES_CRUK0017

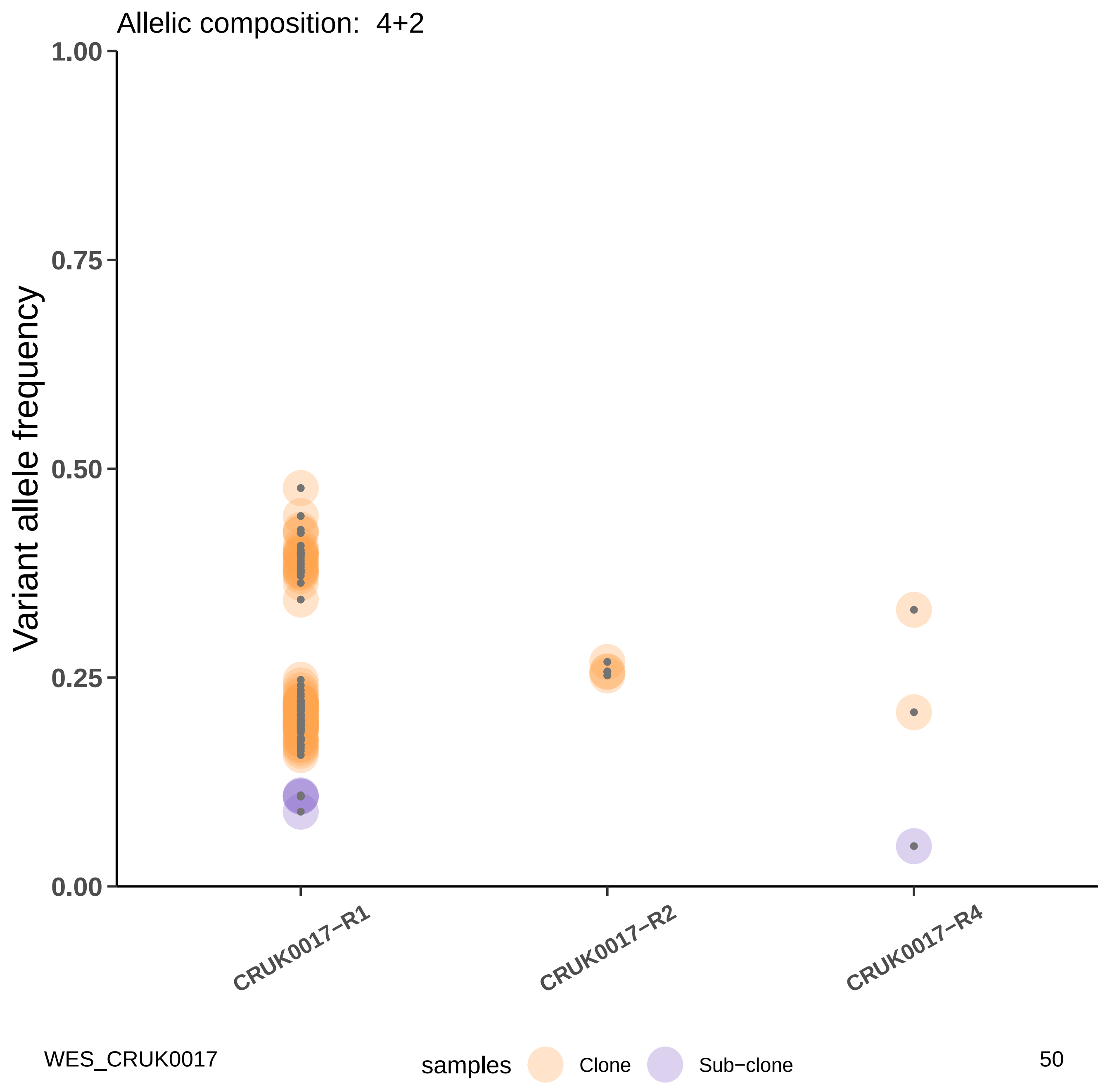

WES_CRUK0017

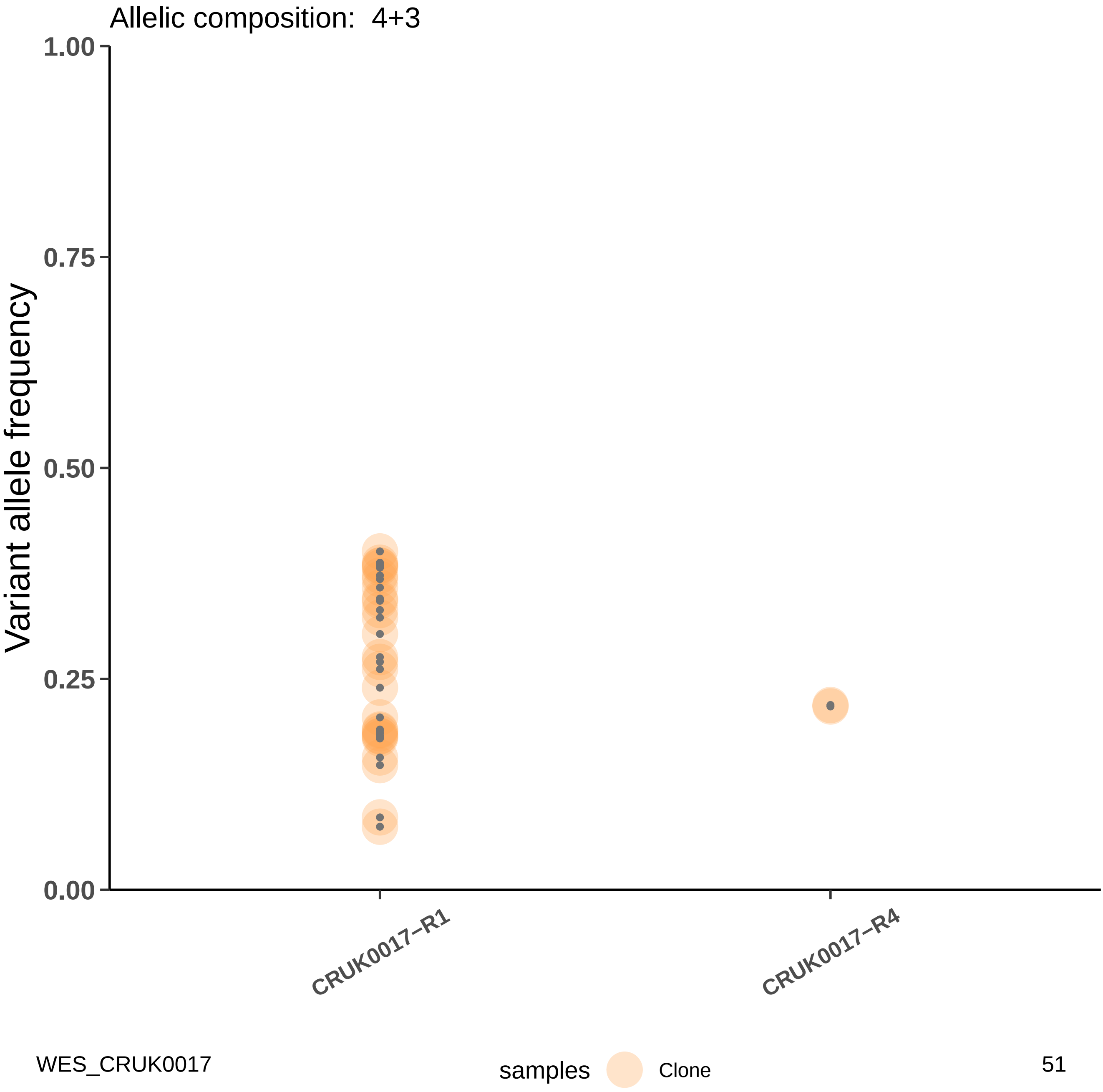
Allelic composition: 4+3
Variant allele frequency
1.00
0.75
0.50
0.25
0.00
CRUK0017−R1
CRUK0017−R4
samples
Clone

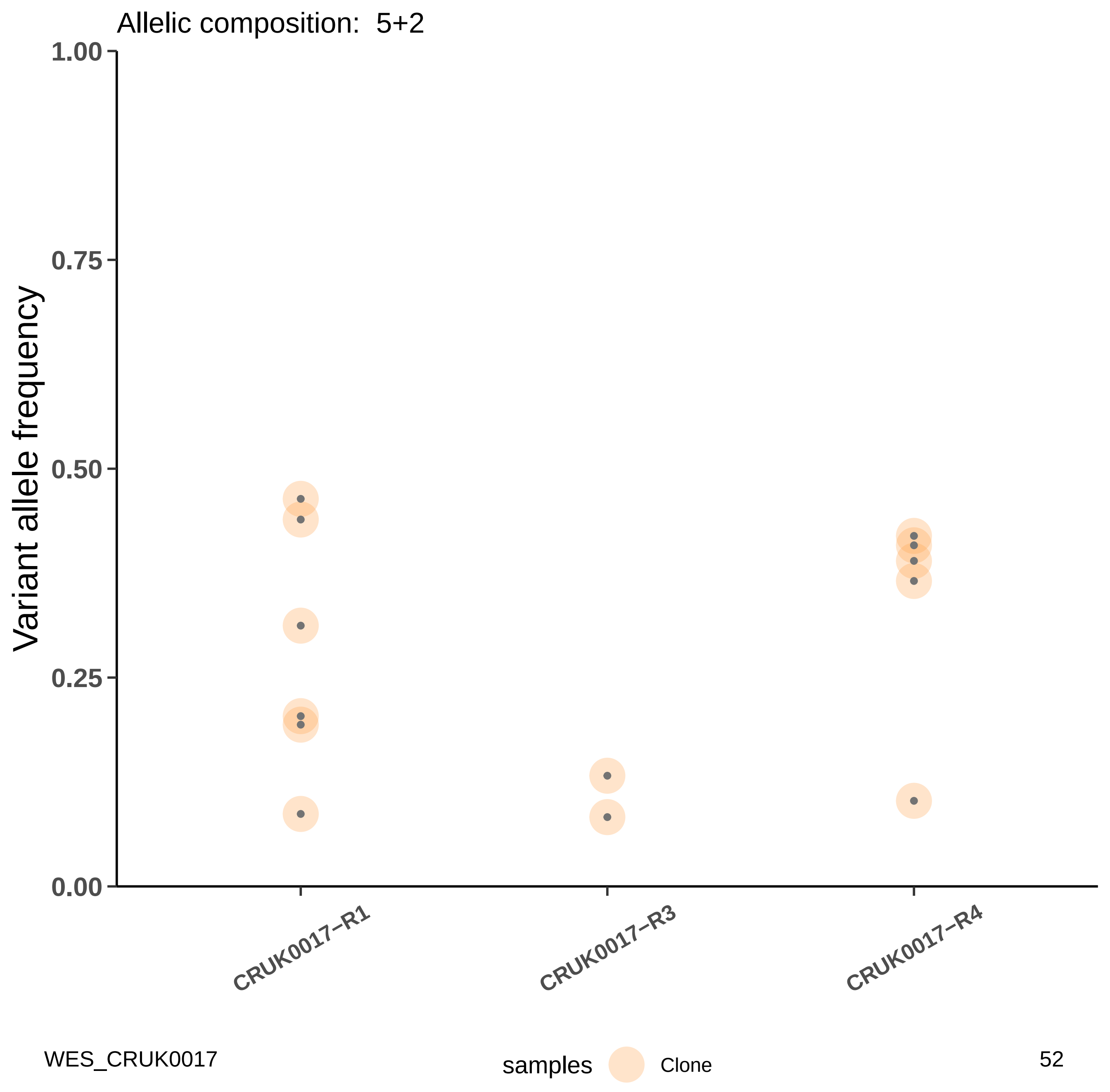

WES_CRUK0017

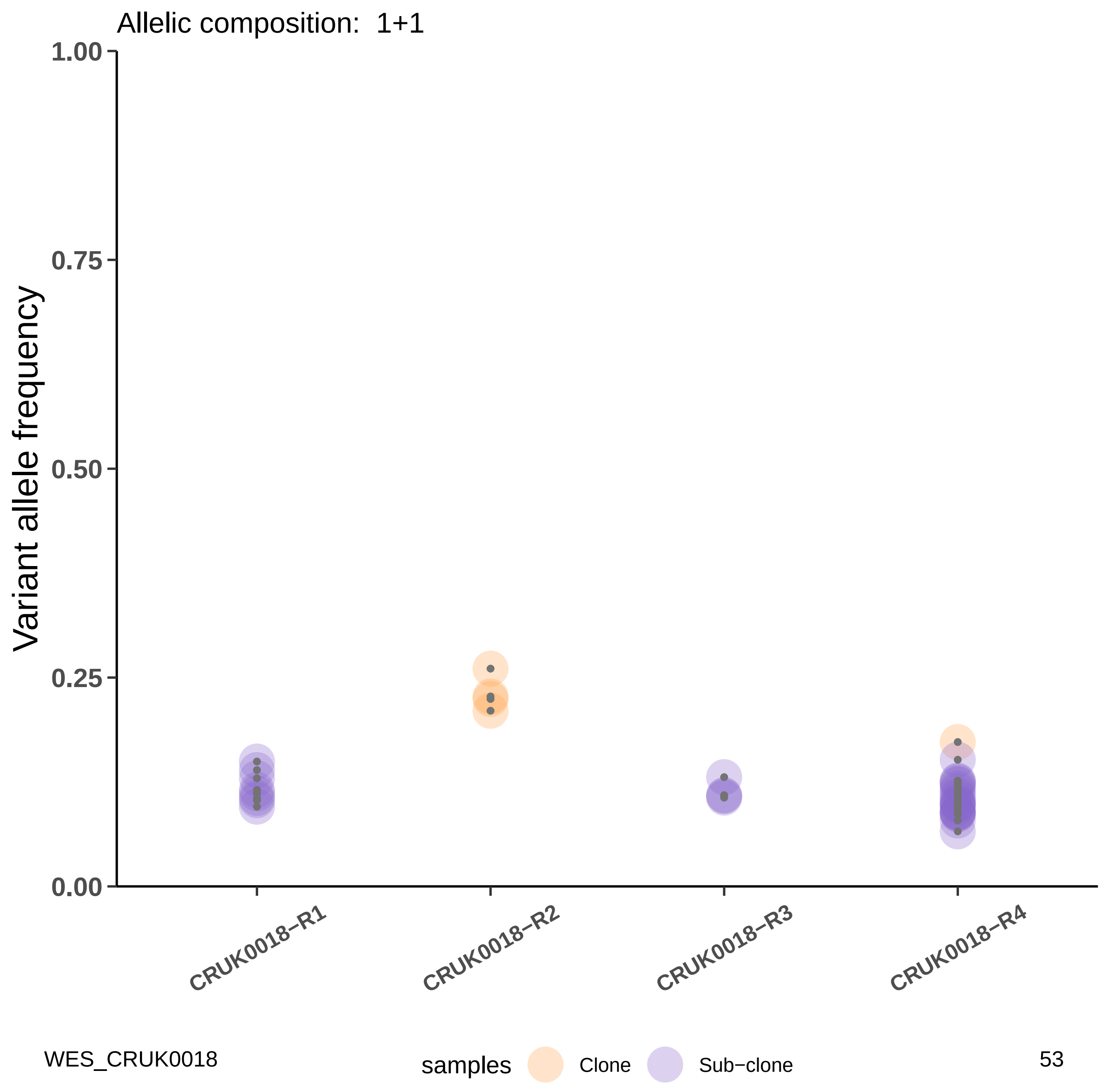

Allelic composition: 1+1
Variant allele frequency
1.00
0.75
0.50
0.25
0.00
CRUK0018−R1
CRUK0018−R2
CRUK0018−R3
CRUK0018−R4
samples
Clone
Sub−clone

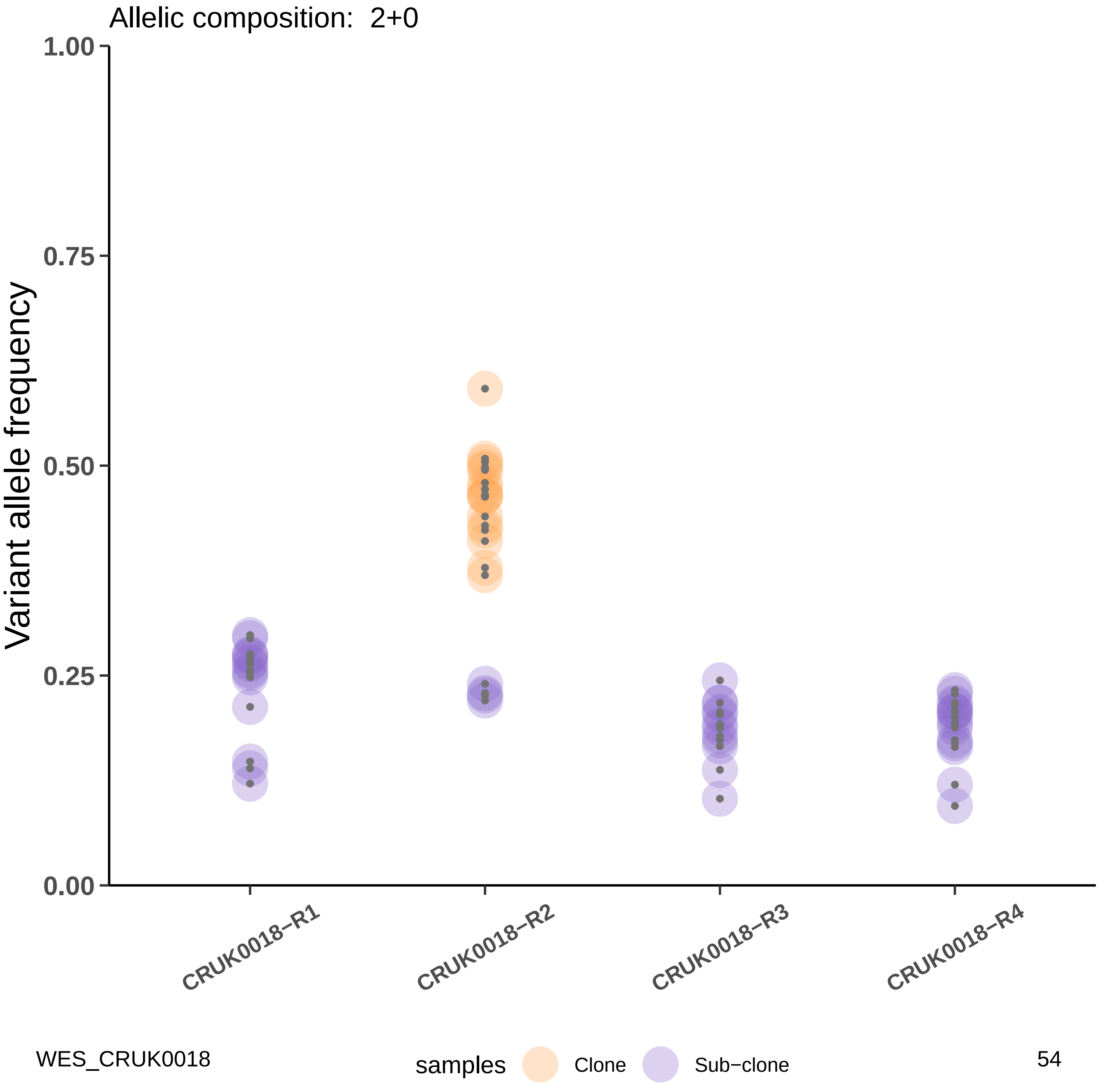

WES_CRUK0018

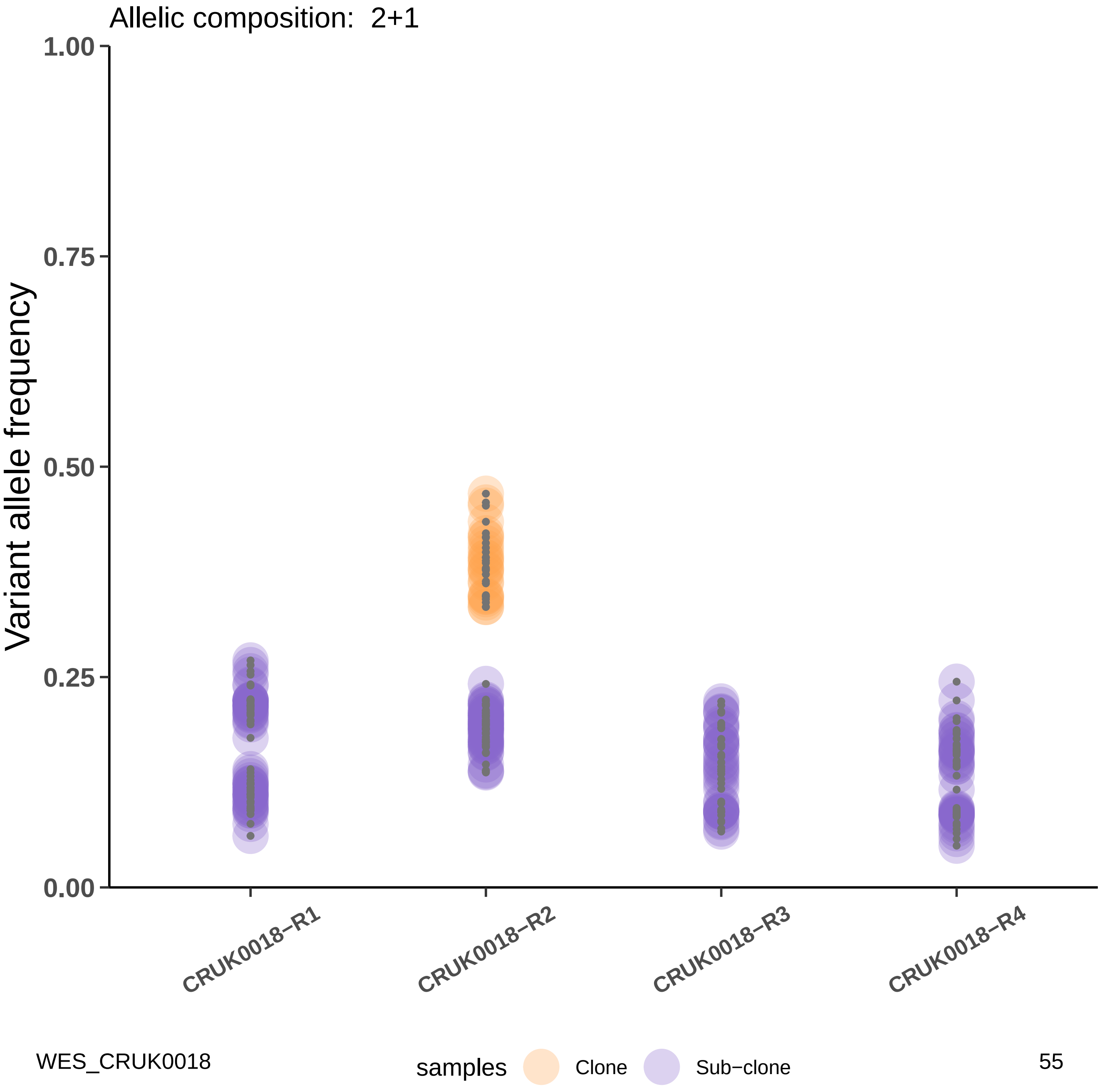

WES_CRUK0018

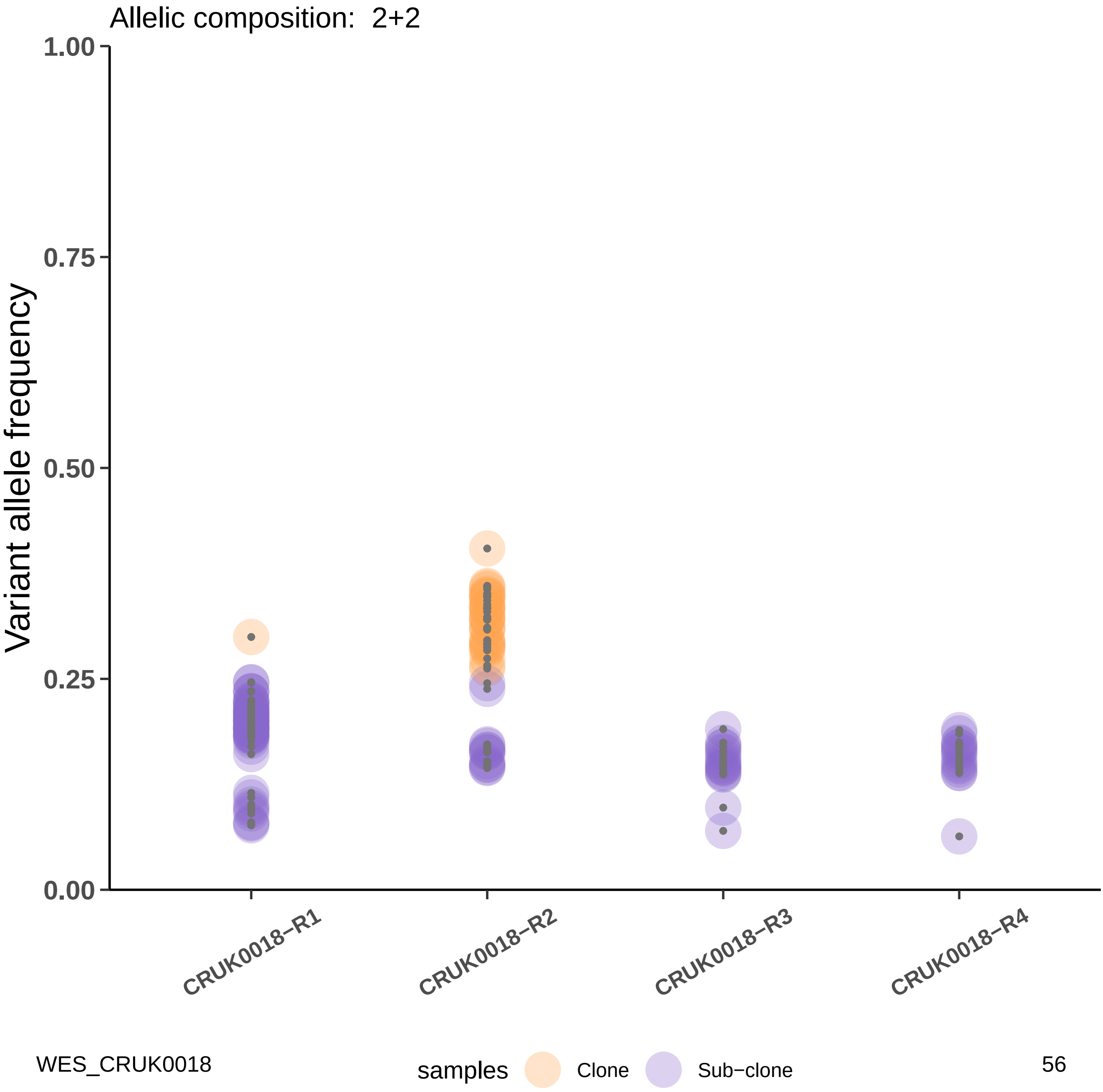

WES_CRUK0018

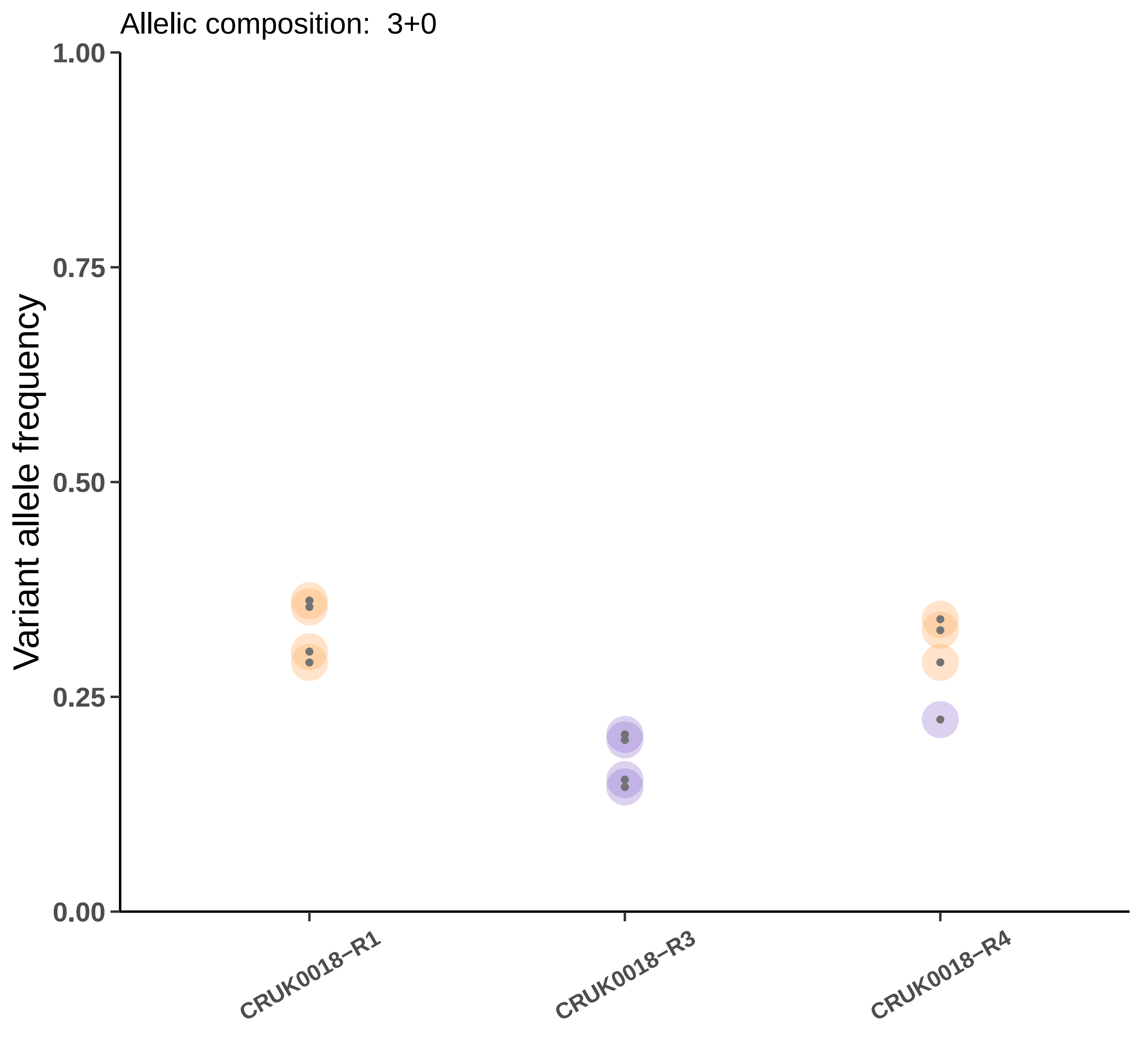

WES_CRUK0018

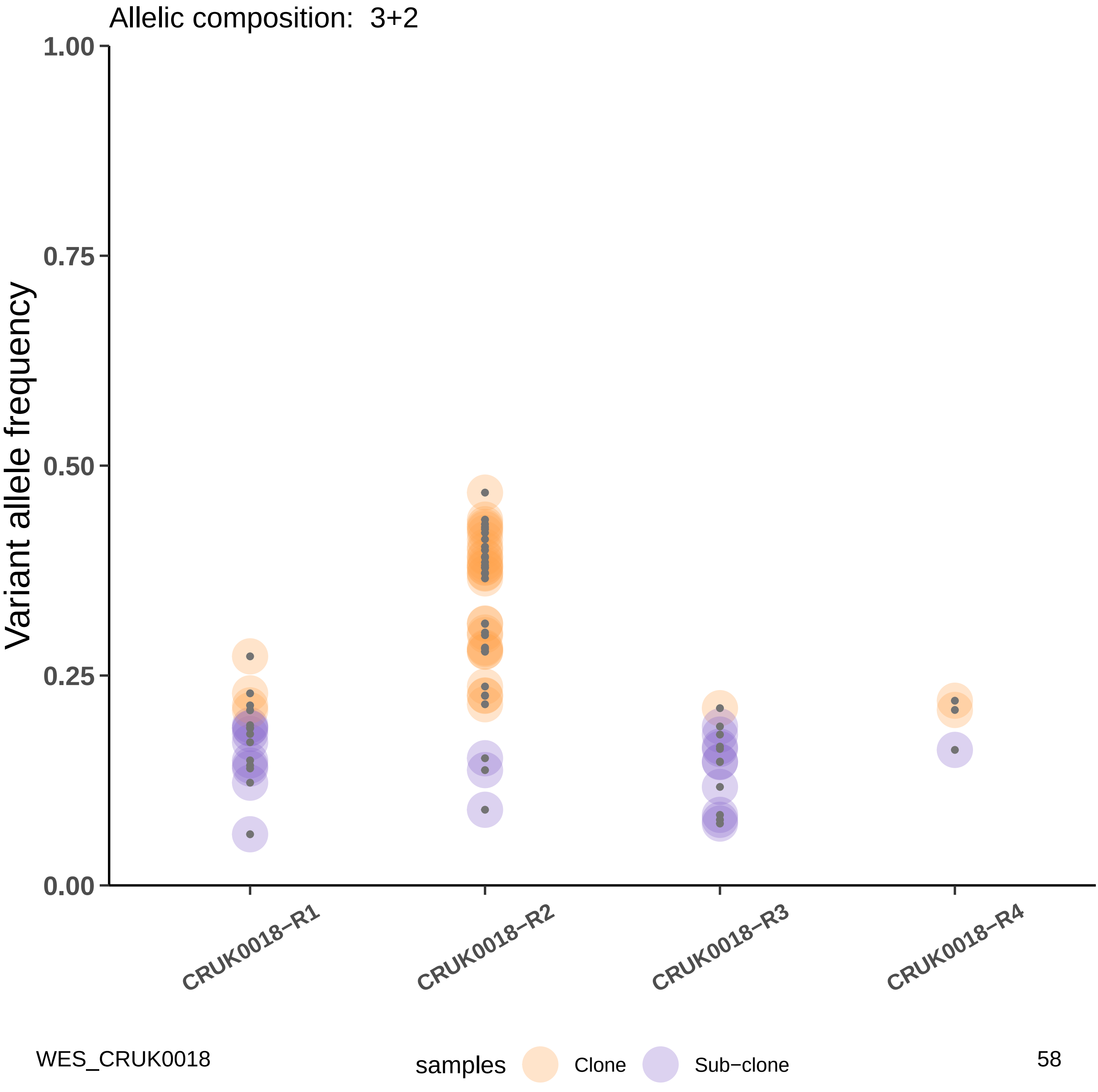

WES_CRUK0018

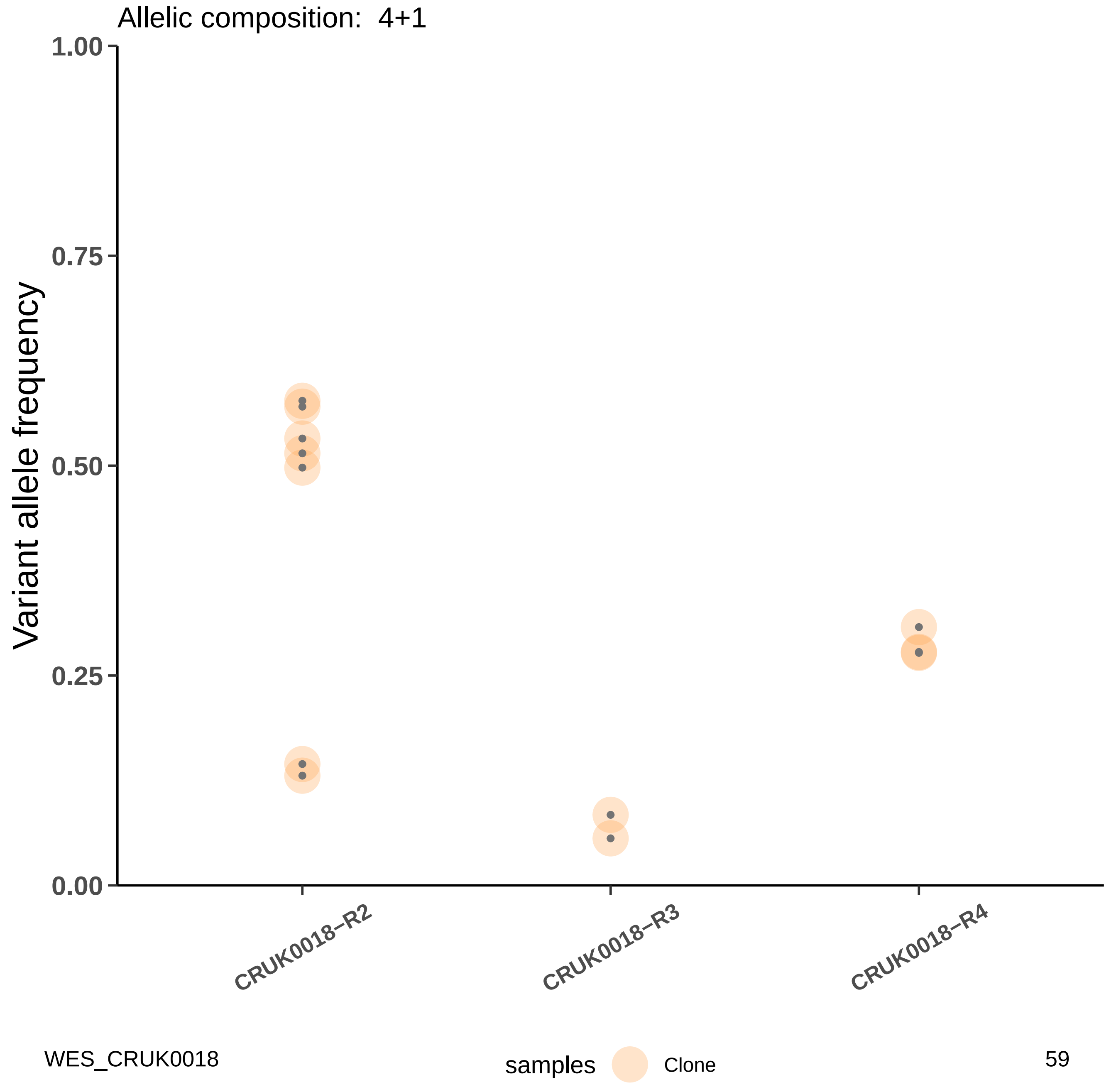

WES_CRUK0018

Allelic composition: 4+2

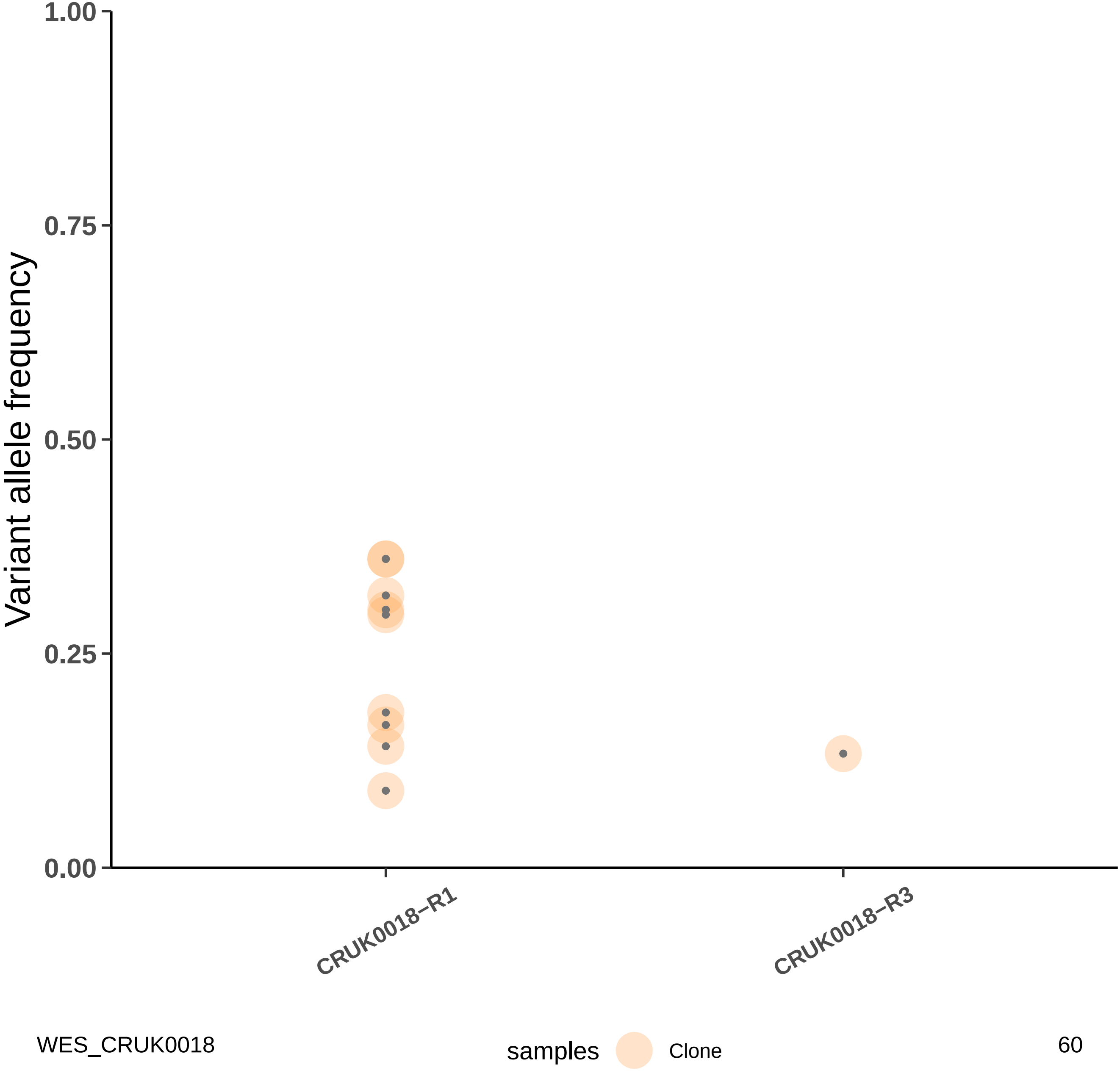

WES_CRUK0018

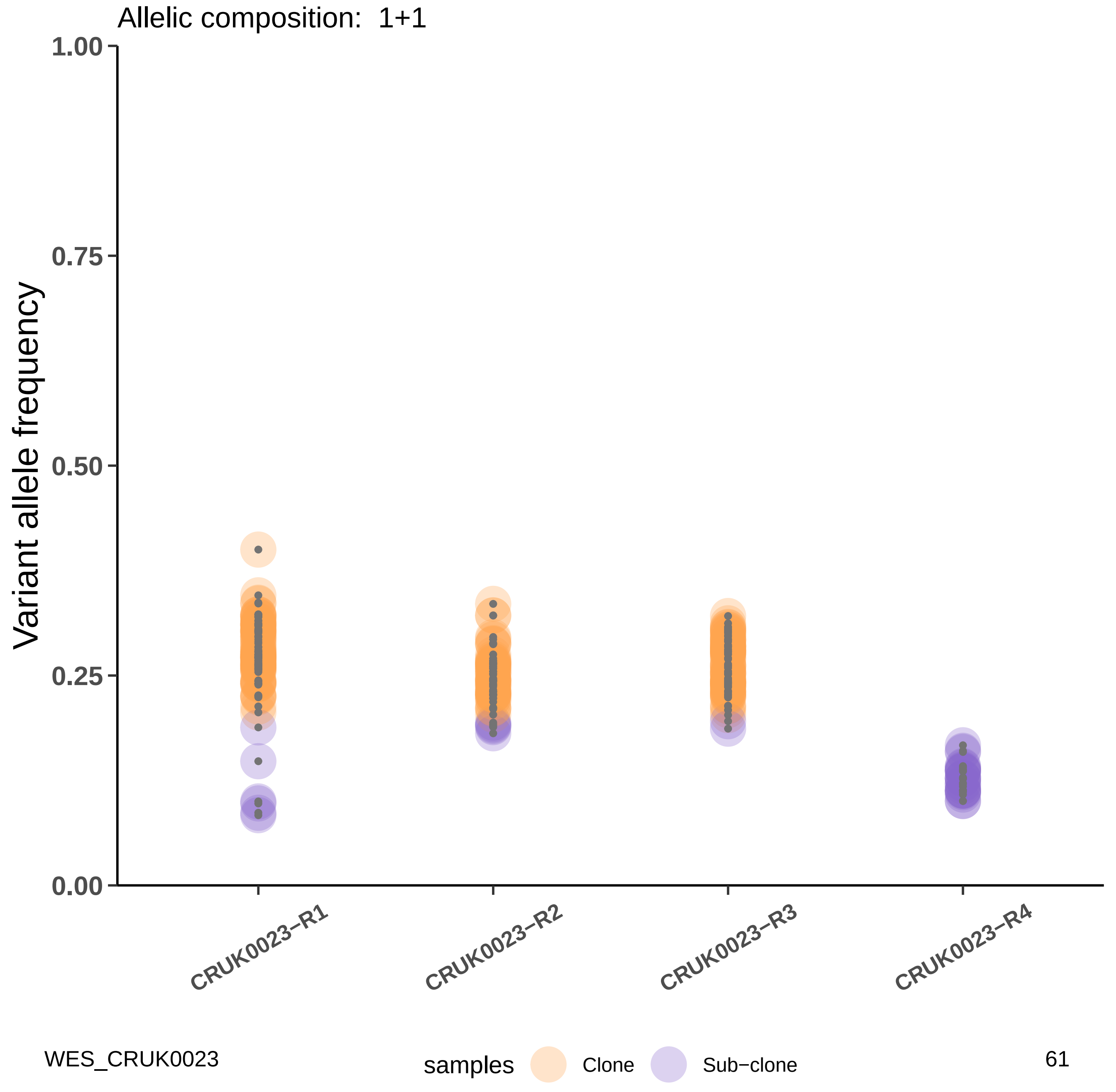

WES_CRUK0023

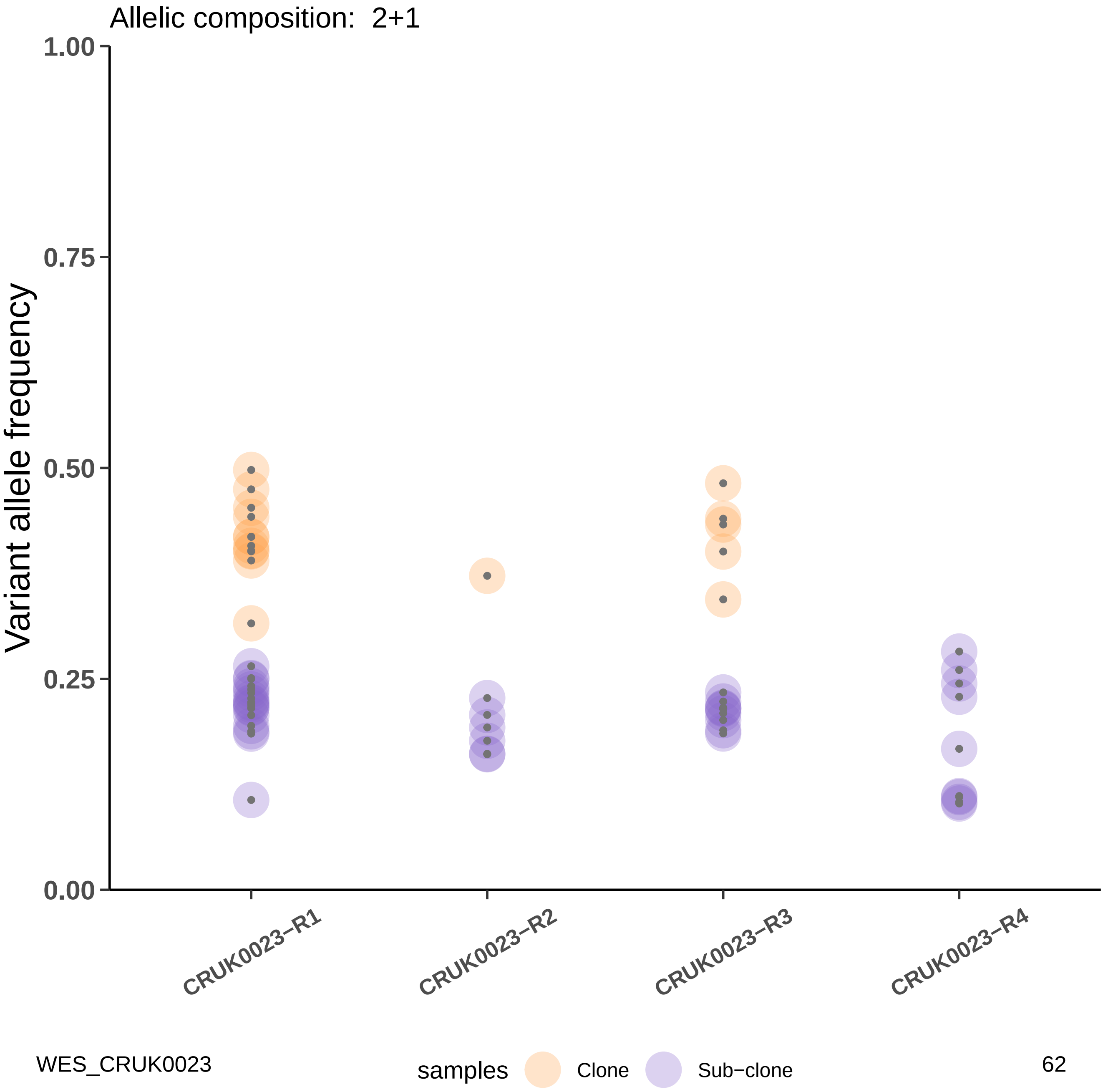

WES_CRUK0023

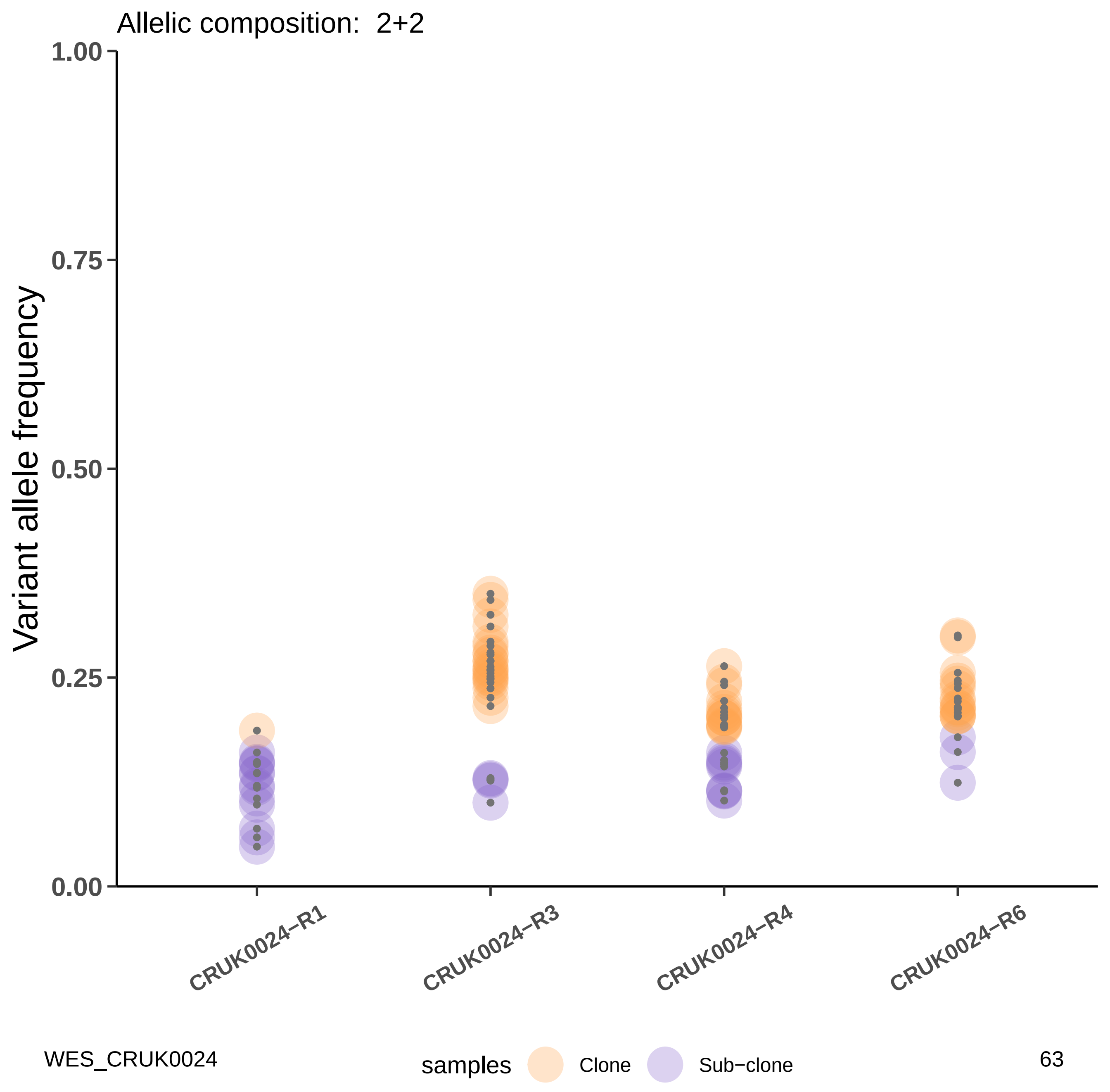

WES_CRUK0024

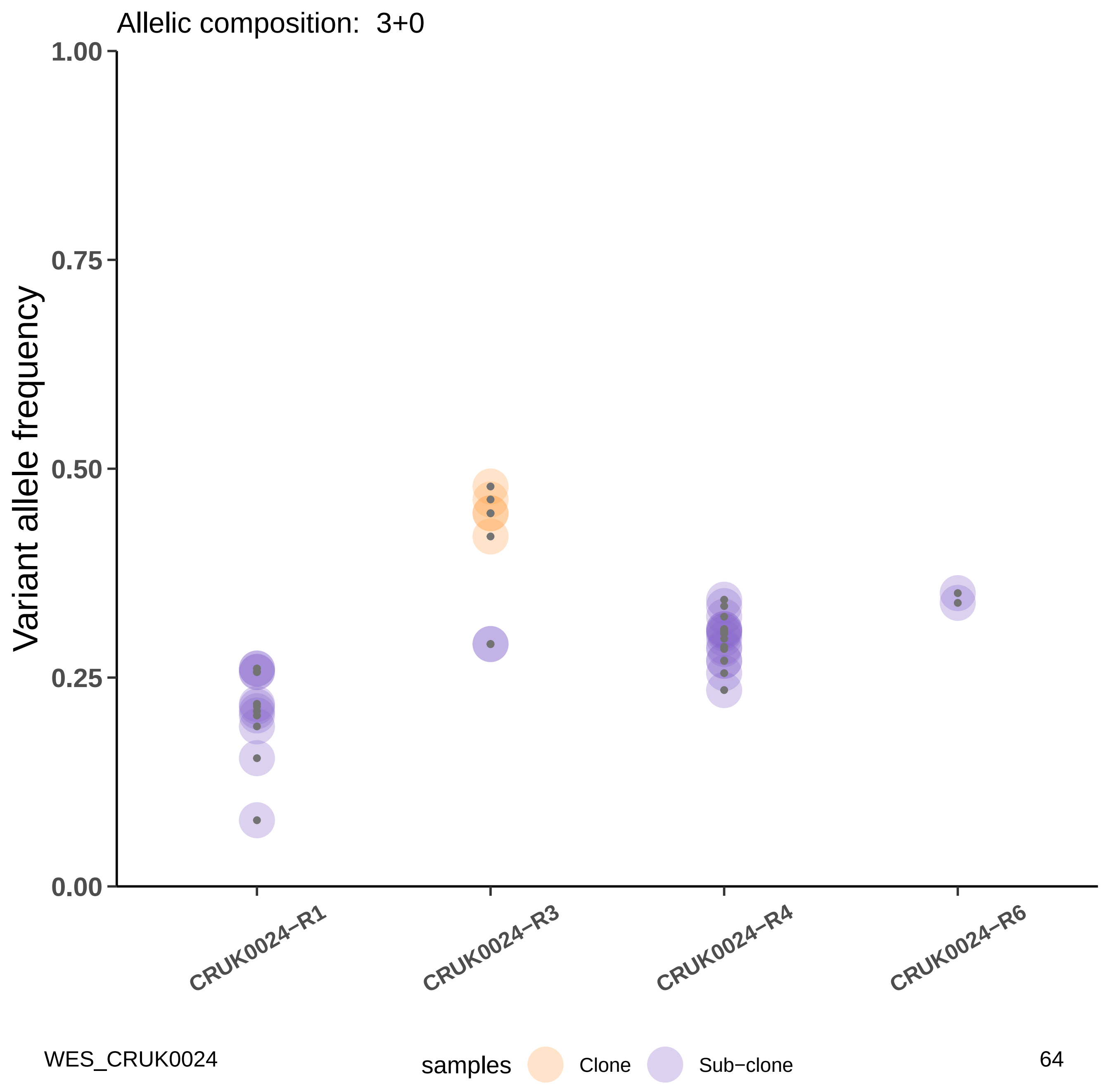

WES_CRUK0024

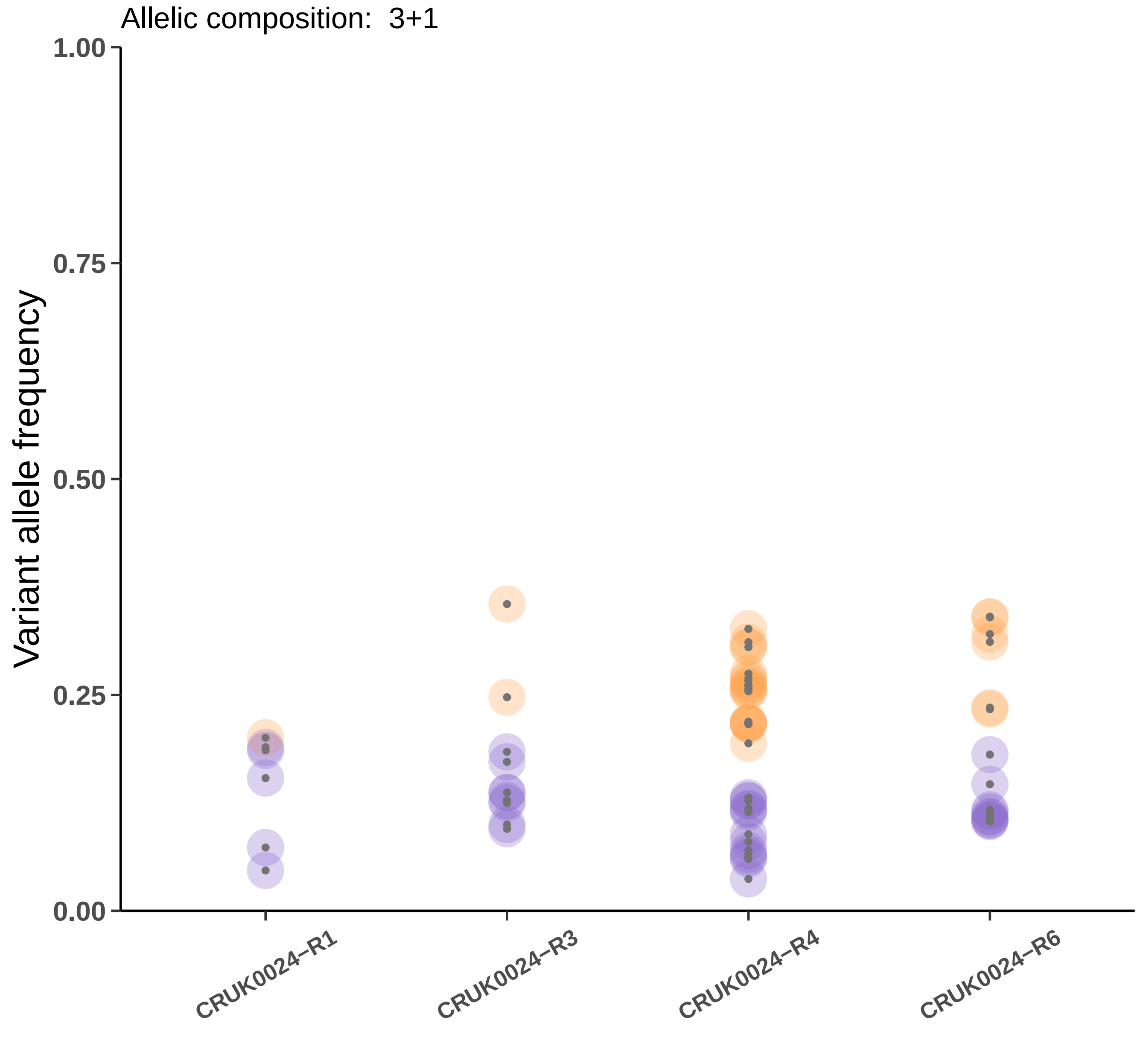

WES_CRUK0024

Allelic composition: 3+2

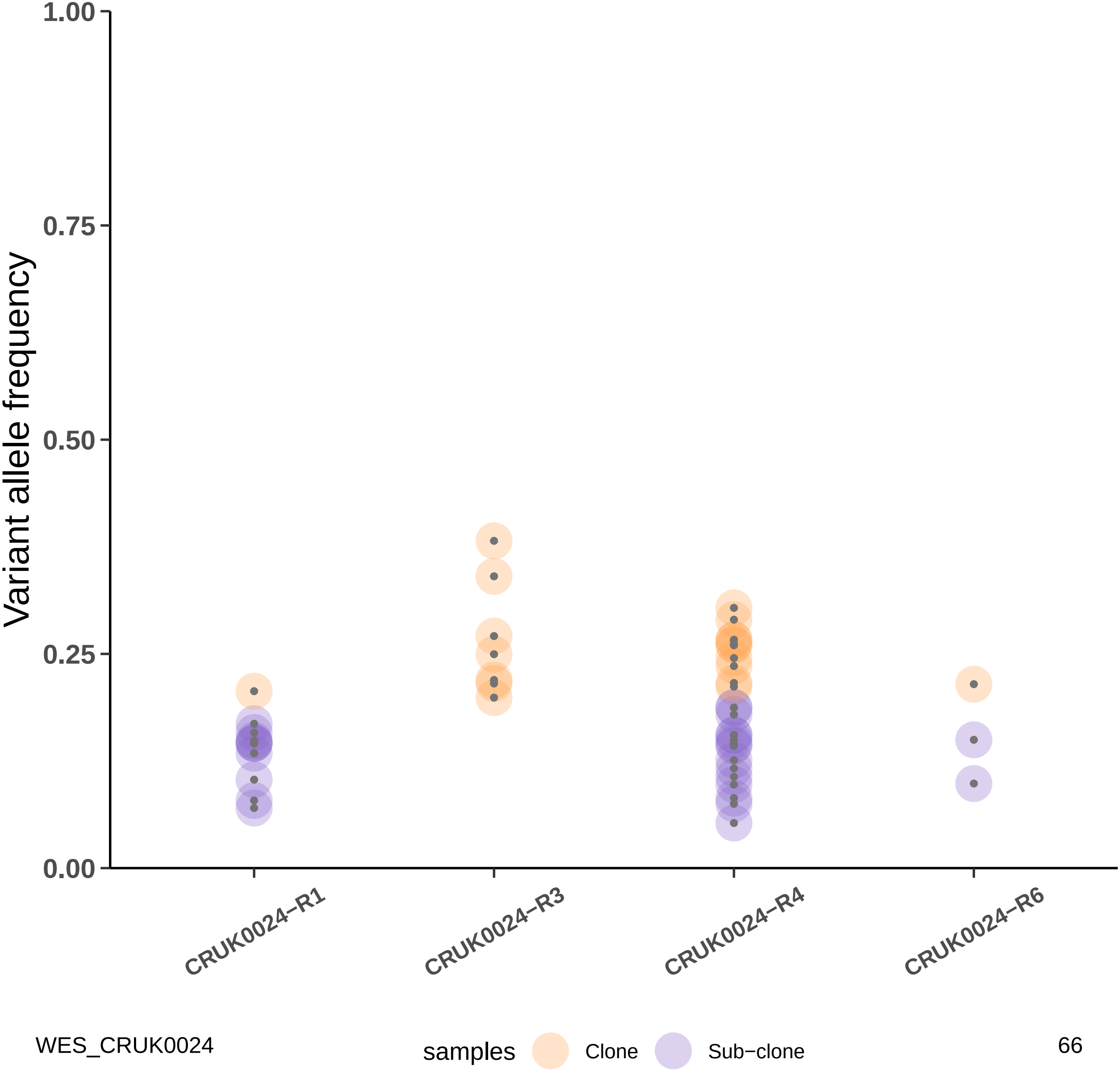

WES_CRUK0024

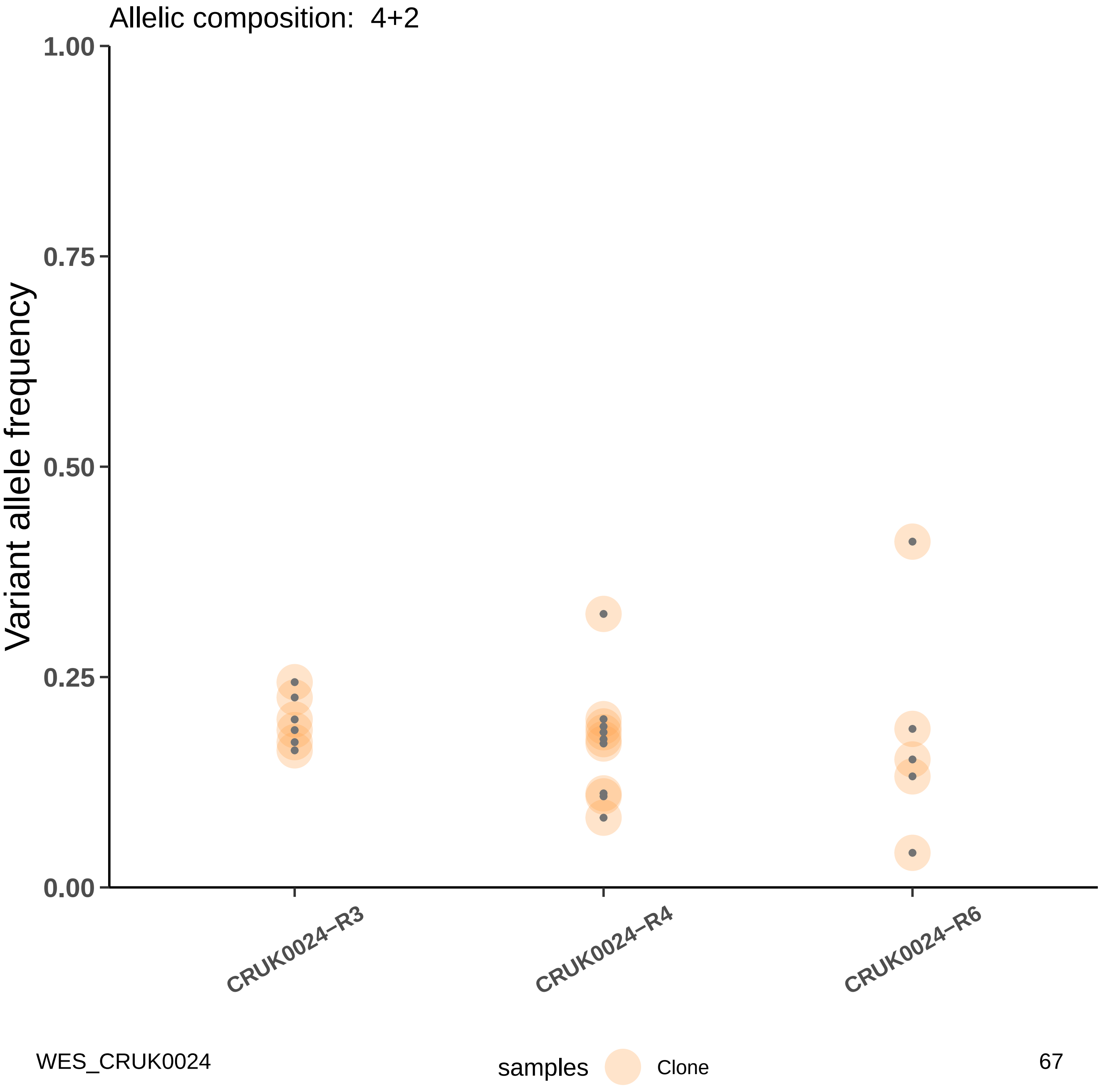

Allelic composition: 4+2
Variant allele frequency
1.00
0.75
0.50
0.25
0.00
CRUK0024−R3
CRUK0024−R4
CRUK0024−R6
samples
Clone

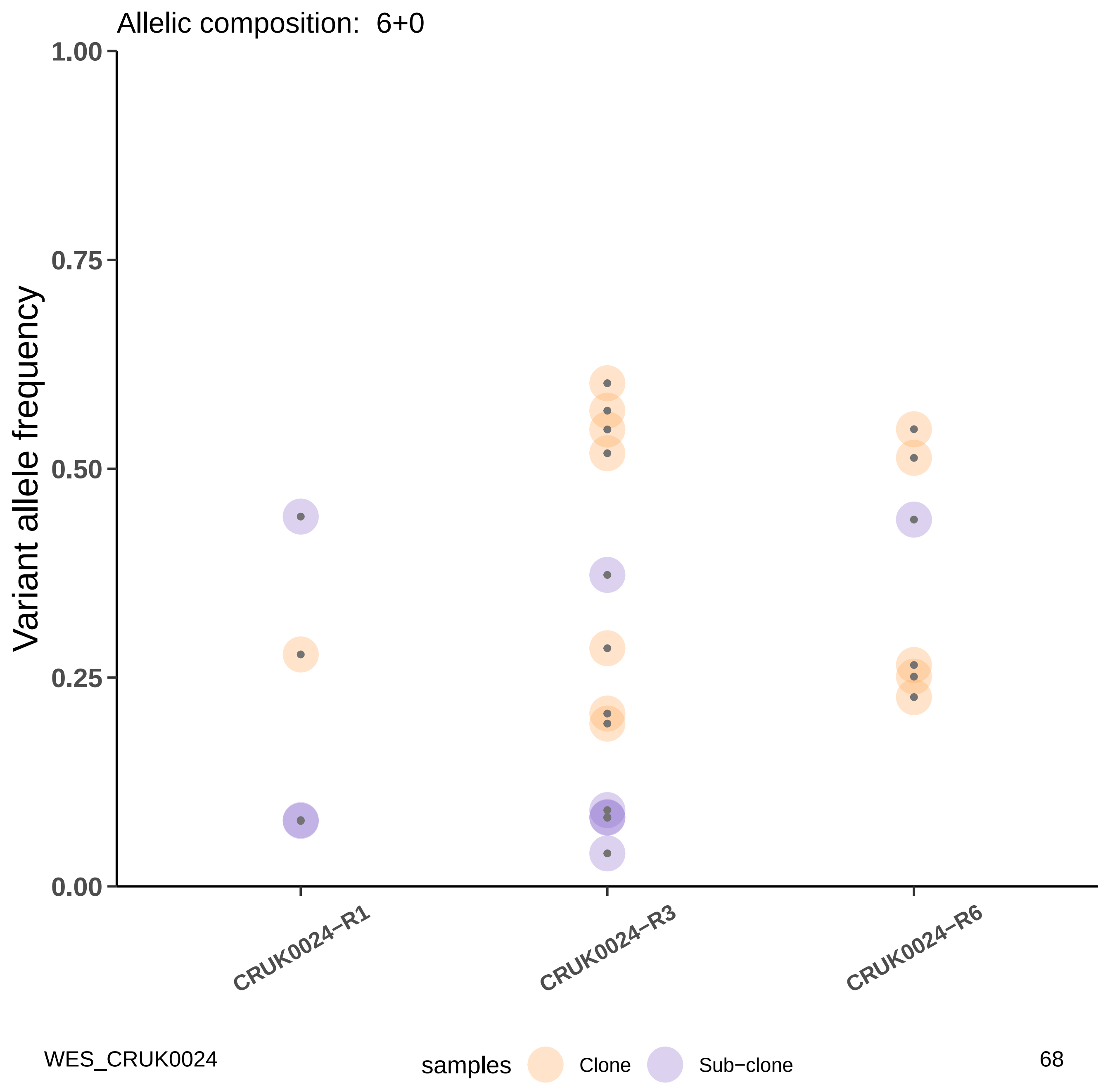

WES_CRUK0024

Allelic composition: 2+0

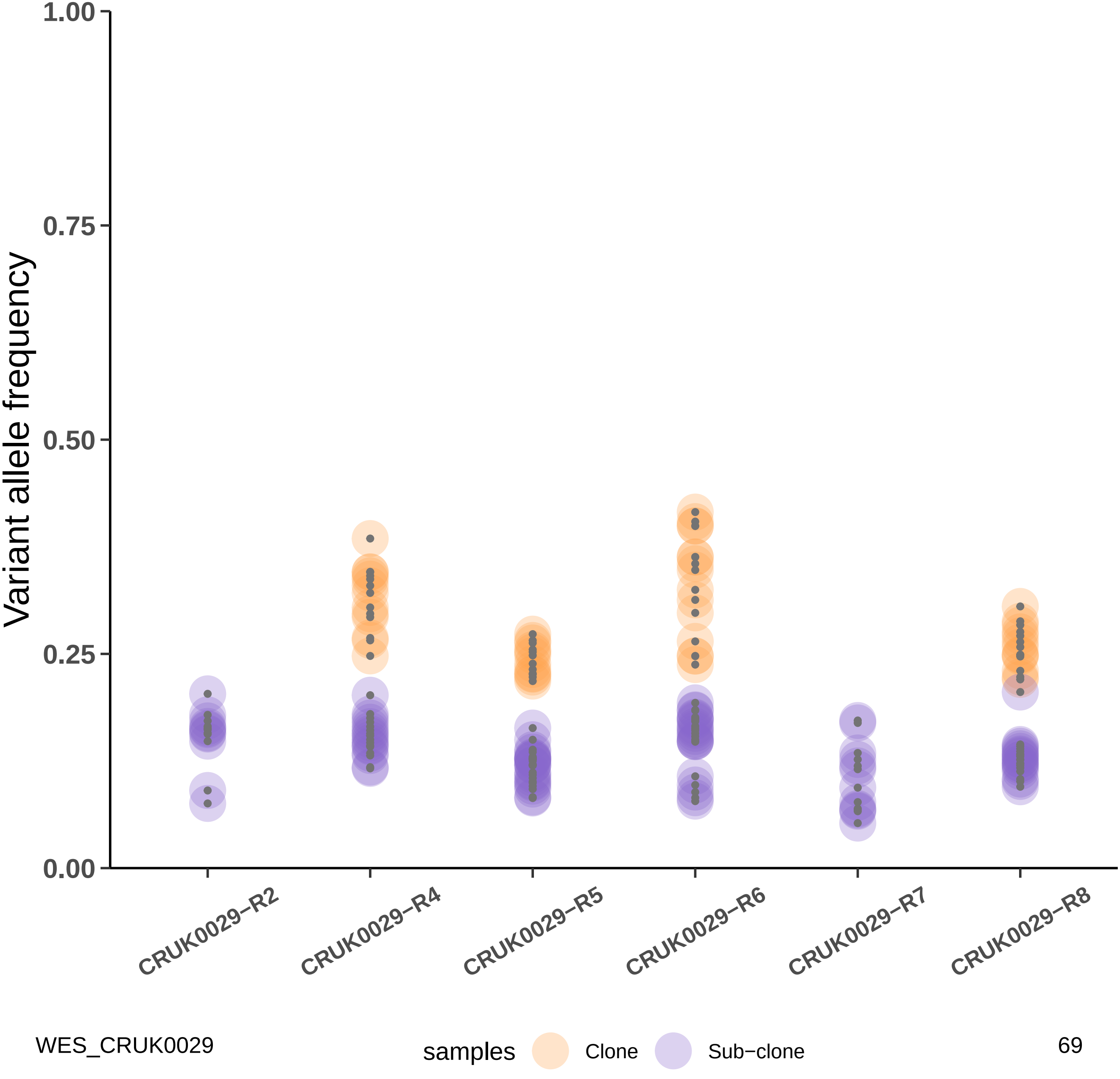

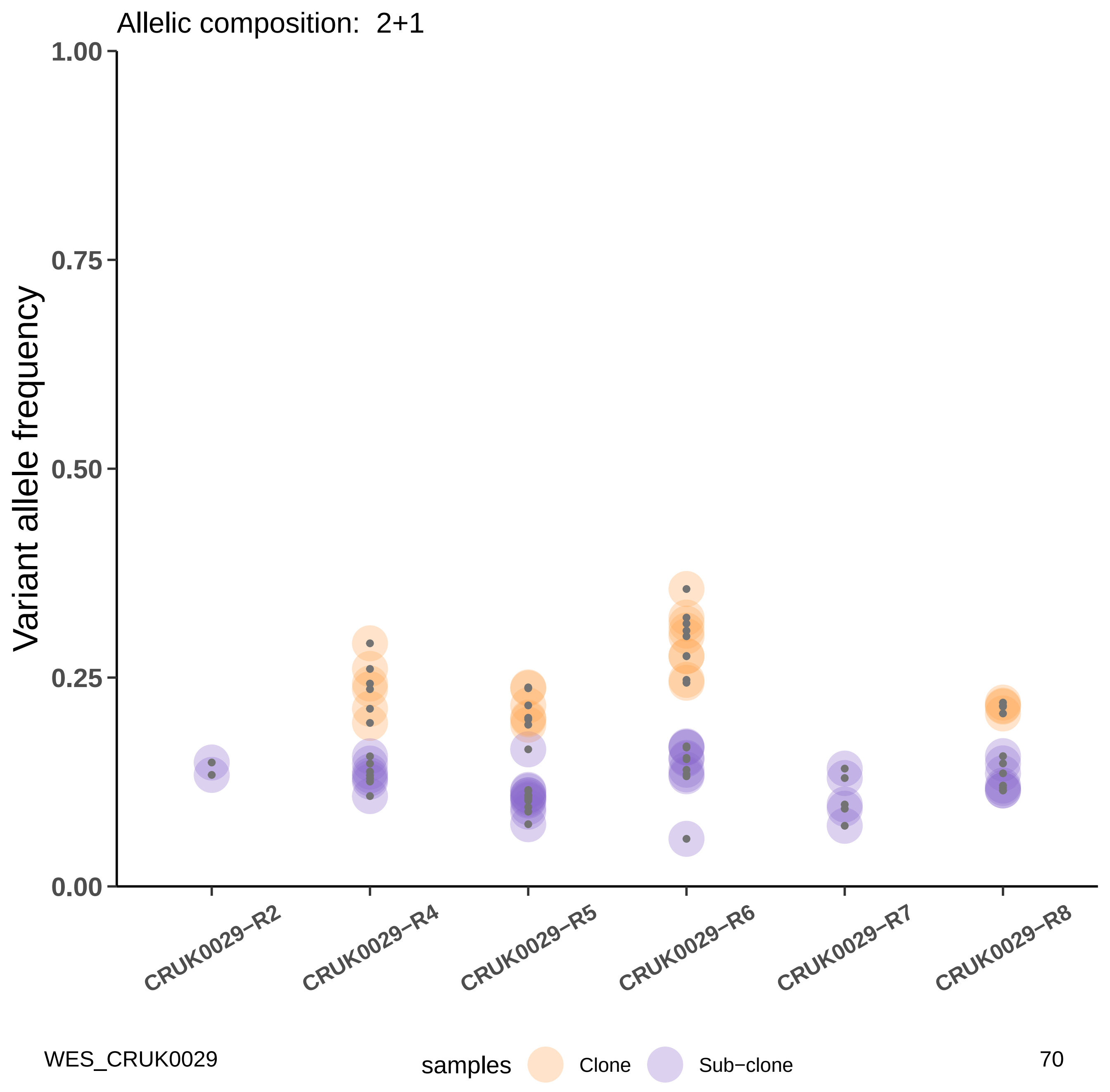

WES_CRUK0029

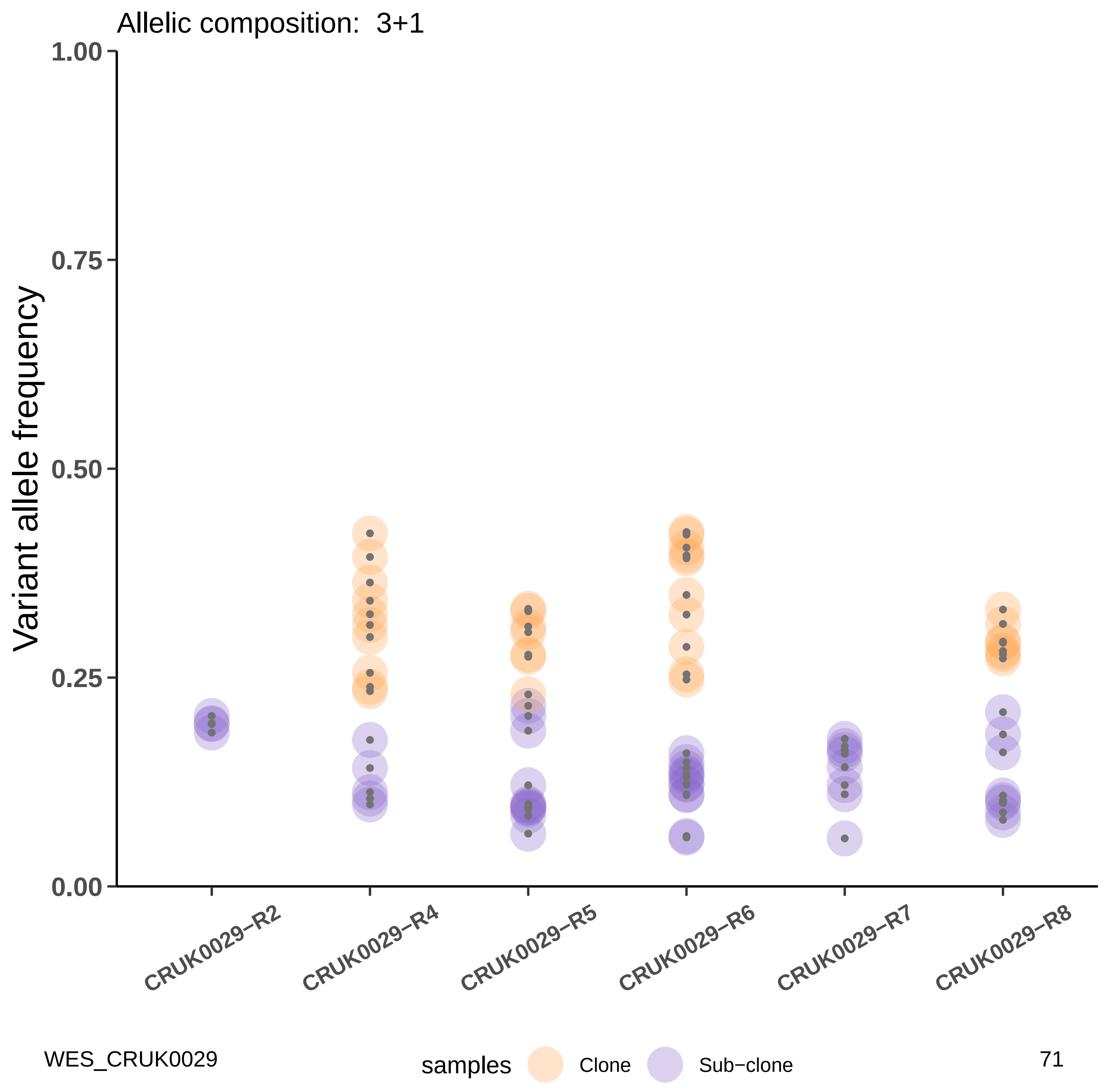

Allelic composition: 3+1
1.00
0.75
0.50
0.25
0.00
Variant allele frequency
CRUK0029−R2
CRUK0029−R4
CRUK0029−R5
CRUK0029−R6
CRUK0029−R7
CRUK0029−R8
samples
Clone
Sub−clone

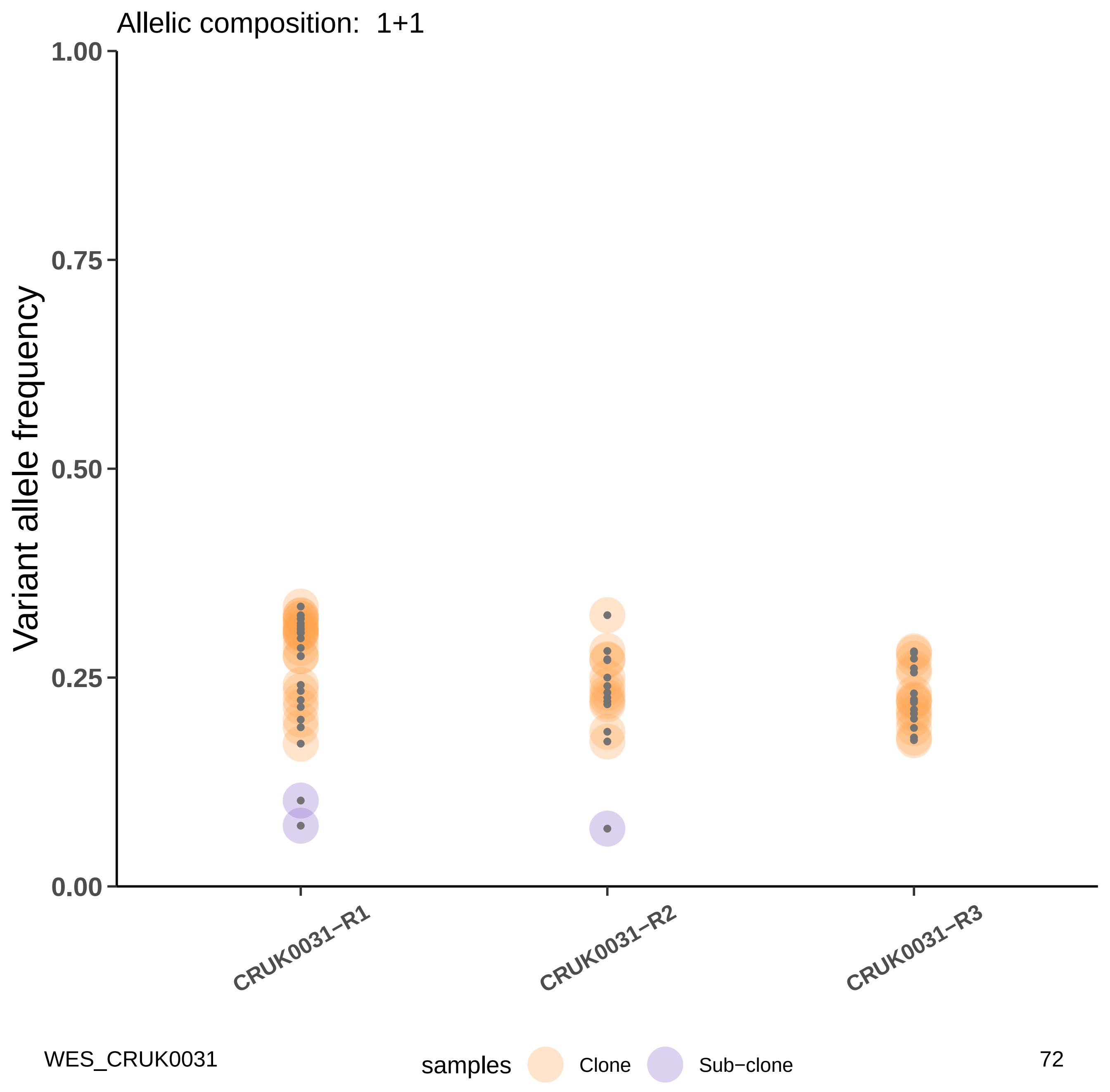

WES_CRUK0031

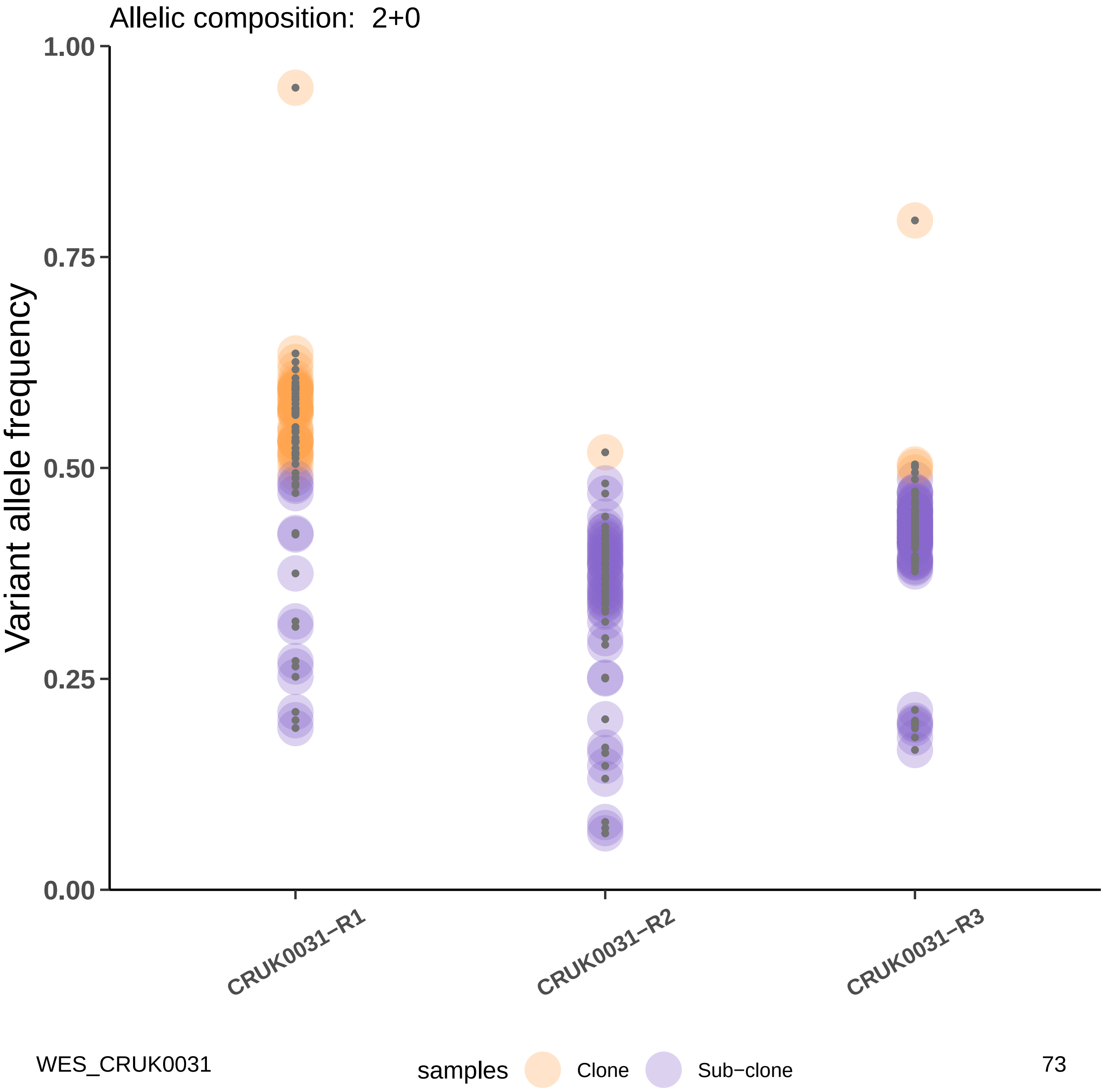

WES_CRUK0031

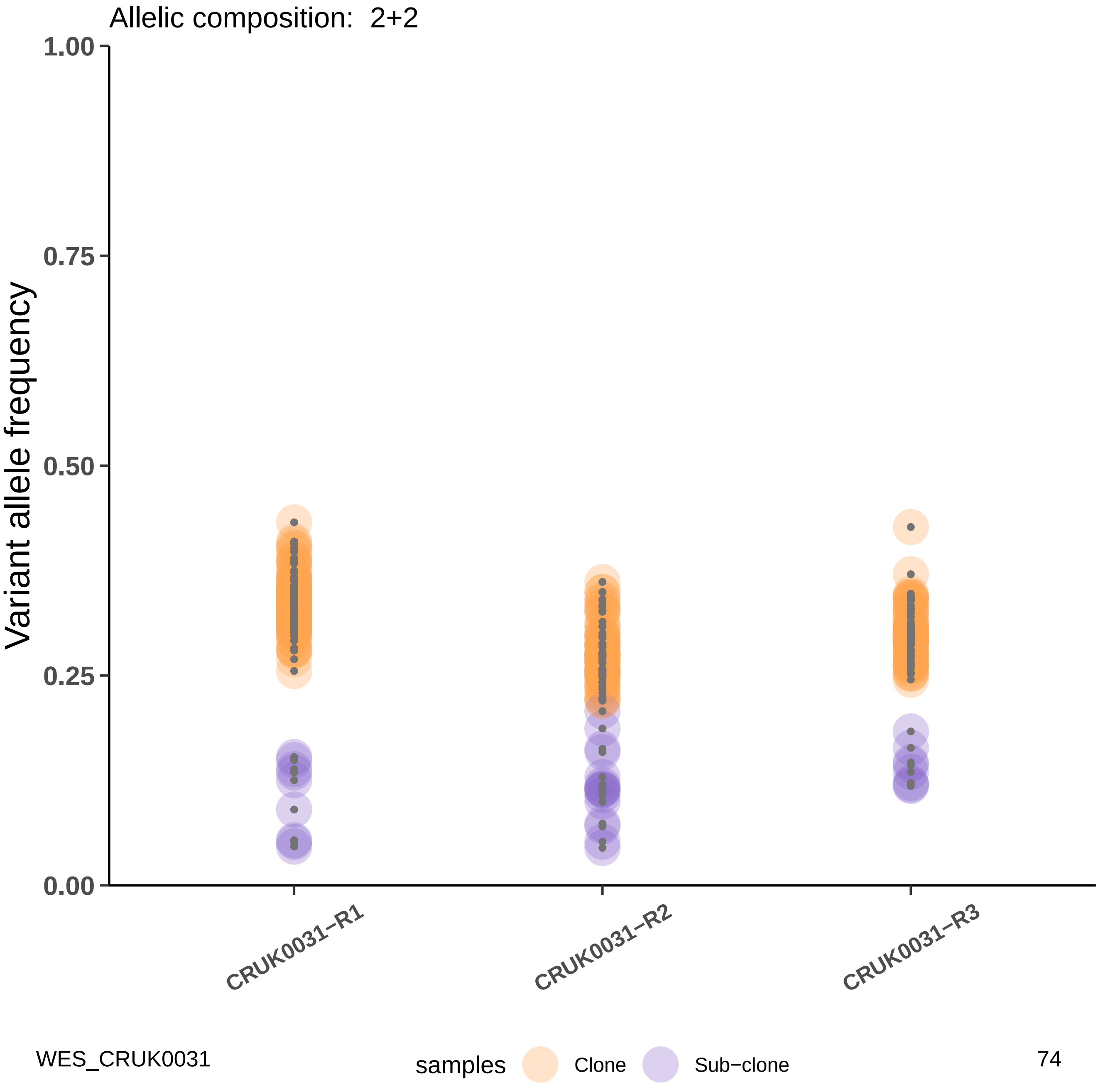

WES_CRUK0031

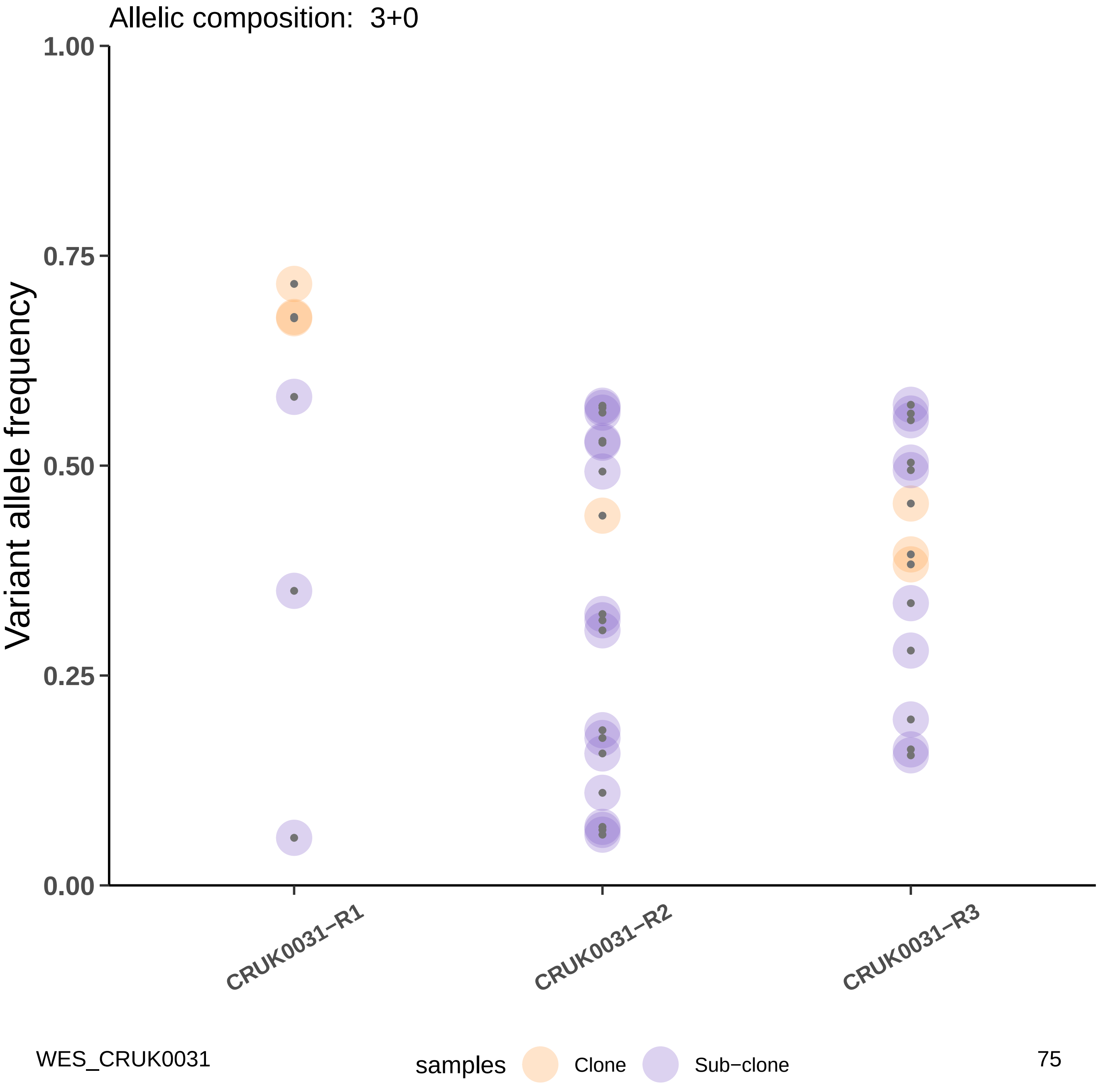

WES_CRUK0031

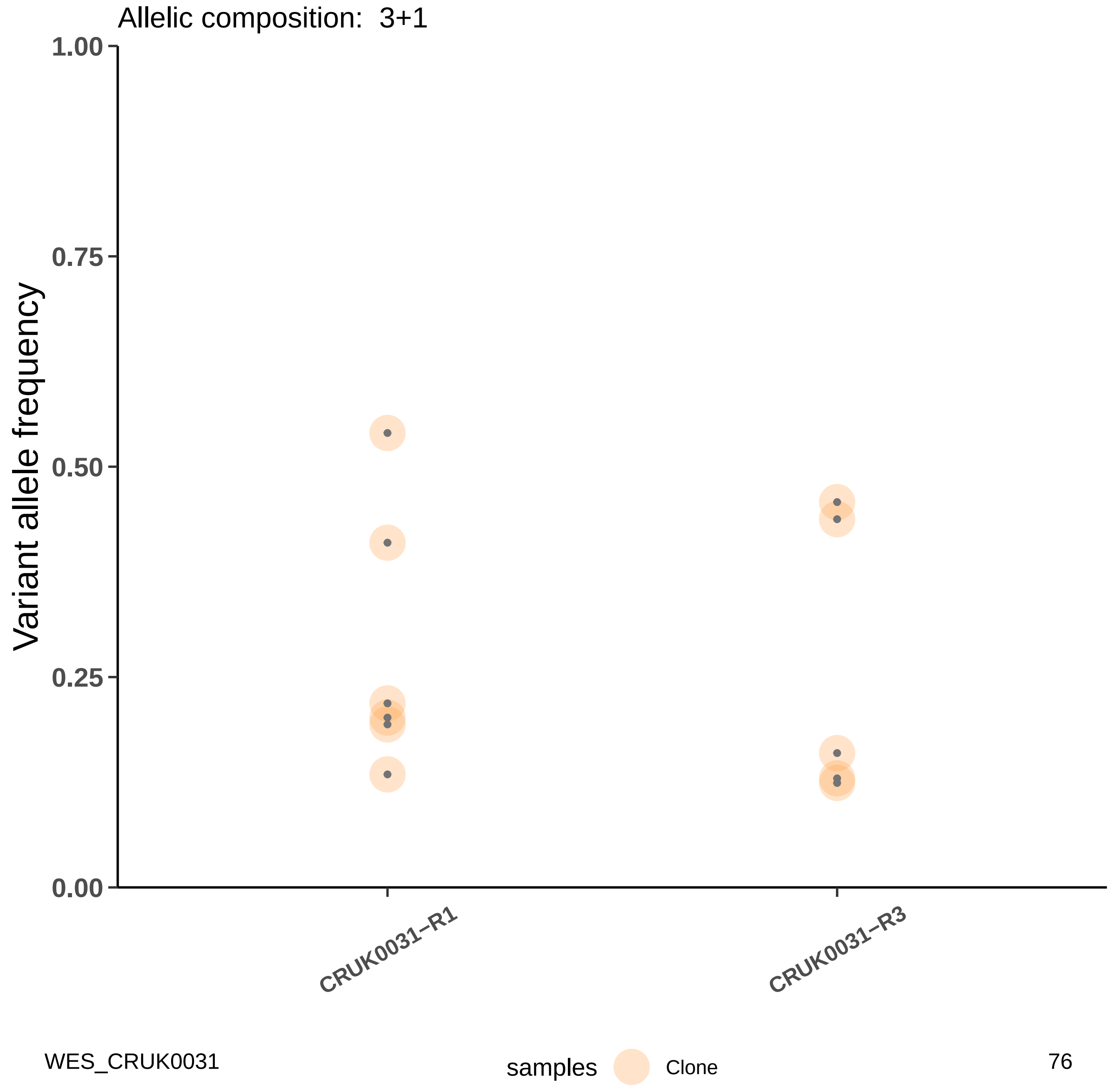

Allelic composition: 3+1
Variant allele frequency
1.00
0.75
0.50
0.25
0.00
CRUK0031–R1
CRUK0031–R3
samples
Clone

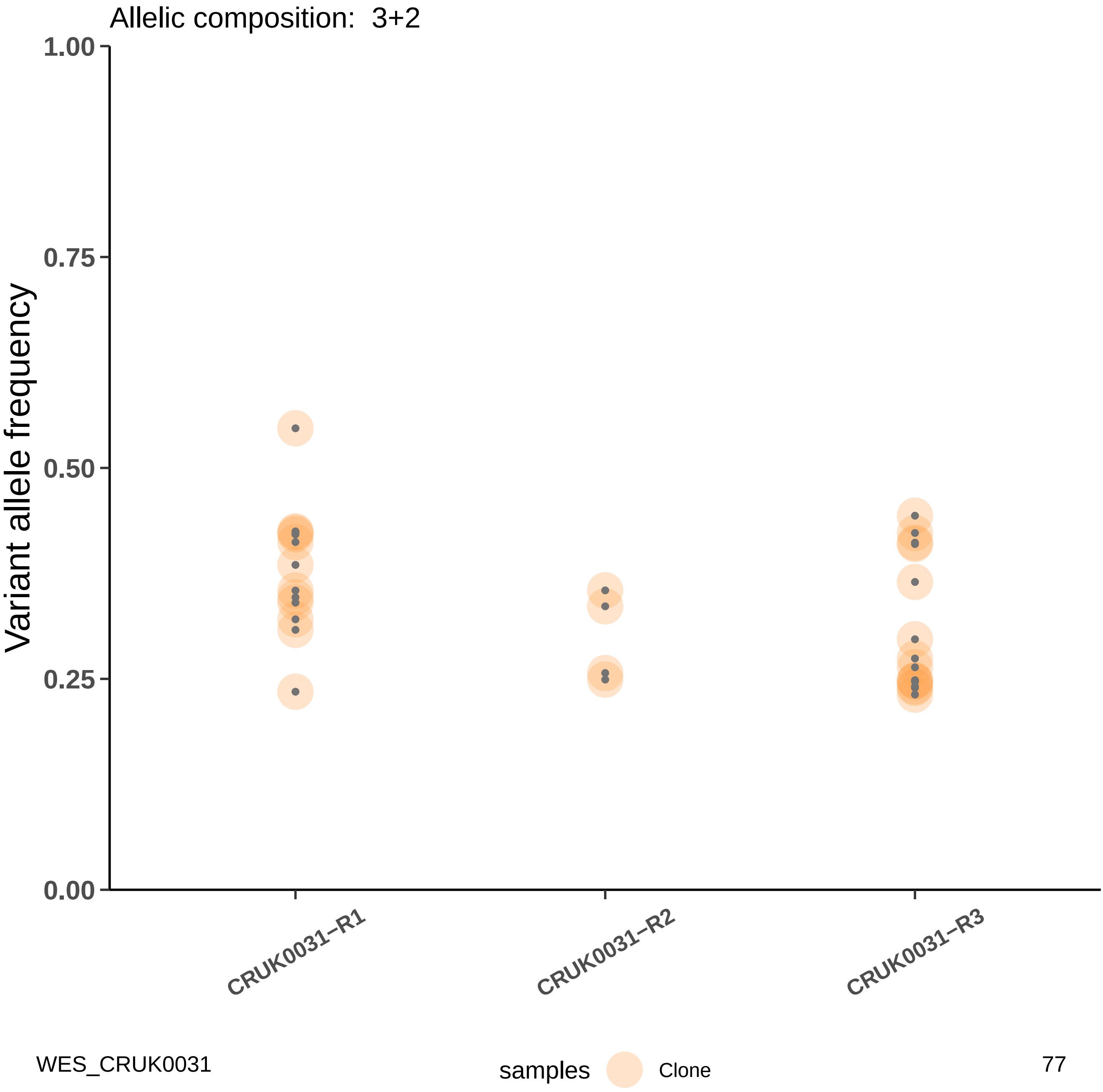

WES_CRUK0031

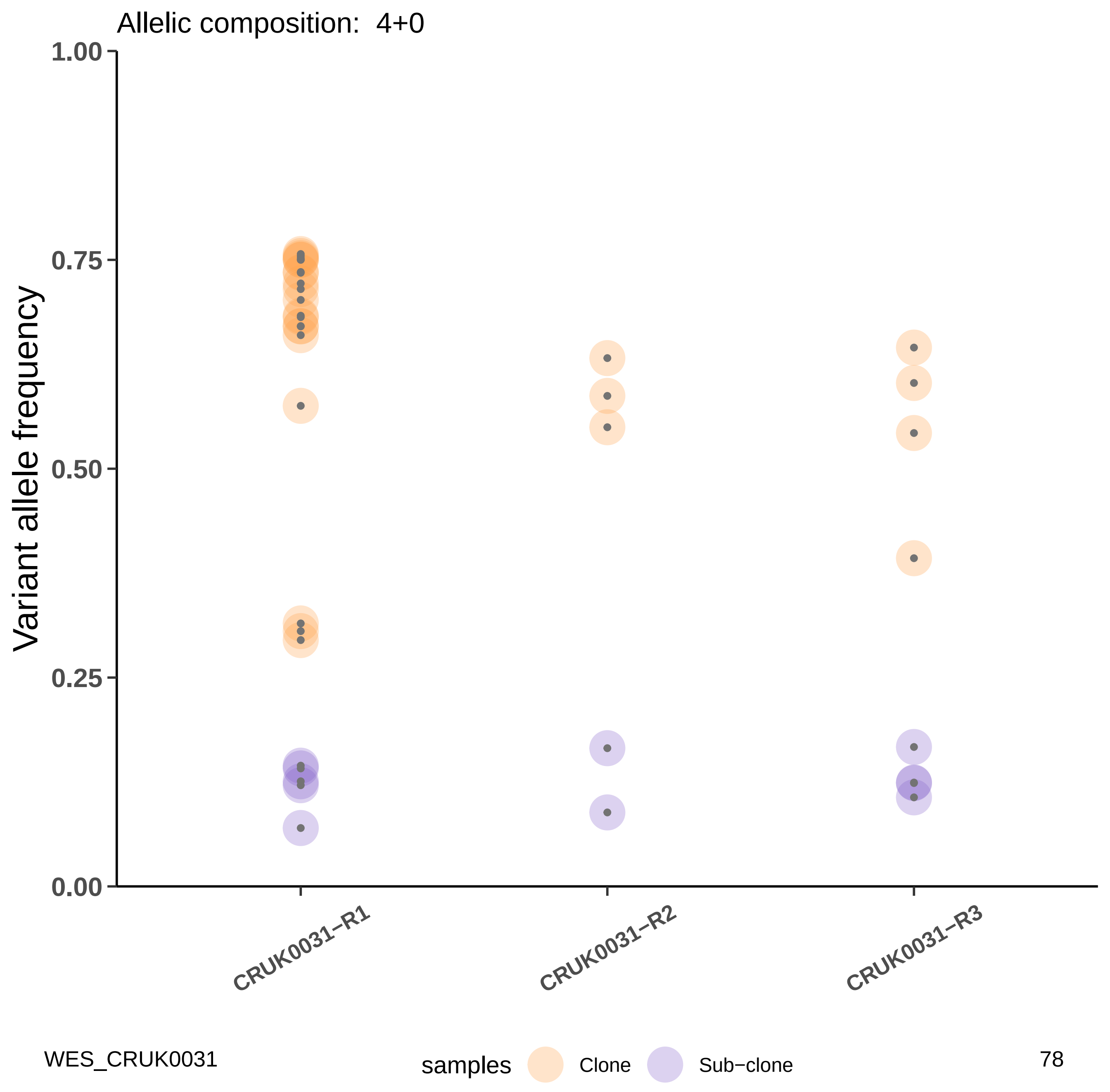

WES_CRUK0031

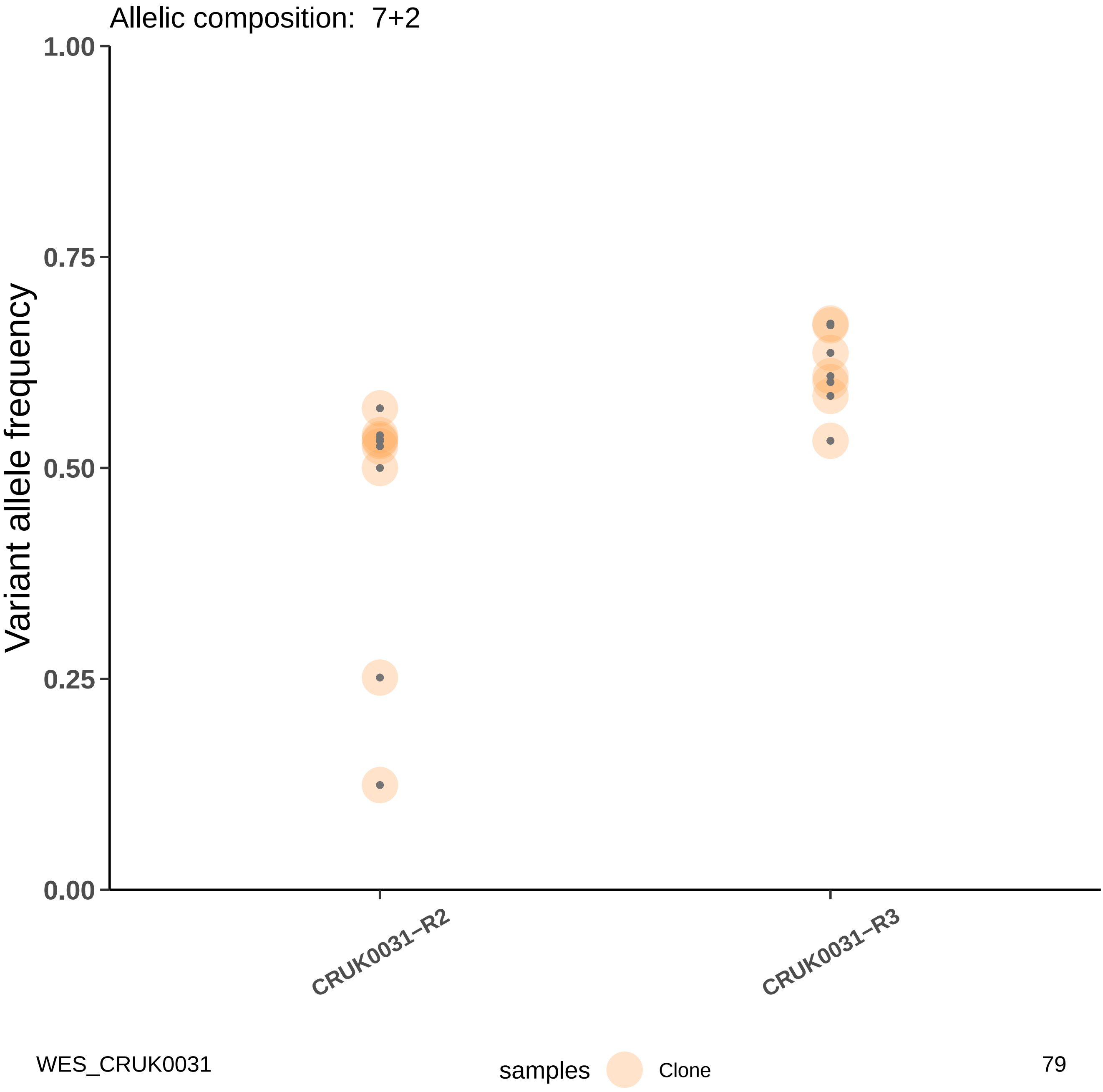

WES_CRUK0031

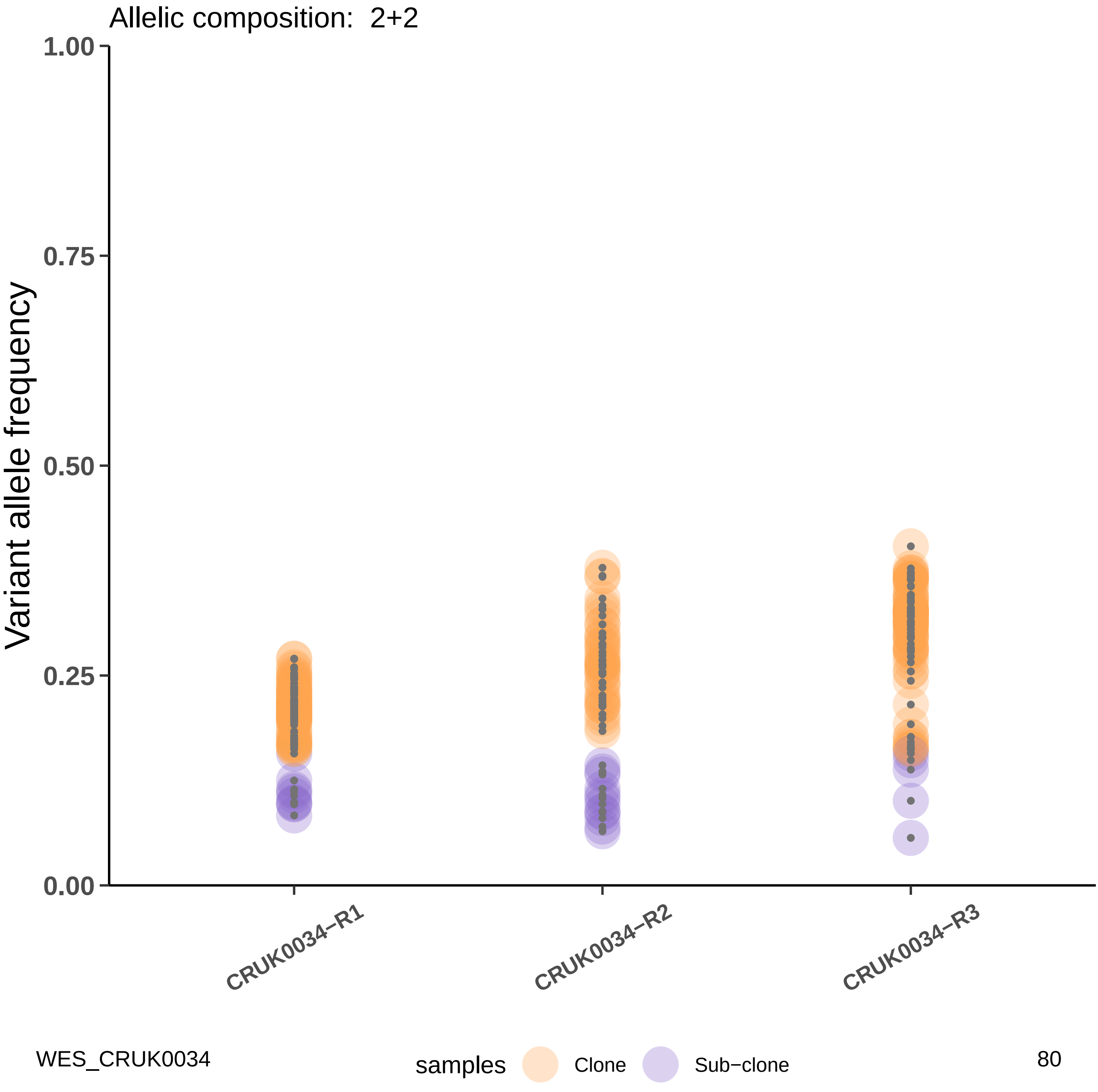
Allelic composition: 2+2
Variant allele frequency
1.00
0.75
0.50
0.25
0.00
CRUK0034−R1
CRUK0034−R2
CRUK0034−R3
samples
Clone
Sub−clone

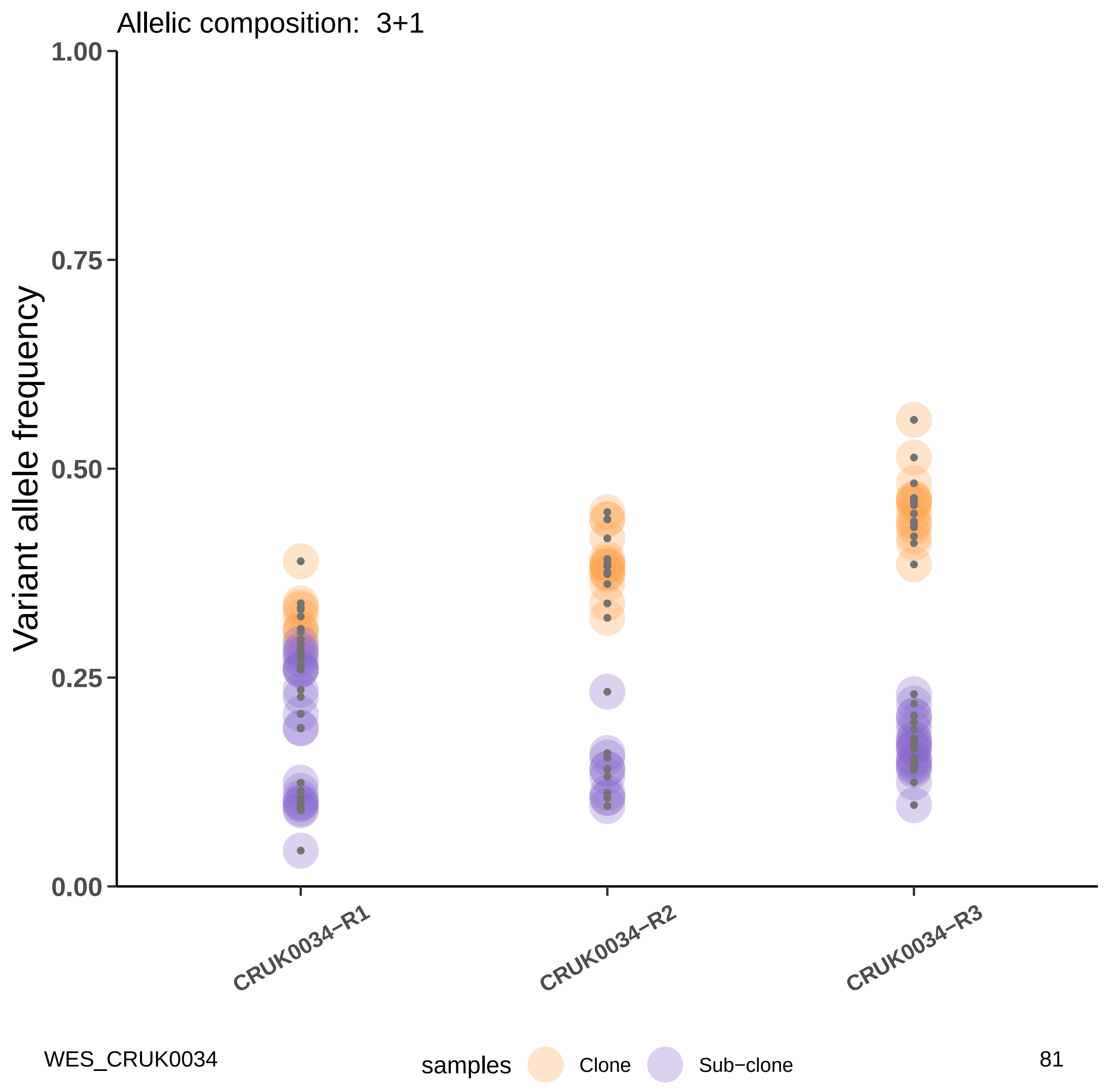

WES_CRUK0034

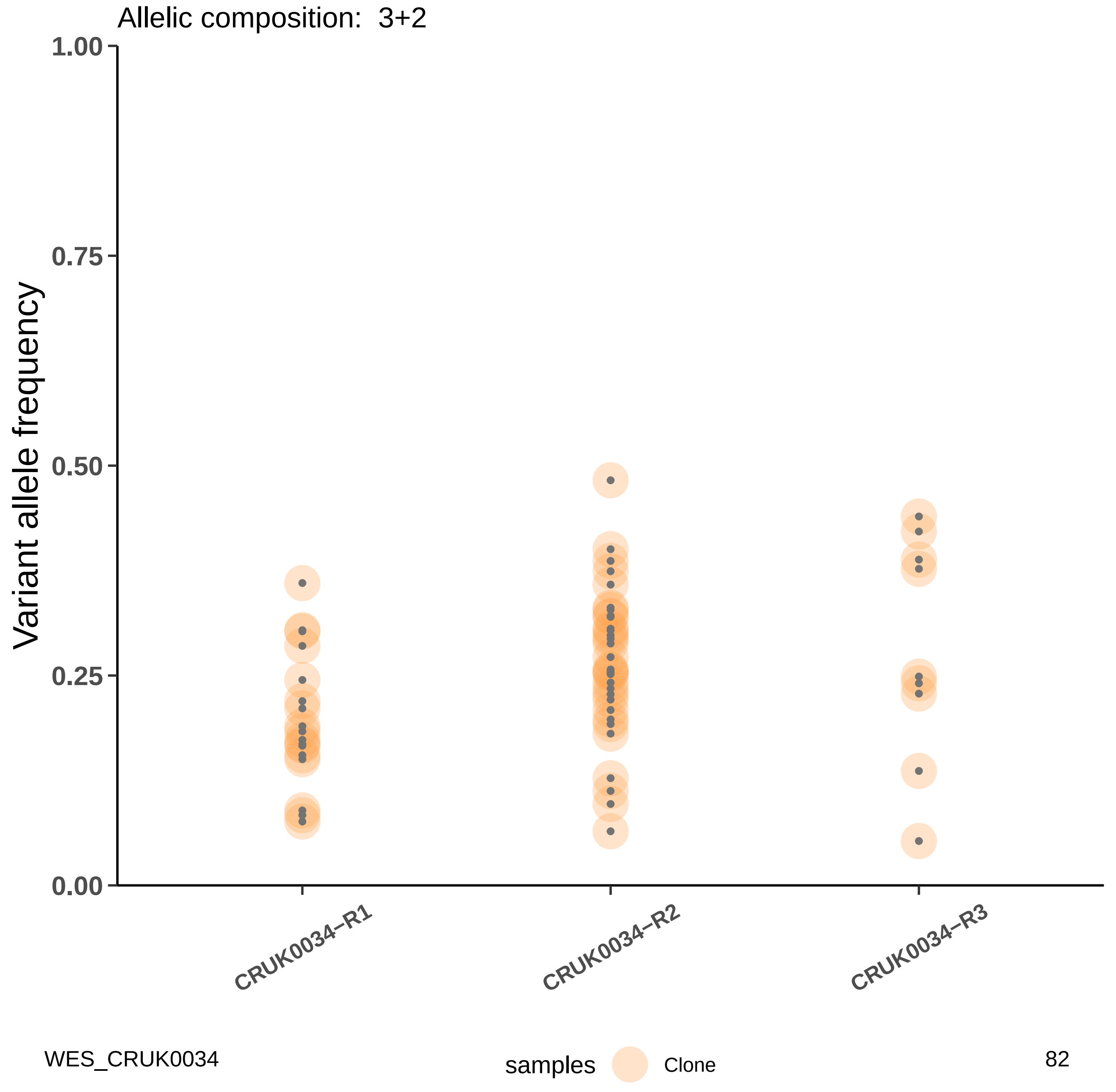

WES_CRUK0034

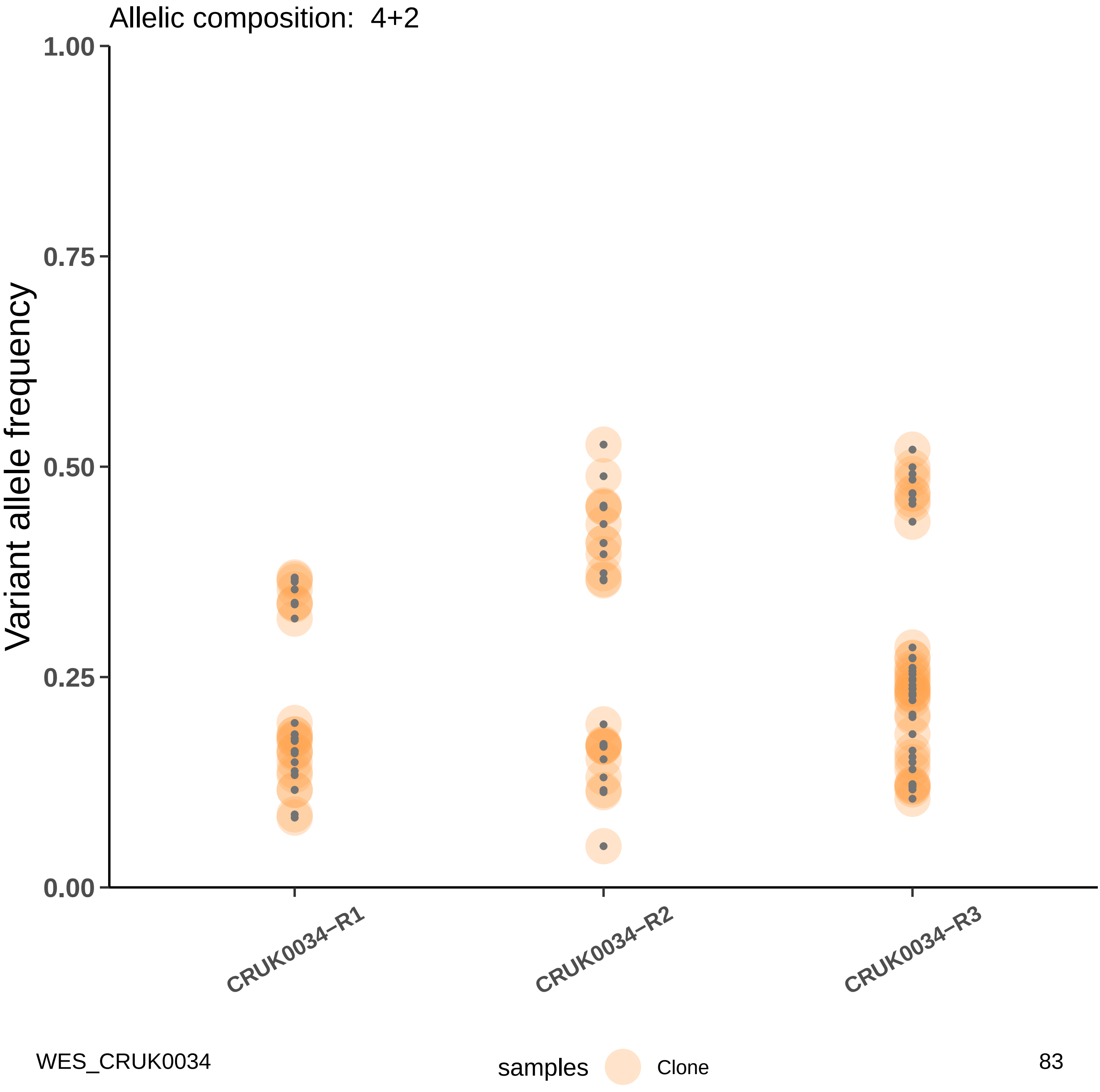

WES_CRUK0034

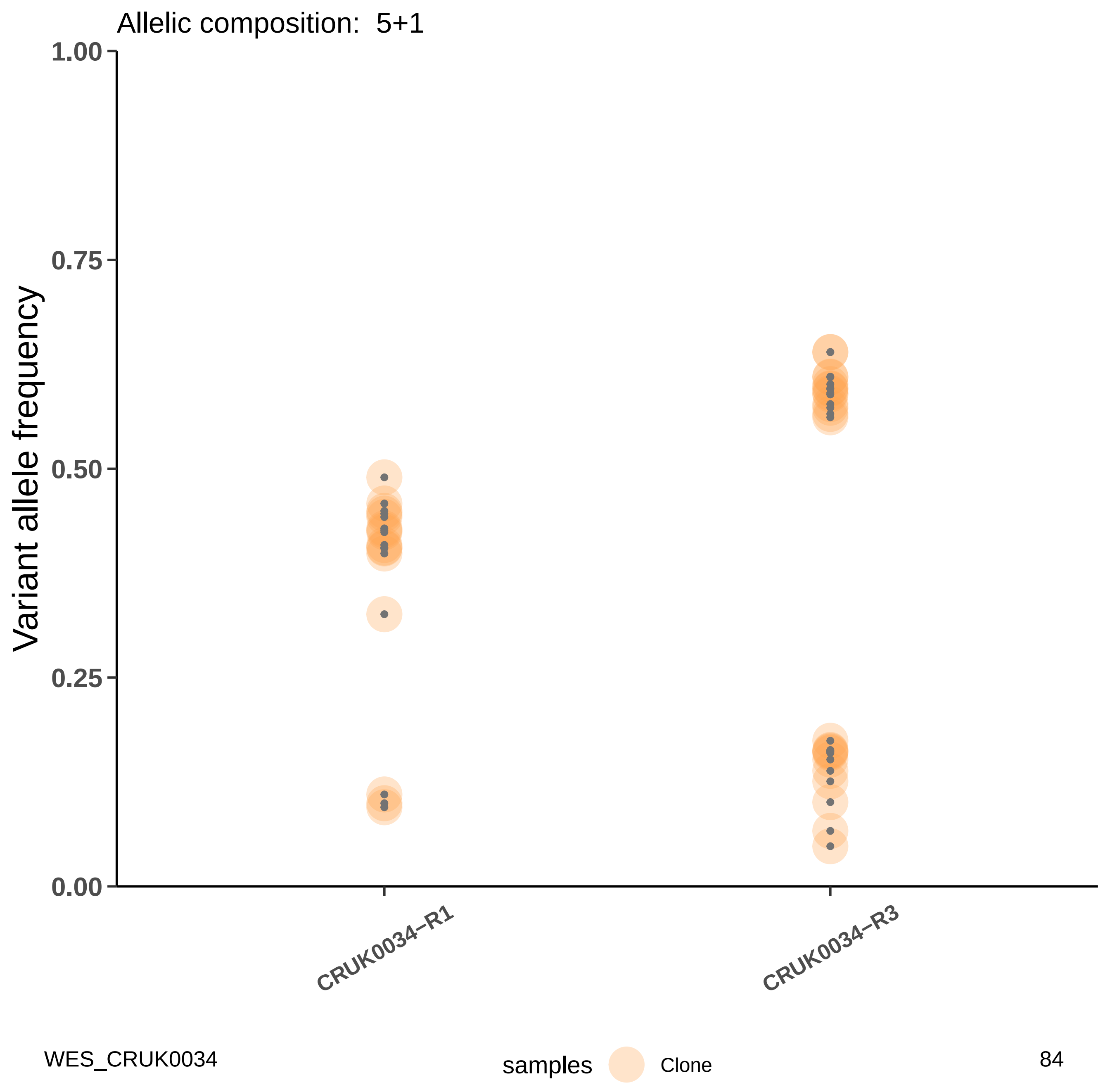

WES_CRUK0034

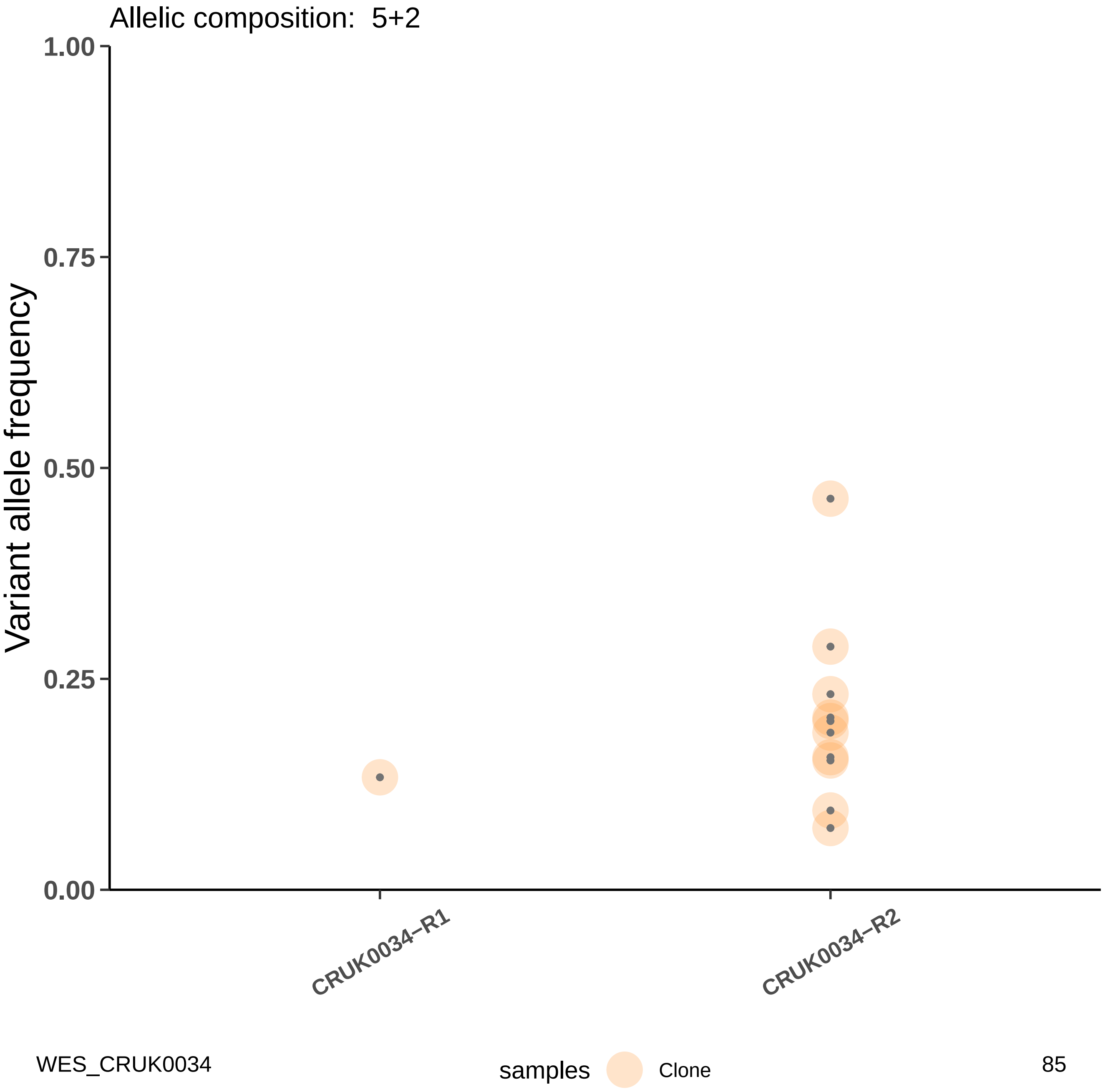

WES_CRUK0034

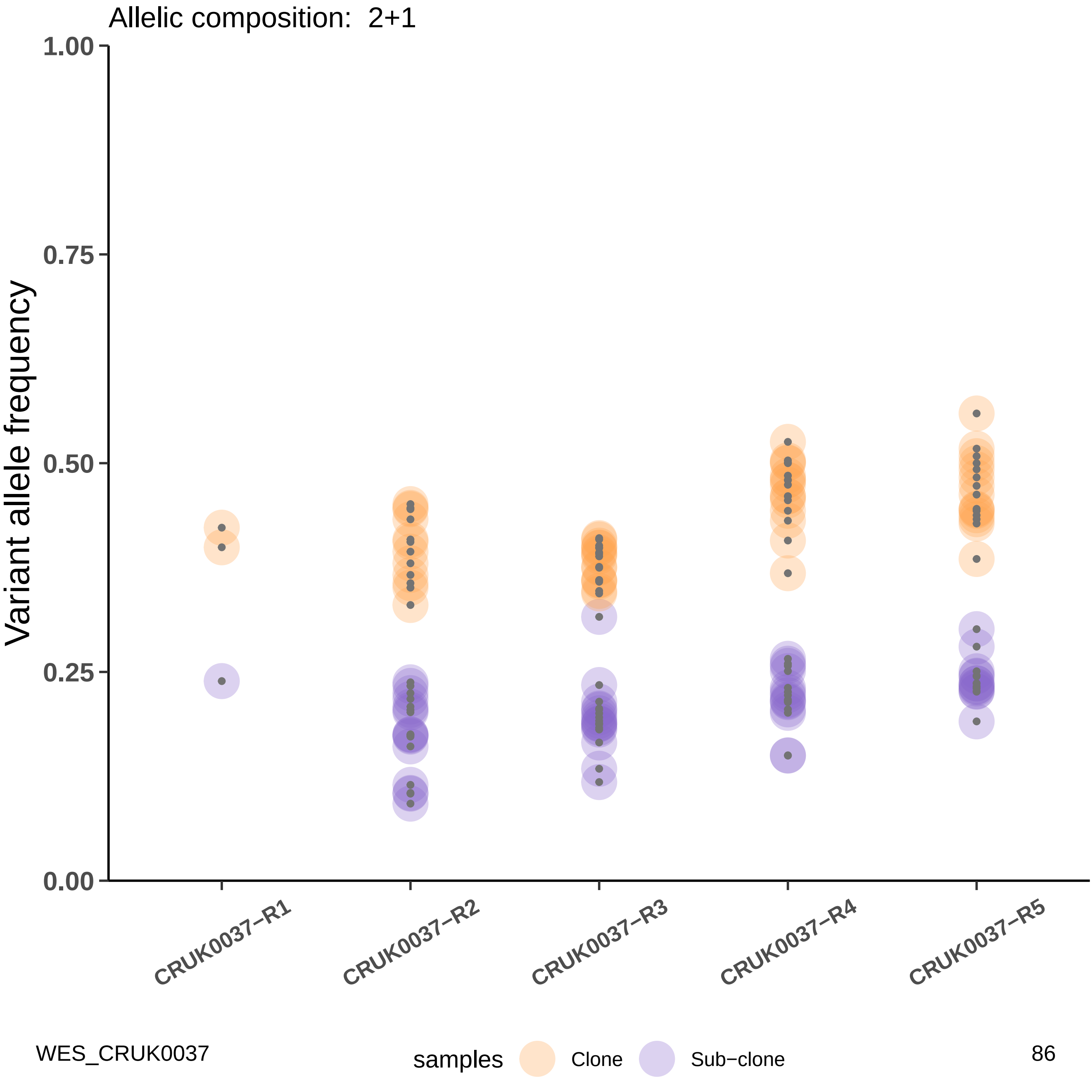

WES_CRUK0037

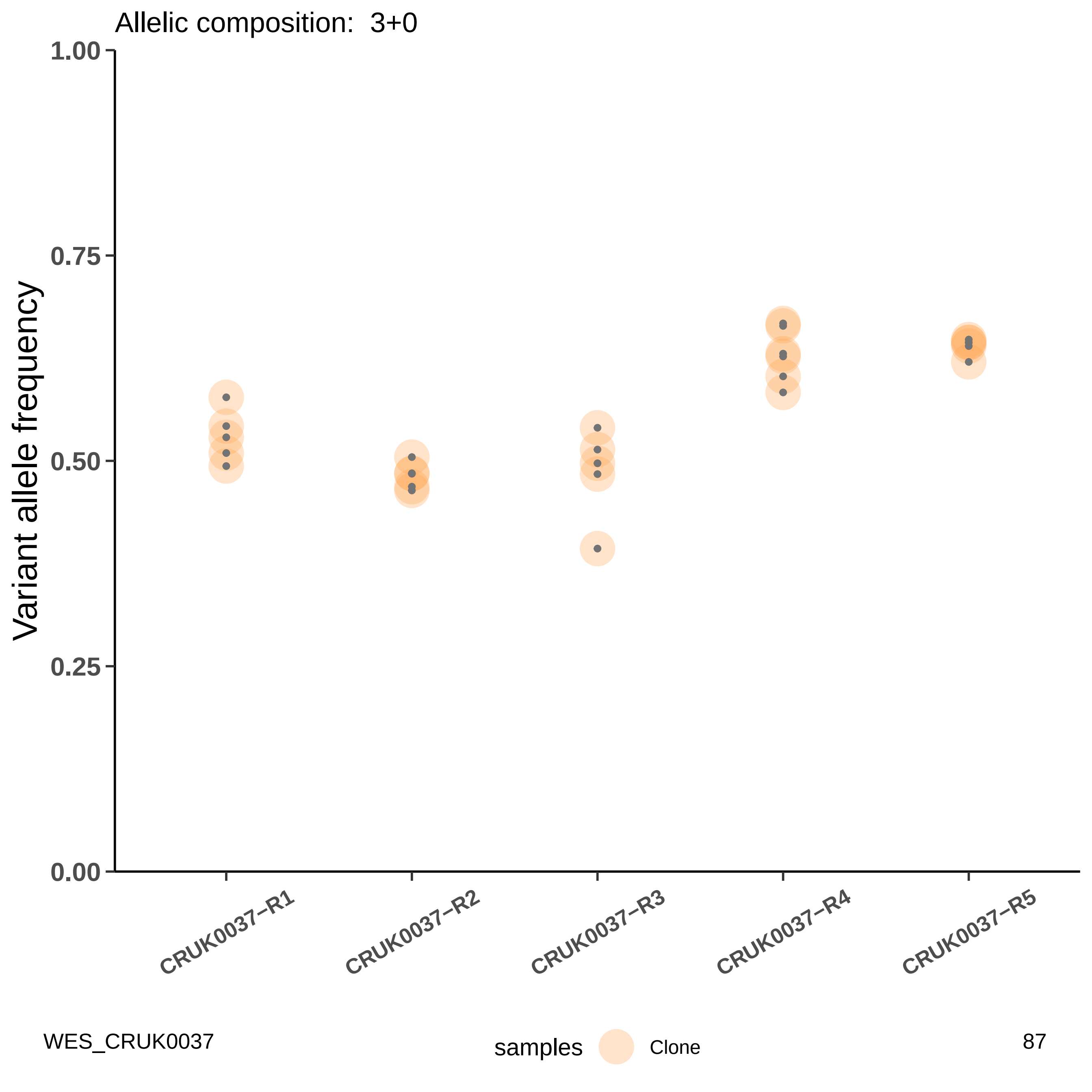
Allelic composition: 3+0
Variant allele frequency
1.00
0.75
0.50
0.25
0.00
CRUK0037−R1
CRUK0037−R2
CRUK0037−R3
CRUK0037−R4
CRUK0037−R5
WES_CRUK0037
samples
Clone
87

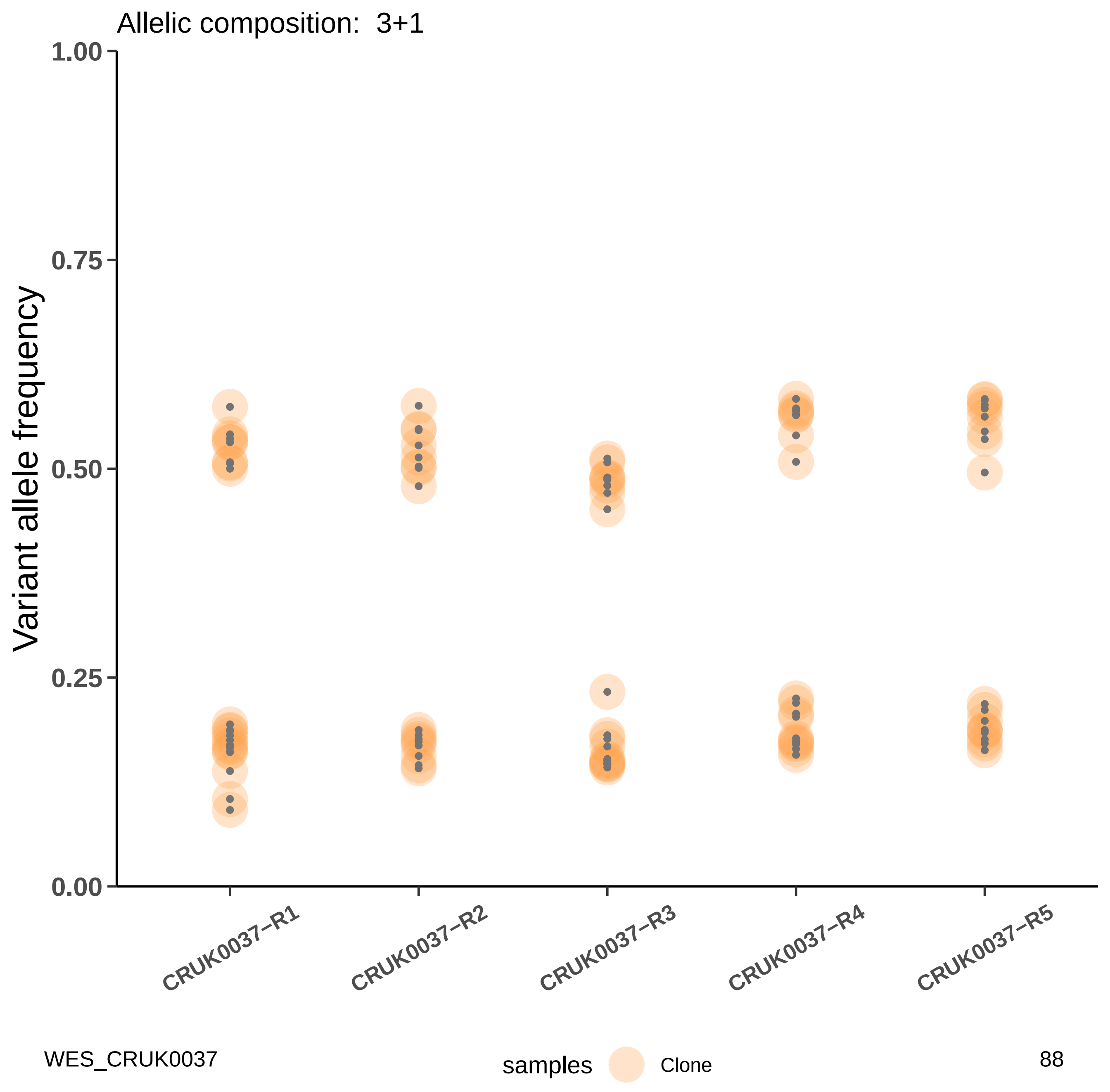

WES_CRUK0037

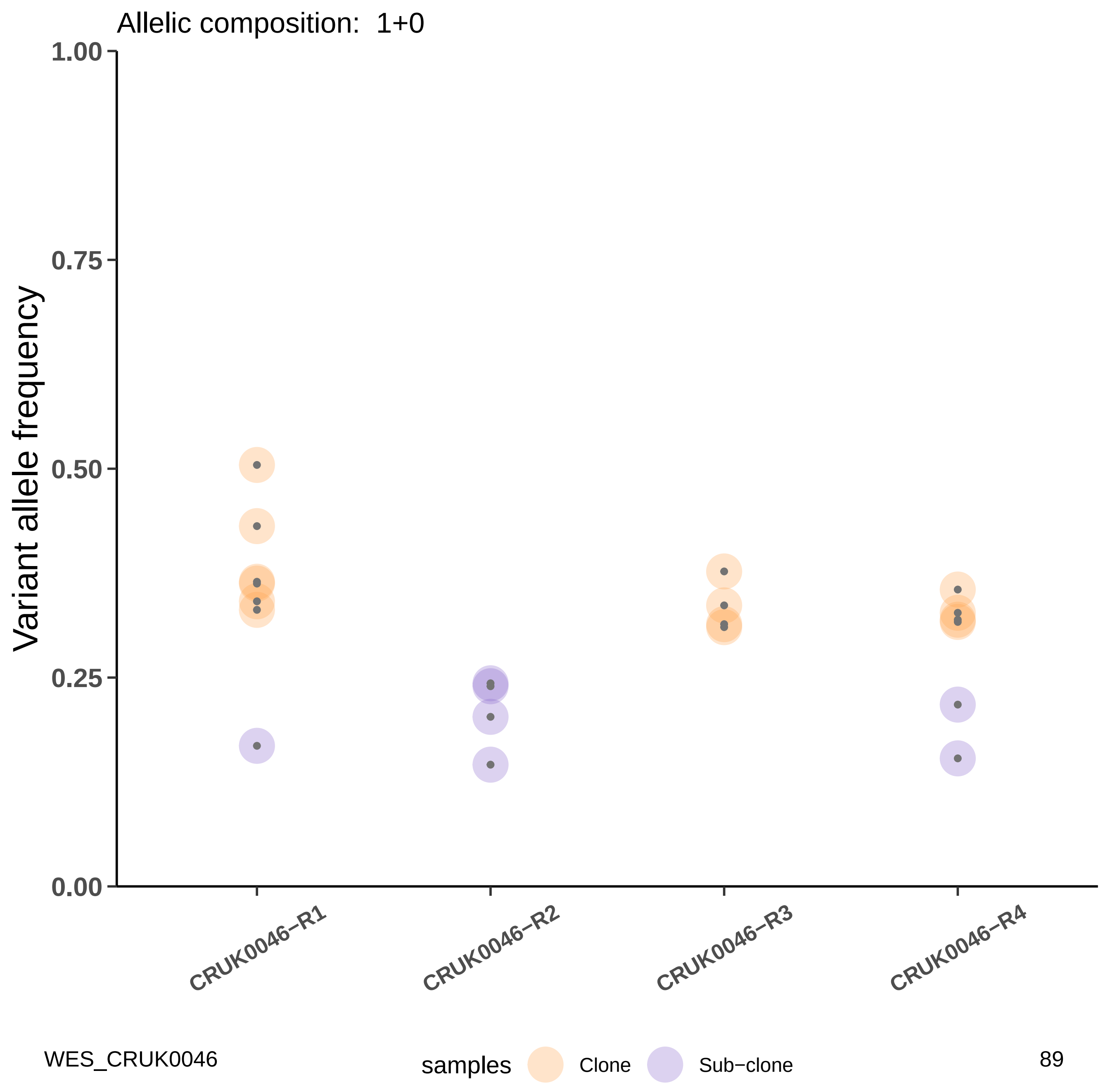

WES_CRUK0046

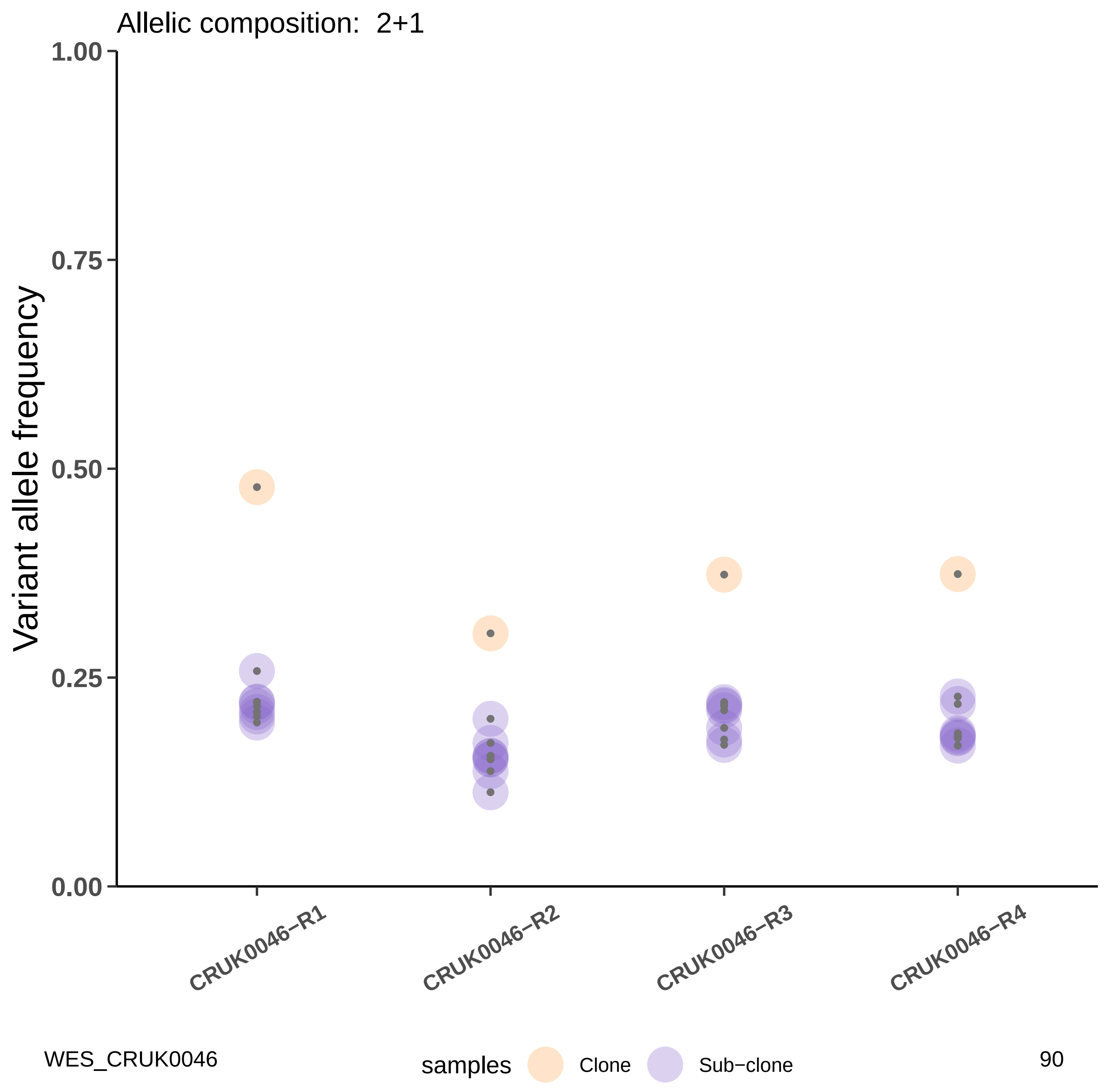

WES_CRUK0046

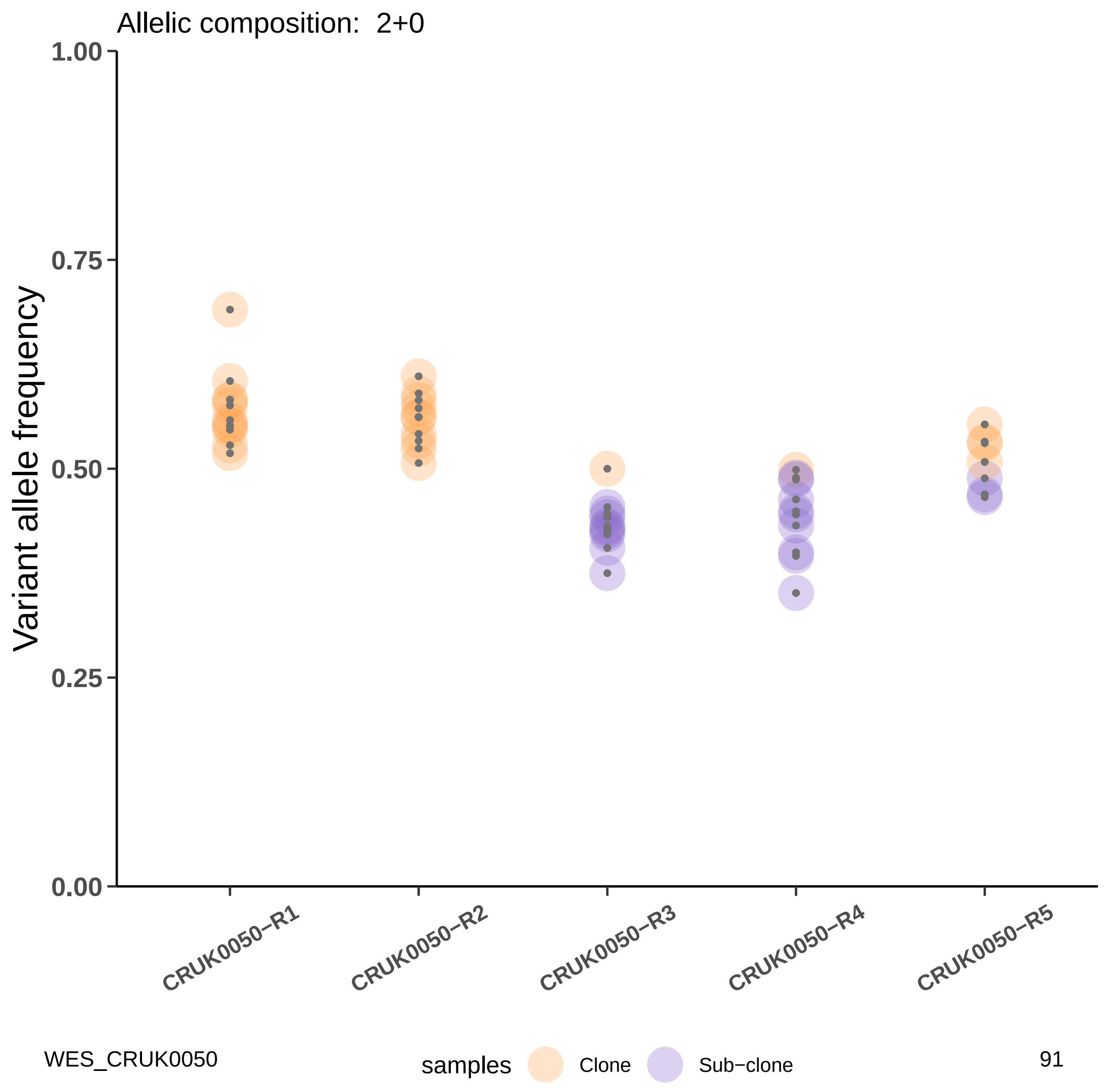

WES_CRUK0050

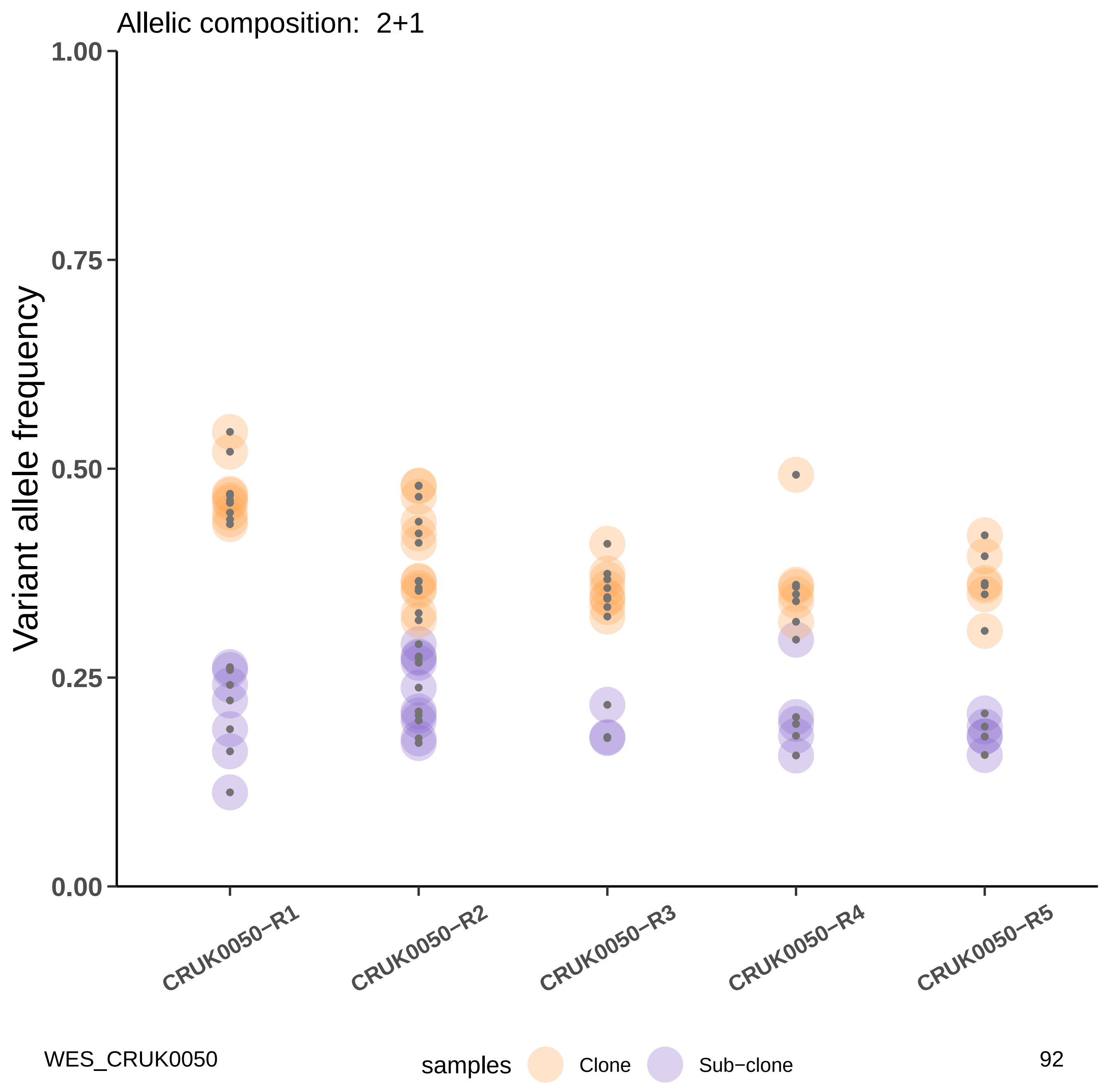

WES_CRUK0050

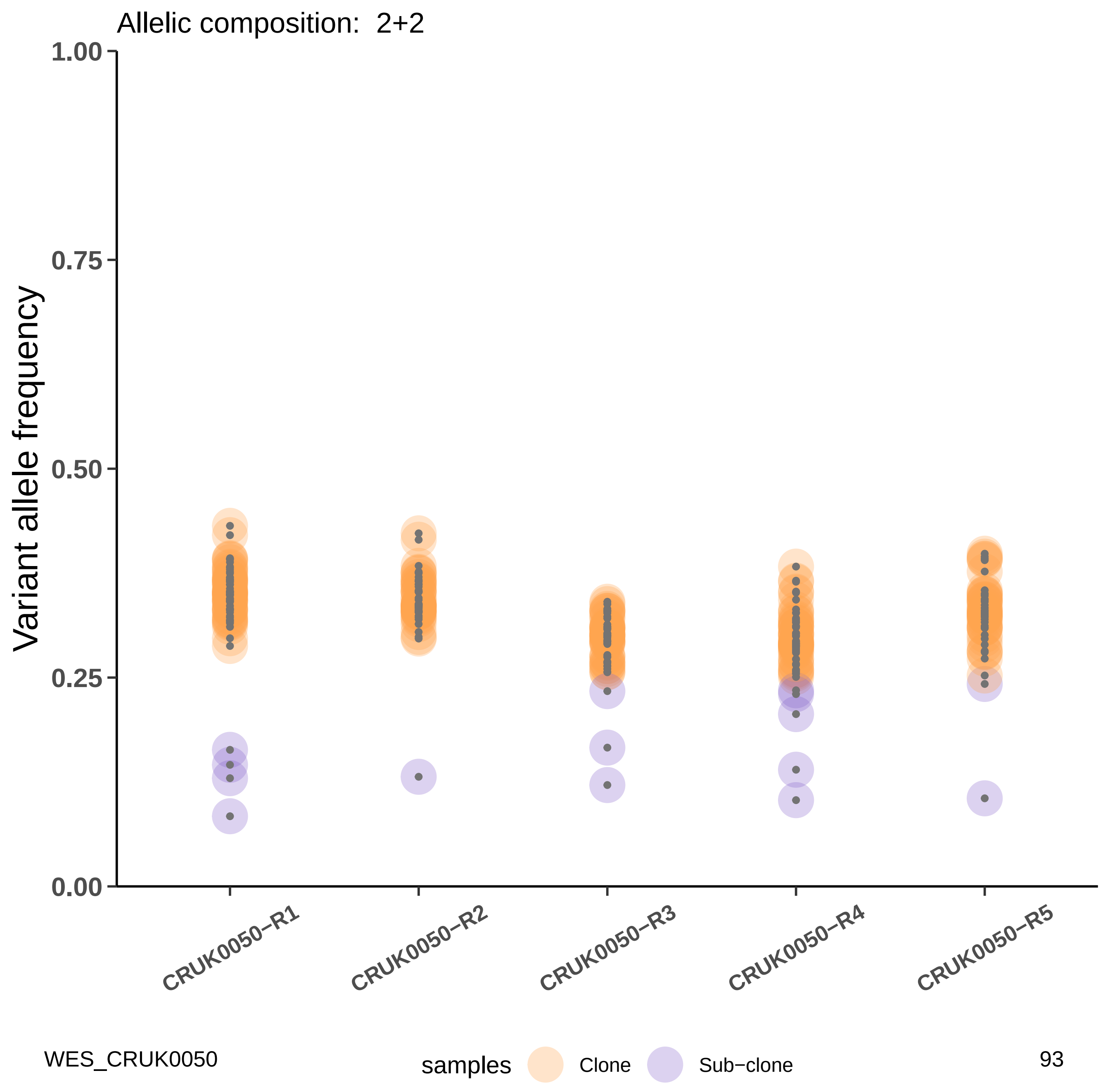

WES_CRUK0050

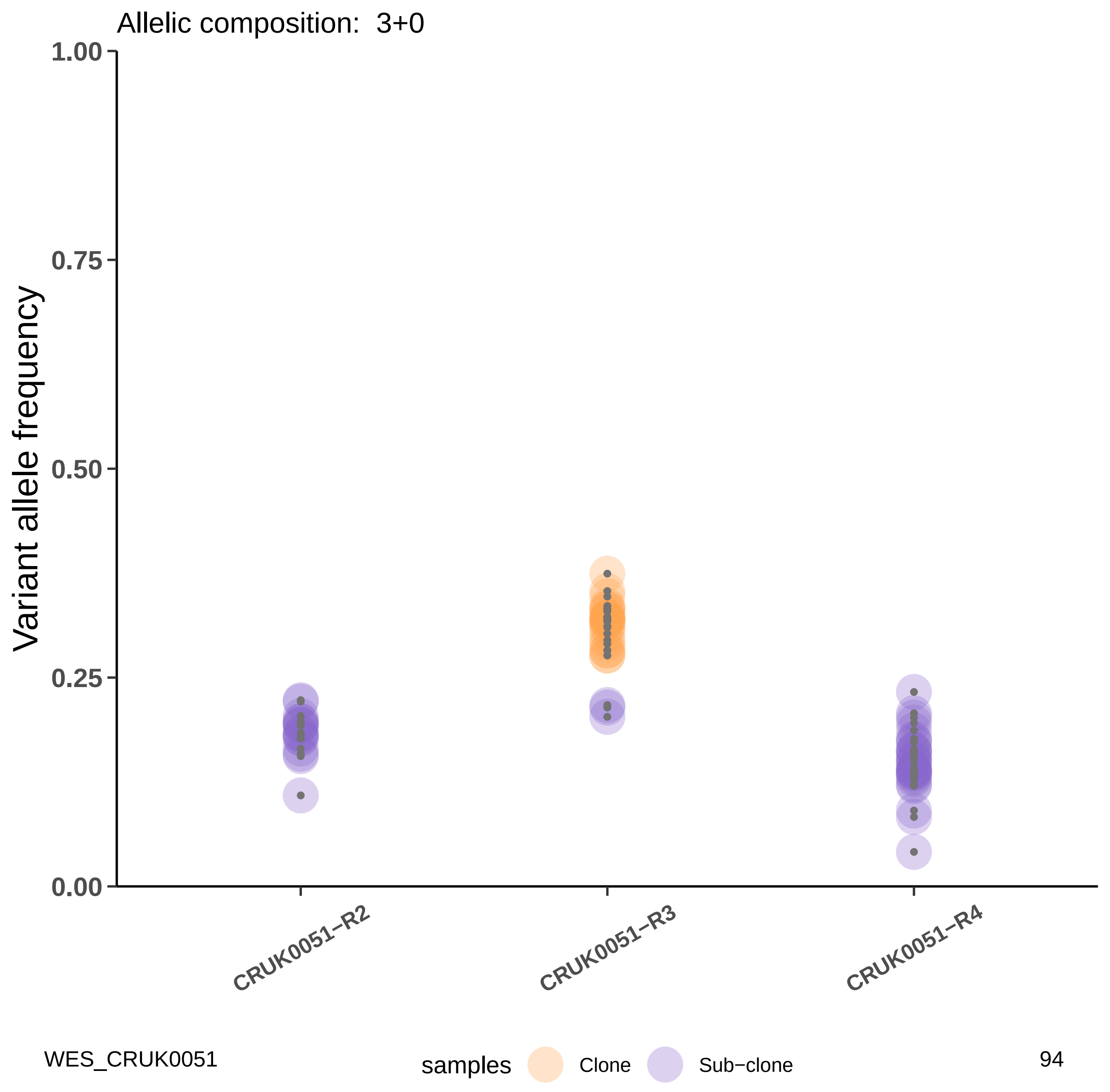

WES_CRUK0051

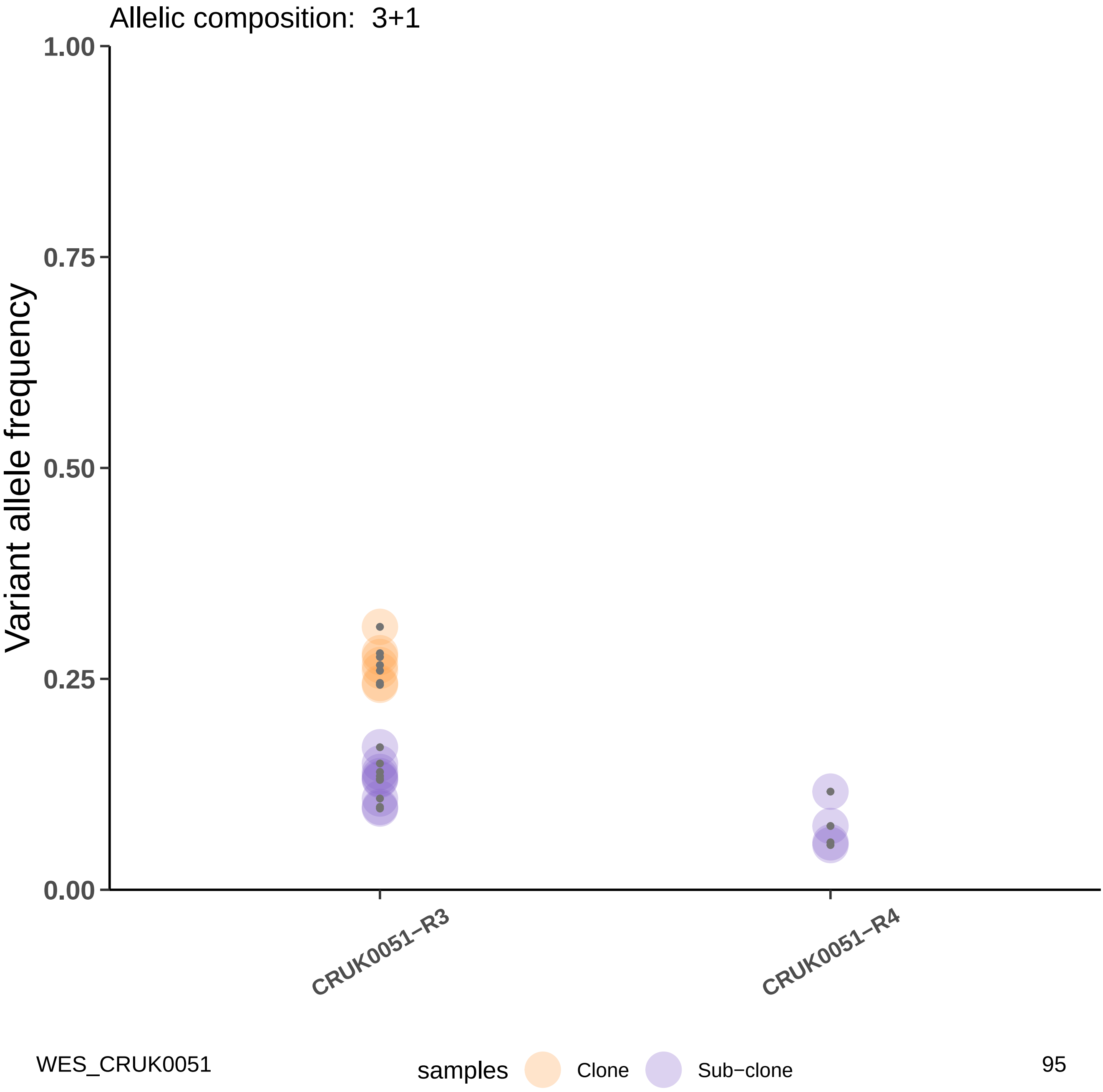

WES_CRUK0051

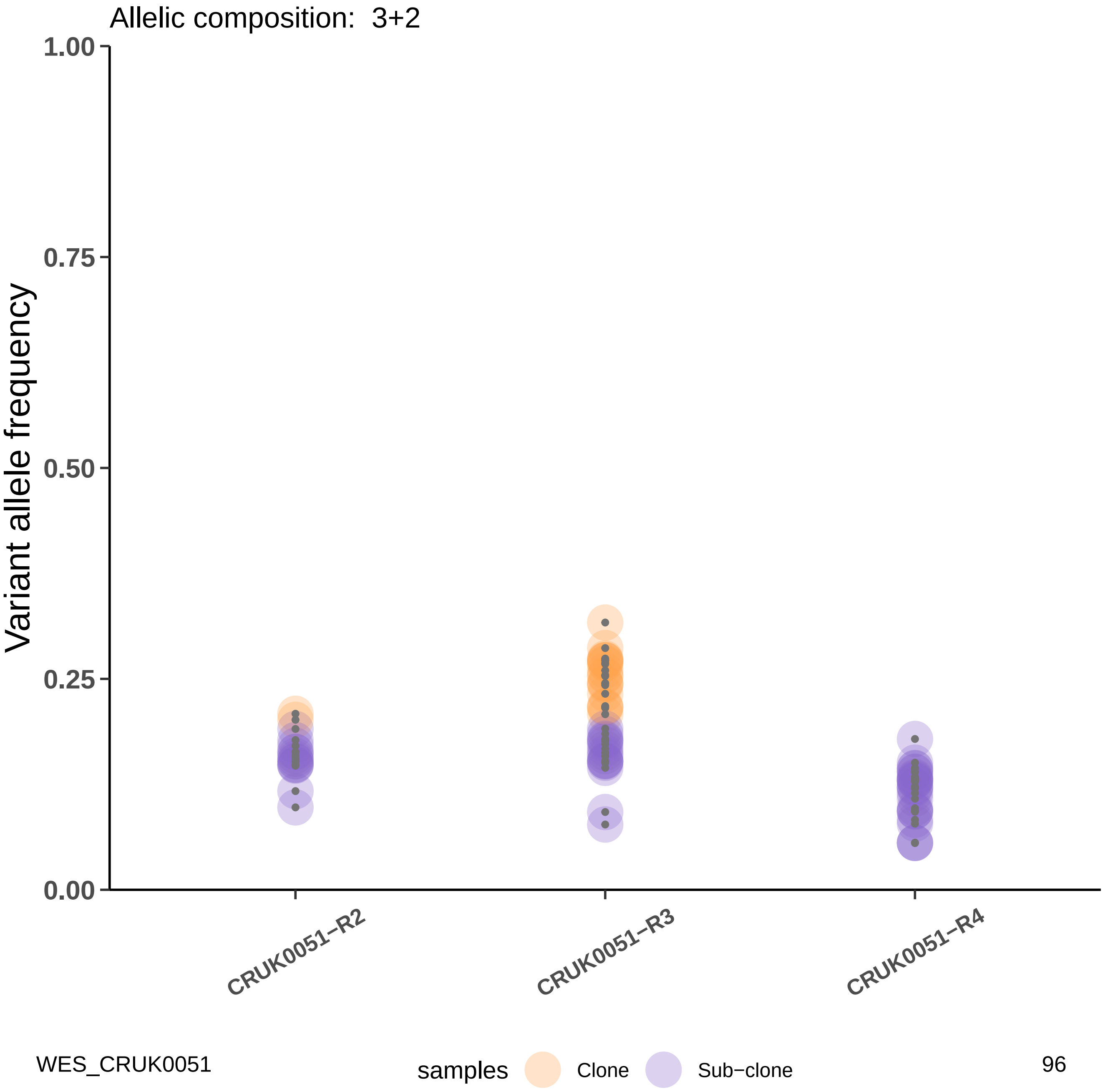

WES_CRUK0051

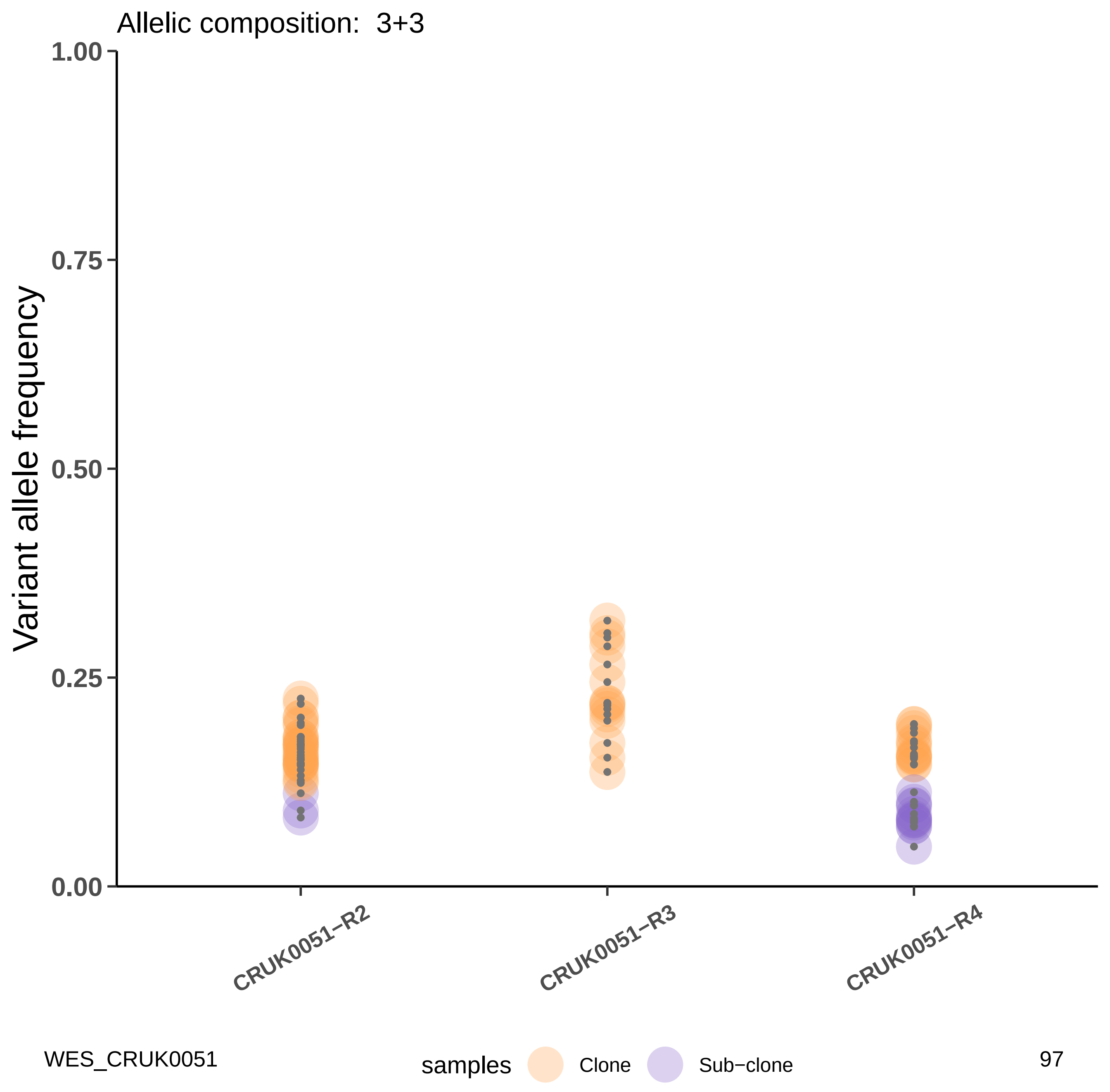

WES_CRUK0051

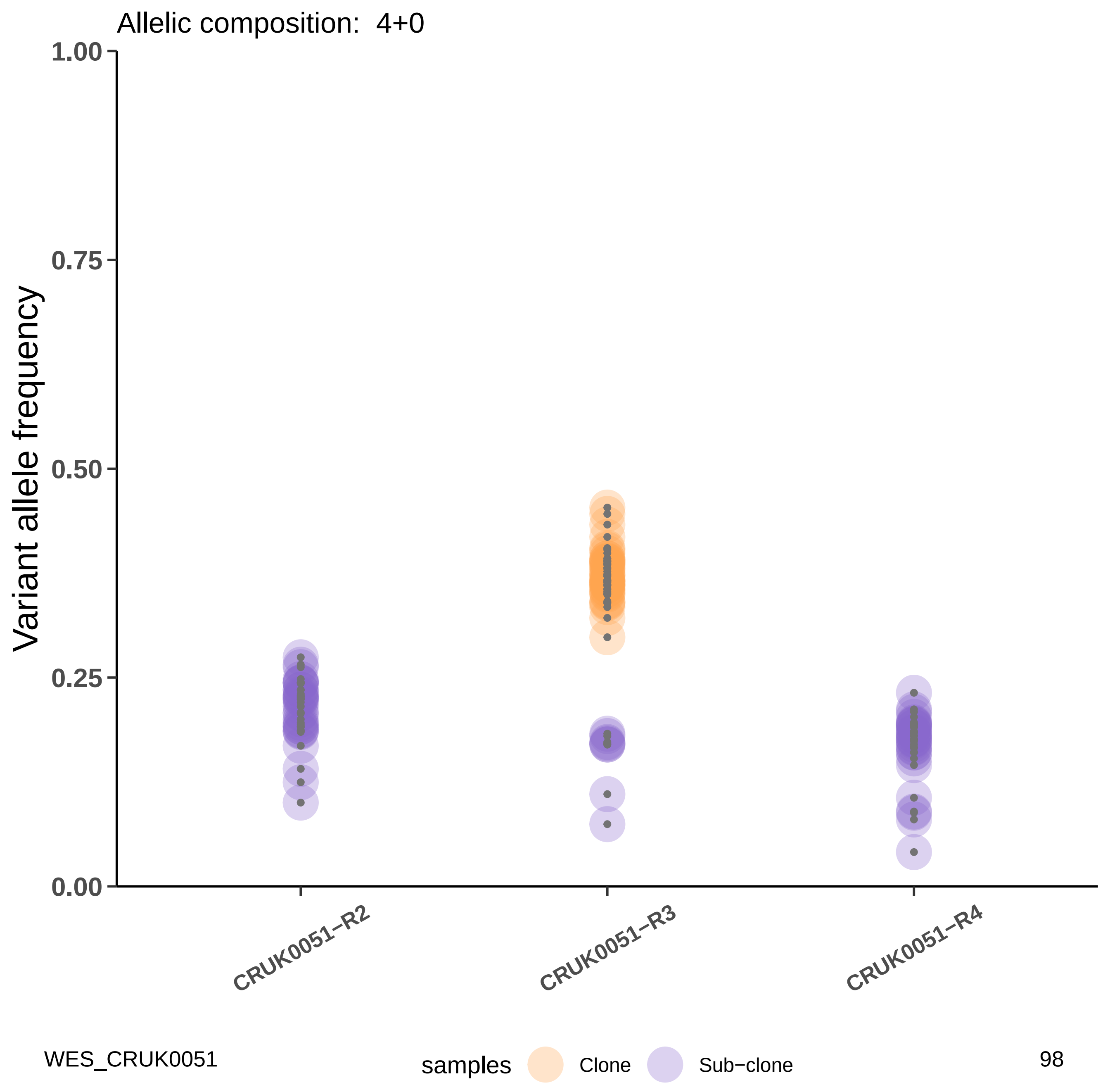

Allelic composition: 4+0
Variant allele frequency
1.00
0.75
0.50
0.25
0.00
CRUK0051−R2
CRUK0051−R3
CRUK0051−R4
WES_CRUK0051
samples
Clone
Sub−clone

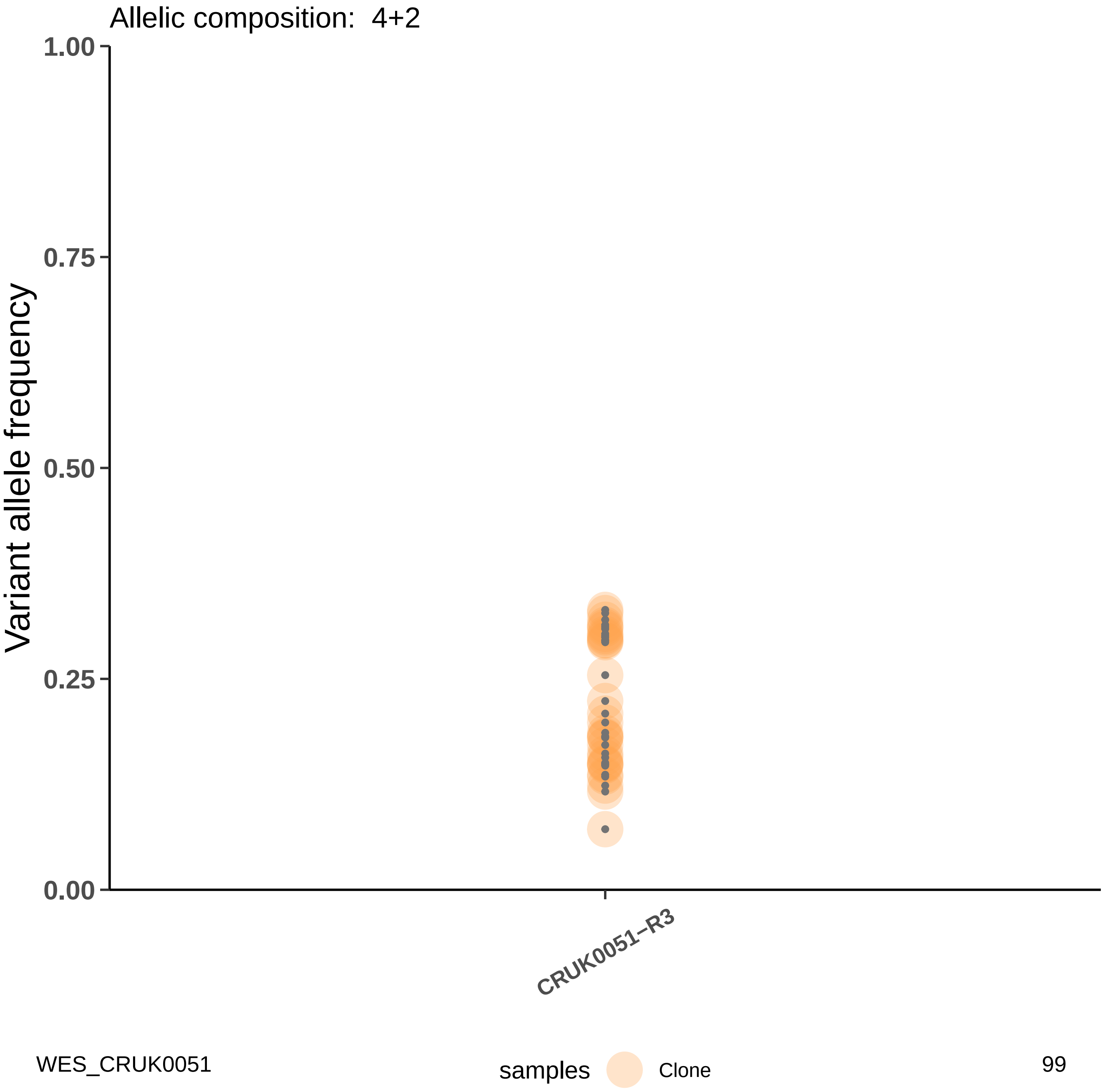

WES_CRUK0051

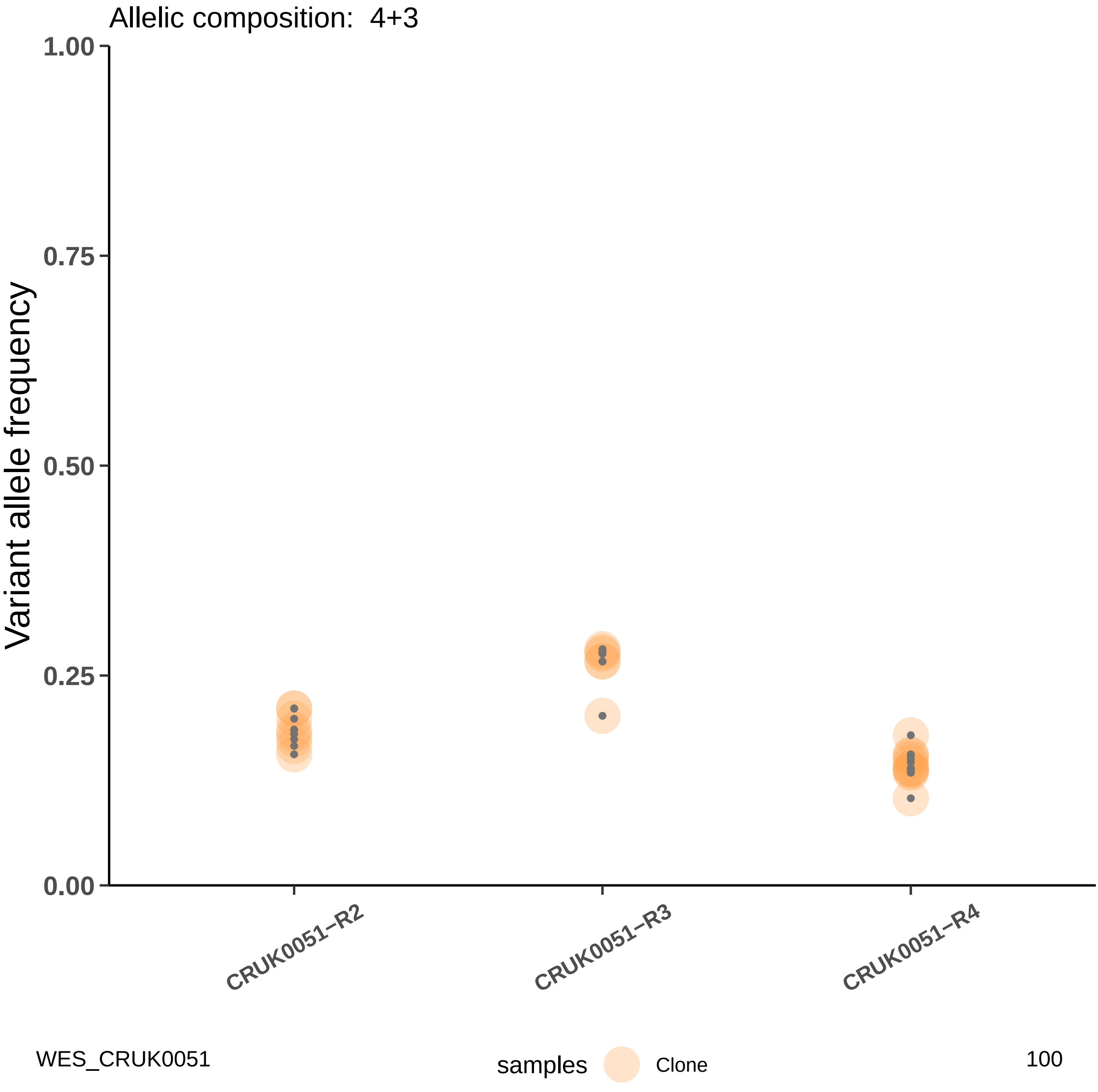

WES_CRUK0051

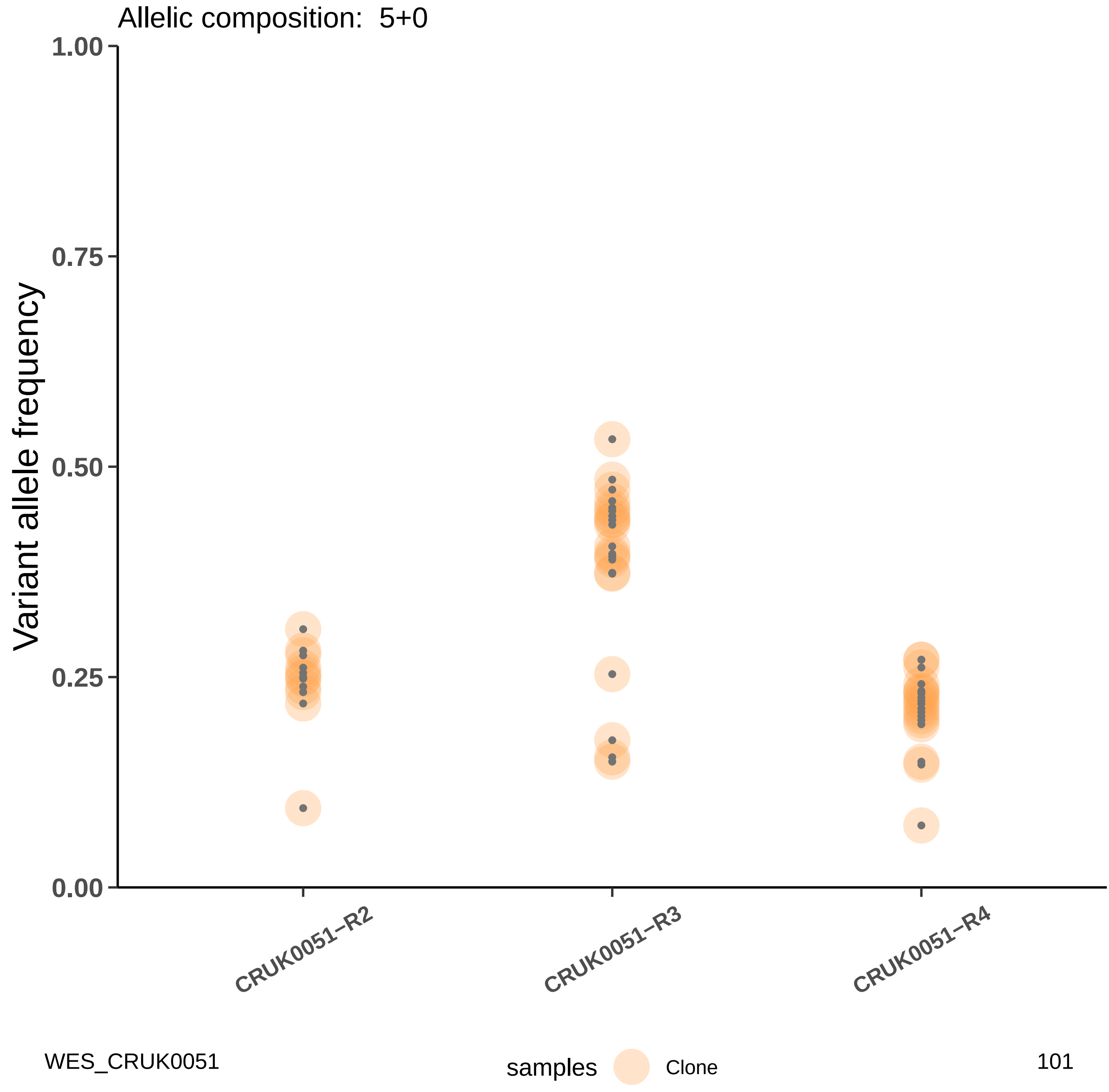

WES_CRUK0051

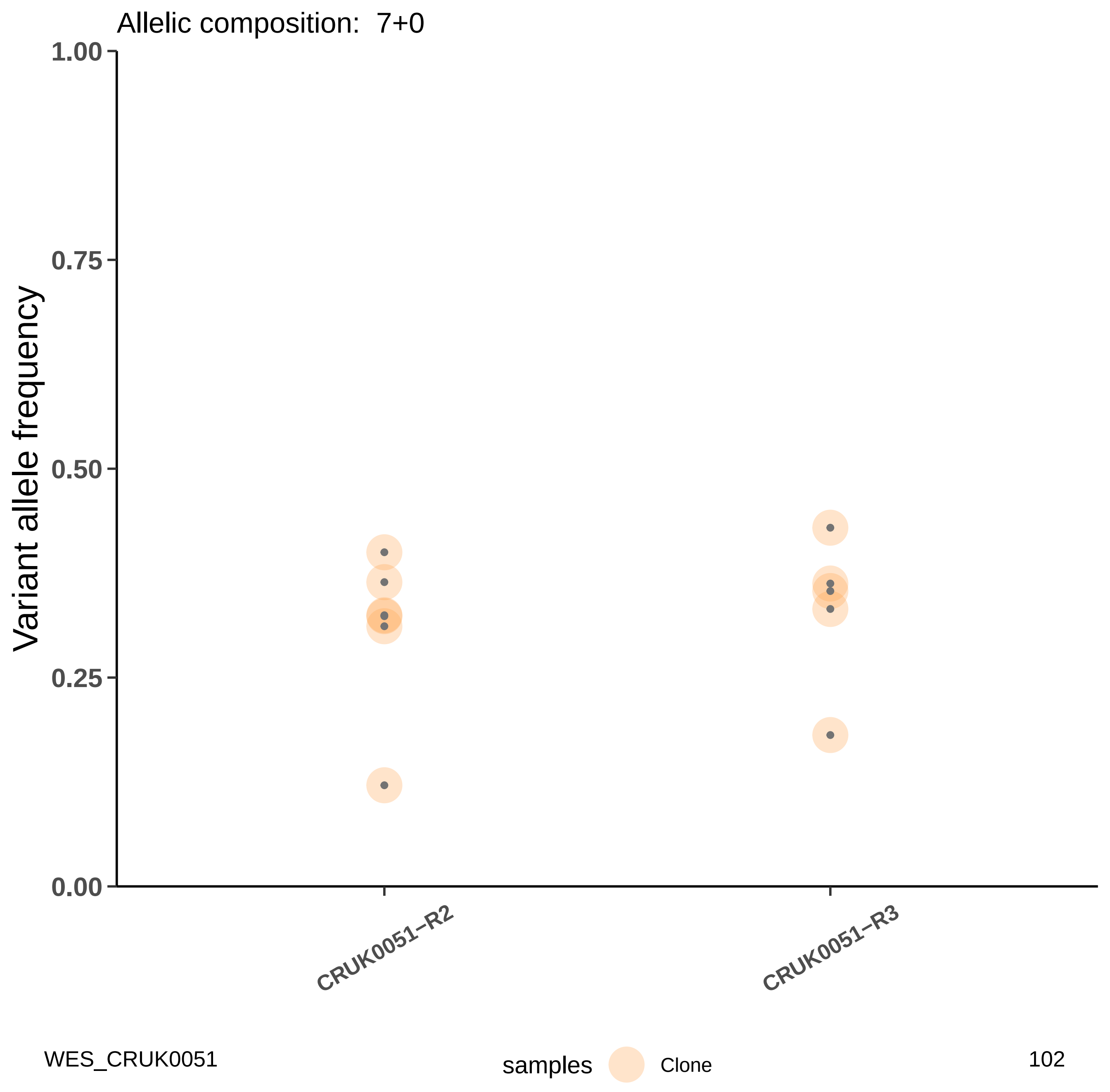

WES_CRUK0051

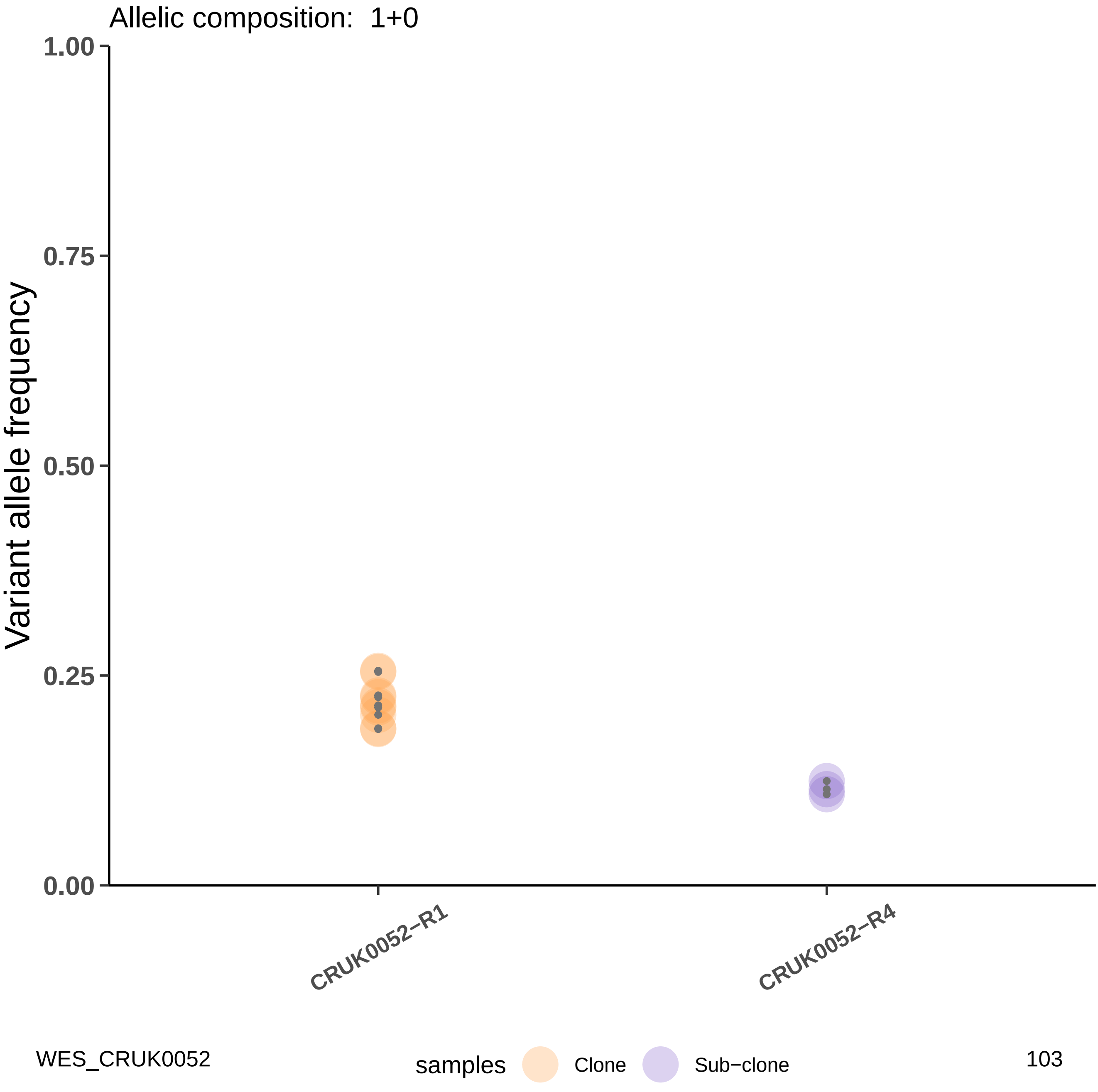

WES_CRUK0052

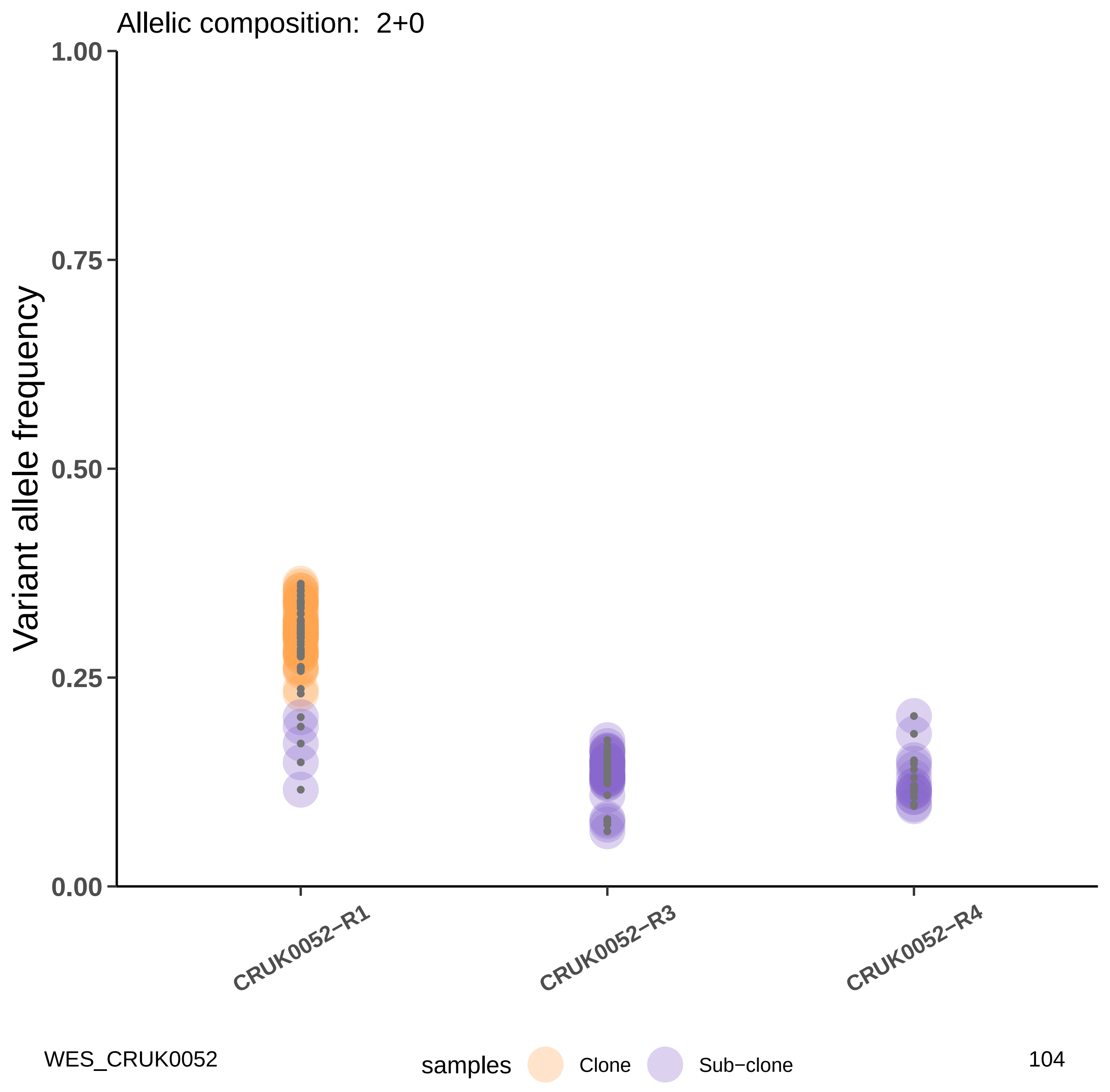

WES_CRUK0052

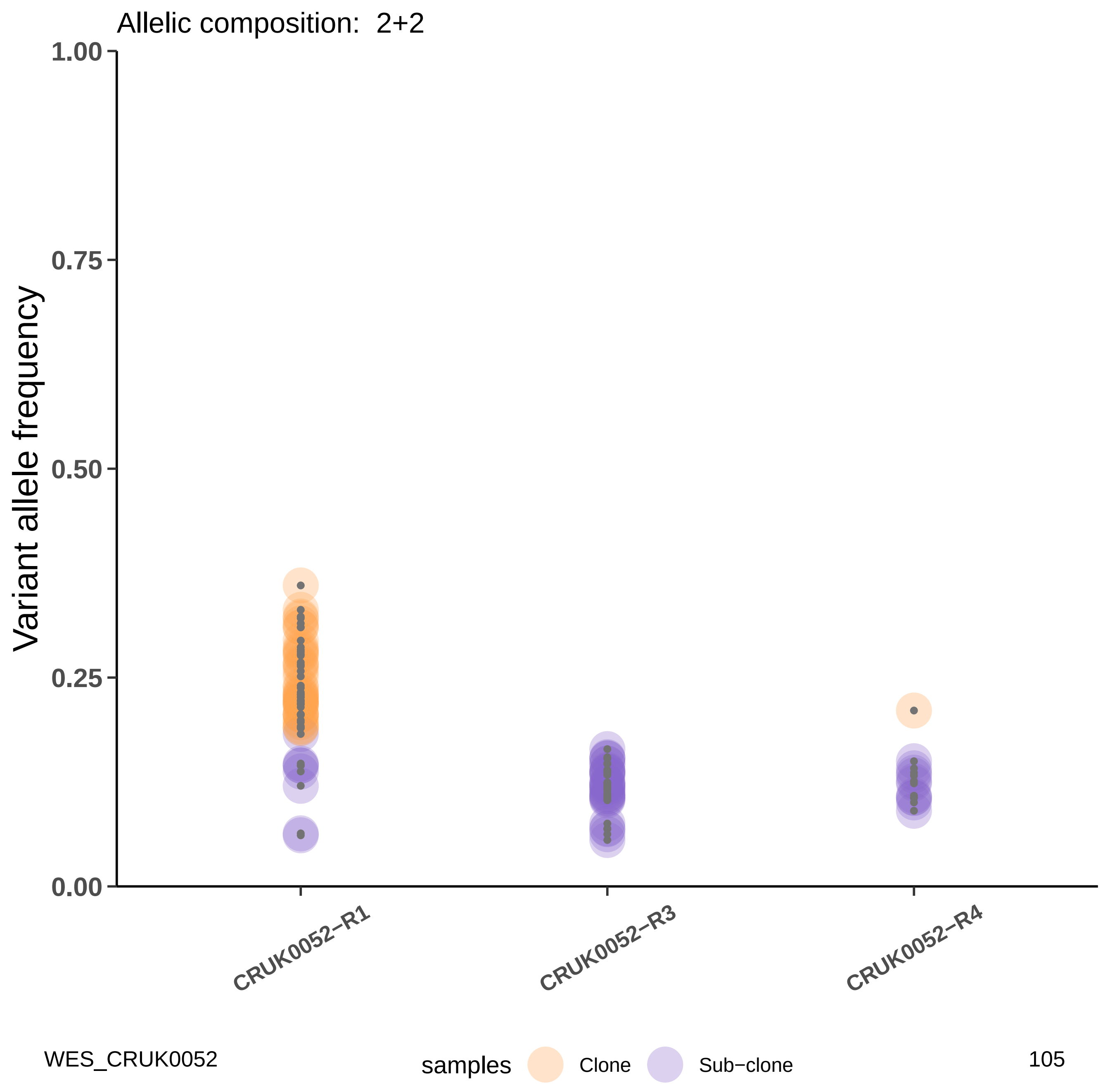

WES_CRUK0052

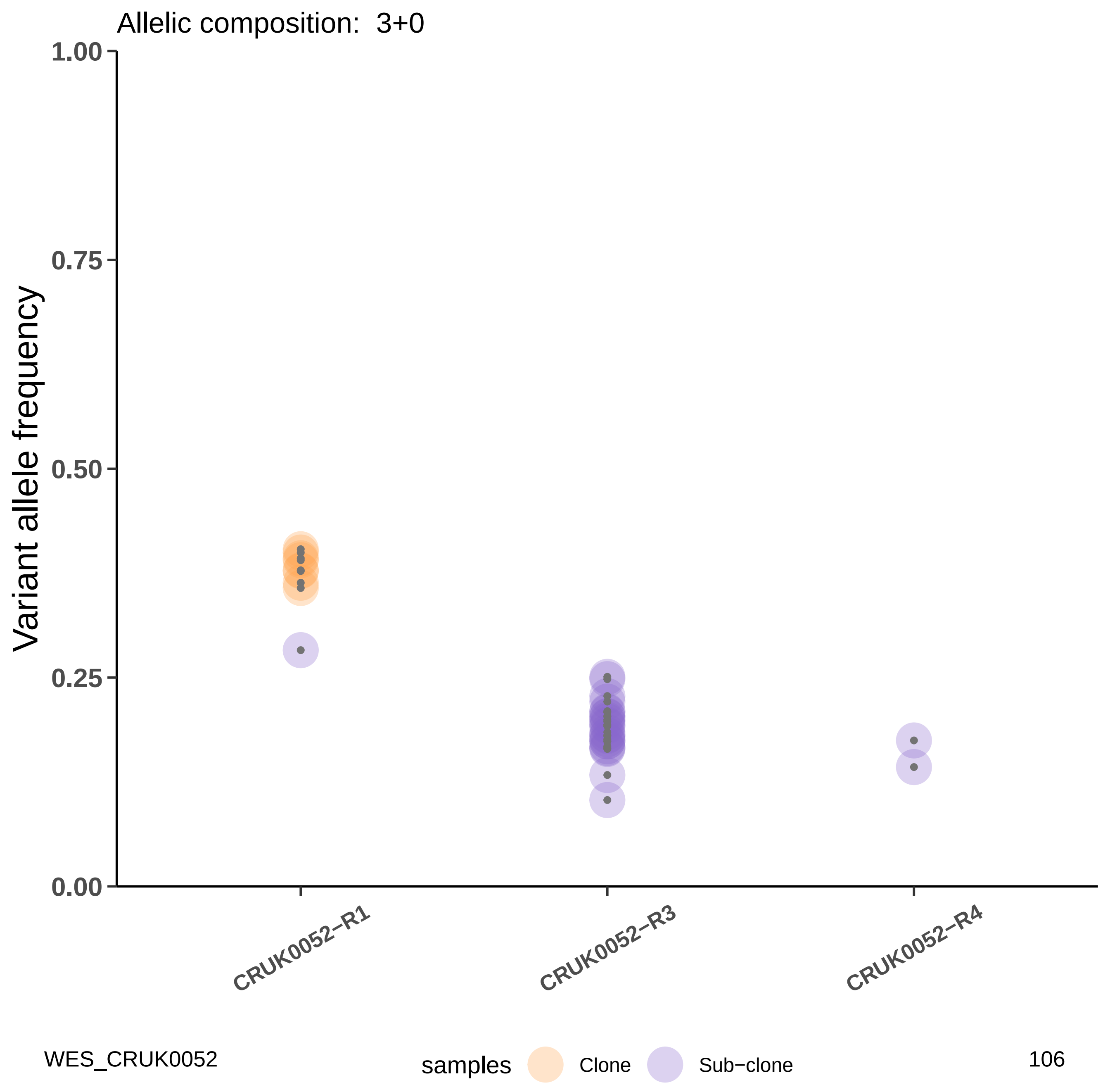

WES_CRUK0052

Allelic composition: 3+1

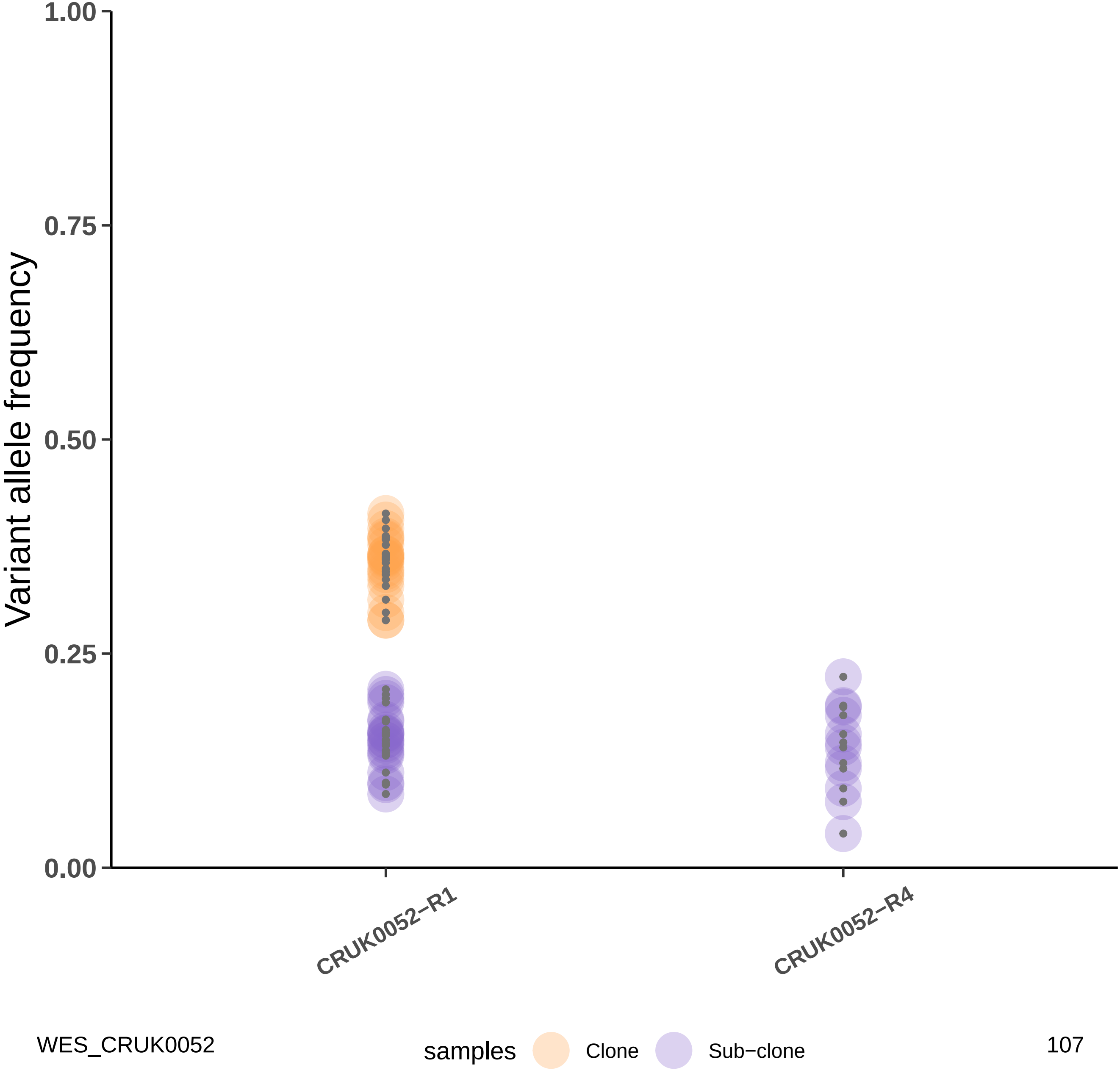

WES_CRUK0052

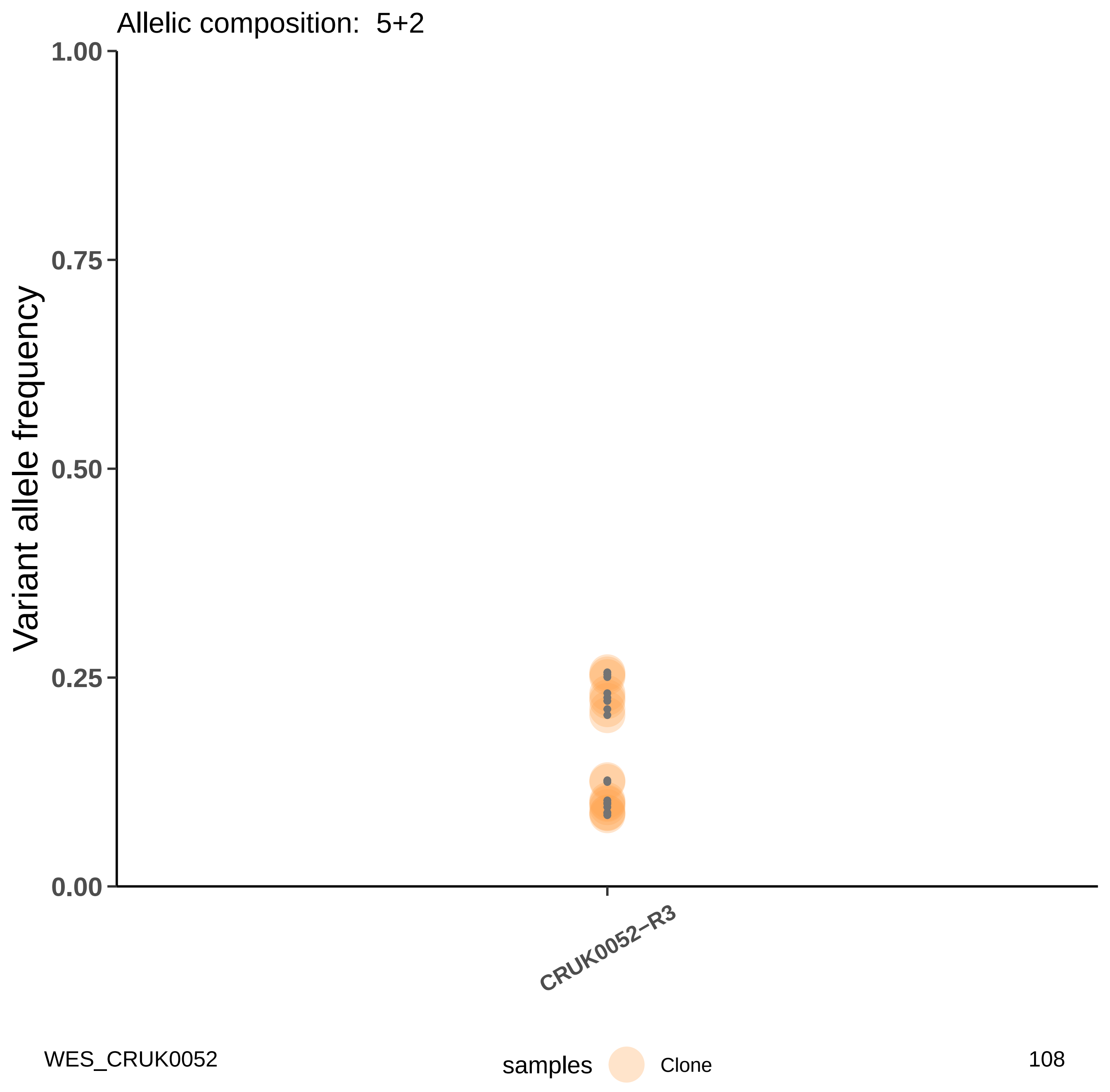

WES_CRUK0052

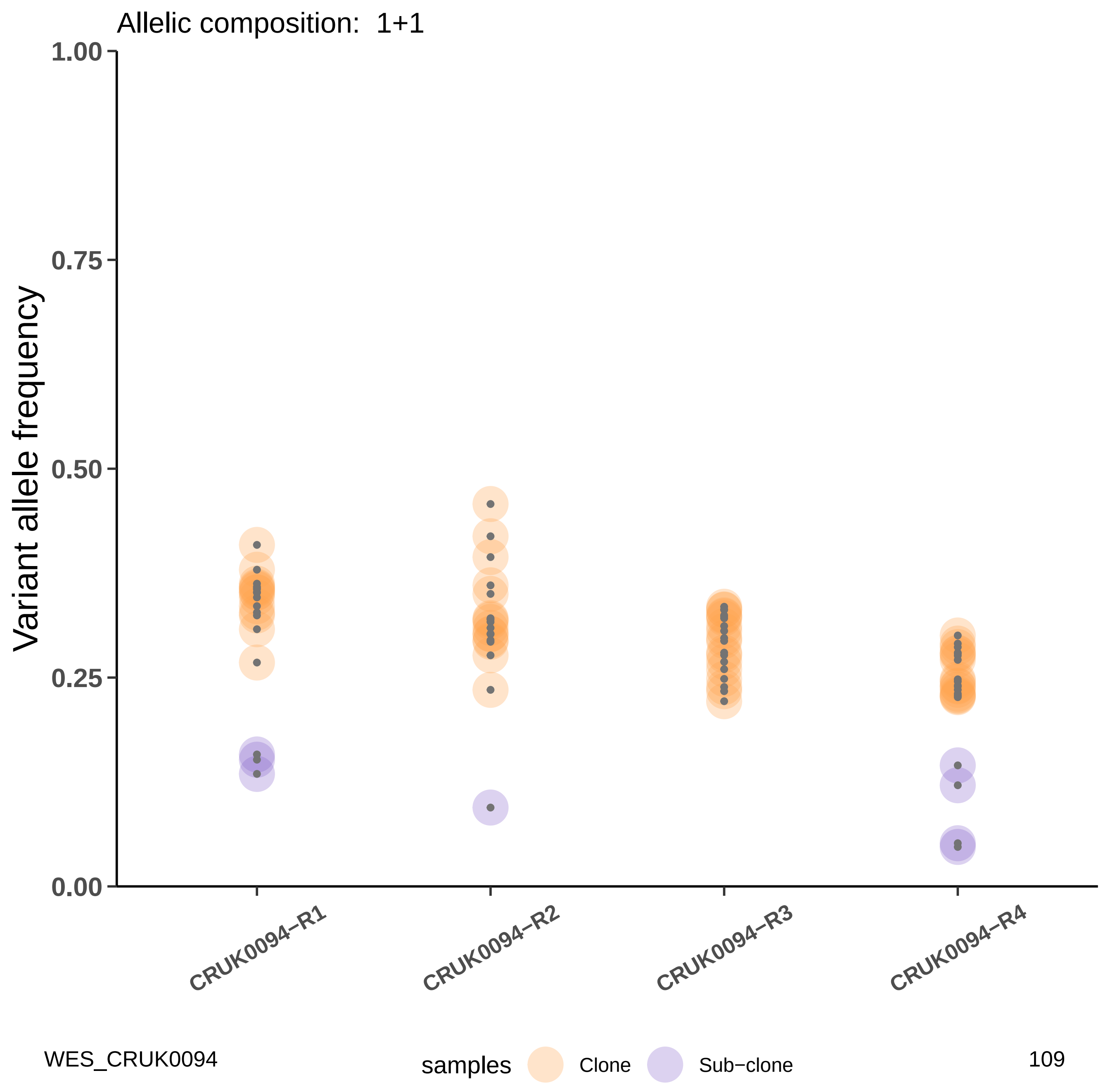

WES_CRUK0094
